# Epidemiology of a major honey bee pathogen, deformed wing virus: potential worldwide replacement of genotype A by genotype B

**DOI:** 10.1016/j.ijppaw.2022.04.013

**Published:** 2022-05-10

**Authors:** Robert J. Paxton, Marc O. Schäfer, Francesco Nazzi, Virginia Zanni, Desiderato Annoscia, Fabio Marroni, Diane Bigot, Eoin R. Laws-Quinn, Delphine Panziera, Christina Jenkins, Hassan Shafiey

**Affiliations:** a General Zoology, Institute for Biology, Martin Luther University Halle-Wittenberg, Hoher Weg 8, 06120, Halle (Saale), Germany; b German Centre for Integrative Biodiversity Research (iDiv) Halle-Jena-Leipzig, Puschstrasse 4, 04103, Leipzig, Germany; c Institute of Infectology Medicine, Federal Research Institute for Animal Health, Friedrich-Loeffler-Institut, Südufer 10, 17493, Greifswald, Insel Riems, Germany; d Dipartimento di Scienze AgroAlimentari, Ambientali e Animali, Università degli Studi di Udine, Via delle Scienze 206, 33100, Udine, Italy

**Keywords:** *Apis mellifera*, RNA, DWV, Iflaviridae, Evolution, Recombination

## Abstract

The western honey bee (*Apis mellifera*) is of major economic and ecological importance, with elevated rates of colony losses in temperate regions over the last two decades thought to be largely caused by the exotic ectoparasitic mite *Varroa destructor* and deformed wing virus (DWV), which the mite transmits. DWV currently exists as two main genotypes: the formerly widespread DWV-A and the more recently described and rapidly expanding DWV-B. It is an excellent system to understand viral evolution and the replacement of one viral variant by another. Here we synthesise published results on the distribution and prevalence of DWV-A and -B over the period 2008–2021 and present novel data for Germany, Italy and the UK to suggest that (i) DWV-B has rapidly expanded worldwide since its first description in 2004 and (ii) that it is potentially replacing DWV-A. Both genotypes are also found in wild bee species. Based on a simple mathematical model, we suggest that interference between viral genotypes when co-infecting the same host is key to understanding their epidemiology. We finally discuss the consequences of genotype replacement for beekeeping and for wild pollinator species.

## Highlights

• DWV vectored by *Varroa destructor* mites is the major cause of honey bee colony loss.
• DWV genotype B has largely replaced DWV genotype A in Europe.
• DWV genotype B is nowadays spread worldwide, expect Australia.
• Recombinational meltdown might explain why genotype B replaces genotype A.
• Replacement of DWV-A by DWV-B may exacerbate colony loss and pathogen spillover.

## Graphical abstract

**Figure:**
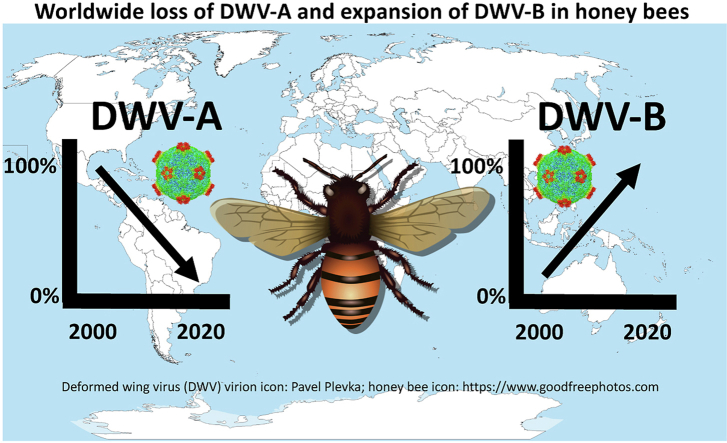

## 1 Introduction

Emerging infectious diseases (EIDs) are a cause of increasing concern to the health of wildlife, domestic animals and humans (Daszak et al., 2000). RNA viruses feature prominently as the aetiological agent in many of these cases (Jones et al., 2008); examples include squirrel pox in squirrels (Tompkins et al., 2003), African swine fever in pigs (Sauter-Louis et al., 2021) and the ongoing Covid-19 epidemic in humans (Li et al., 2021). Coronavirus SARS-CoV-2 that causes Covid-19 disease in humans has been the focus of intense scrutiny as it has swept through human populations since its first detection in late 2019 in Wuhan, China. Its epidemiology has been characterised by the emergence of novel variants with enhanced virulence or transmission that spread rapidly to replace former variants: Alpha, Delta (Li et al., 2021) and, most recently, Omicron (Mallapaty, 2022). Managed western honey bee (*Apis mellifera*) populations in most temperate regions of the world have witnessed elevated colony mortality for over the past two decades (reviewed in Osterman et al., 2021), arguably caused by deformed wing virus (DWV), an EID, which is vectored between honey bees by the exotic ectoparasitic mite *Varroa destructor* (Bowen-Walker et al., 1999). DWV is also widespread among wild bee species and other flower-visiting insects (Martin and Brettell, 2019; Nanetti et al., 2021), suggesting viral spillover from honey bees. Analogous to the worldwide spread of novel SARS-CoV-2 variants and their replacement of former variants in humans, evidence points to the worldwide spread of one DWV variant (genotype B) and its ongoing replacement of another, formerly prevalent DWV variant (genotype A) in the USA and UK (Ryabov et al., 2017; Grindrod et al., 2021; Kevill et al., 2021), and potentially elsewhere (Manley et al., 2019; see also Norton et al., 2021). Like SARS-Cov-2 and many other viruses, DWV represents a case of evolution in ecological time (McMahon et al., 2018).

*Apis mellifera* is the world's most important managed pollinator (Osterman et al., 2021). It has been transported across terrestrial biomes, where it plays an important role in its native and introduced range as a crop pollinator (Klein et al., 2007) and flower visitor of wild plant species (Hung et al., 2018). Following introduction to eastern Asia and contact with the native eastern honey bee *Apis cerana*, *A. mellifera* acquired the *V. destructor* mite from *A. cerana*, its presumed original host (reviewed in Rosenkranz et al., 2010; Nazzi and Le Conte, 2016; Traynor et al., 2020). The mite has subsequently become distributed more-or-less worldwide in the last 70 years as an ectoparasite of *A. mellifera* (Traynor et al., 2020), presumably through trade in *A. mellifera* colonies and queens. Australia is the only major landmass that harbours *A. mellifera* colonies that are currently free of *V. destructor*.

DWV was a rarely detected virus before the arrival of *V. destructor* into *A. mellifera* populations (Allen and Ball, 1996). It remains at low prevalence in populations of *A. mellifera* devoid of *V. destructor* e.g. northern Sweden (Forsgren et al., 2012) and Newfoundland (Shutler et al., 2014). After *V. destructor* first invades a population of honey bees, DWV prevalence and load (titre per bee) increase dramatically, as on Hawaii (Martin et al., 2012) and New Zealand (Mondet et al., 2014). The mite acts as a very efficient vector of the virus (Gisder et al., 2009; Möckel et al., 2011; reviewed in Yañez et al., 2020) and DWV has thereby become ubiquitous across most of the native and introduced range of *A. mellifera* (Grozinger and Flenniken 2019; Martin and Brettell 2019; Beaurepaire et al., 2020). For example, a survey of US honey bees detected DWV in most colonies (Traynor et al., 2016), suggesting that it is likely present in all colonies infested by *V. destructor*. Though there is dispute as to whether DWV is present in Australian honey bees (Wilfert et al., 2016; Roberts et al., 2017), if present, then it must be at negligible prevalence or at the limit of assay detection. Australia can therefore be seen as an outlier with regard to DWV, presumably because its honey bees are devoid of *V. destructor*.

DWV has become the best-studied honey bee virus (McMenamin and Flenniken, 2018; Grozinger and Flenniken, 2019; Martin and Brettell, 2019) because of its omnipresence in *A. mellifera* colonies across most of the world and because it (and its vector, *V. destructor*) are closely tied to colony decline (Dainat et al., 2012; Nazzi et al., 2012; Francis et al., 2013; Natsopoulou et al., 2017; Annoscia et al., 2017, 2019). Deformed wing virus is a positive single stranded RNA (+ssRNA) Picorna-like virus in the family *Iflaviridae*. It is known that DWV variants recombine and several unique recombinants have been reported (Moore et al., 2011; Zioni et al., 2011; Wang et al., 2013; Ryabov et al., 2014; Dalmon et al., 2017; Barroso-Arévalo et al., 2019; Brettell et al., 2019, 2020; Daughenbaugh et al., 2021). First detected in 1982 (Bailey and Ball, 1991) and fully sequenced by Lanzi et al. (2006), phylogenetic evidence based on three gene sequences has shown the original variant (genotype A) of DWV to have a worldwide distribution with a presumed European origin (Wilfert et al., 2016). Ongus et al. (2004) described a novel viral variant, *Varroa destructor virus-1* from *V. destructor* in the Netherlands, which has subsequently been synonymised with DWV and renamed DWV genotype B (DWV-B) (McMahon et al., 2016) to differentiate it from the originally described variant: DWV genotype A (DWV-A). These two genotypes exhibit ca. 16% sequence divergence (Ongus et al., 2004), well within the typical range of intraspecific sequence differences for viruses (Bobay and Ochman, 2018). A third variant has since been described, DWV-C (Mordecai et al., 2016), though unlike DWV-A and DWV-B it is rarely reported. A fourth variant, DWV-D, has recently been recovered from Egyptian honey bees collected in the 1970s but has never been reported since (de Miranda et al., 2022).

That most DWV sequence records are DWV-A (Berényi et al., 2007; Wilfert et al., 2016) suggests that DWV-A is indeed a long-standing and widespread pathogen of *A. mellifera* across the world (potentially excepting Australia). DWV-B's first description in Dutch *V. destructor* (Ongus et al., 2004) suggests that it was new to *A. mellifera* or geographically restricted in distribution, though its absence pre-2004 might also represent under-recording. Since Ongus et al. (2004), DWV-B has been reported from a growing number of countries e.g. Israel (Zioni et al., 2011), France (Gauthier et al., 2011), and Germany (Wilfert et al., 2016), and it appears to be increasing in prevalence on mainland USA (Ryabov et al., 2017), Hawaii (Grindrod et al., 2021) and the UK (Kevill et al., 2021), potentially driving down DWV-A's prevalence. That DWV-A DWV-B recombinants have frequently been detected using ultradeep sequencing technologies (Moore et al., 2011; Zioni et al., 2011; Wang et al., 2013; Ryabov et al., 2014; Dalmon et al., 2017; Barroso-Arévalo et al., 2019; Brettell et al., 2019, 2020; Daughenbaugh et al., 2021) means that reporting of a genotype's prevalence on the basis of a small region of the viral genome, as often performed in qPCR-based studies, does not capture the full genetic diversity of a viral population and may obscure the role that recombination itself plays in competitive interactions among co-infecting viral genotypes.

Two important traits of a pathogen that dictate its epidemiology are its virulence (ability to cause harm to a host) and its transmission, both of which are often correlated with pathogen titre (Schmid-Hempel, 2011). DWV is associated with *A. mellifera* colony decline and is widespread in honey bee populations, suggesting it has both high virulence and high transmissibility. But the relative virulence and transmissibility of its genotypes A and B are a point of disagreement in the literature, though fundamental for understanding their dynamics.

In a longitudinal study of three UK colonies over one year, Mordecai et al. (2015) found that DWV-A was replaced by DWV-B and suggested that DWV-B was benign or of low virulence, protecting colonies from the more virulent DWV-A (described as a superinfection exclusion event). DWV-B was subsequently found to exhibit higher virulence in laboratory experiments with adult honey bees, likely due to the faster rate of replication of DWV-B over DWV-A and the higher titres that DWV-B reached over DWV-A before adult mortality (McMahon et al., 2016). Subsequent experimental comparison of the virulence of DWV-A versus DWV-B (Tehel et al., 2020; Al Naggar and Paxton, 2021) have supported the initial demonstration of higher virulence and viral loads reached by DWV-B over DWV-A in adult honey bees. However, these experiments need to be repeated by other research groups to gauge whether they are applicable across isolates of DWV genotypes, subspecies of honey bee and climatic zones. Experiments comparing the impact of DWV-A and DWV-B on honey bee pupae suggest that DWV-B exhibits the same (Tehel et al., 2019) or apparently lower (Norton et al., 2020) virulence compared to DWV-A despite higher viral titres of DWV-B over DWV-A in both studies; interpretation of pupal mortality in the latter study is complicated by the termination of the experiment before adult eclosion. The field lacks experimental tests of the virulence of DWV-A and DWV-B for entire colonies of *A. mellifera*. We conclude from the experimental evidence of impacts on individual bees that DWV-B likely has an equal or somewhat higher virulence than DWV-A.

The primary mode of transmission of DWV between honey bees within a colony is horizontal, primarily vector-based, by *V. destructor* (Gisder et al., 2009; Möckel et al., 2011; reviewed in Yañez et al., 2020); when a colony is heavily infected, DWV-contaminated larval food (Yue and Genersch, 2005) and cannibalism of DWV-infected pupae by adult *A. mellifera* as well as trophallaxis contribute to its horizontal transmission (Posada-Florez et al., 2021). Transmission of DWV between colonies has not been explicitly researched but is presumably also primarily mite-based, accompanying the between-colony dispersal of *V. destructor* phoretically on honey bees. Such mite influx into colonies is often very high in the temperate autumn (Frey and Rosenkranz, 2014). For example, Greatti et al. (1992) found as many as 70 mites entering a *V. destructor*-free colony per day during the temperate autumn, when robbing (theft of honey from a colony by members of another colony) is high and colonies often have high mite loads and viral titres (e.g. Natsopoulou et al., 2017).

In terms of differential transmission, then, the question is whether *V. destructor* is more likely to transmit DWV-B than DWV-A. Gisder and Genersch (2021) recently stated that DWV-B can replicate within *V. destructor* but that DWV-A cannot, suggesting that DWV-B may have a higher rate of transmission than DWV-A when vectored by *V. destructor* mites. However, in their first description of DWV-B, Ongus et al. (2004) presented evidence of replication within the mite (i.e. presence of the negative-sense RNA strand) of both DWV genotypes by genotype-specific PCR primer sets. Later, Annoscia et al. (2019) demonstrated viral replication within most mites used in their experiments at a time when DWV-A predominated (we confirm DWV-A's predominance in section 3.2). More recently, Posada-Florez et al. (2019) has suggested that the presence of the negative-sense strand of DWV-A in mites could be related to its acquisition from host pupae upon which they were feeding and not related to replication in the mite *per se*. In support of DWV-A replicating in mites, Annoscia et al. (2019) did not find a correlation between the viral load of a mite and the viral load of the host bee upon which it had fed, suggesting that high viral loads in a mite (and negative-sense viral RNA) were dependent on viral replication in the mite and not from its source host. Further studies addressing DWV-A's ability to replicate in *V. destructor* are needed to achieve clarity on this topic.

DWV genotypes may possess other genotype-specific traits that permit differential transmission via *V. destructor*. A recent colony-level field experiment that manipulated *V. destructor* infestation in the presence of DWV-A and DWV-B found that DWV-B reached higher titres and dominated over DWV-A (Norton et al., 2021), attributed to the adaptation of DWV-B to enhanced transmission by the mite. The higher titres that DWV-B reach in pupae (Tehel et al., 2019; Norton et al., 2020) and adult honey bees (McMahon et al., 2016; Tehel et al., 2020; Al Naggar and Paxton, 2021) following experimental infection compared to DWV-A mean that DWV-B is more likely to be acquired by a *V. destructor* mite feeding upon an infected host, further supporting the notion that DWV-B has a higher rate of transmission compared to DWV-A. Moreover, pupal infection experiments with cloned DWV-variants suggest that DWV-B has a slightly higher rate of replication than DWV-A in the 24 h following inoculation (Gusachenko et al., 2021). The evidence therefore points to DWV-B being more transmissible than DWV-A via *V. destructor*, both within and between colonies. This conclusion notwithstanding, it should be stressed that genotype A is also very efficiently vectored by *V. destructor* (Yañez et al., 2020). Interestingly, DWV-A/B recombinants may exhibit even higher virulence and transmission in comparison to one or both parental genotypes (Moore et al., 2011; Ryabov et al., 2014).

Here, by examining published (sequence database and literature) and own (novel) data, we review the idea that DWV-B is expanding its range and replacing DWV-A. We then describe the spread of DWV genotypes in an epidemiological model to highlight a critical aspect of the interaction among competing viral variants, namely interference, whose elucidation will allow better prediction of the outcome when two viral variants coinfect the same host. We conclude that there is overwhelming support for the view that DWV-B is spreading at the expense of DWV-A, at least in temperate regions of the world, whilst interference between genotypes in coinfection is likely critical to their epidemiology.

## 2 Material and methods

### 2.1 DWV-A and DWV-B in publicly available transcriptome datasets

To gain an unbiased insight into the relative occurrence of DWV-A and DWV-B, we interrogated NCBI's Sequence Read Archive (SRA) for entries up to December 31, 2021. As it is a poly-A tailed RNA virus, DWV is captured in next generation sequence (NGS) ‘transcriptome’ libraries that target the sequencing of, or are enriched for, eukaryote mRNA (Gerth and Hurst, 2017). We searched the publicly accessible NCBI SRA for DWV sequences in submissions of transcriptome datasets (NGS reads) generated for (i) the honey bee (*A. mellifera*), (ii) *V. destructor* ectoparasites and vectors of DWV among honey bees, and (iii) a bumble bee (*Bombus terrestris*) common to Europe, North Africa and the western Asia that has often been reported to harbour DWV (e.g. Fürst et al., 2014). Organism search terms were the species names (in Latin) and the strategy search term was RNAseq.

To avoid an upward or genotype-specific bias in viral representation in NGS libraries, we manually excluded *A. mellifera* datasets whose metadata suggested they were generated as part of dedicated infection experiments with DWV or *V. destructor*. Following Cornman (2017), we took the date of submission minus 2 years for libraries lacking a collection date. Libraries with less than 10,000 total reads were discarded.

After filtering, 2990 libraries (2538 libraries for the honey bee, 99 libraries for *V. destructor* and 353 libraries for *B. terrestris*) remained for analysis, which were naïvely mapped to the NCBI reference genomes of DWV-A (NC_004830) and DWV-B (NC_006494) using hisat2 v. 2.2.1 (Kim et al., 2019) with default settings for the 1432 paired-end (PE) libraries and Bowtie2 (Langmead and Salzberg, 2012) on the trimmed 1558 single-read (SR) libraries, discarding those reads with mapping quality less than 10. These mapping and quality criteria ensured that each read mapping to DWV (either DWV-A or DWV-B) could be uniquely assigned to either DWV-A or DWV-B, when reads that mapped to both DWV-A and DWV-B with equal or comparable likelihood (representing <5% of mapping reads) were removed from the dataset. The number of reads mapped to either DWV-A or DWV-B were then counted (Supplementary Table S1). For each year, the absolute percentage of each virus was defined as the total number of reads of that genotype divided by the total number of reads of DWV (both genotypes) for that time interval. There were <10 libraries per year for 2004 and 2009 and so they were discarded for visualisation of the data.

### 2.2 First records of DWV-B in the published literature

To determine when DWV-B was first recorded in honey bees in a country, we carried out a systematic review of the literature by searching Web of Science with the terms “Deformed wing virus” AND “genotype B″ OR “DWV-B″ OR “VDV-1” for the years 2000–2021 inclusive, following PRISMA guidelines (Page et al., 2021). We generated 58 articles, which we then examined to determine host species, geographic locality and date of collection of the insects harbouring DWV-B. We augmented records with others known to us (expert knowledge) that these search terms had not highlighted.

For each country, we then selected the publication with the earliest collection record for DWV-B and, if there were two or more publications with the same year of collection for a country, the earliest publication. Though not an exhaustive search, it led to 29 independent records (Supplementary Table S2) of countries or regions (Hawaii and Continental USA were split as geographically independent regions) in which DWV-B was first detected in *A. mellifera* or *V. destructor*. Though these 29 records only represent the first known occurrence of DWV-B at a locality in honey bees or *V. destructor*, they are nevertheless instructive in providing a pattern of spread of the genotype across the world. For each publication, we also extracted information on the prevalence of DWV-A and DWV-B, when given.

Our approach suffers from under-recording because DWV is primarily detected by PCR/qPCR and variant detection depends on the choice of PCR oligonucleotide primers. PCR primers that amplify both DWV-A and DWV-B with similarly high efficiency are often employed in pathogen screening of honey bees and other insects (e.g. Pislak Osterman et al., 2021), which do not permit genotype designation unless combined with Sanger or NGS sequencing of PCR products (e.g. as in Fürst et al., 2014).

On the other side, DWV-B may be over-recorded because of false positives. Genotype designation with DWV-B specific primers is typically based on the qPCR amplification of a small region of the DWV genome without sequencing the PCR product to confirm genotype identity (see Supplementary Table S2). PCR artefacts might lead to ‘false positives’, though a melt curve analysis post-qPCR should overcome this by confirming PCR product identity as a peak at the expected melt temperature (Bustin et al., 2009).

An additional caveat of the data is that recombinants between DWV-A and DWV-B cannot be recognised unless the isolate is sequenced at multiple loci across the genome or is subject to NGS-sequencing (for an exemplary study combining qPCR and NGS, see Ryabov et al., 2017). Strictly speaking, studies employing PCR of a single genomic region for determining viral genotype can only state that the region amplified was DWV-A or DWV-B. We therefore add additional information to Supplementary Table S2 when papers confirmed genotype identity (e.g. by Sanger sequence of a PCR product or by NGS-based whole genome consensus sequence assembly of the isolate).

### 2.3 Temporal change in the prevalence of DWV-A and DWV-B

Understanding the dynamics of DWV genotypes within a population requires temporal sampling and DWV genotype-specific screening of that population at two or more time points. The published literature provides three cases: continental USA (Ryabov et al., 2017), Hawaii (Grindrod et al., 2021) and mainland United Kingdom (Kevill et al., 2021). Here we complement these data with three new, independent, temporal datasets from mainland UK, Germany and northeast Italy.

#### 2.3.1 Mainland UK

Our data represent DWV-genotypes of individual honey bees collected at flowers from the same seven UK sites in 2011 (n = 55 honey bees) and 2017 (n = 106 honey bees).

In 2011, honey bees were collected at flowers from 26 sites across the UK as part of a study addressing DWV spillover between *A. mellifera* and *Bombus* spp. (Fürst et al., 2014). DWV genotypes of DWV-positive samples were determined by qPCR using genotype-specific primers targeting the *RdRp* gene (McMahon et al., 2015); data are presented in McMahon et al. (2016). In 2017, we re-sampled honey bees at flowers from seven of the 26 sites (Supplementary Fig. S1) as in Fürst et al. (2014) and analysed them for DWV genotype using the same primers and methods of RNA extraction, cDNA synthesis and qPCR as in McMahon et al. (2016); methodological details are provided in full in Tehel et al. (2019). These methods adhere to qPCR best practice (MIQE guidelines, Bustin et al., 2009) and include use of positive and negative controls on each qPCR plate, amplification of a honey bee reference gene (*ß-Actin*), technical duplication, post qPCR melt curve analysis and use of a Cq (quantification cycle) threshold of 35. Data are provided in Supplementary Table S3.

#### 2.3.2 Germany

Our data represent DWV-genotypes of a pooled sample of 10 honey bees (n = 1 pool per colony) collected from collapsing colonies in 2013 (n = 70 colonies) and 2019/2020 (n = 152 colonies) from across Germany.

As part of the governmental support of beekeeping in Germany, beekeepers can send a sample of honey bees from colonies that unexpectedly decline in workforce for pesticide residue and pathogen testing to help determine the cause of decline. We have retrospectively re-analysed RNA extracts of 2013, 2019 and 2020 samples for DWV-A and DWV-B using a TaqMan-based qPCR assay, exactly as described in Dittes et al. (2020). We combine 2019 and 2020 samples for ease of interpretation of temporal trends in genotype prevalence. Data and qPCR primer and probe sequences are provided in Supplementary Table S4.

#### 2.3.3 Northeast Italy

Our data represent Illumina reads of NGS transcriptome libraries prepared from the RNA of honey bees used in University of Udine experiments from 2009 to 2013 (68 libraries) and 2018–2020 (35 libraries).

Samples for both time periods comprised a mix of honey bees that either had or had not been parasitised by *V. destructor* mites. For the 2009–2013 datasets, RNA was derived from pooled honey bees (n = 11 pools each of 10 honey bees) or individual honey bees (n = 57 honey bees), totalling 6.13 x 10^8^ NGS reads and already published in Nazzi et al. (2012), Annoscia et al. (2017, 2019) and Zanni et al. (2017); these publications give full details of library preparation and NGS data analysis. For the 2018–2020 datasets, RNA was derived from individual honey bees (n = 35 honey bees), totalling 16.19 x 10^8^ NGS reads. These represent unpublished data that have been analysed using the same bioinformatics pipeline applied to the 2009–2013 datasets. Raw data are provided in Supplementary Table S5.

After removing reads mapping to *A. mellifera*, those uniquely mapping to DWV-A, DWV-B or DWV-C using STAR (Dobin et al., 2013) were retained for further analysis. Each library was defined as containing DWV-A or DWV-B if the number of reads uniquely mapping to that genotype was >500. The prevalence of DWV-A and DWV-B was then calculated at the level of the library. By doing so, we treat each library as a separate unit of replication. The alternative approach would be to count each read of each library. Though a valid approach, our other novel data and published (literature) data present prevalence in independent samples (individual honey bees or individual colonies) as a binary (DWV-A present: yes/no; DWV-B present: yes/no). For consistency in data presentation, we therefore present northeast Italy data at the level of the NGS library (yes/no).

DWV-C reads were detected in some of the 2009–2013 datasets as a low proportion (0.11%) of all reads mapping to DWV (Supplementary Table S5). It was not detected in any of the 2018–2020 libraries and therefore data relating to DWV-C are not presented graphically here.

### 2.4 Epidemiological model describing the dynamics of viral genotypes

To help interpret the pattern of spread of DWV-B and its potential replacement of DWV-A in a honey bee population, we developed a deterministic epidemiological model to describe the dynamics of two viral variants in continuous time within an infinitely large population of colonies. Ours is a form of compartmental model in which there are four compartments: susceptible and three different states of infected colonies.

We firstly define a colony as being in one of the four states:

• H (healthy, more accurately defined as uninfected by either DWV genotype),
• A (infected only with DWV-A),
• B (infected only with DWV-B), or
• M (mixed), when infected by both DWV-A and DWV-B.

Symbols A, B and M are used to indicate both the state of colonies and their frequency in the population. We initiated simulations by setting the frequency of colonies infected by DWV-A at 0.3 (A = 0.3) and those infected by DWV-B at 0.01 (B = 0.01) to reflect a population of honey bee colonies in which DWV-A predominates and is first invaded by DWV-B, the most plausible real-life scenario (Ryabov et al., 2017; Grindrod et al., 2021; Kevill et al., 2021).

Secondly, we assigned a fatality rate (ν) to each colony state. Initially, we set ν_A_ = ν_B_ = ν_M_ = 0.03 i.e. all states in which a colony is infected with DWV (DWV-A, DWV-B or both) have a 3% increased rate of mortality over state H (uninfected by DWV-A or DWV-B) per model iteration. In simulations, we also varied ν_B_ and ν_M_ to explore the effect of elevated virulence of DWV-B over DWV-A, as observed in adult honey bees (McMahon et al., 2016).

Thirdly, we assign an intrinsic transmission rate (μ) to each viral genotype. We initially set μ_A_ = 0.1 and μ_B_ = 0.15 to reflect the higher titre, and presumably higher transmission, of DWV-B in infected adult and pupal honey bees (McMahon et al., 2016; Tehel et al., 2019; Norton et al., 2020). We also doubled μ_B_ and ν_M_ to explore their impact on the frequencies of viral genotypes and colony states.

Finally, we define an interference term: m_A_, the extent to which genotype B influences the transmission rate of genotype A when both are present in a colony, whereby a positive value represents increasing transmission of DWV-A (cooperation; DWV-B enhances the transmission of DWV-A) and a negative value represents a decrease in transmission (DWV-B inhibits the transmission of DWV-A). The same is true for m_B_. We focus here on interference (negative values of m) because our empirical data (Section 3.3) suggest interference (potential elimination of DWV-A).

We can then specify the conditional transmission of DWV-A in the population in the presence of DWV-B as:

Equation 1 $$\mu_{A|B}=\mu_{A}+m_{A}\left(B+M\right)$$

Note that the conditional transmission rate is dependent on the frequency of colonies B and M. We could alternatively have defined the conditional transmission rate of DWV-A as a value independent of the frequency of DWV-B. However, under the reasonable assumption that DWV-B has the same distribution within each colony as among colonies, then frequency dependent transmission of A (and B) is a more plausible scenario.

The conditional transmission rate of DWV-B in the presence of DWV-A is the analogue of Equation (1) as:

Equation 2 $$\mu_{B|A}=\mu_{B}+m_{B}\left(A+M\right)$$

The change in frequency of A in the population of colonies is then given by:

Equation 3 $$\frac{dA}{dt}=\left(1-A-B-M\right)\left(A\mu_{A}+M\mu_{A|B}\right)-\left(M+B\right)A\mu_{B|A}-A\nu_{A}$$

in which the first term captures the conversion of colonies of state H to colonies of state A by DWV-A emanating from colonies of state A (DWV-A is transmitted from colony state A to colony state H at the rate of μ_A_) or M (DWV-A is transmitted from colony state M (or A) to colony state H (or B) at the rate of μ_A|B_, which depends on frequency of DWV-B in the population). The second term captures the conversion of colonies infected by DWV-A (A) to colonies of state M through acquisition of DWV-B, which is dependent on the conditional transmission rate of DWV-B. The third term is the death (loss) of colonies of state A. Our compartment model can therefore be interpreted as a form of SIR (susceptible-infected-removed) model in which infection by DWV-A or DWV-B ultimately leads to colony death (removal).

For DWV-B, the change in frequency of B in the population of colonies is given by the analogue of Equation (3) as:

Equation 4 $$\frac{dB}{dt}=\left(1-A-B-M\right)\left(B\mu_{B}+M\mu_{B|A}\right)-\left(M+A\right)B\mu_{A|B}-B\nu_{B}$$

The change in frequencies of M is then:

Equation 5 $$\frac{dM}{dt}=\left(1-A-B-M\right)M\mu_{A|B}\mu_{B|A}+A\left(M+B\right)\mu_{B|A}+B\left(M+A\right)\mu_{A|B}-M\nu_{M}$$

As Equations Equation 3, Equation 4, Equation 5) are non-linear, we used simulations with the package “*deSolve*” (Soetaert et al., 2010) in R v. 4.1.1 (R Core Team) to describe the dynamics of colony states and, thereby, also of DWV genotypes in a population of honey bee colonies across plausible parameter values.

Figures were generated in R v. 4.1.1 (R Core Team, 2021).

## 3 Results

### 3.1 DWV genotype representation in NGS datasets

Our interrogation of NCBI's SRA revealed 2990 RNAseq libraries (Supplementary Table S1) that we analysed. The majority of the libraries that had used *A. mellifera* as source material contained DWV reads (Supplementary Table S1). Approximately 5% of the 92 x 10^9^ reads in the *A. mellifera* libraries mapped to DWV, mostly to DWV-A (ca. 87% of all reads that mapped to either DWV-A or DWV-B) (Table 1).

**Table 1:** Publicly available NCBI NGS transcriptome reads of honey bees (*Apis mellifera*), a bumble bee (*Bombus terrestris*) and *Varroa destructor* mites mapping to DWV (either DWV-A or DWV-B) and the % of those DWV reads mapping uniquely to either DWV-A or DWV-B.

Host species	No. libraries	Total No. reads		As a % of all DWV reads	
			% DWV	% DWV-A	% DWV-B
*Apis mellifera*	2572	92403168213	5.26	86.79	13.21
*Bombus terrestris*	354	12110521624	<0.01	81.90	18.10
*Varroa destructor*	99	5193737105	36.68	61.56	38.44

Of the 99 RNAseq libraries that had used *V. destructor* as source material, approximately 37% of the 5 x 10^9^ reads mapped to DWV, predominantly to DWV-A (ca. 62% of all reads that mapped to either DWV-A or DWV-B, Table 1). Moreover, every *V. destructor* NGS library contained one or more DWV read (Supplementary Table S1), demonstrating that *V. destructor* mites typically harbour DWV and supporting the view that they are important vectors of the virus.

In the 12 x 10^9^ reads from 354 RNAseq libraries designated *B. terrestris*, <0.01% mapped to DWV, of which ca. 82% mapped to DWV-A (Table 1). Most *B. terrestris* libraries lacked DWV reads (Supplementary Table S1). These data reinforce the view that DWV-A and DWV-B are occasional viruses in this bumble bee species.

**Fig. 1:**
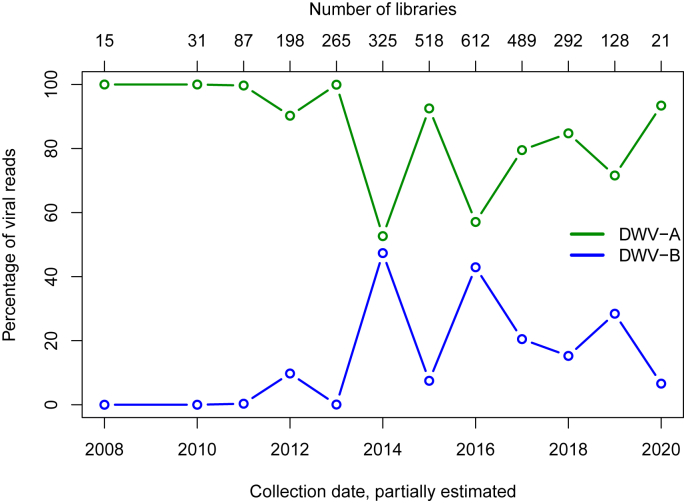
Relative proportion of DWV genotype A and B reads in publicly available NCBI transcriptome datasets of honey bees, *V. destructor* mites and bumble bees.

From 2008 to 2020, most DWV reads mapped to DWV-A (Fig. 1), reinforcing the view that DWV-A is, or was, the major genotype in honey bees, their *V. destructor* mites and bumble bees. After 2014, though, and despite considerable year-to-year variability, there seems to have been an increase in the relative representation of DWV-B in datasets (Fig. 1). Inter-annual variation in the global NCBI database may reflect the geographic source of sequenced material, which in any one year is often dominated by one or a few sequencing initiatives, each of which comprise multiple libraries. Splitting the *A. mellifera* dataset into periods up to 2015 and 2016 onwards, DWV-B rose from 78% to 84% in Europe and from 3% to 16% in USA (as a % of all DWV reads in a library) (Table 2), suggesting an ongoing expansion of DWV-B in prevalence or range and, in Europe, its dominance over DWV-A.

**Table 2:** Publicly available NCBI NGS transcriptome reads of honey bees mapping to DWV (either DWV-A or DWV-B) and the % of those DWV reads mapping uniquely to either DWV-A or DWV-B for Europe and USA for two time intervals, up to 2015 and after 2015.

Region	Time	No. libraries	Total No. reads		As a % of all DWV reads	
				% DWV	% DWV-A	% DWV-B
*Europe*	*<= 2015*	171	1940200000	21.52	22.3	77.7
*Europe*	*> (2015)*	106	2338637206	0.01	15.51	84.49
USA	≤ 2015	658	27879555219	11.26	96.9	3.1
*USA*	*> (2015)*	395	8626425643	8.72	83.96	16.04

Interestingly, the oldest NCBI SRA entry for DWV-B is in an RNAseq library of Jordanian honey bees collected in 2004 and comprising 35% DWV-A reads and 11% DWV-B reads (Supplementary Table S1, Bioproject PRJNA437728). DWV-B may have been long resident within *A. mellifera*'s native range in western Asia.

### 3.2 DWV-B spreads around the world

**Fig. 2:**
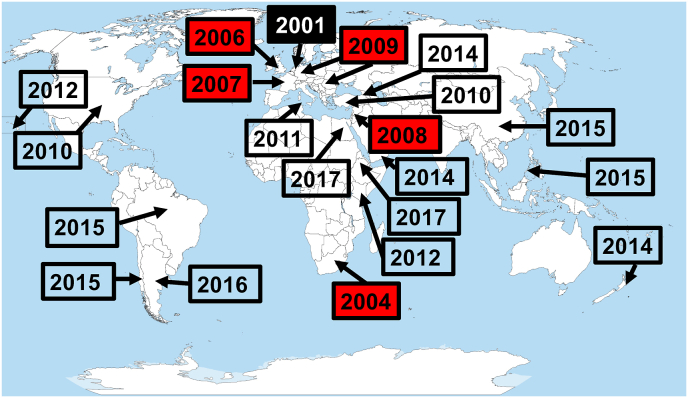
First published records of DWV genotype B in *Varroa destructor* (closed box) or in *Apis mellifera* (red boxes: pre-2010; open boxes: 2010 onwards) from a country or geographic region; citations are in Table 3. (For interpretation of the references to colour in this figure legend, the reader is referred to the Web version of this article.)

Following its first recording (and description) in Dutch *V. destructor* collected in 2001 (as VDV-1, Ongus et al., 2004), DWV-B's known distribution has enlarged rapidly to encompass all major landmasses of the world excepting Australia (Fig. 2). In Europe, it has been repeatedly detected in *A. mellifera* sampled before 2010 in the UK, France, Germany and Romania (Fig. 2, Table 3).

**Table 3:** First records of DWV-B from a country or region in the literature; see Supplementary Table S3 for additional details.

Year of collection	Country	Region	Host	n	DWV-A prevalence	DWV-B prevalence	Citation
2001	Netherlands	Europe	*Varroa destructor*	1 pool mites	present	present	Ongus et al. (2004)
2004	South Africa	Africa	*Apis mellifera*	1 colony	present	present	de Souza et al. (2021)
2006	England	Europe	*Apis mellifera*	14 colonies	100%	57%	Kevill et al. (2017)
2008	Israel	Asia	*Apis mellifera*	3 colonies	100%	100%	Zioni et al. (2011)
2007–2009	France	Europe	*Apis mellifera*	30 queens	100%	67%	Gauthier et al. (2011)
2009	Germany	Europe	*Apis mellifera*	15 bees	67%	73%	Wilfert et al. (2016)
2009	Romania	Europe	*Apis mellifera*	5 bees/mites	80%	40%	Wilfert et al. (2016)
2009–2010	Sweden	Europe	*Apis mellifera*	7 NGS libs.^a^	high	low	Thaduri et al. (2018)
2010	Turkey	Asia	*Apis mellifera*	6 sites	100%	100%	Tozkar et al. (2015)
2010	USA	N. America	*Apis mellifera*	75 colonies	100%	3%	Ryabov et al. (2017)
2011	Tunisia	Africa	*Apis mellifera*	56 colonies	27%	7%	Abdi et al. (2018)
2011	UK	Europe	*Bombus* spp.	490 workers	3%	8%	Fürst et al., 2014
2011–2012	Luxemburg	Europe	*Apis mellifera*	20 colonies	90%	100%	Clermont et al. (2015)
2012	Belgium	Europe	*Apis mellifera*	1 colony	absent	present	Benaets et al. (2017)
2012–2013	USA (Hawaii)	Pacific	*Apis mellifera*	4 NGS libs.^a^	100%	100%	Mordecai et al. (2016)
2012–2013	Kenya	Africa	*Apis mellifera*	16 NGS libs.^a^	45%	55%	Onyango et al. (2016)
2014	Georgia	Asia	*Apis mellifera*	40 bees	>29%	<29%	Radzevičiūtė et al. (2017)
2014	Austria	Europe	*Apis mellifera*	4 colonies	0%	100%	Tritschler et al. (2017)
2014	Yemen	Asia	*Apis mellifera*	16 sites	38%	6%	Haddad et al. (2018)
2014	New Zealand	Pacific	*Apis mellifera*	8 NGS libs.^a^	present	present	Mondet et al. (2015)
2015	Philippines	Asia	*Apis mellifera*	2 colonies	100%	100%	de Guzman et al. (2020)
2015	Spain	Europe	*Apis mellifera*	10 colonies	90%	90%	Barroso-Arévalo et al., 2019
2015	Brazil	S. America	*Apis mellifera*	27 colonies	100%	11%	de Souza et al. (2019)
2015	Chile	S. America	*Apis mellifera*	612 colonies	71%	3%	Riveros et al. (2020)
2015	China	Asia	*Apis mellifera*	117 colonies	46%	2%	Diao et al. (2019)
2016	UK	Europe	*Eristalis arbustorum*	20 *E. a.* flies	0%	5%	Bailes et al. (2018)
2016	Argentina	S. America	*Apis mellifera*	45 apiaries	91%	47%	Brasesco et al. (2021)
2017	Ethiopia	Africa	*Apis mellifera*	20 colonies	0%	100%	Gebremedhn et al. (2020)
2017–2018	Egypt	Africa	*Apis mellifera*	14 sites	present	present	Abd-El-Samie et al. (2021)
2019	Czech Republic	Europe	*Apis mellifera*	250 colonies	58%	26%	Mráz et al. (2021)
2020	Italy	Europe	*Apis mellifera*	171 colonies	51%	18%	Bordin et al. (2022)

Thaduri et al. (2018) and post 2010 records for *Apis mellifera* from Europe have not been added to Fig. 2.

First records in *Bombus* spp. and the hover fly *Eristalis arbustorum* have also not been added to Fig. 2.

a NGS libs., next generation libraries.

In these European countries in geographic proximity to the Netherlands, DWV-B was detected at moderate to high prevalence (Table 3), when given, suggesting that it was already well established and not a recent introduction. Because Europe held many early DWV-B records, we did not plot in Fig. 2 those adjacent European countries with much later records. We also did not plot Sweden (samples collected in 2009–2010; see Thaduri et al., 2018) because NGS analysis suggested the presence of ‘few’ DWV-B reads, which did not permit even partial genome assembly.

Records from east and southern Africa paint a similar picture of rapid spread or long-standing presence (Fig. 2, Table 3). Among these localities, DWV-B's first detection was in the only sample collected in 2004 from South Africa (de Souza et al., 2021). When first detected, DWV-B was at high prevalence (Table 3), supporting the idea that it has had a long-standing presence in the region in *A. mellifera*. Records from North Africa suggest a slightly more recent introduction of DWV-B, in which it was apparently not widespread but nevertheless detected at multiple sites, suggesting a rather recent introduction (Fig. 2, Table 3).

The picture in the New World suggests a very recent introduction of DWV-B (Fig. 2, Table 3). The earliest records of DWV-B are from pooled colony samples of *A. mellifera* collected in 2010 in the USA (Ryabov et al., 2017), and it has since been detected in Brazil, Chile and Argentina (Fig. 2). In three of these four cases, DWV-B was found at low prevalence, in one or a few colonies, whereas DWV-A was at high prevalence, supporting the notion that DWV-B was a recent introduction to those localities at the date of sampling (Table 3). In one of these four cases, that from Argentina, DWV-B was at considerable prevalence (46%) in apiaries around the Buenos Aires region, though DWV-A was at high prevalence throughout the country (91%; Table 3).

In western Asia, where *A. mellifera* is native, first recording of DWV-B is typically represented by multiple cases per country, suggesting that the viral genotype had previously been under-recorded (Fig. 2, Table 3).

The New World pattern is repeated in New Zealand and eastern Asia, where *A. mellifera* is not native. The first record of DWV-B from New Zealand is 2014 and that from China is 2015 (Fig. 2, Table 3). Diao et al.’s (2019) data show that DWV-A was widespread and at substantial prevalence (46%) in *A. mellifera* colonies in China in 2015 whereas DWV-B was rare (<1% of colonies). A second study of Chinese honey bees, also sampled in 2015, found essentially the same pattern: DWV-A was widespread and prevalent in *A. mellifera* and *Apis cerana* at the apiary level whereas DWV-B was at lower prevalence in *A. mellifera* and was not detect in *Apis cerana* (Yuan et al., 2021). These data lend weight to the idea that DWV-B is a recent introduction to the honey bees of south and east Asia.

DWV-B's first record in non-*Apis* bees is from a study of British bumble bees (*Bombus* spp.) collected in 2011 (Fürst et al., 2014) and from British hover flies (Diptera, Syrphidae) collected in 2016 (Bailes et al., 2018) (Table 3). Under-recording of other insect species likely leads to a delayed date of first recording DWV-B in them.

### 3.3 DWV-B has risen in predominance within populations

**Fig. 3:**
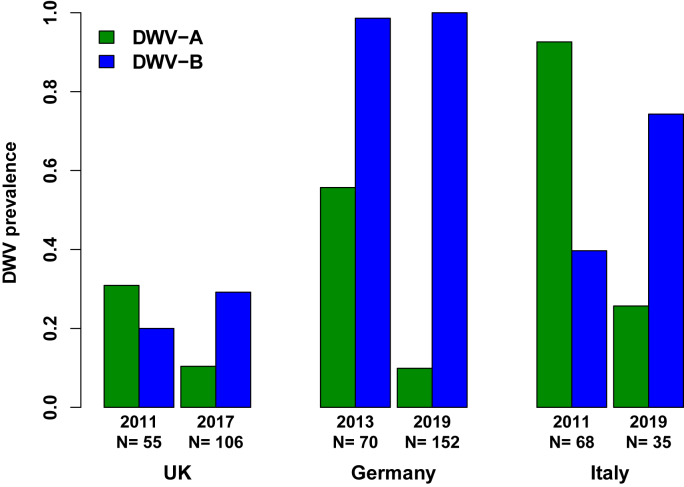
Temporal change in the prevalence of DWV-A and DWV-B in honey bees in three original datasets separated by 5–6 years from the same sampling localities in the UK (individual honey bees collected at flowers), Germany (pooled honey bees from collapsing colonies) and Italy (NGS reads from pooled or individual honey bees); Germany 2019 samples were summed 2019–2020; Italy 2011 samples were summed 2009–2013 and Italy 2019 samples were summed 2018–2020.

Our novel data on the temporal change over a five-to-six year timespan in the prevalence of DWV in honey bees from mainland UK, Germany and northeast Italy reveal a consistent pattern that supports the notion that DWV-B is replacing DWV-A (Fig. 3). In the UK, the change in predominance of DWV-B over DWV-A seems to have happened between 2011 and 2017. In Germany, DWV-B was already the predominant genotype in 2013, being found in almost all colonies (>98%) and remained dominant in 2019/20 (found in 100% of colonies) whilst DWV-A fell from a prevalence of 56%–10% of colonies in the same time period. In Italy, there has been a change from DWV-A being present in most NGS libraries from 2009/13 (DWV-A: 93% of libraries; DWV-B: 40% of libraries) to DWV-B in 2018/20 (DWV-A: 26% of libraries; DWV-B: 74% of libraries). We note that some 2018/20 NGS libraries contained a recombinant DWV-A/B virus (Supplementary Fig. S2).

For the UK, the absolute prevalence of DWV was low, likely reflecting the source of samples, namely honey bees randomly collected at flowers whilst foraging, and the analytical approach used to detect virus (less sensitive, non-TaqMan based qPCR). In German, DWV-B was dominant at both time-frames and found in almost all colonies. The pooling of honey bees from collapsing colonies (i.e. those most likely to be infected by pathogens) in the German dataset coupled to very sensitive TaqMan based qPCR assays might account for DWV-B's apparent ubiquity in Germany. For the Italian datasets derived from randomly selected honey bees, very sensitive NGS-based analysis represents the highest resolution of DWV-genotype prevalence. Though the source of collected honey bees and methods of detection of DWV differed among countries, the methods of data collection for each country were internally consistent across a country's two periods of sampling, allowing conclusions to be drawn over trends in the prevalence of viral genotypes.

**Fig. 4:**
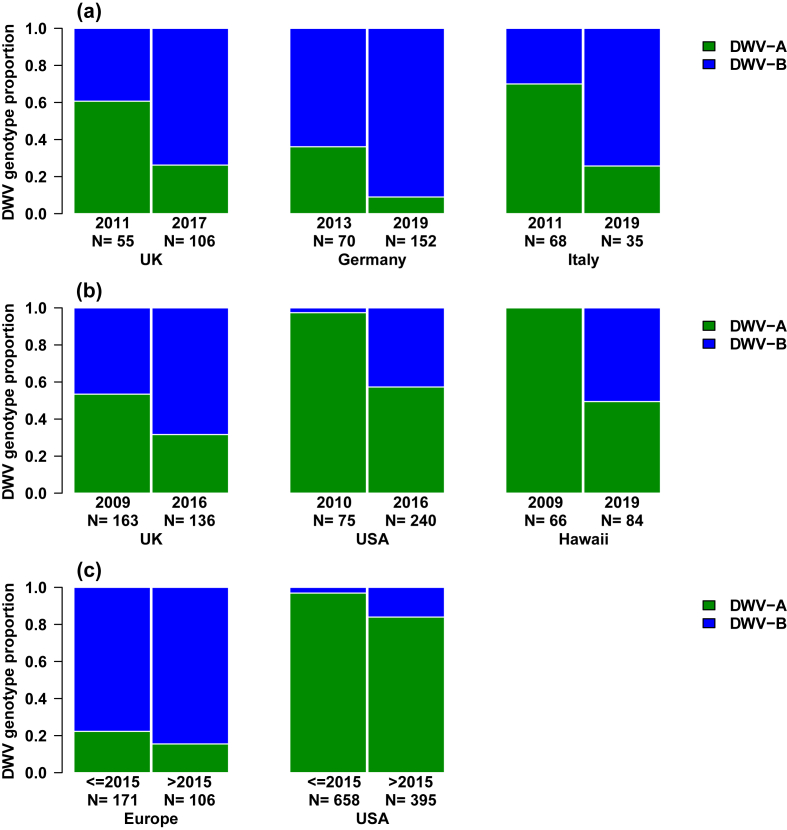
Temporal change in the proportion of DWV-A to DWV-B across our own datasets (a) (prevalence in Fig. 3); in published datasets (b) (UK data in Kevill et al. (2021); continental USA data in Ryabov et al. (2017); and Hawaii data in Grindrod et al. (2021)); and (c) in NCBI NGS honey bee datasets of Fig. 1 presented by geographic origin.

To synthesise our novel data and facilitate comparison across published datasets, we present the relative proportion of DWV-A and DWV-B records (individual honey bees, colony samples, NGS libraries) in Fig. 4. As described above, our novel datasets suggest that DWV-B has risen in prevalence in three European countries (Fig. 4a). Published datasets paint a similar picture (Fig. 4b). Kevill et al.’s (2021) UK dataset is very similar to our own data across a similar time-frame, supporting an ongoing rise in DWV-B's prevalence. On continental USA (Ryabov et al., 2017) and on Hawaii (Grindrod et al., 2021), change-over from DWV-A to DWV-B seems to be ongoing but delayed relative to genotype dynamics in European countries. We tentatively suggest that DWV-B may even be replacing DWV-A.

The profound difference in the dynamics of DWV-B replacement across continents is made stark by a comparison of the NGS-reads in NCBI's public database. Plotting pre- and post-2015 datasets (Supplementary Table S1) in relation to region of origin (USA versus Europe) reveals that DWV-B reads predominate in European NGS libraries whereas they make up a small albeit growing number of reads in US accessions (Fig. 4c).

### 3.4 Epidemiological model predicts rise in DWV-B and replacement of DWV-A

We initiated simulations of our model by assuming that DWV-B enters a population of honey bee colonies as a rare variant (B = 0.01) in which DWV-A is already at moderate prevalence (A = 0.3). Furthermore, we initially assumed colony-to-colony genotype-specific intrinsic transmission rates:

μ_A_ = 0.1, μ_B_ = 0.15 (assuming DWV-B is 50% more transmissible than DWV-A, based on its higher rate of replication, an assumption we changed in simulations), and fatality rates:

ν_A_ = ν_B_ = ν_M_ = 0.03 (assuming all infected colonies regardless of state (A, B or M) have the same fatality rate, an assumption we relaxed in simulations).

**Fig. 5:**
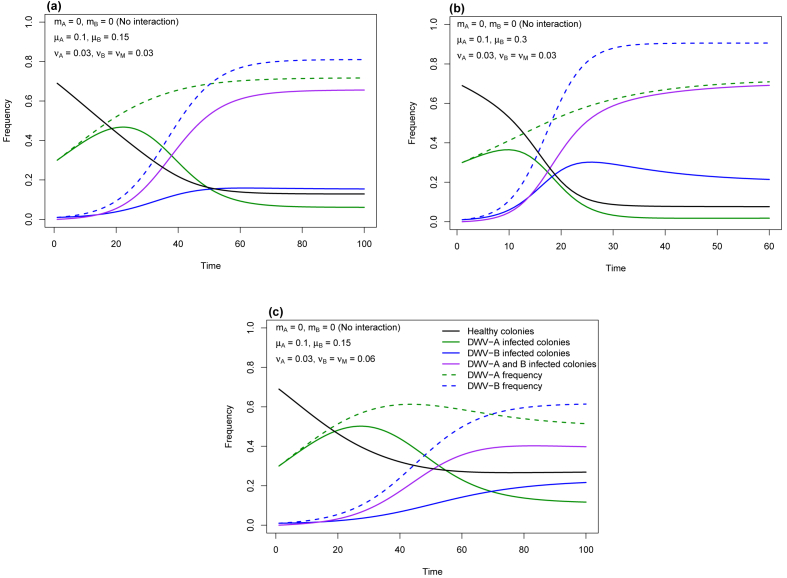
The dynamics of DWV-A and DWV-B from an epidemiological model in which viral genotypes do not interact when co-infecting a host honey bee colony. Parameter values for the genotype-specific intrinsic rate of transmissionμ, fatality (virulence) ν, and interaction (m = 0) are given in the top left of each plot and (c) additionally provides the key to coding of lines. (a) transmission rate of DWV-B > DWV-A, virulence of all colony states is equal; (b) transmission rate of DWV-B is doubled, virulence of all colony states is equal; (c) transmission rate of DWV-B > DWV-A, virulence of colony states B and M is doubled. The unit of Time (abscissa) is in model iterations; one Time unit may represent three or more months to a year.

Assuming no interaction between DWV-A and DWV-B when co-infecting the same colony (m_A_ = m_B_ = 0), our epidemiological model predicts that DWV-B will rise rapidly and that both DWV-B and DWV-A will eventually reach high frequency (Fig. 5a). This means that most colonies become infected with both DWV-A and DWV-B (status M). Doubling the intrinsic rate of transmission of DWV-B (μ_B_ = 0.30) increases its frequency (Fig. 5b) whereas doubling the fatality rate of DWV-B to 0.06 (ν_B_ = ν_M_ = 0.06) reduces both its and DWV-A's frequency (Fig. 5c). DWV-A is not, though, replaced by DWV-B.

Mathematically, the magnitude of change in μ or ν of a viral genotype has an equal (though opposite) effect on its equilibrium frequency. This is because it is directly dependent on μ/ν in this simple model in which viral genotypes do not interact.

**Fig. 6:**
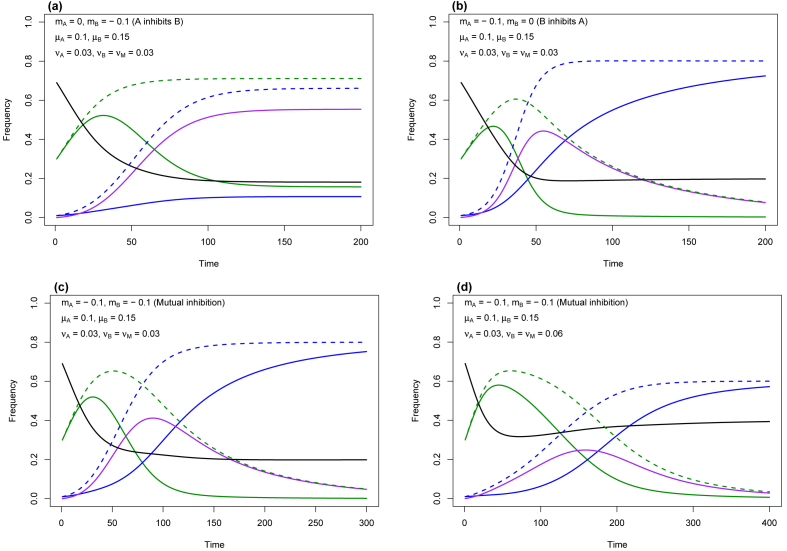
The dynamics of DWV-A and DWV-B from an epidemiological model in which viral genotypes interact antagonistically when co-infecting a host honey bee colony. Parameter values for the genotype-specific intrinsic rate of transmissionμ, virulence ν, and interaction (m) are given in the top left of each plot. See Fig. 5c for the key to coding of lines. (a) transmission rate of DWV-B > DWV-A, virulence of all colony states is equal, DWV-A inhibits DWV-B; (b) transmission rate of DWV-B > DWV-A, virulence of all colony states is equal, DWV-B inhibits DWV-A; (c) transmission rate of DWV-B > DWV-A, virulence of all colony states is equal, DWV-A and DWV-B mutually inhibit each other; (d) transmission rate of DWV-B > DWV-A, virulence of colony states B and M is doubled, DWV-A and DWV-B mutually inhibit each other. The unit of Time (abscissa) is in model iterations; one Time unit may represent three or more months to a year.

Introducing into the model an interaction term between DWV-A and DWV-B when co-infecting the same colony (m_A_ ≠ m_B_ ≠0), our epidemiological model predicts that either DWV-A or DWV-B will dominate, partly in accordance with the bias in interaction strength and direction. If DWV-A inhibits DWV-B but not *vice versa* (m_A_ = 0, m_B_ = −0.1), DWV-A dominates (Fig. 6a); if DWV-B inhibits DWV-A but not *vice versa* (m_A_ = −0.1, m_B_ = 0), DWV-B dominates (Fig. 6b).

Assuming mutual inhibition (each viral genotype inhibits the transmission of the other i.e. m_A_ = m_B_ = −0.1) leads to dominance of DWV-B and loss of DWV-A (Fig. 6c) as well as the loss of colonies in status A and M and their replacement by colonies of status B. The rise in the prevalence of DWV-B and its replacement of DWV-A is also found under mutual inhibition, even when the fatality rate induced by DWV-B is doubled (ν_B_ = ν_M_ = 0.06, Fig. 6d). Regardless of the initial frequency of A, replacement is deterministic and, under these parameter values, DWV-B replaces DWV-A (equilibrium values of colony states are B = 0.8, H = 0.2, A = 0; simulations not shown). As the fatality rate of colonies infected by DWV-B (ν_B_ and ν_M_) rises, then the equilibrium frequency of DWV-B drops until a threshold (where ν_B_ = ν_M_ = 0.083), at which point DWV-B is lost from the population and DWV-A rises from zero to high prevalence (Supplementary Fig. S3).

## 4 Discussion

We present multiple, independent datasets, including publicly available NGS transcriptome (RNAseq) datasets, literature-based records of regional first occurrences, own (novel) and published prevalence datasets and theoretical modelling that collectively suggest the recent (in the last 5–20 years) arrival of DWV-B and its potential replacement of DWV-A as a major pathogen of honey bees across most of the world, excepting Australia. We now critically evaluate the data, highlight knowledge gaps, and then go on to address both the causes and consequences of the switch in DWV genotype from A to B that, in many countries, is likely ongoing.

### 4.1 DWV-A has dominated publicly accessible NGS transcriptome datasets

Publicly available honey bee RNAseq datasets have been previously interrogated for DWV reads (Cornman et al., 2013; Cornman, 2017; Gerth and Hurst, 2017), revealing that libraries frequently comprise a substantive proportion of DWV reads and suggesting that DWV is indeed a widespread and often unseen pathogen of *A. mellifera* across most of its native and introduced range. That we also frequently detected DWV in publicly accessible NGS datasets after excluded NGS libraries derived from infection experiments underscores the ubiquity of DWV in ostensibly healthy honey bees.

Cornman (2017) and Gerth and Hurst (2017) both found a preponderance of DWV-A reads over DWV-B reads in their analyses of honey bee NGS datasets, as did we. These independent analyses of overlapping datasets suggest that the lack of DWV-B, particularly before 2016, is probably not due to under-recording or bioinformatic error but rather that DWV-B is, or was, at very low prevalence at the time it was first detected. Why it is, or was, rare is not clear, though two plausible hypotheses are that it originally occurred in a formerly isolated population of *A. mellifera* or that it has relatively recently switched host (e.g. from another insect species or from *V. destructor*) to *A. mellifera* (Wilfert et al., 2016).

Though our analysis of NGS datasets suggests a slight upward trend in the occurrence of DWV-B over DWV-A in more recent years (2016–2020), the overall picture is that DWV-A remains dominant, which is somewhat at odds with the data on DWV genotype prevalence from screening colonies by qPCR. The low abundance of DWV-B reads may reflect a time-lag between viral prevalence in a population of honey bees and the appearance of viral reads in NGS datasets deposited in public repositories or the insensitivity of NGS datasets to reflect the prevalence of viral genotypes in a population of honey bees. Based on the data on viral prevalence in individual honey bees or colonies presented here (section 3.3), we predict that future NGS transcriptome libraries of ostensibly healthy honey bees will contain an increasing proportion of DWV-B over DWV-A reads.

### 4.2 Rapid geographic spread of DWV-B

From its first discovery in *V. destructor* collected in 2001 (Ongus et al., 2004), DWV-B has been remarkably successful in dispersing to all major land masses excepting Australia, and in its association not only with *A. mellifera* and *V. destructor* but also with a range of other insect flower-visitor species (Fürst et al., 2014; Bailes et al., 2018; Brettell et al., 2019, 2020). Taking into consideration its prevalence at first detection in a country or region, our assessment of the available data suggests that it has been present in sub-Saharan Africa, western Asia and Europe for the past two decades, if not longer. Interestingly, all pre-2010 records for DWV-B are in the native range of *A. mellifera*, and all are associated with DWV-B at not insubstantial prevalence. These data suggest that DWV-B may be a long-standing pathogen of *A. mellifera*, perhaps in a formerly isolated honey bee population. In stark contrast, in the New World, south and eastern Asia and New Zealand, DWV-B's first detection has generally been as an isolated case, suggesting it is a genuinely recent arrival in those regions, possibly having dispersed to them only in the past 10 years or more recently.

It is difficult to know exactly when DWV-B first dispersed to a country or geographic region because of the potential biases that might have led to its under-recording. Its first discovery in Dutch *V. destructor* in 2001 (Ongus et al., 2004) undoubtedly post-dates its arrival in the Netherlands. It also does not necessarily signify that DWV-B was a former parasite of *V. destructor* that switched host to *A. mellifera* because the genetically similar DWV-A has always been associated with *A. mellifera* (Bailey and Ball, 1991), even before the jump of *V. destructor* to *A. mellifera* over 70 years ago in south and east Asia (Traynor et al., 2020). Furthermore, in the source populations of *V. destructor* in south and east Asia, DWV-B is a relatively recent introduction, further supporting the idea that DWV-B has not been a long-term associate of *V. destructor*. It is more likely that DWV-B already resided in *A. mellifera*'s native range of Africa, west Asia and Europe, possibly in *A. mellifera* itself.

In exactly which population(s) of *A. mellifera* DWV-B might have originally resided is not clear. A molecular clock estimate suggests that DWV-B had a last common ancestor with DWV-A ca. 150 years ago (Mordecai et al., 2016). A phylogeny of DWV-B sequences obtained from bees collected in 2015 from islands off the British and French coasts demonstrate that DWV-B is currently undergoing a major demographic expansion (Manley et al., 2019), suggesting that the putative origin of DWV-B lies elsewhere.

### 4.3 Replacement of DWV-A by DWV-B

If uncertainty remains over precisely when DWV-B was first present in a region's honey bees, temporal sampling consistently demonstrates a rise in the prevalence of DWV-B once it enters a region, seemingly at the expense of DWV-A. This pattern has been previously demonstrated in the USA and UK (Ryabov et al., 2017; Grindrod et al., 2021; Kevill et al., 2021), and we now confirm the pattern in the UK with an independent dataset and expand geographic scope by showing DWV-B's rise in prevalence in two additional European countries, Germany and Italy.

The much later detection of DWV-B in the USA (first detection in 2010) compared to Europe (first detection in 2001) may account for why DWV-B's rise in prevalence and its potential displacement of DWV-A is delayed in the USA in comparison to Europe. Based on the available European data, we predict that DWV-B will also increase in prevalence in the following one to two decades in the USA. Since the dramatic overwinter losses of US honey bee colonies in 2006/07 (Oldroyd, 2007), the heightened intensity of pathogen monitoring of *A. mellifera* colonies (e.g. Traynor et al., 2016) will facilitate future description of the pattern of genotype replacement in America.

DWV-C has been a rarely reported variant in *A. mellifera* populations since its first discovery in UK honey bees (Mordecai et al., 2016). We found it at very low prevalence in NGS reads of Italian honey bees from pre-2013 but it was absent from more recent (2018–2020) Italian datasets, concurrent with the rise of DWV-B in those same datasets. Kevill et al. (2017) also found it in pre-2010 UK honey bee samples, but not in more recent UK datasets, also concurrent with the rise of DWV-B. It may be that DWV-A (and possibly DWV-B) has replaced DWV-C in the same manner in which DWV-B is now potentially replacing DWV-A. It is also possible that other bee species are reservoir hosts of DWV-C (e.g. de Souza et al. (2019) found it to be at high prevalence in Brazilian *Melipona nitida* stingless bee populations), that DWV-C occasionally spills over to honey bees and that DWV-B halts DWV-C's growth in honey bees with greater efficacy than DWV-A.

Interestingly, the new variant DWV-D has recently been sequenced from exhumed Egyptian honey bees collected in the 1970s and subsequently stored at Rothamsted Research, UK (de Miranda et al., 2022). DWV-D is not found in public sequence repositories such as NCBI's SRA, suggesting that DWV-D has disappeared from honey bee populations. A plausible hypothesis is that DWV-D has been replaced by DWV-A. Replacement of one genotype (or variant) by another with higher fitness might be an ongoing process in the evolution of DWV, as it is in SARS-CoV-2 in human populations (Li et al., 2021), including the current replacement of variant Omicron BA.1 by variant Omicron BA.2 (Callaway, 2022).

### 4.4 Epidemiological modelling of DWV-A and DWV-B

Interactions among pathogens when coinfecting hosts are considered to play an important role in shaping their epidemiology (Read and Taylor, 2001); examples include vector-borne rodent malaria (Pollitt et al., 2013), *Nosema* in *A. mellifera* (Natsopoulou et al., 2015), and here DWV, also in honey bees. In this regard, epidemiological models can be useful in predicting and interpreting the dynamics of two co-occurring pathogens by, for example, pinpointing critical biological aspects that provide a mechanistic explanation for their observed dynamics. An example, Natsopoulou et al.’s (2015) epidemiological model of the resident honey bee Microsporidian pathogen *Nosema apis* and the invasive EID *Nosema ceranae* has demonstrated the importance of the competitive superiority of *N. ceranae* yet its cold intolerance in explaining its replacement of *N. apis* in colonies of honey bees in warm climatic zones.

Inspired by the model of Natsopoulou et al. (2015), our epidemiological model describing the dynamics of two co-occurring genotypes of DWV can capture the increase in frequency of DWV-B in a population (and the increase in frequency of colonies infected by DWV-B). Importantly, our model highlights how DWV-B's replacement of DWV-A is only possible when two genotypes interact negatively.

But are our model parameter values describing colony-level virulence and transmission plausible, if not in absolute then in relative terms? We first consider our model parameter: virulence. In the laboratory, DWV-B is more virulent in adult honey bees than DWV-A, causing a reduction in median lifespan of 53% and 38% respectively (McMahon et al., 2016). Plugging these virulence estimates into the BEEHAVE model (Becher et al., 2014) that simulates the dynamics of a honey bee colony results in colony death after 3 years and 4 years for DWV-B and DWV-A, respectively (McMahon et al., 2016). To date, the field lacks experimental estimation of colony-level virulence of DWV-A or DWV-B. The nearest is a study by Norton et al. (2021), in which the number of *V. destructor* mites per colony was experimentally manipulated in colonies infected by both DWV-A and DWV-B; colony mortality, though not a goal of the experiment, was recorded in colonies infected primarily by DWV-B. There has been a counter-suggestion in the literature that DWV-B is benign and therefore less virulent that DWV-A (Mordecai et al., 2015). We review the evidence that largely rejects this claim in section 4.5 but note that, if it were benign, then DWV-B would likely go rapidly to fixation (all colonies infected with DWV-B). Given discrepancy in the literature, we therefore set our parameter ν (colony-level virulence) of DWV-A at 0.03 and of DWV-B at 0.03 or 0.06.

We next consider our model parameter: transmission. Several experimental studies in which honey bee adults and pupae have been inoculated with DWV-A or DWV-B show that DWV-B replicates faster that DWV-A (e.g. McMahon et al., 2016; Tehel et al., 2019; Norton et al., 2020), at least during its first experimental passage through a host pupa (Ray et al., 2021). High titres are generally – though not always – associated with greater pathogen transmission (Schmid-Hempel, 2011), which provides the basis for our initial estimate of μ (the colony-level rate of transmission) to lie at 0.1 and 0.15 for DWV-A and DWV-B, respectively. Gusachenko et al. (2020) and Gisder and Genersch (2021) have recently shown that DWV-B can replicate in *V. destructor* mites. This would potentially provide an additional mechanism that elevates the rate of transmission of DWV-B over that of DWV-A, assuming that DWV-B parasitism does not shorten the lifespan of a mite. It has long been speculated that a viral genetic variant may adapt to *V. destructor*-mediated transmission (Neumann et al., 2012). DWV-B, now rising to prominence across the world (excepting Australia), may be the current DWV variant favoured through *V. destructor* vectoring.

Our most plausible model of the take-over of DWV-B, which incorporates viral genotype-genotype inhibition, predicts a rise in DWV-B to high prevalence (Fig. 6a) and its complete elimination of DWV-A (Fig. 6b–d) after 50–250 model iterations. Each iteration represents colony birth, death and conversion from one infection state to another, processes that happen continuously throughout the annual honey bee colony cycle but which, for mathematical ease, we defined in model iterations or discrete intervals of unit ‘Time’ (the abscissa unit of Fig. 6). Honey bee colonies in temperate regions may swarm annually (e.g. Seeley, 1985, pp. 43–46), suggesting that, as an upper estimate, our ‘Time’ unit equates with one year. Colonies may, though, convert from a healthy to an infected state (A or B or M) far more frequently through the acquisition of *V. destructor*-parasitised (and DWV-infected) drifting or robbing worker honey bees (Greatti et al., 1992; Frey and Rosenkranz, 2014). They also swarm between two and four times per year in subtropical and tropical climates (Seeley, 1985, pp. 148–149). This suggests that our ‘Time’ axis may equate with months and that take-over by DWV-B within a population of honey bees may happen far faster than 50–250 years e.g. 12–60 years when swarming four times per year. Understanding the dynamics of viral acquisition by a colony would help to place greater confidence on the timing of our model dynamics as well as contribute to better-informed guidance on beekeeping disease management.

### 4.5 Could recombinational meltdown explain the replacement of DWV-A by DWV-B?

The empirical data we have presented leave little room for doubt that DWV-B is spreading, both across the world and, within a population of honey bees, among colonies i.e. it increases in prevalence. Our model captures this ongoing phenomenon. Two crucial questions are (i) whether DWV-B is replacing DWV-A (i.e. eliminating DWV-A) and, if so, (ii) by what mechanism?

Our German prevalence data may provide an answer to the first question: is DWV-B replacing DWV-A. Given DWV-B's first description in Dutch *V. destructor* (Ongus et al., 2004) and therefore assuming an origin of DWV-B, or its early arrival, on continental Europe, honey bees in countries adjacent to the Netherlands, such as Germany, are likely to have been amongst the first to have acquired DWV-B. Amongst our three original datasets, the dynamics of co-occurring DWV-A and DWV-B are likely to have proceeded the furthest in Germany, which has a long land border with the Netherlands. Indeed, our data suggest the greatest domination of DWV-B and loss of DWV-A in German colonies, supporting the idea that DWV-B not only rises in prevalence but also replaces DWV-A. If so, replacement likely takes many years, as captured by our epidemiological model, because DWV-B prevalence in the first German sample in 2013 was already very high (0.99, versus 1.00 in 2019/20) over a timeframe when DWV-A dropped in prevalence from 0.56 to 0.10. Alternatively, DWV-B may suppress DWV-A to below a threshold level of detection. A more thorough analysis that includes absolute quantification of viral titre and not merely scoring viral prevalence as a binary (present/absent) might give greater insight into the dynamics of the two genotypes.

A caveat of our German data is that colony samples were derived from collapsing colonies, potentially elevating viral prevalence, particularly that of the more virulent DWV-B. Ideally, random sampling of a large number of colonies is needed to address this caveat. We note, however, that Traynor et al. (2016) found DWV (DWV genotype undetermined) to be at high (up to 100%) prevalence in a large collection of randomly sampled US honey bee colonies, demonstrating that DWV can reach very high prevalence. Furthermore, Natsopoulou et al. (2017), in a random sample of 28 colonies collected in SW Germany in 2011, found 27 of 28 to be infected with DWV-B and none to be infected with DWV-A, suggestive of replacement.

The second question relates to the mechanism of interaction that permits replacement of DWV-A by DWV-B. Our model incorporates antagonism between DWV-A and DWV-B when coinfecting a colony (m_A_ and m_B_) but does not specify the mechanism. Several can be envisaged. Firstly, two viruses coinfecting the same host may compete for limiting host resources (e.g. viruses coinfecting the human respiratory tract; Pinky and Dobrovolny, 2016). Secondly, immune escape, e.g. through antigenic drift, may allow one viral variant to spread through a host population and replace a former variant (Telenti et al., 2021), as in the case of Covid-19, in which Delta has replaced Alpha and Omicron seems to be replacing Delta in human populations (Mallapaty, 2022). Unlike humans, honey bees do not possess an acquired immune system, arguing against the role of immune escape. But they do possess an innate immune system (Evans et al., 2006), which has been hypothesised to permit novel variant escape (Ryabov et al., 2019). Such a mechanism might explain why DWV-B gains an advantage of DWV-A when initially entering a population dominated by the latter. But given that this mechanism is essentially a form of negative frequency-dependent selection in a host lacking an acquired immune system, it likely cannot explain the dominance of DWV-B because, when at high prevalence, DWV-B would self-inhibit. A third mechanism of negative interaction that might lead to DWV-B eliminating DWV-A from a population of honey bees is recombination.

Viral recombination, e.g. through template switching during replication, can occur at a high rate in RNA viruses and be of major evolutionary significance (Simon-Loriere and Holmes, 2011). It is a well-established mechanism of evolution in members of the *Picornaviridae* (e.g. mammalian enteroviruses, Oberste et al., 2004). It requires an individual host cell to be co-infected by two or more viral variants. Experimental co-infection of honey bees by McMahon et al. (2016) led to the generation of 0.15% DWV-A/B recombinants, though the method of their detection in an NGS dataset through mis-matched PE reads likely underestimated the recombination rate. Whether co-infection of individual cells occurs frequently enough in a co-infected host to cause recombination meltdown remains an open question, though the frequent occurrent of DWV-A/DWV-B co-infected honey bees (McMahon et al., 2016) suggests it is plausible.

Recombinants have indeed been frequently detected between DWV-A and DWV-B in honey bee populations (e.g. Moore et al., 2011), especially those harbouring both genotypes e.g. Israel (Zioni et al., 2011; Daughenbaugh et al., 2021), the UK (Wang et al., 2013), France (Dalmon et al., 2017), Tunisia (Abdi et al., 2018), Hawaii (Brettell et al., 2019, 2020), Spain (Barroso-Arévalo et al., 2019), and Egypt (Abd-El-Samie et al. et al., 2021). We also detected a DWV-A/B recombinant in our recent (2018–2020) Italian dataset. Though a DWV-A/B recombinant with higher virulence than DWV-A was found to predominate in British honey bees in the Warwick-HRI apiary (Moore et al., 2011; Ryabov et al., 2014), recombinants have been found to be surprisingly infrequent in more recent analyses of the continental US honey bee population (Ryabov et al., 2017). Furthermore, experimental co-infection of honey bees with DWV-A and a putative DWV-A/B recombinant and passaging through honey bee pupae did not appear to lead to novel recombinants, though the original A/B recombinant was stable over repeated passages (Ray et al., 2021). Finally, Gusachenko et al. (2021) found that recombinant DWV did not out-replicate parental variants in laboratory assays (reviewed in Woodford and Evans, 2021).

These data argue for frequent recombination between DWV-A and DWV-B as DWV-B enters a honey bee population in which DWV-A is at high prevalence, though it leaves open the functional relevance of DWV-A/B recombinants. We hypothesise that the negative interaction between DWV-A and DWV-B is recombination, which leads to the generation of non-functional virus that is thereby eliminated from the host population i.e. a form of error catastrophe though recombinational meltdown. Under this scenario, co-infection by DWV-A and DWV-B could lead to the elimination of DWV-A from a population and the spread of DWV-B through its higher intrinsic rate of transmission.

Superinfection exclusion has been suggested to explain the replacement of DWV-A by DWV-B in three colonies of British honey bees (Mordecai et al., 2015), with the implication that there might exist a bias by which DWV-B blocks DWV-A. Gusachenko et al. (2021) have now demonstrated in a series of elegant inoculation experiments with virus generated by a reverse genetic system (Woodford and Evans, 2021) that competition between DWV-A and DWV-B is reciprocal, showing no directionality or dominance of one genotype over the other. We suggest that our epidemiological model of mutual inhibition (m_A_ = m_B_) represents the most parsimonious null model with which to explore explicitly the role of recombination in the evolutionary trajectory of DWV.

Viral recombination occurs when a host individual (a host cell) is co-infected, yet our model incorporates interaction between genotypes in co-infected colonies. Co-infection experiments to date have explored recombination in individual host honey bees (McMahon et al., 2016; Ray et al., 2021), which are an easier experimental unit of replication than colonies. Though it may seem a reasonable assumption to scale up the impact of viral recombination at the level of the individual to that at the level of the colony, it would be desirable to run colony-level experiments (as opposed to observations) to support this assumption. Furthermore, development of epidemiological models incorporating both within-colony as well as between-colony transmission (e.g. Betti and Shaw, 2021) will add greater realism and strengthen the predictive power of attempts to describe the ecological and evolutionary dynamics of DWV. Though the conditional transmission parameter of our model incorporated a frequency dependent element that can be considered to mirror within-colony interaction between two viral genotypes, explicit incorporation of these two hierarchical levels of biological organisation (individual, colony) e.g. through explicit incorporation of social networks, would strengthen epidemiological modelling (Sah et al., 2018) and prediction of viral evolutionary trajectory (e.g. Leventhal et al., 2015).

If recombinational meltdown might be the mechanism by which DWV-B replaces DWV-A, then this mechanism is unlikely to explain the evolutionary trajectory of viruses such as SARS-CoV-2. Recombinational meltdown requires frequent co-infection of the same host (the same host cell). DWV often exhibits extremely high prevalence, with up to 100% of colonies infected (Traynor et al., 2016). The prevalence of SARS-Cov-2 is thankfully much lower («10%) and therefore mutation and selection, rather than recombination, likely play a larger role in its evolutionary dynamics (e.g. Li et al., 2021).

### 4.6 The consequences of DWV-B replacing DWV-A

What impact DWV-B will have on honey bee populations, if and when it replaces DWV-A, is currently unclear. As we have pointed out above, experiments on individual honey bees suggest that DWV-B is more virulent than DWV-A, at least in adult hosts (McMahon et al., 2016), suggesting that it may lead to greater colony losses in the future. Observational studies that correlate colony mortality within a honey bee population with the occurrence of DWV-A versus DWV-B have been used to infer DWV-genotype virulence. For a USA dataset, colony mortality was associated with infection by DWV-A but not with DWV-B (Kevill et al., 2019); for one UK dataset, colony mortality was associated with infection by DWV-A but not DWV-B (Kevill et al., 2017), but for another UK dataset it was associated neither with DWV-A not DWV-B (Kevill et al., 2019); for a Czech dataset, colony mortality was associated with infection by DWV-B (and DWV-C) but not with DWV-A (Mráz et al., 2021). These conflicting reports likely emphasise the weaknesses of observational studies in explaining causation. Colony-level experiments are currently lacking, but would help resolve this open issue over the colony-level virulence of DWV-B. It is, though, prudent to suggest that both DWV-A and DWV-B cause honey bee colonies to collapse. Replacement of DWV-A by DWV-B is likely to cause greater loss of colonies, if not because of the greater virulence of DWV-B then because of its greater transmissibility, and possibly because of both. That beekeepers in Germany have been warned to pay greater attention to the impact of *V. destructor* on their colonies because of increased colony losses (Boecking and Genersch, 2008), coeval with the rise of DWV-B in that country, may be more than coincidence.

DWV has been widely detected not only in honey bees but also in a wide range of other bee species (Tehel et al., 2016), other insects, and other invertebrates (Nanetti et al., 2021), with the common assumption that DWV spills over to them from *A. mellifera*, its presumed reservoir host (Graystock et al., 2016a; Tehel et al., 2022). If DWV-B rises to high prevalence in *A. mellifera* populations across its distribution and, within a colony, to high titre, it is likely to become even more prevalent in other insects. The consequences of DWV-B infection on insects other that the honey bee are little explored. Commercial *B. terrestris* seems not to suffer reduced survival in benign laboratory conditions when inoculated with DWV-B (Tehel et al., 2020) but other experiments using DWV of undetermined genotype have suggested that it is detrimental to them (Fürst et al., 2014; Graystock et al., 2016b). There is a need to extend these experiments across host species and in a natural environment to be able to judge the impact of DWB-B on the wider insect community.

### 4.7 Conclusions

DWV-B has possibly originated in the native range of *A. mellifera* (Africa, west Asia and Europe), though revealing more precise details await detailed analysis of historical (preserved) specimens, if such still exist. It has more recently spread to the Americas and south and east Asia, and will likely soon be found in all countries hosting *A. mellifera* infested by *V. destructor*.

Where it has been present for many years, DWV-B has been found to rise markedly in prevalence, potentially replacing DWV-A. This pattern can be accounted for by its higher transmissibility despite potentially higher virulence. DWV-B (and DWV-A/B recombinants) may be better adapted for transmission by *V. destructor* than DWV-A. It will be important to determine whether replication in mites leads to increased transmissibility between colonies in the field.

Error catastrophe through recombination meltdown might be a mechanism by which DWV-B replaces DWV-A, though recombination might also give rise to novel variants of high virulence that warrant monitoring. Epidemiological modelling across a greater range of parameter values and in which recombination is explicitly incorporated will bring further insight into the dynamics of DWV-B, as will epidemiological models that can span from within-colony to between-colony dynamics. Empirical estimates of colony-level transmission and virulence are needed to improve modelling of DWV-B's dynamics as well as its impact, not only on beekeeping but also on the broader community of insects.

## Author contributions

Conceptualization: RJP; formal analysis: RJP, DB, VZ, FM, HS; mathematical modelling: HS; generation of original data: MOS, FN, VZ, DA, FM, ERLQ, DP, CJ; statistics: RJP, HS; writing - original draft: RJP. All authors reviewed and commented on the submitted manuscript.

## Funding

This research was funded by the German 10.13039/100005930Research Foundation (10.13039/501100001659DFG) (project: Pa632/10–1), the DFG's German Centre for Integrative Biodiversity Research (iDiv Flexpool projects W47011118 and W47021118), and the Centre for International Science and Technology Cooperation (CISTC), the Vice-presidency for Science and Technology of Iran. The funders played no role in the study design, the collection, analysis and interpretation of data, in writing or in the decision to submit the manuscript.

## Declaration of competing interest

None.
