# Supplementary material for: Epidemiology of a major honey bee pathogen, deformed wing virus: potential worldwide replacement of genotype A by genotype B
Source: Int J Parasitol Parasites Wildl. 2022 May 10;18:157–71. doi: 10.1016/j.ijppaw.2022.04.013 (PMC9112108; doi:10.1016/j.ijppaw.2022.04.013)
Supplement: Multimedia component 1 [file mmc1.pdf]

Supplementary Information to

Epidemiology of a major honey bee pathogen, deformed wing virus: potential worldwide replacement of genotype A by genotype B

Robert J. Paxton[a,b], Marc O. Schäfer[c], Francesco Nazzi[d], Virginia Zanni[d], Desiderato Annoscia[d], Fabio Marroni[d], Diane Bigot[a], Eoin R. Laws-Quinn[a], Delphine Panziera[a], Christina Jenkins[a] and Hassan Shafiey[a,b]

This file contains:

Supplementary Figures S1-S3

Supplementary Tables S1-S5

Supplementary Figures

Supplementary Figure S1. Location of seven sites (red dots) from which honey bees were sampled at flowers in 2017 on mainland UK; heatmap shows the prevalence of DWV-B in honey bees sampled at 26 sites in 2011, which included the seven sites resampled in 2017 (data from McMahon et al., 2016).

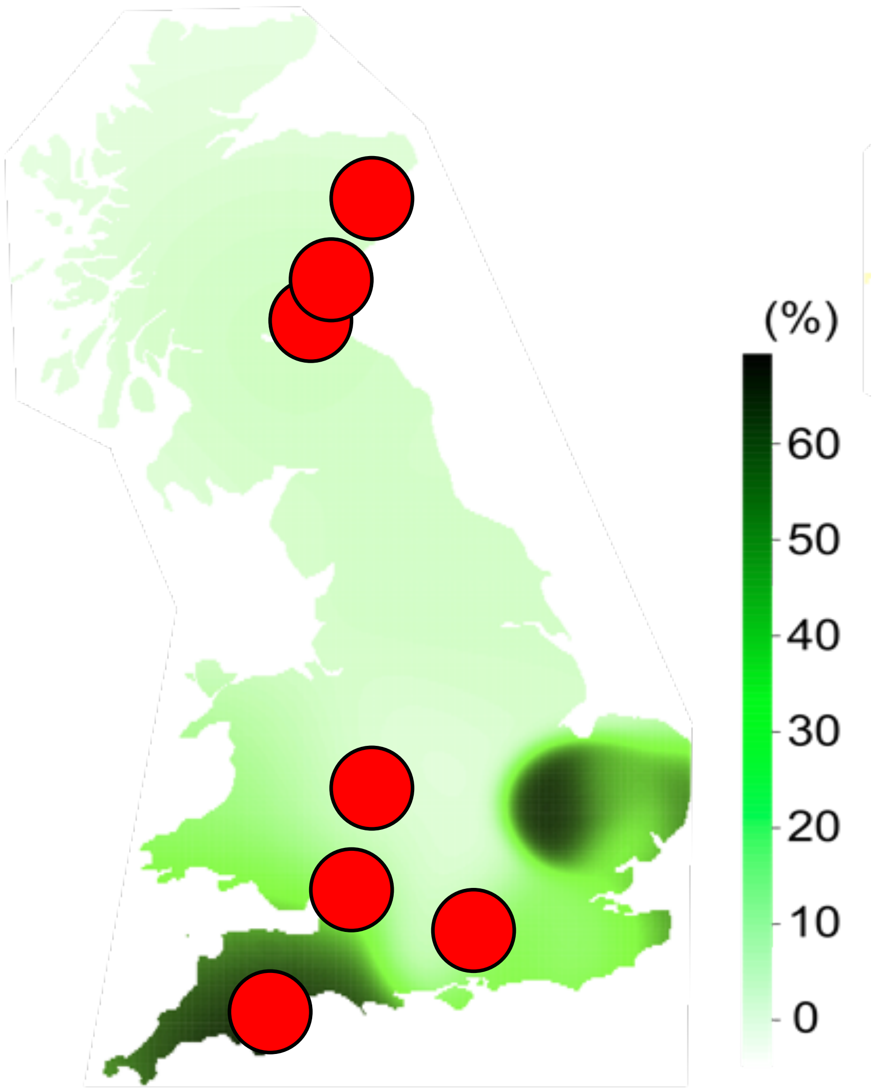

Supplementary Figure S2. Pile-up of NGS transcriptome reads of DWV from Italian honey bees collected in 2018 (see Supplementary Table S5). Three of the seven varroa-uninfested honey bees sampled in 2018 exhibited a recombinant genome with a DWV-A/DWV-B recombination point at the VP2 (capsid protein) encoding region (ca. 1800-1900). The same recombination pattern was observed in four varroa-uninfested and three mite infested honey bees collected in 2020. DWV isolated from honey bees collected from 2009 to 2013 did not show any recombinants between DWV-A and DWV-B.

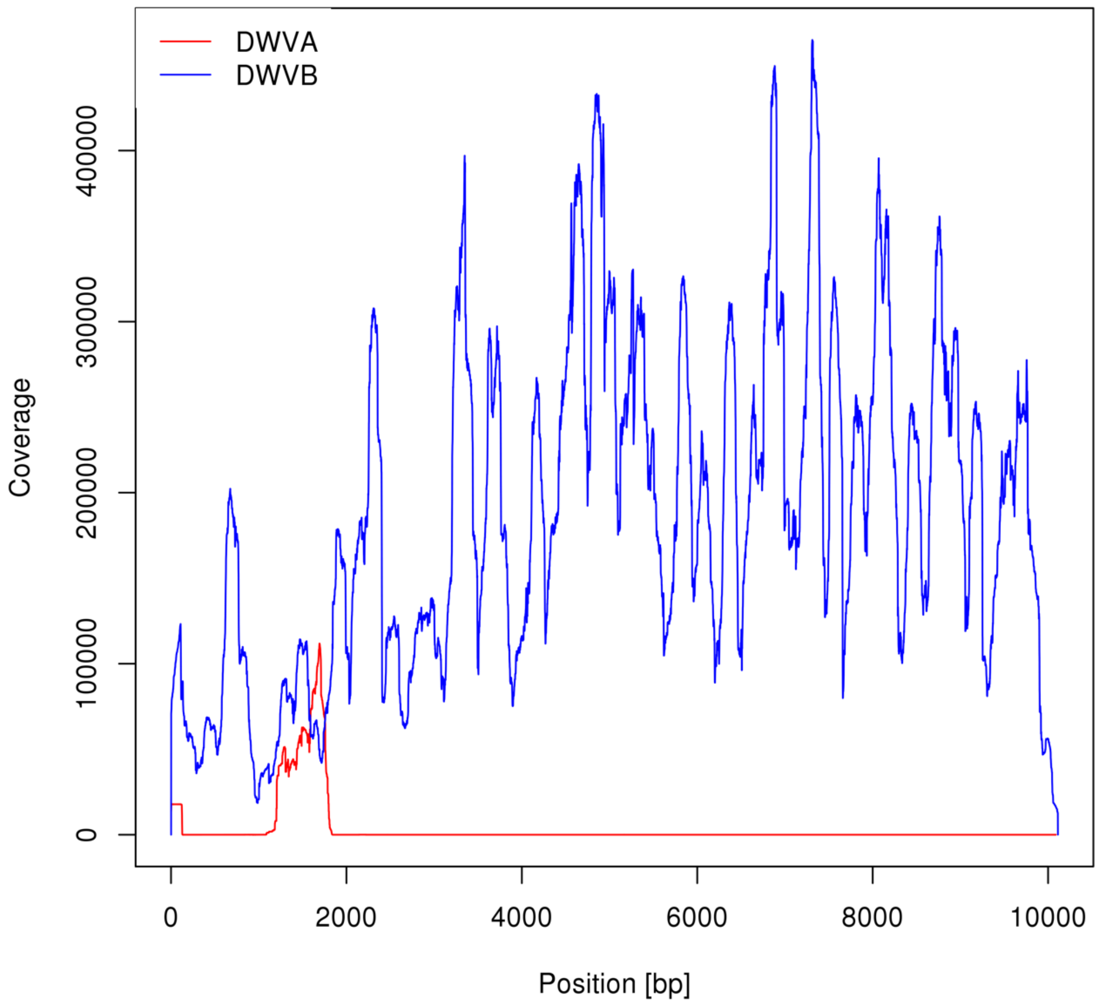

Supplementary Figure S3. Equilibrium frequencies of DWV-A (green dashed line) and DWV-B (blue dashed line) from our epidemiological model for a varying rate of fatality of colonies of status B and M ($\nu_B = \nu_M$) when each genotype is under mutual inhibition ($m_A = m_B = -0.1$). When colonies infected by DWV-B (status B and M) suffer very low mortality (fatality rates: $\nu_B = \nu_M < \nu_A$), DWB-B rises to a high equilibrium frequency and DWV-A is eliminated from the population of honey bee colonies. As the mortality of colonies infected by DWV-B (status B and M) rises above a threshold of $\nu_B = \nu_M = 0.083$, DWV-B is lost from the population and DWV-A reaches high equilibrium frequency.

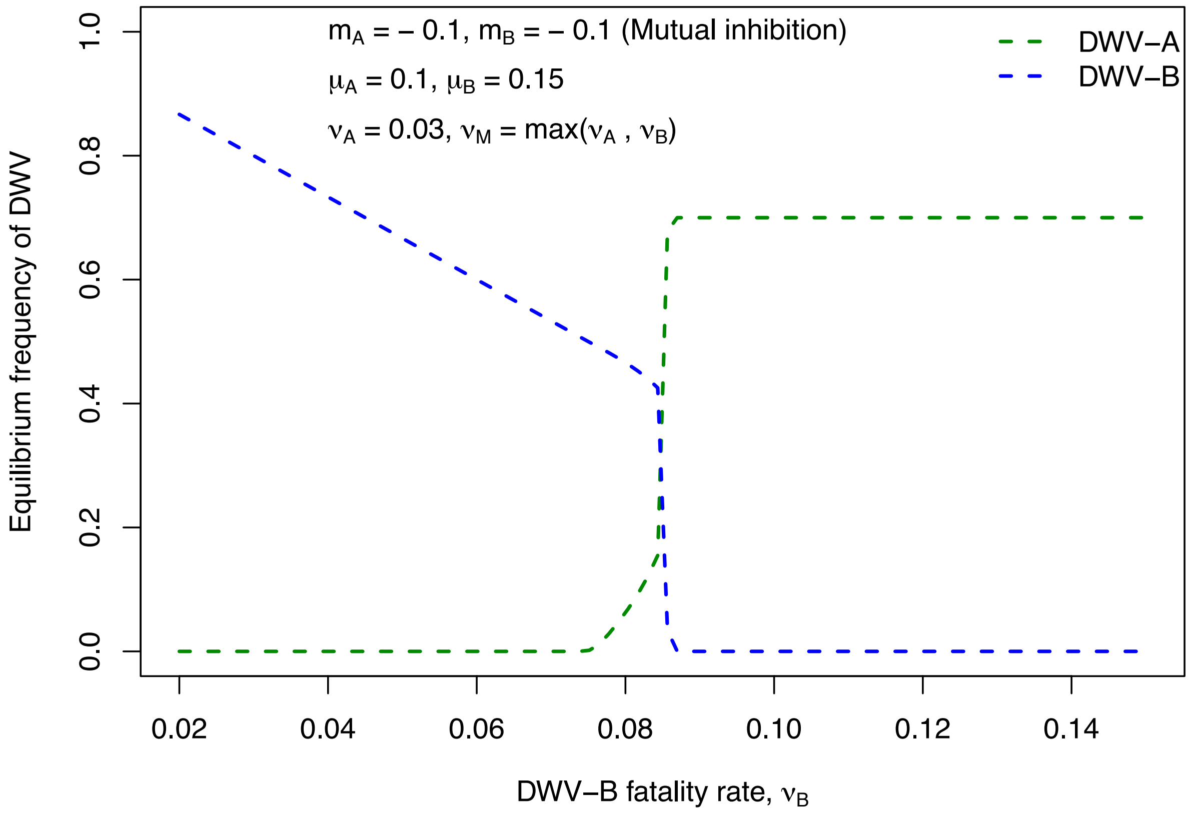

Supplementary Tables

Supplementary Table S1. NGS RNAseq reads in NCBI's SRA through to the end of 2021 for *Apis mellifera*, *Varroa destructor* and *Bombus terrestris* that were mapped to DWV-A and DWV-B.

<u>Justification for read alignment settings.</u>

Borrowing from the manuals to hisat2 and Bowtie2: the alignment scores in hisat2 and bowtie2 measure how genetically similar a read is to a reference. The score is calculated in a sophisticated way i.e. its value depends on the length of the read, the quality of bases in the read and the type of genetic change (mismatch vs indel). However, roughly speaking, hisat2, by default, returns reads with at least 95% genetic similarity (5 mismatches for a 100-nucleotide read). For Bowtie2, this number is about 90%. The mapping quality, on the other hand, measures the specificity with which a read can be mapped to a part of the genome. Mapping quality is defined as -10*log (p to the base of 10), where p is the probability that a read is mapped to the wrong place in the genome. A mapping quality of 10 and higher (as in our case) indicates at most 10% error in mapping position. Taking both alignment score and mapping quality, and considering that the genetic difference between DWV-A and B is ca. 17%, our read alignment settings allow us to detect DWV and to distinguish its two genotypes.

Supplementary Table S2. First records of DWV-B from a country or region in the literature, containing supplemental details to those provided in Table 3 of the manuscript.

Supplementary Table S3. Raw data on the prevalence of DWV-A and DWV-B at two time points in individual honey bees collected from flowers from seven sites in the UK.

Supplementary Table S4. Raw data on the prevalence of DWV-A and DWV-B at two time points in colonies of honey bees (one pool per colony) collected from collapsing colonies across Germany; years 2019 and 2020 were pooled for data presentation.

Supplementary Table S5. Raw data on the prevalence of DWV-A and DWV-B at two time points in NGS RNAseq reads of honey bees in Udine, NE Italy; data from years 2009-2013 and years 2018-2020 were each pooled for data presentation.

Table S1. NGS RNAseq reads in NCBI's SRA through to the end of 2021 for Apis mellifera, Varroa destructor and Bombus terrestris that were uniquely mapped to DWV-A and DWV-B.

Bioproject	lib	host	layout	RelDate	ColDate	EstColDate	country	region	Total Reads	DWV-A	Percent A	DWV-B	Percent B
PRJDB5837	DRR093876	Apis mellifera	single end	2019	NA	2017	Japan	Asia	57129472	2568825	4.5	28833	0.05
PRJDB5837	DRR093877	Apis mellifera	single end	2019	NA	2017	Japan	Asia	50627223	9740	0.02	123	0
PRJDB5837	DRR093878	Apis mellifera	single end	2019	NA	2017	Japan	Asia	58762218	11231	0.02	129	0
PRJDB5837	DRR093879	Apis mellifera	single end	2019	NA	2017	Japan	Asia	52660098	5741	0.01	74	0
PRJDB5837	DRR093880	Apis mellifera	single end	2019	NA	2017	Japan	Asia	53625763	74495	0.14	683	0
PRJDB5837	DRR093881	Apis mellifera	single end	2019	NA	2017	Japan	Asia	55993708	51510	0.09	418	0
PRJDB6980	DRR129201	Apis mellifera	single end	2018	2017	2017	Japan	Asia	857201	164	0.02	20	0
PRJDB6980	DRR129202	Apis mellifera	single end	2018	2017	2017	Japan	Asia	773045	12	0	0	0
PRJDB6980	DRR129203	Apis mellifera	single end	2018	2017	2017	Japan	Asia	751089	4	0	0	0
PRJDB6980	DRR129204	Apis mellifera	single end	2018	2017	2017	Japan	Asia	873506	0	0	0	0
PRJDB6980	DRR129205	Apis mellifera	single end	2018	2017	2017	Japan	Asia	1100788	0	0	0	0
PRJDB6980	DRR129206	Apis mellifera	single end	2018	2017	2017	Japan	Asia	1014845	4	0	0	0
PRJDB6980	DRR129207	Apis mellifera	single end	2018	2017	2017	Japan	Asia	1207299	0	0	0	0
PRJDB6980	DRR129208	Apis mellifera	single end	2018	2017	2017	Japan	Asia	772651	2	0	0	0
PRJDB6980	DRR129209	Apis mellifera	single end	2018	2017	2017	Japan	Asia	945803	7	0	0	0
PRJDB6980	DRR129210	Apis mellifera	single end	2018	2017	2017	Japan	Asia	937370	18	0	7	0
PRJDB6980	DRR129211	Apis mellifera	single end	2018	2017	2017	Japan	Asia	956121	6	0	0	0
PRJDB6980	DRR129212	Apis mellifera	single end	2018	2017	2017	Japan	Asia	993693	694	0.07	92	0.01
PRJDB6980	DRR129213	Apis mellifera	single end	2018	2017	2017	Japan	Asia	888771	99	0.01	4	0
PRJDB6980	DRR129214	Apis mellifera	single end	2018	2017	2017	Japan	Asia	1022733	247	0.02	22	0
PRJDB6980	DRR129215	Apis mellifera	single end	2018	2017	2017	Japan	Asia	943435	20	0	6	0
PRJDB6980	DRR129216	Apis mellifera	single end	2018	2017	2017	Japan	Asia	824672	34	0	5	0
PRJDB6980	DRR129217	Apis mellifera	single end	2018	2017	2017	Japan	Asia	951594	0	0	0	0
PRJDB6980	DRR129218	Apis mellifera	single end	2018	2017	2017	Japan	Asia	1128429	1	0	0	0
PRJDB6980	DRR129219	Apis mellifera	single end	2018	2017	2017	Japan	Asia	968535	0	0	0	0
PRJDB6980	DRR129220	Apis mellifera	single end	2018	2017	2017	Japan	Asia	805330	499095	61.97	21986	2.73
PRJEB1945	ERR274418	Apis mellifera	single end	2013	NA	2011	NA	NA	41112761	502	0	421	0
PRJEB1945	ERR274419	Apis mellifera	single end	2013	NA	2011	NA	NA	36895833	26772	0.07	27785	0.08
PRJEB1945	ERR274420	Apis mellifera	single end	2013	NA	2011	NA	NA	18581971	57	0	33	0
PRJEB1945	ERR274421	Apis mellifera	single end	2013	NA	2011	NA	NA	21706593	99	0	52	0
PRJEB1945	ERR274422	Apis mellifera	single end	2013	NA	2011	NA	NA	41408380	635	0	515	0
PRJEB1945	ERR274423	Apis mellifera	single end	2013	NA	2011	NA	NA	48206639	36003	0.07	37103	0.08
PRJEB1945	ERR274424	Apis mellifera	single end	2013	NA	2011	NA	NA	15071975	105	0	38	0
PRJNA122153	SRR042523	Apis mellifera	single end	2010	NA	2008	NA	NA	9027681	23	0	0	0
PRJNA562696	SRR10030209	Apis mellifera	single end	2019	2014	2014	USA	North America	32614630	1	0	0	0
PRJNA562696	SRR10030210	Apis mellifera	single end	2019	2014	2014	USA	North America	29713036	0	0	0	0
PRJNA562696	SRR10030211	Apis mellifera	single end	2019	2014	2014	USA	North America	34634718	34	0	0	0
PRJNA562696	SRR10030212	Apis mellifera	single end	2019	2014	2014	USA	North America	31649640	7	0	0	0
PRJNA562696	SRR10030213	Apis mellifera	single end	2019	2014	2014	USA	North America	23825381	1	0	0	0
PRJNA562696	SRR10030214	Apis mellifera	single end	2019	2014	2014	USA	North America	31647319	1	0	0	0
PRJNA562696	SRR10030215	Apis mellifera	single end	2019	2014	2014	USA	North America	33440508	2	0	0	0
PRJNA562696	SRR10030216	Apis mellifera	single end	2019	2014	2014	USA	North America	30176365	10	0	0	0
PRJNA562696	SRR10030217	Apis mellifera	single end	2019	2014	2014	USA	North America	32969244	4	0	0	0
PRJNA562696	SRR10030218	Apis mellifera	single end	2019	2014	2014	USA	North America	36437471	3	0	0	0
PRJNA562696	SRR10030219	Apis mellifera	single end	2019	2014	2014	USA	North America	33168309	12	0	0	0
PRJNA562696	SRR10030220	Apis mellifera	single end	2019	2014	2014	USA	North America	31004697	7	0	0	0
PRJNA562696	SRR10030221	Apis mellifera	single end	2019	2014	2014	USA	North America	28700071	1	0	0	0
PRJNA562696	SRR10030222	Apis mellifera	single end	2019	2014	2014	USA	North America	33115693	1	0	0	0
PRJNA562696	SRR10030223	Apis mellifera	single end	2019	2014	2014	USA	North America	36005390	0	0	0	0
PRJNA562696	SRR10030224	Apis mellifera	single end	2019	2014	2014	USA	North America	30120743	1	0	0	0
PRJNA562696	SRR10030225	Apis mellifera	single end	2019	2014	2014	USA	North America	36298267	29	0	0	0
PRJNA562696	SRR10030226	Apis mellifera	single end	2019	2014	2014	USA	North America	26587078	0	0	0	0
PRJNA562696	SRR10030227	Apis mellifera	single end	2019	2014	2014	USA	North America	37707929	0	0	0	0
PRJNA562696	SRR10030228	Apis mellifera	single end	2019	2014	2014	USA	North America	30246822	8	0	0	0
PRJNA562696	SRR10030229	Apis mellifera	single end	2019	2014	2014	USA	North America	33794604	1	0	0	0
PRJNA562696	SRR10030230	Apis mellifera	single end	2019	2014	2014	USA	North America	29240999	0	0	0	0
PRJNA562696	SRR10030231	Apis mellifera	single end	2019	2014	2014	USA	North America	39154997	10	0	0	0
PRJNA562696	SRR10030232	Apis mellifera	single end	2019	2014	2014	USA	North America	27007871	1	0	0	0
PRJNA562696	SRR10030233	Apis mellifera	single end	2019	2014	2014	USA	North America	31499016	0	0	0	0
PRJNA562696	SRR10030234	Apis mellifera	single end	2019	2014	2014	USA	North America	25110163	129	0	0	0
PRJNA562696	SRR10030235	Apis mellifera	single end	2019	2014	2014	USA	North America	32193740	4	0	3	0
PRJNA562696	SRR10030236	Apis mellifera	single end	2019	2014	2014	USA	North America	29796432	40	0	0	0
PRJNA562696	SRR10030237	Apis mellifera	single end	2019	2014	2014	USA	North America	32160988	0	0	0	0
PRJNA562696	SRR10030238	Apis mellifera	single end	2019	2014	2014	USA	North America	28965239	60	0	0	0
PRJNA562696	SRR10030239	Apis mellifera	single end	2019	2014	2014	USA	North America	28875400	5	0	0	0
PRJNA562696	SRR10030240	Apis mellifera	single end	2019	2014	2014	USA	North America	34664847	0	0	0	0
PRJNA562696	SRR10030241	Apis mellifera	single end	2019	2014	2014	USA	North America	35673668	183	0	2	0
PRJNA562696	SRR10030242	Apis mellifera	single end	2019	2014	2014	USA	North America	35212384	50	0	0	0
PRJNA562696	SRR10030243	Apis mellifera	single end	2019	2014	2014	USA	North America	30175324	3	0	0	0
PRJNA562696	SRR10030244	Apis mellifera	single end	2019	2014	2014	USA	North America	25108157	10	0	0	0
PRJNA562696	SRR10030245	Apis mellifera	single end	2019	2014	2014	USA	North America	29543474	23	0	1	0
PRJNA562696	SRR10030246	Apis mellifera	single end	2019	2014	2014	USA	North America	28064601	0	0	0	0
PRJNA562696	SRR10030247	Apis mellifera	single end	2019	2014	2014	USA	North America	23675284	6683	0.03	38	0
PRJNA562696	SRR10030248	Apis mellifera	single end	2019	2014	2014	USA	North America	30189225	21	0	0	0
PRJNA562696	SRR10030249	Apis mellifera	single end	2019	2014	2014	USA	North America	30774849	0	0	0	0
PRJNA562696	SRR10030250	Apis mellifera	single end	2019	2014	2014	USA	North America	28234128	80	0	1	0
PRJNA562696	SRR10030251	Apis mellifera	single end	2019	2014	2014	USA	North America	31756578	3	0	0	0
PRJNA562696	SRR10030252	Apis mellifera	single end	2019	2014	2014	USA	North America	31415328	0	0	0	0
PRJNA562696	SRR10030253	Apis mellifera	single end	2019	2014	2014	USA	North America	28804355	3	0	0	0
PRJNA562696	SRR10030254	Apis mellifera	single end	2019	2014	2014	USA	North America	30910002	4	0	0	0
PRJNA562696	SRR10030255	Apis mellifera	single end	2019	2014	2014	USA	North America	28403726	5	0	0	0
PRJNA562696	SRR10030256	Apis mellifera	single end	2019	2014	2014	USA	North America	31536486	2	0	0	0
PRJNA562696	SRR10030257	Apis mellifera	single end	2019	2014	2014	USA	North America	30427068	0	0	1	0
PRJNA562696	SRR10030258	Apis mellifera	single end	2019	2014	2014	USA	North America	30082199	11	0	0	0
PRJNA562696	SRR10030259	Apis mellifera	single end	2019	2014	2014	USA	North America	30126353	1	0	0	0
PRJNA562696	SRR10030260	Apis mellifera	single end	2019	2014	2014	USA	North America	33933530	0	0	0	0
PRJNA562696	SRR10030261	Apis mellifera	single end	2019	2014	2014	USA	North America	31588132	5	0	0	0
PRJNA562696	SRR10030262	Apis mellifera	single end	2019	2014	2014	USA	North America	33358022	1	0	0	0
PRJNA562696	SRR10030263	Apis mellifera	single end	2019	2014	2014	USA	North America	29822880	1	0	0	0
PRJNA562696	SRR10030264	Apis mellifera	single end	2019	2014	2014	USA	North America	31820211	14	0	0	0
PRJNA562696	SRR10030265	Apis mellifera	single end	2019	2014	2014	USA	North America	29733517	2	0	0	0
PRJNA562696	SRR10030266	Apis mellifera	single end	2019	2014	2014	USA	North America	32521540	0	0	0	0
PRJNA562696	SRR10030267	Apis mellifera	single end	2019	2014	2014	USA	North America	30823787	20	0	0	0
PRJNA562696	SRR10030268	Apis mellifera	single end	2019	2014	2014	USA	North America	35373547	1	0	0	0
PRJNA562696	SRR10030269	Apis mellifera	single end	2019	2014	2014	USA	North America	33177396	1	0	0	0
PRJNA562696	SRR10030270	Apis mellifera	single end	2019	2014	2014	USA	North America	32426322	0	0	0	0
PRJNA562696	SRR10030271	Apis mellifera	single end	2019	2014	2014	USA	North America	32426925	2	0	0	0
PRJNA562696	SRR10030272	Apis mellifera	single end	2019	2014	2014	USA	North America	38325918	3	0	0	0
PRJNA562696	SRR10030273	Apis mellifera	single end	2019	2014	2014	USA	North America	30250505	0	0	0	0
PRJNA562696	SRR10030274	Apis mellifera	single end	2019	2014	2014	USA	North America	40538496	17	0	0	0
PRJNA562696	SRR10030275	Apis mellifera	single end	2019	2014	2014	USA	North America	36735704	35	0	0	0
PRJNA562696	SRR10030276	Apis mellifera	single end	2019	2014	2014	USA	North America	30342981	19	0	0	0
PRJNA562696	SRR10030277	Apis mellifera	single end	2019	2014	2014	USA	North America	27138195	5	0	0	0
PRJNA562696	SRR10030278	Apis mellifera	single end	2019	2014	2014	USA	North America	28218754	7	0	0	0

PRJNA562696	SRR10030279	Apis mellifera	single end	2019	2014	2014	USA	North America	27724574	0	0	0	0
PRJNA562696	SRR10030280	Apis mellifera	single end	2019	2014	2014	USA	North America	31111929	1	0	0	0
PRJNA227348	SRR1028781	Apis mellifera	single end	2015	NA	2013	NA	NA	7344483	0	0	0	0
PRJNA227348	SRR1028782	Apis mellifera	single end	2015	NA	2013	NA	NA	6968503	0	0	0	0
PRJNA227348	SRR1028783	Apis mellifera	single end	2015	NA	2013	NA	NA	7326580	0	0	0	0
PRJNA227348	SRR1028784	Apis mellifera	single end	2015	NA	2013	NA	NA	7371304	0	0	0	0
PRJNA579390	SRR10343221	Apis mellifera	single end	2020	NA	2018	NA	NA	33454166	0	0	0	0
PRJNA579390	SRR10343222	Apis mellifera	single end	2020	NA	2018	NA	NA	33454166	0	0	0	0
PRJNA579390	SRR10343223	Apis mellifera	single end	2020	NA	2018	NA	NA	36223497	6	0	0	0
PRJNA579390	SRR10343224	Apis mellifera	single end	2020	NA	2018	NA	NA	36223497	6	0	0	0
PRJNA579390	SRR10343225	Apis mellifera	single end	2020	NA	2018	NA	NA	34378792	15	0	2	0
PRJNA579390	SRR10343226	Apis mellifera	single end	2020	NA	2018	NA	NA	34378792	16	0	2	0
PRJNA579390	SRR10343227	Apis mellifera	single end	2020	NA	2018	NA	NA	32150794	8	0	0	0
PRJNA579390	SRR10343228	Apis mellifera	single end	2020	NA	2018	NA	NA	32150794	8	0	0	0
PRJNA579390	SRR10343229	Apis mellifera	single end	2020	NA	2018	NA	NA	33277891	31	0	0	0
PRJNA579390	SRR10343230	Apis mellifera	single end	2020	NA	2018	NA	NA	33277891	30	0	0	0
PRJNA579390	SRR10343231	Apis mellifera	single end	2020	NA	2018	NA	NA	33648919	5	0	0	0
PRJNA579390	SRR10343232	Apis mellifera	single end	2020	NA	2018	NA	NA	33648919	5	0	0	0
PRJNA229500	SRR1035614	Apis mellifera	single end	2015	NA	2013	NA	NA	11405509	0	0	0	0
PRJNA229500	SRR1035615	Apis mellifera	single end	2015	NA	2013	NA	NA	10618686	4	0	0	0
PRJNA228948	SRR1036397	Apis mellifera	single end	2015	2012	2012	China	Asia	18038916	0	0	0	0
PRJNA228948	SRR1036398	Apis mellifera	single end	2015	2012	2012	China	Asia	12000000	0	0	0	0
PRJNA228948	SRR1036399	Apis mellifera	single end	2015	2012	2012	China	Asia	12000000	0	0	0	0
PRJNA593999	SRR10598578	Apis mellifera	single end	2020	2016	2016	USA	North America	32352831	8	0	0	0
PRJNA593999	SRR10598589	Apis mellifera	single end	2020	2016	2016	USA	North America	31429725	2	0	0	0
PRJNA593999	SRR10598600	Apis mellifera	single end	2020	2016	2016	USA	North America	31670305	26	0	0	0
PRJNA593999	SRR10598611	Apis mellifera	single end	2020	2016	2016	USA	North America	38222182	16	0	0	0
PRJNA593999	SRR10598622	Apis mellifera	single end	2020	2016	2016	USA	North America	31418775	79	0	0	0
PRJNA593999	SRR10598628	Apis mellifera	single end	2020	2016	2016	USA	North America	39411076	1	0	0	0
PRJNA593999	SRR10598629	Apis mellifera	single end	2020	2016	2016	USA	North America	31352405	0	0	0	0
PRJNA593999	SRR10598630	Apis mellifera	single end	2020	2016	2016	USA	North America	35428104	1	0	0	0
PRJNA593999	SRR10598631	Apis mellifera	single end	2020	2016	2016	USA	North America	33780723	0	0	0	0
PRJNA593999	SRR10598632	Apis mellifera	single end	2020	2016	2016	USA	North America	27917610	1	0	0	0
PRJNA593999	SRR10598633	Apis mellifera	single end	2020	2016	2016	USA	North America	34929511	40	0	0	0
PRJNA593999	SRR10598634	Apis mellifera	single end	2020	2016	2016	USA	North America	29170272	1	0	0	0
PRJNA593999	SRR10598635	Apis mellifera	single end	2020	2016	2016	USA	North America	34755595	1	0	0	0
PRJNA593999	SRR10598636	Apis mellifera	single end	2020	2016	2016	USA	North America	34926669	0	0	1	0
PRJNA593999	SRR10598637	Apis mellifera	single end	2020	2016	2016	USA	North America	32913814	1	0	0	0
PRJNA593999	SRR10598638	Apis mellifera	single end	2020	2016	2016	USA	North America	36882580	1	0	0	0
PRJNA593999	SRR10598639	Apis mellifera	single end	2020	2016	2016	USA	North America	32286134	0	0	0	0
PRJNA593999	SRR10598640	Apis mellifera	single end	2020	2016	2016	USA	North America	34584539	3	0	0	0
PRJNA593999	SRR10598641	Apis mellifera	single end	2020	2016	2016	USA	North America	30171589	0	0	0	0
PRJNA593999	SRR10598642	Apis mellifera	single end	2020	2016	2016	USA	North America	37144651	11	0	1	0
PRJNA593999	SRR10598643	Apis mellifera	single end	2020	2016	2016	USA	North America	30935496	2	0	1	0
PRJNA593999	SRR10598644	Apis mellifera	single end	2020	2016	2016	USA	North America	29457655	0	0	0	0
PRJNA593999	SRR10598645	Apis mellifera	single end	2020	2016	2016	USA	North America	31266511	1	0	0	0
PRJNA593999	SRR10598646	Apis mellifera	single end	2020	2016	2016	USA	North America	29556176	0	0	0	0
PRJNA593999	SRR10598647	Apis mellifera	single end	2020	2016	2016	USA	North America	39494637	1	0	0	0
PRJNA593999	SRR10598648	Apis mellifera	single end	2020	2016	2016	USA	North America	32190009	0	0	0	0
PRJNA593999	SRR10598649	Apis mellifera	single end	2020	2016	2016	USA	North America	29664697	7	0	0	0
PRJNA593999	SRR10598650	Apis mellifera	single end	2020	2016	2016	USA	North America	31500589	0	0	0	0
PRJNA593999	SRR10598651	Apis mellifera	single end	2020	2016	2016	USA	North America	33029606	2	0	0	0
PRJNA593999	SRR10598652	Apis mellifera	single end	2020	2016	2016	USA	North America	30789203	0	0	0	0
PRJNA593999	SRR10598653	Apis mellifera	single end	2020	2016	2016	USA	North America	33190502	0	0	1	0
PRJNA593999	SRR10598654	Apis mellifera	single end	2020	2016	2016	USA	North America	33552368	3	0	0	0
PRJNA593999	SRR10598655	Apis mellifera	single end	2020	2016	2016	USA	North America	29589656	6	0	0	0
PRJNA593999	SRR10598656	Apis mellifera	single end	2020	2016	2016	USA	North America	34310005	0	0	0	0
PRJNA593999	SRR10598657	Apis mellifera	single end	2020	2016	2016	USA	North America	35673640	0	0	0	0
PRJNA593999	SRR10598658	Apis mellifera	single end	2020	2016	2016	USA	North America	37476319	0	0	0	0
PRJNA593999	SRR10598659	Apis mellifera	single end	2020	2016	2016	USA	North America	37813209	0	0	0	0
PRJNA593999	SRR10598660	Apis mellifera	single end	2020	2016	2016	USA	North America	44269153	11	0	0	0
PRJNA593999	SRR10598661	Apis mellifera	single end	2020	2016	2016	USA	North America	34438409	29	0	0	0
PRJNA593999	SRR10598662	Apis mellifera	single end	2020	2016	2016	USA	North America	30983454	1	0	0	0
PRJNA593999	SRR10598663	Apis mellifera	single end	2020	2016	2016	USA	North America	27452046	57	0	0	0
PRJNA593999	SRR10598664	Apis mellifera	single end	2020	2016	2016	USA	North America	28952717	67	0	0	0
PRJNA593999	SRR10598665	Apis mellifera	single end	2020	2016	2016	USA	North America	31498346	44	0	0	0
PRJNA593999	SRR10598666	Apis mellifera	single end	2020	2016	2016	USA	North America	30234588	1	0	0	0
PRJNA593999	SRR10598667	Apis mellifera	single end	2020	2016	2016	USA	North America	29582894	7	0	0	0
PRJNA594208	SRR10606709	Apis mellifera	single end	2020	NA	2018	NA	NA	25971713	1	0	0	0
PRJNA599219	SRR10836842	Apis mellifera	single end	2020	NA	2018	NA	NA	11090069	0	0	0	0
PRJNA599219	SRR10836843	Apis mellifera	single end	2020	NA	2018	NA	NA	8856370	0	0	0	0
PRJNA599219	SRR10836844	Apis mellifera	single end	2020	NA	2018	NA	NA	9937948	0	0	0	0
PRJNA599219	SRR10836845	Apis mellifera	single end	2020	NA	2018	NA	NA	11301110	0	0	0	0
PRJNA599219	SRR10836846	Apis mellifera	single end	2020	NA	2018	NA	NA	9483685	0	0	0	0
PRJNA599219	SRR10836847	Apis mellifera	single end	2020	NA	2018	NA	NA	9762272	0	0	0	0
PRJNA599219	SRR10836848	Apis mellifera	single end	2020	NA	2018	NA	NA	9380950	0	0	0	0
PRJNA599219	SRR10836849	Apis mellifera	single end	2020	NA	2018	NA	NA	9583287	0	0	0	0
PRJNA599219	SRR10836850	Apis mellifera	single end	2020	NA	2018	NA	NA	9705893	0	0	0	0
PRJNA599219	SRR10836851	Apis mellifera	single end	2020	NA	2018	NA	NA	8486899	0	0	0	0
PRJNA599219	SRR10836852	Apis mellifera	single end	2020	NA	2018	NA	NA	26423213	0	0	0	0
PRJNA599219	SRR10836853	Apis mellifera	single end	2020	NA	2018	NA	NA	11761823	0	0	0	0
PRJNA599219	SRR10836854	Apis mellifera	single end	2020	NA	2018	NA	NA	8827719	0	0	0	0
PRJNA599219	SRR10836855	Apis mellifera	single end	2020	NA	2018	NA	NA	10812212	0	0	0	0
PRJNA599219	SRR10836856	Apis mellifera	single end	2020	NA	2018	NA	NA	7301835	0	0	0	0
PRJNA607066	SRR11096262	Apis mellifera	single end	2020	NA	2018	USA	North America	31331290	10055532	32.09	2324	0.01
PRJNA607066	SRR11096263	Apis mellifera	single end	2020	NA	2018	USA	North America	33802741	7530234	22.28	1627	0
PRJNA607066	SRR11096264	Apis mellifera	single end	2020	NA	2018	USA	North America	29310031	34062	0.12	105	0
PRJNA607066	SRR11096265	Apis mellifera	single end	2020	NA	2018	USA	North America	31202159	45667	0.15	101	0
PRJNA607066	SRR11096266	Apis mellifera	single end	2020	NA	2018	USA	North America	31311515	38411	0.12	101	0
PRJNA607066	SRR11096267	Apis mellifera	single end	2020	NA	2018	USA	North America	25318759	3587056	14.17	507	0
PRJNA607066	SRR11096268	Apis mellifera	single end	2020	NA	2018	USA	North America	28370097	5326275	18.77	701	0
PRJNA607066	SRR11096269	Apis mellifera	single end	2020	NA	2018	USA	North America	23929953	7342089	30.68	1254	0.01
PRJNA607066	SRR11096270	Apis mellifera	single end	2020	NA	2018	USA	North America	30476652	7558083	24.8	1629	0.01
PRJNA607066	SRR11096271	Apis mellifera	single end	2020	NA	2018	USA	North America	31398202	8917085	28.4	1841	0.01
PRJNA607066	SRR11096272	Apis mellifera	single end	2020	NA	2018	USA	North America	41907494	10668879	25.46	2197	0.01
PRJNA607066	SRR11096273	Apis mellifera	single end	2020	NA	2018	USA	North America	29162295	8839992	30.31	1515	0.01
PRJNA607066	SRR11096274	Apis mellifera	single end	2020	NA	2018	USA	North America	31250407	9531648	30.5	7491	0.02
PRJNA607066	SRR11096275	Apis mellifera	single end	2020	NA	2018	USA	North America	37543684	11011751	29.33	1923	0.01
PRJNA607066	SRR11096276	Apis mellifera	single end	2020	NA	2018	USA	North America	35102188	52418	0.15	116	0
PRJNA607066	SRR11096277	Apis mellifera	single end	2020	NA	2018	USA	North America	26005588	9601332	36.92	1321	0.01
PRJNA607066	SRR11096278	Apis mellifera	single end	2020	NA	2018	USA	North America	26894214	37627	0.14	97	0
PRJNA607066	SRR11096279	Apis mellifera	single end	2020	NA	2018	USA	North America	33798302	12715964	37.62	2273	0.01
PRJNA607066	SRR11096280	Apis mellifera	single end	2020	NA	2018	USA	North America	28285408	52272	0.18	123	0
PRJNA607066	SRR11096281	Apis mellifera	single end	2020	NA	2018	USA	North America	24793202	44584	0.18	93	0
PRJNA607066	SRR11096282	Apis mellifera	single end	2020	NA	2018	USA	North America	39563149	69047	0.17	112	0
PRJNA607066	SRR11096283	Apis mellifera	single end	2020	NA	2018	USA	North America	25116271	45808	0.18	64	0

PRJNA607066	SRR11096284	Apis mellifera	single end	2020	NA	2018	USA	North America	31935561	58363	0.18	139	0
PRJNA607066	SRR11096285	Apis mellifera	single end	2020	NA	2018	USA	North America	28524988	15876275	55.66	2526	0.01
PRJNA607066	SRR11096286	Apis mellifera	single end	2020	NA	2018	USA	North America	37137899	19544648	52.63	3136	0.01
PRJNA607066	SRR11096287	Apis mellifera	single end	2020	NA	2018	USA	North America	39450826	23128260	58.63	3709	0.01
PRJNA607066	SRR11096288	Apis mellifera	single end	2020	NA	2018	USA	North America	49107805	23739989	48.34	4959	0.01
PRJNA607066	SRR11096289	Apis mellifera	single end	2020	NA	2018	USA	North America	26294610	13993944	53.22	10860	0.04
PRJNA607066	SRR11096290	Apis mellifera	single end	2020	NA	2018	USA	North America	22727980	16255431	71.52	1939	0.01
PRJNA607066	SRR11096291	Apis mellifera	single end	2020	NA	2018	USA	North America	27066957	14310094	52.87	2315	0.01
PRJNA607066	SRR11096292	Apis mellifera	single end	2020	NA	2018	USA	North America	33949001	21661095	63.8	19732	0.06
PRJNA607066	SRR11096293	Apis mellifera	single end	2020	NA	2018	USA	North America	41659417	24284051	58.29	4341	0.01
PRJNA607066	SRR11096294	Apis mellifera	single end	2020	NA	2018	USA	North America	39860922	24365030	61.13	4182	0.01
PRJNA607066	SRR11096295	Apis mellifera	single end	2020	NA	2018	USA	North America	29992766	18615771	62.07	2509	0.01
PRJNA607066	SRR11096296	Apis mellifera	single end	2020	NA	2018	USA	North America	34498511	17155098	49.73	2433	0.01
PRJNA607066	SRR11096297	Apis mellifera	single end	2020	NA	2018	USA	North America	27050635	15170089	56.08	2112	0.01
PRJNA607066	SRR11096298	Apis mellifera	single end	2020	NA	2018	USA	North America	28676148	19754737	68.89	3389	0.01
PRJNA607066	SRR11096299	Apis mellifera	single end	2020	NA	2018	USA	North America	28211893	15789650	55.97	1911	0.01
PRJNA616244	SRR11447325	Apis mellifera	single end	2020	NA	2018	NA	NA	53240318	1	0	27	0
PRJNA616244	SRR11447326	Apis mellifera	single end	2020	NA	2018	NA	NA	49588827	0	0	0	0
PRJNA616244	SRR11447327	Apis mellifera	single end	2020	NA	2018	NA	NA	50572593	0	0	17	0
PRJNA616244	SRR11447328	Apis mellifera	single end	2020	NA	2018	NA	NA	43635753	4	0	212	0
PRJNA616244	SRR11447329	Apis mellifera	single end	2020	NA	2018	NA	NA	46112509	6	0	51	0
PRJNA616244	SRR11447330	Apis mellifera	single end	2020	NA	2018	NA	NA	49695577	3	0	3	0
PRJNA616244	SRR11447331	Apis mellifera	single end	2020	NA	2018	NA	NA	50823552	1	0	1	0
PRJNA616244	SRR11447332	Apis mellifera	single end	2020	NA	2018	NA	NA	42822349	2	0	7	0
PRJNA616244	SRR11447333	Apis mellifera	single end	2020	NA	2018	NA	NA	46033632	3	0	9	0
PRJNA616244	SRR11447334	Apis mellifera	single end	2020	NA	2018	NA	NA	50345141	2	0	0	0
PRJNA631022	SRR11787192	Apis mellifera	single end	2021	2014	2014	Brazil	South America	26737359	3	0	0	0
PRJNA631022	SRR11787193	Apis mellifera	single end	2021	2013	2013	Brazil	South America	17450484	0	0	1	0
PRJNA631022	SRR11787194	Apis mellifera	single end	2021	2012	2012	Brazil	South America	33968207	427	0	460	0
PRJNA631022	SRR11787195	Apis mellifera	single end	2021	2011	2011	Brazil	South America	16477446	6	0	14	0
PRJNA631022	SRR11787196	Apis mellifera	single end	2021	2016	2016	Brazil	South America	46114520	0	0	0	0
PRJNA631022	SRR11787197	Apis mellifera	single end	2021	2015	2015	Brazil	South America	27422580	0	0	0	0
PRJNA631022	SRR11787198	Apis mellifera	single end	2021	2014	2014	Brazil	South America	34388243	35	0	33	0
PRJNA631022	SRR11787199	Apis mellifera	single end	2021	2013	2013	Brazil	South America	15446157	0	0	1	0
PRJNA631022	SRR11787200	Apis mellifera	single end	2021	2012	2012	Brazil	South America	18885127	0	0	1	0
PRJNA631022	SRR11787201	Apis mellifera	single end	2021	2011	2011	Brazil	South America	19966313	47	0	34	0
PRJNA244920	SRR1254941	Apis mellifera	single end	2018	NA	2016	NA	NA	8626009	22	0	1	0
PRJNA244920	SRR1254942	Apis mellifera	single end	2018	NA	2016	NA	NA	8957903	66118	0.74	2423	0.03
PRJNA244920	SRR1254943	Apis mellifera	single end	2018	NA	2016	NA	NA	7586964	14	0	0	0
PRJNA661322	SRR12584096	Apis mellifera	single end	2020	2017	2017	USA	North America	4271308	9307	0.22	3	0
PRJNA661322	SRR12584097	Apis mellifera	single end	2020	2017	2017	USA	North America	4587587	158969	3.47	39	0
PRJNA661322	SRR12584098	Apis mellifera	single end	2020	2017	2017	USA	North America	4544013	156469	3.44	48	0
PRJNA661322	SRR12584099	Apis mellifera	single end	2020	2017	2017	USA	North America	4393379	150939	3.44	35	0
PRJNA661322	SRR12584100	Apis mellifera	single end	2020	2017	2017	USA	North America	4908374	11458	0.23	6	0
PRJNA661322	SRR12584101	Apis mellifera	single end	2020	2017	2017	USA	North America	4875263	12319	0.25	3	0
PRJNA661322	SRR12584102	Apis mellifera	single end	2020	2017	2017	USA	North America	4723354	12666	0.27	4	0
PRJNA661322	SRR12584103	Apis mellifera	single end	2020	2017	2017	USA	North America	5668938	1985	0.04	2	0
PRJNA661322	SRR12584104	Apis mellifera	single end	2020	2017	2017	USA	North America	5580542	2544	0.05	2	0
PRJNA661322	SRR12584105	Apis mellifera	single end	2020	2017	2017	USA	North America	5453653	2783	0.05	2	0
PRJNA661322	SRR12584106	Apis mellifera	single end	2020	2017	2017	USA	North America	2918971	229559	7.86	31	0
PRJNA661322	SRR12584107	Apis mellifera	single end	2020	2017	2017	USA	North America	5650897	1594	0.03	1	0
PRJNA661322	SRR12584108	Apis mellifera	single end	2020	2017	2017	USA	North America	5578723	2214	0.04	2	0
PRJNA661322	SRR12584109	Apis mellifera	single end	2020	2017	2017	USA	North America	5393079	2417	0.04	3	0
PRJNA661322	SRR12584110	Apis mellifera	single end	2020	2017	2017	USA	North America	4723753	1271	0.03	0	0
PRJNA661322	SRR12584111	Apis mellifera	single end	2020	2017	2017	USA	North America	4675412	1691	0.04	1	0
PRJNA661322	SRR12584112	Apis mellifera	single end	2020	2017	2017	USA	North America	4547289	1764	0.04	1	0
PRJNA661322	SRR12584113	Apis mellifera	single end	2020	2017	2017	USA	North America	2254489	223221	9.9	72	0
PRJNA661322	SRR12584114	Apis mellifera	single end	2020	2017	2017	USA	North America	2259647	221027	9.78	55	0
PRJNA661322	SRR12584115	Apis mellifera	single end	2020	2017	2017	USA	North America	2269245	223039	9.83	48	0
PRJNA661322	SRR12584116	Apis mellifera	single end	2020	2017	2017	USA	North America	2967118	270135	9.1	23	0
PRJNA661322	SRR12584117	Apis mellifera	single end	2020	2017	2017	USA	North America	2068919	2125	0.1	0	0
PRJNA661322	SRR12584118	Apis mellifera	single end	2020	2017	2017	USA	North America	3034331	279128	9.2	22	0
PRJNA661322	SRR12584119	Apis mellifera	single end	2020	2017	2017	USA	North America	2992440	272498	9.11	25	0
PRJNA661322	SRR12584120	Apis mellifera	single end	2020	2017	2017	USA	North America	2334073	746307	31.97	59	0
PRJNA661322	SRR12584121	Apis mellifera	single end	2020	2017	2017	USA	North America	2388791	766806	32.1	59	0
PRJNA661322	SRR12584122	Apis mellifera	single end	2020	2017	2017	USA	North America	2360872	748570	31.71	47	0
PRJNA661322	SRR12584123	Apis mellifera	single end	2020	2017	2017	USA	North America	2251587	1563	0.07	0	0
PRJNA661322	SRR12584124	Apis mellifera	single end	2020	2017	2017	USA	North America	2294731	1818	0.08	1	0
PRJNA661322	SRR12584125	Apis mellifera	single end	2020	2017	2017	USA	North America	2284963	1920	0.08	0	0
PRJNA661322	SRR12584126	Apis mellifera	single end	2020	2017	2017	USA	North America	2648451	1334	0.05	1	0
PRJNA661322	SRR12584127	Apis mellifera	single end	2020	2017	2017	USA	North America	2684469	1496	0.06	0	0
PRJNA661322	SRR12584128	Apis mellifera	single end	2020	2017	2017	USA	North America	2127710	2435	0.11	0	0
PRJNA661322	SRR12584129	Apis mellifera	single end	2020	2017	2017	USA	North America	2665666	1621	0.06	2	0
PRJNA661322	SRR12584130	Apis mellifera	single end	2020	2017	2017	USA	North America	2330936	502130	21.54	74	0
PRJNA661322	SRR12584131	Apis mellifera	single end	2020	2017	2017	USA	North America	2372591	513647	21.65	70	0
PRJNA661322	SRR12584132	Apis mellifera	single end	2020	2017	2017	USA	North America	2350479	502721	21.39	80	0
PRJNA661322	SRR12584133	Apis mellifera	single end	2020	2017	2017	USA	North America	2611271	663218	25.4	154	0.01
PRJNA661322	SRR12584134	Apis mellifera	single end	2020	2017	2017	USA	North America	2657107	680349	25.6	129	0
PRJNA661322	SRR12584135	Apis mellifera	single end	2020	2017	2017	USA	North America	2637869	668882	25.36	132	0.01
PRJNA661322	SRR12584136	Apis mellifera	single end	2020	2017	2017	USA	North America	3382777	1491658	44.1	130	0
PRJNA661322	SRR12584137	Apis mellifera	single end	2020	2017	2017	USA	North America	3469932	1532951	44.18	113	0
PRJNA661322	SRR12584138	Apis mellifera	single end	2020	2017	2017	USA	North America	3434161	1501510	43.72	163	0
PRJNA661322	SRR12584139	Apis mellifera	single end	2020	2017	2017	USA	North America	2094387	2545	0.12	1	0
PRJNA661322	SRR12584140	Apis mellifera	single end	2020	2017	2017	USA	North America	2977158	414024	13.91	76	0
PRJNA661322	SRR12584141	Apis mellifera	single end	2020	2017	2017	USA	North America	2986954	410816	13.75	82	0
PRJNA661322	SRR12584142	Apis mellifera	single end	2020	2017	2017	USA	North America	3020486	414535	13.72	84	0
PRJNA661322	SRR12584143	Apis mellifera	single end	2020	2017	2017	USA	North America	3604870	2716	0.08	0	0
PRJNA661322	SRR12584144	Apis mellifera	single end	2020	2017	2017	USA	North America	3666763	2854	0.08	1	0
PRJNA661322	SRR12584145	Apis mellifera	single end	2020	2017	2017	USA	North America	3659623	3235	0.09	3	0
PRJNA661322	SRR12584146	Apis mellifera	single end	2020	2017	2017	USA	North America	2133447	508088	23.82	48	0
PRJNA661322	SRR12584147	Apis mellifera	single end	2020	2017	2017	USA	North America	2137261	518435	24.26	42	0
PRJNA661322	SRR12584148	Apis mellifera	single end	2020	2017	2017	USA	North America	2162866	509997	23.58	49	0
PRJNA661322	SRR12584149	Apis mellifera	single end	2020	2017	2017	USA	North America	2423917	381625	15.74	30	0
PRJNA661322	SRR12584150	Apis mellifera	single end	2020	2017	2017	USA	North America	4287754	582198	13.58	81	0
PRJNA661322	SRR12584151	Apis mellifera	single end	2020	2017	2017	USA	North America	2456626	390280	15.89	40	0
PRJNA661322	SRR12584152	Apis mellifera	single end	2020	2017	2017	USA	North America	2440404	383395	15.71	34	0
PRJNA661322	SRR12584153	Apis mellifera	single end	2020	2017	2017	USA	North America	3014739	1008613	33.46	185	0.01
PRJNA661322	SRR12584154	Apis mellifera	single end	2020	2017	2017	USA	North America	3092099	1033179	33.41	220	0.01
PRJNA661322	SRR12584155	Apis mellifera	single end	2020	2017	2017	USA	North America	3047098	1010864	33.17	238	0.01
PRJNA661322	SRR12584156	Apis mellifera	single end	2020	2017	2017	USA	North America	2498835	9425	0.38	0	0
PRJNA661322	SRR12584157	Apis mellifera	single end	2020	2017	2017	USA	North America	2550446	9852	0.39	3	0
PRJNA661322	SRR12584158	Apis mellifera	single end	2020	2017	2017	USA	North America	2521642	9840	0.39	2	0
PRJNA661322	SRR12584159	Apis mellifera	single end	2020	2017	2017	USA	North America	3033682	157058	5.18	10	0
PRJNA661322	SRR12584160	Apis mellifera	single end	2020	2017	2017	USA	North America	3098662	162328	5.24	9	0
PRJNA661322	SRR12584161	Apis mellifera	single end	2020	2017	2017	USA	North America	4321889	586553	13.57	108	0
PRJNA661322	SRR12584162	Apis mellifera	single end	2020	2017	2017	USA	North America	3067944	159367	5.19	19	0

PRJNA661322	SRR12584163	Apis mellifera	single end	2020	2017	2017	USA	North America	2498391	26175	1.05	4	0
PRJNA661322	SRR12584164	Apis mellifera	single end	2020	2017	2017	USA	North America	2570721	27461	1.07	0	0
PRJNA661322	SRR12584165	Apis mellifera	single end	2020	2017	2017	USA	North America	2522417	26577	1.05	1	0
PRJNA661322	SRR12584166	Apis mellifera	single end	2020	2017	2017	USA	North America	2489222	349133	14.03	30	0
PRJNA661322	SRR12584167	Apis mellifera	single end	2020	2017	2017	USA	North America	2524029	356625	14.13	35	0
PRJNA661322	SRR12584168	Apis mellifera	single end	2020	2017	2017	USA	North America	2507252	350946	14	47	0
PRJNA661322	SRR12584169	Apis mellifera	single end	2020	2017	2017	USA	North America	3420406	2599	0.08	1	0
PRJNA661322	SRR12584170	Apis mellifera	single end	2020	2017	2017	USA	North America	3336790	2967	0.09	4	0
PRJNA661322	SRR12584171	Apis mellifera	single end	2020	2017	2017	USA	North America	3212443	2868	0.09	2	0
PRJNA661322	SRR12584172	Apis mellifera	single end	2020	2017	2017	USA	North America	4332757	583884	13.48	116	0
PRJNA661322	SRR12584173	Apis mellifera	single end	2020	2017	2017	USA	North America	3333251	398700	11.96	61	0
PRJNA661322	SRR12584174	Apis mellifera	single end	2020	2017	2017	USA	North America	3356879	398791	11.88	64	0
PRJNA661322	SRR12584175	Apis mellifera	single end	2020	2017	2017	USA	North America	3363303	397596	11.82	56	0
PRJNA661322	SRR12584176	Apis mellifera	single end	2020	2017	2017	USA	North America	3466826	425876	12.28	58	0
PRJNA661322	SRR12584177	Apis mellifera	single end	2020	2017	2017	USA	North America	3490718	428304	12.27	55	0
PRJNA661322	SRR12584178	Apis mellifera	single end	2020	2017	2017	USA	North America	3506626	427801	12.2	52	0
PRJNA661322	SRR12584179	Apis mellifera	single end	2020	2017	2017	USA	North America	2238943	287003	12.82	25	0
PRJNA661322	SRR12584180	Apis mellifera	single end	2020	2017	2017	USA	North America	2309454	297767	12.89	20	0
PRJNA661322	SRR12584181	Apis mellifera	single end	2020	2017	2017	USA	North America	2278159	291572	12.8	21	0
PRJNA661322	SRR12584182	Apis mellifera	single end	2020	2017	2017	USA	North America	3234576	326209	10.09	54	0
PRJNA661322	SRR12584183	Apis mellifera	single end	2020	2017	2017	USA	North America	4050903	1933	0.05	0	0
PRJNA661322	SRR12584184	Apis mellifera	single end	2020	2017	2017	USA	North America	3264613	328707	10.07	49	0
PRJNA661322	SRR12584185	Apis mellifera	single end	2020	2017	2017	USA	North America	4493697	205520	4.57	17	0
PRJNA661322	SRR12584186	Apis mellifera	single end	2020	2017	2017	USA	North America	4516345	453832	10.05	235	0.01
PRJNA661322	SRR12584187	Apis mellifera	single end	2020	2017	2017	USA	North America	4403323	438137	9.95	215	0
PRJNA661322	SRR12584188	Apis mellifera	single end	2020	2017	2017	USA	North America	4304688	426905	9.92	233	0.01
PRJNA661322	SRR12584189	Apis mellifera	single end	2020	2017	2017	USA	North America	3549411	585610	16.5	166	0
PRJNA661322	SRR12584190	Apis mellifera	single end	2020	2017	2017	USA	North America	3463634	572594	16.53	161	0
PRJNA661322	SRR12584191	Apis mellifera	single end	2020	2017	2017	USA	North America	3350317	547666	16.35	133	0
PRJNA661322	SRR12584192	Apis mellifera	single end	2020	2017	2017	USA	North America	4907534	806085	16.43	105	0
PRJNA661322	SRR12584193	Apis mellifera	single end	2020	2017	2017	USA	North America	4810960	782228	16.26	114	0
PRJNA661322	SRR12584194	Apis mellifera	single end	2020	2017	2017	USA	North America	4681415	754445	16.12	96	0
PRJNA661322	SRR12584195	Apis mellifera	single end	2020	2017	2017	USA	North America	2888857	438710	15.19	71	0
PRJNA661322	SRR12584196	Apis mellifera	single end	2020	2017	2017	USA	North America	3940760	1522	0.04	0	0
PRJNA661322	SRR12584197	Apis mellifera	single end	2020	2017	2017	USA	North America	3877680	1979	0.05	0	0
PRJNA661322	SRR12584198	Apis mellifera	single end	2020	2017	2017	USA	North America	3758617	2032	0.05	3	0
PRJNA661322	SRR12584199	Apis mellifera	single end	2020	2017	2017	USA	North America	2535429	1558567	61.47	312	0.01
PRJNA661322	SRR12584200	Apis mellifera	single end	2020	2017	2017	USA	North America	2491819	1521293	61.05	305	0.01
PRJNA661322	SRR12584201	Apis mellifera	single end	2020	2017	2017	USA	North America	2414325	1458035	60.39	342	0.01
PRJNA661322	SRR12584202	Apis mellifera	single end	2020	2017	2017	USA	North America	2736703	1167	0.04	15	0
PRJNA661322	SRR12584203	Apis mellifera	single end	2020	2017	2017	USA	North America	2705713	1392	0.05	11	0
PRJNA661322	SRR12584204	Apis mellifera	single end	2020	2017	2017	USA	North America	2593195	1512	0.06	16	0
PRJNA661322	SRR12584205	Apis mellifera	single end	2020	2017	2017	USA	North America	2668434	1026944	38.48	57	0
PRJNA661322	SRR12584206	Apis mellifera	single end	2020	2017	2017	USA	North America	3197306	2834	0.09	2	0
PRJNA661322	SRR12584207	Apis mellifera	single end	2020	2017	2017	USA	North America	2644847	1010641	38.21	82	0
PRJNA661322	SRR12584208	Apis mellifera	single end	2020	2017	2017	USA	North America	2522711	956425	37.91	69	0
PRJNA661322	SRR12584209	Apis mellifera	single end	2020	2017	2017	USA	North America	2880013	1143	0.04	0	0
PRJNA661322	SRR12584210	Apis mellifera	single end	2020	2017	2017	USA	North America	2830970	1380	0.05	0	0
PRJNA661322	SRR12584211	Apis mellifera	single end	2020	2017	2017	USA	North America	2716475	1557	0.06	1	0
PRJNA661322	SRR12584212	Apis mellifera	single end	2020	2017	2017	USA	North America	2834000	2219904	78.33	184	0.01
PRJNA661322	SRR12584213	Apis mellifera	single end	2020	2017	2017	USA	North America	2801620	2183497	77.94	214	0.01
PRJNA661322	SRR12584214	Apis mellifera	single end	2020	2017	2017	USA	North America	2672443	2066825	77.34	208	0.01
PRJNA661322	SRR12584215	Apis mellifera	single end	2020	2017	2017	USA	North America	3538113	43010	1.22	3	0
PRJNA661322	SRR12584216	Apis mellifera	single end	2020	2017	2017	USA	North America	3498106	42530	1.22	3	0
PRJNA661322	SRR12584217	Apis mellifera	single end	2020	2017	2017	USA	North America	3258237	2996	0.09	3	0
PRJNA661322	SRR12584218	Apis mellifera	single end	2020	2017	2017	USA	North America	3351661	40132	1.2	7	0
PRJNA661322	SRR12584219	Apis mellifera	single end	2020	2017	2017	USA	North America	3476039	2285831	65.76	1416	0.04
PRJNA661322	SRR12584220	Apis mellifera	single end	2020	2017	2017	USA	North America	3454456	2256645	65.33	1428	0.04
PRJNA661322	SRR12584221	Apis mellifera	single end	2020	2017	2017	USA	North America	3309398	2137916	64.6	1430	0.04
PRJNA661322	SRR12584222	Apis mellifera	single end	2020	2017	2017	USA	North America	3951563	974935	24.67	332	0.01
PRJNA661322	SRR12584223	Apis mellifera	single end	2020	2017	2017	USA	North America	3876585	950400	24.52	327	0.01
PRJNA661322	SRR12584224	Apis mellifera	single end	2020	2017	2017	USA	North America	3762720	911572	24.23	344	0.01
PRJNA661322	SRR12584225	Apis mellifera	single end	2020	2017	2017	USA	North America	3034087	489932	16.15	111	0
PRJNA661322	SRR12584226	Apis mellifera	single end	2020	2017	2017	USA	North America	2983222	467159	15.66	102	0
PRJNA661322	SRR12584227	Apis mellifera	single end	2020	2017	2017	USA	North America	2902642	462117	15.92	118	0
PRJNA661322	SRR12584228	Apis mellifera	single end	2020	2017	2017	USA	North America	3244125	3303	0.1	2	0
PRJNA661322	SRR12584229	Apis mellifera	single end	2020	2017	2017	USA	North America	2068189	859433	41.55	175	0.01
PRJNA661322	SRR12584230	Apis mellifera	single end	2020	2017	2017	USA	North America	2035287	833359	40.95	166	0.01
PRJNA661322	SRR12584231	Apis mellifera	single end	2020	2017	2017	USA	North America	1981217	806392	40.7	157	0.01
PRJNA661322	SRR12584232	Apis mellifera	single end	2020	2017	2017	USA	North America	4082872	3430	0.08	4	0
PRJNA661322	SRR12584233	Apis mellifera	single end	2020	2017	2017	USA	North America	4018734	4008	0.1	1	0
PRJNA661322	SRR12584234	Apis mellifera	single end	2020	2017	2017	USA	North America	3876821	4219	0.11	0	0
PRJNA661322	SRR12584235	Apis mellifera	single end	2020	2017	2017	USA	North America	4611196	3048062	66.1	1449	0.03
PRJNA661322	SRR12584236	Apis mellifera	single end	2020	2017	2017	USA	North America	4578460	2997791	65.48	1383	0.03
PRJNA661322	SRR12584237	Apis mellifera	single end	2020	2017	2017	USA	North America	4465033	2883290	64.57	1389	0.03
PRJNA661322	SRR12584238	Apis mellifera	single end	2020	2017	2017	USA	North America	3410799	2495	0.07	1	0
PRJNA661322	SRR12584239	Apis mellifera	single end	2020	2017	2017	USA	North America	2873865	227079	7.9	29	0
PRJNA661322	SRR12584240	Apis mellifera	single end	2020	2017	2017	USA	North America	3357913	2919	0.09	0	0
PRJNA661322	SRR12584241	Apis mellifera	single end	2020	2017	2017	USA	North America	3276918	3097	0.09	1	0
PRJNA661322	SRR12584242	Apis mellifera	single end	2020	2017	2017	USA	North America	2809419	1399593	49.82	114	0
PRJNA661322	SRR12584243	Apis mellifera	single end	2020	2017	2017	USA	North America	2809384	1382318	49.2	147	0.01
PRJNA661322	SRR12584244	Apis mellifera	single end	2020	2017	2017	USA	North America	2714767	1322221	48.7	144	0.01
PRJNA661322	SRR12584245	Apis mellifera	single end	2020	2017	2017	USA	North America	2806384	2067186	73.66	448	0.02
PRJNA661322	SRR12584246	Apis mellifera	single end	2020	2017	2017	USA	North America	2777124	2028628	73.05	417	0.02
PRJNA661322	SRR12584247	Apis mellifera	single end	2020	2017	2017	USA	North America	2666456	1935654	72.59	472	0.02
PRJNA661322	SRR12584248	Apis mellifera	single end	2020	2017	2017	USA	North America	4458983	8298	0.19	2	0
PRJNA661322	SRR12584249	Apis mellifera	single end	2020	2017	2017	USA	North America	3314397	651	0.02	0	0
PRJNA661322	SRR12584250	Apis mellifera	single end	2020	2017	2017	USA	North America	3457007	810	0.02	0	0
PRJNA661322	SRR12584251	Apis mellifera	single end	2020	2017	2017	USA	North America	3301471	902	0.03	1	0
PRJNA661322	SRR12584252	Apis mellifera	single end	2020	2017	2017	USA	North America	3707907	852	0.02	0	0
PRJNA661322	SRR12584253	Apis mellifera	single end	2020	2017	2017	USA	North America	3887052	1105	0.03	0	0
PRJNA661322	SRR12584254	Apis mellifera	single end	2020	2017	2017	USA	North America	3694078	1150	0.03	2	0
PRJNA661322	SRR12584255	Apis mellifera	single end	2020	2017	2017	USA	North America	4050910	908	0.02	0	0
PRJNA661322	SRR12584256	Apis mellifera	single end	2020	2017	2017	USA	North America	4254922	1144	0.03	1	0
PRJNA661322	SRR12584257	Apis mellifera	single end	2020	2017	2017	USA	North America	4028858	1314	0.03	0	0
PRJNA661322	SRR12584258	Apis mellifera	single end	2020	2017	2017	USA	North America	3283708	328413	10	51	0
PRJNA661322	SRR12584259	Apis mellifera	single end	2020	2017	2017	USA	North America	2607005	479136	18.38	202	0.01
PRJNA661322	SRR12584260	Apis mellifera	single end	2020	2017	2017	USA	North America	2627738	478134	18.2	183	0.01
PRJNA661322	SRR12584261	Apis mellifera	single end	2020	2017	2017	USA	North America	2646972	478479	18.08	219	0.01
PRJNA661322	SRR12584262	Apis mellifera	single end	2020	2017	2017	USA	North America	1875376	702956	37.48	34	0
PRJNA661322	SRR12584263	Apis mellifera	single end	2020	2017	2017	USA	North America	1919286	718374	37.43	40	0
PRJNA661322	SRR12584264	Apis mellifera	single end	2020	2017	2017	USA	North America	1905552	706247	37.06	60	0
PRJNA661322	SRR12584265	Apis mellifera	single end	2020	2017	2017	USA	North America	1792685	302104	16.85	39	0
PRJNA661322	SRR12584266	Apis mellifera	single end	2020	2017	2017	USA	North America	1820172	308935	16.97	53	0
PRJNA661322	SRR12584267	Apis mellifera	single end	2020	2017	2017	USA	North America	1812210	304354	16.79	59	0
PRJNA661322	SRR12584268	Apis mellifera	single end	2020	2017	2017	USA	North America	4189026	1082947	25.85	106	0

PRJNA661322	SRR12584269	Apis mellifera	single end	2020	2017	2017	USA	North America	2829433	432773	15.3	68	0
PRJNA661322	SRR12584270	Apis mellifera	single end	2020	2017	2017	USA	North America	4144859	1067103	25.75	101	0
PRJNA661322	SRR12584271	Apis mellifera	single end	2020	2017	2017	USA	North America	3972762	1012686	25.49	77	0
PRJNA661322	SRR12584272	Apis mellifera	single end	2020	2017	2017	USA	North America	3784747	932627	24.64	105	0
PRJNA661322	SRR12584273	Apis mellifera	single end	2020	2017	2017	USA	North America	3728920	913771	24.5	84	0
PRJNA661322	SRR12584274	Apis mellifera	single end	2020	2017	2017	USA	North America	3610325	875980	24.26	111	0
PRJNA661322	SRR12584275	Apis mellifera	single end	2020	2017	2017	USA	North America	5346510	553574	10.35	79	0
PRJNA661322	SRR12584276	Apis mellifera	single end	2020	2017	2017	USA	North America	5248796	538675	10.26	91	0
PRJNA661322	SRR12584277	Apis mellifera	single end	2020	2017	2017	USA	North America	5093209	518994	10.19	100	0
PRJNA661322	SRR12584278	Apis mellifera	single end	2020	2017	2017	USA	North America	4737422	218775	4.62	21	0
PRJNA661322	SRR12584279	Apis mellifera	single end	2020	2017	2017	USA	North America	4623619	213355	4.61	21	0
PRJNA661322	SRR12584280	Apis mellifera	single end	2020	2017	2017	USA	North America	2882635	442878	15.36	74	0
PRJNA661322	SRR12584281	Apis mellifera	single end	2020	2017	2017	USA	North America	4399593	8973	0.2	5	0
PRJNA661322	SRR12584282	Apis mellifera	single end	2020	2017	2017	USA	North America	2932975	233461	7.96	11	0
PRJNA661322	SRR12584283	Apis mellifera	single end	2020	2017	2017	USA	North America	4087923	2109	0.05	1	0
PRJNA661322	SRR12584284	Apis mellifera	single end	2020	2017	2017	USA	North America	4107145	2381	0.06	0	0
PRJNA668751	SRR12810599	Apis mellifera	single end	2021	NA	2019	NA	NA	73545785	1	0	0	0
PRJNA668751	SRR12810600	Apis mellifera	single end	2021	NA	2019	NA	NA	69219183	1	0	0	0
PRJNA668751	SRR12810601	Apis mellifera	single end	2021	NA	2019	NA	NA	86089063	3	0	0	0
PRJNA668751	SRR12810602	Apis mellifera	single end	2021	NA	2019	NA	NA	70893558	0	0	1	0
PRJNA668751	SRR12810603	Apis mellifera	single end	2021	NA	2019	NA	NA	68941625	1	0	0	0
PRJNA668751	SRR12810604	Apis mellifera	single end	2021	NA	2019	NA	NA	69103047	2	0	0	0
PRJNA668751	SRR12810605	Apis mellifera	single end	2021	NA	2019	NA	NA	74761874	2281	0	4	0
PRJNA668751	SRR12810606	Apis mellifera	single end	2021	NA	2019	NA	NA	88100392	10	0	2	0
PRJNA668751	SRR12810607	Apis mellifera	single end	2021	NA	2019	NA	NA	71080138	1	0	0	0
PRJNA668751	SRR12810608	Apis mellifera	single end	2021	NA	2019	NA	NA	63404944	1	0	0	0
PRJNA668751	SRR12810609	Apis mellifera	single end	2021	NA	2019	NA	NA	64624747	4	0	0	0
PRJNA668751	SRR12810610	Apis mellifera	single end	2021	NA	2019	NA	NA	69666200	0	0	0	0
PRJNA257666	SRR1538449	Apis mellifera	single end	2015	NA	2013	NA	NA	17417565	10	0	3	0
PRJNA257666	SRR1538718	Apis mellifera	single end	2015	NA	2013	NA	NA	9899631	0	0	0	0
PRJNA257666	SRR1538749	Apis mellifera	single end	2015	NA	2013	NA	NA	38030892	0	0	0	0
PRJNA257666	SRR1538849	Apis mellifera	single end	2015	NA	2013	NA	NA	37086884	0	0	0	0
PRJNA257666	SRR1538850	Apis mellifera	single end	2015	NA	2013	NA	NA	163202336	0	0	0	0
PRJNA257666	SRR1538876	Apis mellifera	single end	2015	NA	2013	NA	NA	32219748	0	0	0	0
PRJNA257666	SRR1538877	Apis mellifera	single end	2015	NA	2013	NA	NA	40486094	0	0	0	0
PRJNA257666	SRR1538878	Apis mellifera	single end	2015	NA	2013	NA	NA	37487408	0	0	0	0
PRJNA257666	SRR1538934	Apis mellifera	single end	2015	NA	2013	NA	NA	22902101	954	0	88	0
PRJNA257666	SRR1538974	Apis mellifera	single end	2015	NA	2013	NA	NA	17060175	3	0	0	0
PRJNA257666	SRR1539264	Apis mellifera	single end	2015	NA	2013	NA	NA	40213259	126	0	1	0
PRJNA257666	SRR1539284	Apis mellifera	single end	2015	NA	2013	NA	NA	33295431	9551	0.03	412	0
PRJNA257666	SRR1539287	Apis mellifera	single end	2015	NA	2013	NA	NA	82208246	2	0	1	0
PRJNA257666	SRR1539288	Apis mellifera	single end	2015	NA	2013	NA	NA	32955026	412	0	12	0
PRJNA260922	SRR1574323	Apis mellifera	single end	2015	NA	2013	NA	NA	113029993	0	0	0	0
PRJNA260922	SRR1574325	Apis mellifera	single end	2015	NA	2013	NA	NA	71120522	0	0	0	0
PRJNA260922	SRR1574326	Apis mellifera	single end	2015	NA	2013	NA	NA	33683147	2	0	0	0
PRJNA260922	SRR1574327	Apis mellifera	single end	2015	NA	2013	NA	NA	31171886	7	0	0	0
PRJNA263933	SRR1613229	Apis mellifera	single end	2015	NA	2013	NA	NA	88942714	252	0	14	0
PRJNA268450	SRR1662285	Apis mellifera	single end	2017	NA	2015	France	Europe	19536465	2956022	15.13	1679	0.01
PRJNA268450	SRR1662286	Apis mellifera	single end	2017	NA	2015	France	Europe	45777936	6312043	13.79	2153	0
PRJNA268450	SRR1662287	Apis mellifera	single end	2017	NA	2015	France	Europe	50057199	8806188	17.59	3766	0.01
PRJNA268450	SRR1662288	Apis mellifera	single end	2017	NA	2015	France	Europe	60209077	10832227	17.99	6136	0.01
PRJNA268450	SRR1662289	Apis mellifera	single end	2017	NA	2015	France	Europe	21484068	4601841	21.42	2385	0.01
PRJNA268450	SRR1662290	Apis mellifera	single end	2017	NA	2015	France	Europe	55722219	16377709	29.39	5563	0.01
PRJNA268450	SRR1662291	Apis mellifera	single end	2017	NA	2015	France	Europe	77733897	18604181	23.93	8452	0.01
PRJNA268450	SRR1662292	Apis mellifera	single end	2017	NA	2015	France	Europe	49184602	15798905	32.12	6046	0.01
PRJNA268450	SRR1662293	Apis mellifera	single end	2017	NA	2015	France	Europe	103455670	9623	0.01	39	0
PRJNA268450	SRR1662294	Apis mellifera	single end	2017	NA	2015	France	Europe	52308319	3693	0.01	0	0
PRJNA268450	SRR1662295	Apis mellifera	single end	2017	NA	2015	France	Europe	78188150	6593281	8.43	2962	0
PRJNA268450	SRR1662296	Apis mellifera	single end	2017	NA	2015	France	Europe	54007997	5868	0.01	23	0
PRJNA268450	SRR1662297	Apis mellifera	single end	2017	NA	2015	France	Europe	27540844	6337	0.02	6	0
PRJNA275154	SRR1798739	Apis mellifera	single end	2015	NA	2013	NA	NA	22298993	0	0	0	0
PRJNA275154	SRR1798740	Apis mellifera	single end	2015	NA	2013	NA	NA	28682552	0	0	0	0
PRJNA275154	SRR1798741	Apis mellifera	single end	2015	NA	2013	NA	NA	27029437	0	0	0	0
PRJNA275154	SRR1798742	Apis mellifera	single end	2015	NA	2013	NA	NA	30430854	0	0	0	0
PRJNA275154	SRR1798743	Apis mellifera	single end	2015	NA	2013	NA	NA	26697130	0	0	0	0
PRJNA275154	SRR1798744	Apis mellifera	single end	2015	NA	2013	NA	NA	30591917	1	0	0	0
PRJNA275154	SRR1798745	Apis mellifera	single end	2015	NA	2013	NA	NA	29588813	0	0	0	0
PRJNA275154	SRR1798746	Apis mellifera	single end	2015	NA	2013	NA	NA	31879217	1	0	0	0
PRJNA275154	SRR1798747	Apis mellifera	single end	2015	NA	2013	NA	NA	31461346	0	0	0	0
PRJNA275154	SRR1798748	Apis mellifera	single end	2015	NA	2013	NA	NA	34711889	0	0	0	0
PRJNA275154	SRR1798749	Apis mellifera	single end	2015	NA	2013	NA	NA	27924845	0	0	0	0
PRJNA275154	SRR1798750	Apis mellifera	single end	2015	NA	2013	NA	NA	39301839	0	0	0	0
PRJNA193691	SRR2954344	Apis mellifera	single end	2015	NA	2013	NA	NA	218188846	0	0	0	0
PRJNA193691	SRR2954345	Apis mellifera	single end	2015	NA	2013	NA	NA	220797918	0	0	1	0
PRJNA193691	SRR2954346	Apis mellifera	single end	2015	NA	2013	NA	NA	215385931	0	0	178	0
PRJNA498667	SRR3476887	Apis mellifera	single end	2018	2012	2012	China	Asia	5945373	100038	1.68	1825	0.03
PRJNA498667	SRR3476890	Apis mellifera	single end	2016	2012	2012	China	Asia	5711376	12451	0.22	1133	0.02
PRJNA322249	SRR3547484	Apis mellifera	single end	2016	NA	2014	NA	NA	90099137	7	0	42	0
PRJNA322249	SRR3547485	Apis mellifera	single end	2016	NA	2014	NA	NA	45252474	0	0	3	0
PRJNA322249	SRR3547486	Apis mellifera	single end	2016	NA	2014	NA	NA	91554041	19	0	33	0
PRJNA322249	SRR3547487	Apis mellifera	single end	2016	NA	2014	NA	NA	79970252	0	0	45	0
PRJNA322249	SRR3547488	Apis mellifera	single end	2016	NA	2014	NA	NA	81902819	0	0	6	0
PRJNA322249	SRR3547489	Apis mellifera	single end	2016	NA	2014	NA	NA	95556493	1	0	65	0
PRJNA322249	SRR3547490	Apis mellifera	single end	2016	NA	2014	NA	NA	73540107	0	0	1	0
PRJNA322249	SRR3547491	Apis mellifera	single end	2016	NA	2014	NA	NA	88830782	0	0	10	0
PRJNA322249	SRR3547492	Apis mellifera	single end	2016	NA	2014	NA	NA	99412010	0	0	7	0
PRJNA322249	SRR3547493	Apis mellifera	single end	2016	NA	2014	NA	NA	56918065	0	0	0	0
PRJNA322249	SRR3547494	Apis mellifera	single end	2016	NA	2014	NA	NA	72283198	0	0	12	0
PRJNA325930	SRR3675069	Apis mellifera	single end	2017	NA	2015	NA	NA	56874766	95	0	0	0
PRJNA325930	SRR3675070	Apis mellifera	single end	2017	NA	2015	NA	NA	52557453	229315	0.44	37	0
PRJNA325930	SRR3675071	Apis mellifera	single end	2017	NA	2015	NA	NA	59985500	109	0	0	0
PRJNA325930	SRR3675072	Apis mellifera	single end	2017	NA	2015	NA	NA	57108520	70	0	0	0
PRJNA325930	SRR3675073	Apis mellifera	single end	2017	NA	2015	NA	NA	54393696	74	0	1	0
PRJNA325930	SRR3675074	Apis mellifera	single end	2017	NA	2015	NA	NA	62617737	84	0	0	0
PRJNA325930	SRR3675076	Apis mellifera	single end	2017	NA	2015	NA	NA	61329088	51	0	0	0
PRJNA325930	SRR3675077	Apis mellifera	single end	2017	NA	2015	NA	NA	58679762	179009	0.31	38	0
PRJNA257666	SRR3708802	Apis mellifera	single end	2015	NA	2013	NA	NA	3843019	0	0	0	0
PRJNA257666	SRR3708803	Apis mellifera	single end	2015	NA	2013	NA	NA	38659603	308	0	6	0
PRJNA257666	SRR3708804	Apis mellifera	single end	2015	NA	2013	NA	NA	40420268	5	0	1	0
PRJNA257666	SRR3714103	Apis mellifera	single end	2015	NA	2013	NA	NA	15185089	14	0	1	0
PRJNA257666	SRR3714104	Apis mellifera	single end	2015	NA	2013	NA	NA	16760987	10	0	2	0
PRJNA327226	SRR3730230	Apis mellifera	single end	2016	2014	2014	Czech	Europe	73242	0	0	0	0
PRJNA339620	SRR4045680	Apis mellifera	single end	2017	NA	2015	USA	North America	23851068	5582	0.02	4	0
PRJNA339620	SRR4045681	Apis mellifera	single end	2017	NA	2015	USA	North America	24200871	4910	0.02	2	0
PRJNA339620	SRR4045682	Apis mellifera	single end	2017	NA	2015	USA	North America	28974715	803	0	2	0
PRJNA339620	SRR4045683	Apis mellifera	single end	2017	NA	2015	USA	North America	26563391	376	0	0	0

PRJNA339620	SRR4045684	Apis mellifera	single end	2017	NA	2015	USA	North America	27816174	43	0	0	0
PRJNA339620	SRR4045685	Apis mellifera	single end	2017	NA	2015	USA	North America	27317012	54	0	0	0
PRJNA339620	SRR4045686	Apis mellifera	single end	2017	NA	2015	USA	North America	29696020	10047	0.03	1	0
PRJNA339620	SRR4045687	Apis mellifera	single end	2017	NA	2015	USA	North America	36043172	9933282	27.56	748	0
PRJNA339620	SRR4045688	Apis mellifera	single end	2017	NA	2015	USA	North America	29979388	10423776	34.77	1563	0.01
PRJNA339620	SRR4045689	Apis mellifera	single end	2017	NA	2015	USA	North America	32404786	6431138	19.85	877	0
PRJNA339620	SRR4045690	Apis mellifera	single end	2017	NA	2015	USA	North America	32374092	38	0	0	0
PRJNA339620	SRR4045691	Apis mellifera	single end	2017	NA	2015	USA	North America	28692431	24	0	0	0
PRJNA339620	SRR4045692	Apis mellifera	single end	2017	NA	2015	USA	North America	30797763	54	0	0	0
PRJNA339620	SRR4045693	Apis mellifera	single end	2017	NA	2015	USA	North America	29308771	32	0	0	0
PRJNA339620	SRR4045694	Apis mellifera	single end	2017	NA	2015	USA	North America	33560237	2160	0.01	2	0
PRJNA339620	SRR4045695	Apis mellifera	single end	2017	NA	2015	USA	North America	38166582	3006426	7.88	417	0
PRJNA339620	SRR4045696	Apis mellifera	single end	2017	NA	2015	USA	North America	42718058	825	0	0	0
PRJNA339620	SRR4045697	Apis mellifera	single end	2017	NA	2015	USA	North America	26033309	27	0	0	0
PRJNA339620	SRR4045698	Apis mellifera	single end	2017	NA	2015	USA	North America	31458328	158	0	0	0
PRJNA339620	SRR4045699	Apis mellifera	single end	2017	NA	2015	USA	North America	30080333	48	0	0	0
PRJNA339620	SRR4045700	Apis mellifera	single end	2017	NA	2015	USA	North America	25532025	16	0	0	0
PRJNA339620	SRR4045701	Apis mellifera	single end	2017	NA	2015	USA	North America	22425405	28	0	0	0
PRJNA339620	SRR4045702	Apis mellifera	single end	2017	NA	2015	USA	North America	23773584	303	0	0	0
PRJNA339620	SRR4045703	Apis mellifera	single end	2017	NA	2015	USA	North America	23043782	714	0	1	0
PRJNA339620	SRR4045704	Apis mellifera	single end	2017	NA	2015	USA	North America	24513706	6819	0.03	0	0
PRJNA339620	SRR4045705	Apis mellifera	single end	2017	NA	2015	USA	North America	10120500	4	0	0	0
PRJNA339620	SRR4045706	Apis mellifera	single end	2017	NA	2015	USA	North America	20361455	171	0	0	0
PRJNA339620	SRR4045707	Apis mellifera	single end	2017	NA	2015	USA	North America	22184991	458	0	1	0
PRJNA339620	SRR4045708	Apis mellifera	single end	2017	NA	2015	USA	North America	22526533	83	0	0	0
PRJNA339620	SRR4045709	Apis mellifera	single end	2017	NA	2015	USA	North America	23928796	22	0	0	0
PRJNA339620	SRR4045710	Apis mellifera	single end	2017	NA	2015	USA	North America	21723254	322	0	0	0
PRJNA339620	SRR4045711	Apis mellifera	single end	2017	NA	2015	USA	North America	21572328	354	0	0	0
PRJNA339620	SRR4045712	Apis mellifera	single end	2017	NA	2015	USA	North America	20921751	3320	0.02	1	0
PRJNA339620	SRR4045713	Apis mellifera	single end	2017	NA	2015	USA	North America	26244192	2299757	8.76	117	0
PRJNA339620	SRR4045714	Apis mellifera	single end	2017	NA	2015	USA	North America	22539652	4704	0.02	2	0
PRJNA339620	SRR4045715	Apis mellifera	single end	2017	NA	2015	USA	North America	23057810	2980305	12.93	413	0
PRJNA339620	SRR4045716	Apis mellifera	single end	2017	NA	2015	USA	North America	23647365	6320637	26.73	238	0
PRJNA339620	SRR4045717	Apis mellifera	single end	2017	NA	2015	USA	North America	23782249	3952	0.02	1	0
PRJNA339620	SRR4045718	Apis mellifera	single end	2017	NA	2015	USA	North America	25567748	1685	0.01	2	0
PRJNA339620	SRR4045719	Apis mellifera	single end	2017	NA	2015	USA	North America	22335525	838	0	1	0
PRJNA339620	SRR4045720	Apis mellifera	single end	2017	NA	2015	USA	North America	33645655	1182	0	1	0
PRJNA339620	SRR4045721	Apis mellifera	single end	2017	NA	2015	USA	North America	28581249	50	0	0	0
PRJNA339620	SRR4045722	Apis mellifera	single end	2017	NA	2015	USA	North America	29987204	6	0	0	0
PRJNA339620	SRR4045723	Apis mellifera	single end	2017	NA	2015	USA	North America	33709363	17	0	0	0
PRJNA339620	SRR4045724	Apis mellifera	single end	2017	NA	2015	USA	North America	28524394	1333	0	3	0
PRJNA339620	SRR4045725	Apis mellifera	single end	2017	NA	2015	USA	North America	34583407	4350	0.01	2	0
PRJNA339620	SRR4045726	Apis mellifera	single end	2017	NA	2015	USA	North America	24738968	3317813	13.41	241	0
PRJNA339620	SRR4045727	Apis mellifera	single end	2017	NA	2015	USA	North America	33450301	4953	0.01	4	0
PRJNA339620	SRR4045728	Apis mellifera	single end	2017	NA	2015	USA	North America	36080932	8061	0.02	2	0
PRJNA339620	SRR4045729	Apis mellifera	single end	2017	NA	2015	USA	North America	28953353	182	0	0	0
PRJNA339620	SRR4045730	Apis mellifera	single end	2017	NA	2015	USA	North America	21878414	310	0	1	0
PRJNA339620	SRR4045731	Apis mellifera	single end	2017	NA	2015	USA	North America	20329527	10	0	0	0
PRJNA339620	SRR4045732	Apis mellifera	single end	2017	NA	2015	USA	North America	21963871	345	0	0	0
PRJNA339620	SRR4045733	Apis mellifera	single end	2017	NA	2015	USA	North America	21628165	462	0	0	0
PRJNA339620	SRR4045734	Apis mellifera	single end	2017	NA	2015	USA	North America	25566735	9	0	0	0
PRJNA339620	SRR4045735	Apis mellifera	single end	2017	NA	2015	USA	North America	23109233	4	0	0	0
PRJNA339620	SRR4045736	Apis mellifera	single end	2017	NA	2015	USA	North America	28728276	873	0	0	0
PRJNA339620	SRR4045737	Apis mellifera	single end	2017	NA	2015	USA	North America	22448394	504	0	0	0
PRJNA339620	SRR4045738	Apis mellifera	single end	2017	NA	2015	USA	North America	26696562	309	0	0	0
PRJNA339620	SRR4045739	Apis mellifera	single end	2017	NA	2015	USA	North America	24092159	61	0	0	0
PRJNA339620	SRR4045740	Apis mellifera	single end	2017	NA	2015	USA	North America	21771668	831	0	1	0
PRJNA339620	SRR4045741	Apis mellifera	single end	2017	NA	2015	USA	North America	20308157	1056	0.01	1	0
PRJNA339620	SRR4045742	Apis mellifera	single end	2017	NA	2015	USA	North America	22471944	438	0	0	0
PRJNA339620	SRR4045743	Apis mellifera	single end	2017	NA	2015	USA	North America	22510273	517	0	0	0
PRJNA339620	SRR4045744	Apis mellifera	single end	2017	NA	2015	USA	North America	23241736	2369321	10.19	130	0
PRJNA339620	SRR4045745	Apis mellifera	single end	2017	NA	2015	USA	North America	20328688	5353137	26.33	235	0
PRJNA339620	SRR4045746	Apis mellifera	single end	2017	NA	2015	USA	North America	21501956	5193271	24.15	333	0
PRJNA339620	SRR4045747	Apis mellifera	single end	2017	NA	2015	USA	North America	20052798	2199	0.01	1	0
PRJNA339620	SRR4045748	Apis mellifera	single end	2017	NA	2015	USA	North America	29450687	5444421	18.49	445	0
PRJNA339620	SRR4045749	Apis mellifera	single end	2017	NA	2015	USA	North America	33249676	5708	0.02	0	0
PRJNA339620	SRR4045750	Apis mellifera	single end	2017	NA	2015	USA	North America	28276111	15	0	0	0
PRJNA339620	SRR4045751	Apis mellifera	single end	2017	NA	2015	USA	North America	26518377	4	0	0	0
PRJNA339620	SRR4045752	Apis mellifera	single end	2017	NA	2015	USA	North America	26773549	1506	0.01	2	0
PRJNA339620	SRR4045753	Apis mellifera	single end	2017	NA	2015	USA	North America	27036524	2699	0.01	3	0
PRJNA339620	SRR4045754	Apis mellifera	single end	2017	NA	2015	USA	North America	25380280	3460252	13.63	590	0
PRJNA339620	SRR4045755	Apis mellifera	single end	2017	NA	2015	USA	North America	27331060	3282	0.01	3	0
PRJNA339620	SRR4045756	Apis mellifera	single end	2017	NA	2015	USA	North America	27778478	677	0	0	0
PRJNA339620	SRR4045757	Apis mellifera	single end	2017	NA	2015	USA	North America	25760719	109	0	0	0
PRJNA339620	SRR4045758	Apis mellifera	single end	2017	NA	2015	USA	North America	27464042	29	0	0	0
PRJNA339620	SRR4045759	Apis mellifera	single end	2017	NA	2015	USA	North America	30536091	80	0	0	0
PRJNA339620	SRR4045760	Apis mellifera	single end	2017	NA	2015	USA	North America	25481566	215	0	0	0
PRJNA339620	SRR4045761	Apis mellifera	single end	2017	NA	2015	USA	North America	29027604	510	0	0	0
PRJNA339620	SRR4045762	Apis mellifera	single end	2017	NA	2015	USA	North America	24759954	12	0	0	0
PRJNA339620	SRR4045763	Apis mellifera	single end	2017	NA	2015	USA	North America	20880191	11	0	0	0
PRJNA339620	SRR4045764	Apis mellifera	single end	2017	NA	2015	USA	North America	27342607	1738002	6.36	145	0
PRJNA339620	SRR4045765	Apis mellifera	single end	2017	NA	2015	USA	North America	24541824	503	0	0	0
PRJNA339620	SRR4045766	Apis mellifera	single end	2017	NA	2015	USA	North America	25435347	304	0	0	0
PRJNA339620	SRR4045767	Apis mellifera	single end	2017	NA	2015	USA	North America	23134547	45	0	0	0
PRJNA339620	SRR4045768	Apis mellifera	single end	2017	NA	2015	USA	North America	22337298	304	0	0	0
PRJNA339620	SRR4045769	Apis mellifera	single end	2017	NA	2015	USA	North America	24305214	506	0	0	0
PRJNA339620	SRR4045770	Apis mellifera	single end	2017	NA	2015	USA	North America	23152192	3614	0.02	3	0
PRJNA339620	SRR4045771	Apis mellifera	single end	2017	NA	2015	USA	North America	26723770	7768562	29.07	1475	0.01
PRJNA339620	SRR4045772	Apis mellifera	single end	2017	NA	2015	USA	North America	27411756	1202	0	2	0
PRJNA339620	SRR4045773	Apis mellifera	single end	2017	NA	2015	USA	North America	22201144	99	0	0	0
PRJNA339620	SRR4045774	Apis mellifera	single end	2017	NA	2015	USA	North America	22521482	95	0	0	0
PRJNA339620	SRR4045775	Apis mellifera	single end	2017	NA	2015	USA	North America	21501481	1287	0.01	0	0
PRJNA339620	SRR4045776	Apis mellifera	single end	2017	NA	2015	USA	North America	31291119	7654	0.02	0	0
PRJNA339620	SRR4045777	Apis mellifera	single end	2017	NA	2015	USA	North America	32606578	10800	0.03	4	0
PRJNA339620	SRR4045778	Apis mellifera	single end	2017	NA	2015	USA	North America	29155184	7388136	25.34	661	0
PRJNA339620	SRR4045779	Apis mellifera	single end	2017	NA	2015	USA	North America	31092940	7246043	23.3	1067	0
PRJNA339620	SRR4045780	Apis mellifera	single end	2017	NA	2015	USA	North America	30581426	16	0	0	0
PRJNA339620	SRR4045781	Apis mellifera	single end	2017	NA	2015	USA	North America	28584267	9	0	0	0
PRJNA339620	SRR4045782	Apis mellifera	single end	2017	NA	2015	USA	North America	27954481	3	0	0	0
PRJNA339620	SRR4045783	Apis mellifera	single end	2017	NA	2015	USA	North America	31412020	6	0	0	0
PRJNA339620	SRR4045784	Apis mellifera	single end	2017	NA	2015	USA	North America	39509297	118	0	0	0
PRJNA339620	SRR4045785	Apis mellifera	single end	2017	NA	2015	USA	North America	44822272	2025	0	0	0
PRJNA339620	SRR4045786	Apis mellifera	single end	2017	NA	2015	USA	North America	39170819	154	0	2	0
PRJNA339620	SRR4045787	Apis mellifera	single end	2017	NA	2015	USA	North America	28499821	99	0	0	0
PRJNA339620	SRR4045788	Apis mellifera	single end	2017	NA	2015	USA	North America	27440025	13	0	0	0
PRJNA339620	SRR4045789	Apis mellifera	single end	2017	NA	2015	USA	North America	29242742	62	0	0	0

PRJNA339620	SRR4045790	Apis mellifera	single end	2017	NA	2015	USA	North America	26270064	87	0	0	0
PRJNA339620	SRR4045791	Apis mellifera	single end	2017	NA	2015	USA	North America	26227162	0	0	0	0
PRJNA339620	SRR4045792	Apis mellifera	single end	2017	NA	2015	USA	North America	27410545	342	0	0	0
PRJNA339620	SRR4045793	Apis mellifera	single end	2017	NA	2015	USA	North America	25910325	415	0	0	0
PRJNA339620	SRR4045794	Apis mellifera	single end	2017	NA	2015	USA	North America	25389005	345	0	1	0
PRJNA339620	SRR4045795	Apis mellifera	single end	2017	NA	2015	USA	North America	26082779	13	0	0	0
PRJNA339620	SRR4045796	Apis mellifera	single end	2017	NA	2015	USA	North America	23057857	193	0	0	0
PRJNA339620	SRR4045797	Apis mellifera	single end	2017	NA	2015	USA	North America	22564446	484	0	0	0
PRJNA339620	SRR4045798	Apis mellifera	single end	2017	NA	2015	USA	North America	23314366	334	0	0	0
PRJNA339620	SRR4045799	Apis mellifera	single end	2017	NA	2015	USA	North America	22177390	59	0	0	0
PRJNA339620	SRR4045800	Apis mellifera	single end	2017	NA	2015	USA	North America	23373880	338	0	0	0
PRJNA339620	SRR4045801	Apis mellifera	single end	2017	NA	2015	USA	North America	22902366	337	0	0	0
PRJNA339620	SRR4045802	Apis mellifera	single end	2017	NA	2015	USA	North America	22585146	970	0	0	0
PRJNA339620	SRR4045803	Apis mellifera	single end	2017	NA	2015	USA	North America	22871493	3452	0.02	1	0
PRJNA339620	SRR4045804	Apis mellifera	single end	2017	NA	2015	USA	North America	22971274	1149871	5.01	221	0
PRJNA339620	SRR4045805	Apis mellifera	single end	2017	NA	2015	USA	North America	22034114	4837	0.02	0	0
PRJNA339620	SRR4045806	Apis mellifera	single end	2017	NA	2015	USA	North America	21392104	2101	0.01	0	0
PRJNA339620	SRR4045807	Apis mellifera	single end	2017	NA	2015	USA	North America	24012070	1002	0	1	0
PRJNA339620	SRR4045808	Apis mellifera	single end	2017	NA	2015	USA	North America	21899167	1125	0.01	0	0
PRJNA339620	SRR4045809	Apis mellifera	single end	2017	NA	2015	USA	North America	22302943	3695348	16.57	564	0
PRJNA339620	SRR4045810	Apis mellifera	single end	2017	NA	2015	USA	North America	28194072	1148	0	1	0
PRJNA339620	SRR4045811	Apis mellifera	single end	2017	NA	2015	USA	North America	26795839	46	0	0	0
PRJNA339620	SRR4045812	Apis mellifera	single end	2017	NA	2015	USA	North America	26813133	7	0	0	0
PRJNA339620	SRR4045813	Apis mellifera	single end	2017	NA	2015	USA	North America	30057746	8	0	0	0
PRJNA339620	SRR4045814	Apis mellifera	single end	2017	NA	2015	USA	North America	30793267	1676	0.01	1	0
PRJNA339620	SRR4045815	Apis mellifera	single end	2017	NA	2015	USA	North America	34129168	3018	0.01	3	0
PRJNA339620	SRR4045816	Apis mellifera	single end	2017	NA	2015	USA	North America	28817133	3295	0.01	2	0
PRJNA339620	SRR4045817	Apis mellifera	single end	2017	NA	2015	USA	North America	36630518	11091	0.03	7	0
PRJNA339620	SRR4045818	Apis mellifera	single end	2017	NA	2015	USA	North America	35143427	6411896	18.24	884	0
PRJNA339620	SRR4045819	Apis mellifera	single end	2017	NA	2015	USA	North America	31223453	56	0	0	0
PRJNA339620	SRR4045820	Apis mellifera	single end	2017	NA	2015	USA	North America	22792720	3567362	15.65	81	0
PRJNA339620	SRR4045821	Apis mellifera	single end	2017	NA	2015	USA	North America	21125678	50	0	0	0
PRJNA339620	SRR4045822	Apis mellifera	single end	2017	NA	2015	USA	North America	22380924	222	0	0	0
PRJNA339620	SRR4045823	Apis mellifera	single end	2017	NA	2015	USA	North America	20966792	560	0	1	0
PRJNA339620	SRR4045824	Apis mellifera	single end	2017	NA	2015	USA	North America	24485910	272	0	0	0
PRJNA339620	SRR4045825	Apis mellifera	single end	2017	NA	2015	USA	North America	22692026	21	0	0	0
PRJNA339620	SRR4045826	Apis mellifera	single end	2017	NA	2015	USA	North America	21012665	375	0	1	0
PRJNA339620	SRR4045827	Apis mellifera	single end	2017	NA	2015	USA	North America	23437807	393	0	0	0
PRJNA339620	SRR4045828	Apis mellifera	single end	2017	NA	2015	USA	North America	22529015	274	0	0	0
PRJNA339620	SRR4045829	Apis mellifera	single end	2017	NA	2015	USA	North America	20458054	1	0	0	0
PRJNA339620	SRR4045830	Apis mellifera	single end	2017	NA	2015	USA	North America	23719911	3945688	16.63	228	0
PRJNA339620	SRR4045831	Apis mellifera	single end	2017	NA	2015	USA	North America	23014376	5746	0.02	0	0
PRJNA339620	SRR4045832	Apis mellifera	single end	2017	NA	2015	USA	North America	24236724	549	0	1	0
PRJNA339620	SRR4045833	Apis mellifera	single end	2017	NA	2015	USA	North America	23899689	1039	0	1	0
PRJNA339620	SRR4045834	Apis mellifera	single end	2017	NA	2015	USA	North America	22859426	2771	0.01	1	0
PRJNA339620	SRR4045835	Apis mellifera	single end	2017	NA	2015	USA	North America	26595749	40373	0.15	4	0
PRJNA339620	SRR4045836	Apis mellifera	single end	2017	NA	2015	USA	North America	10608814	2060	0.02	0	0
PRJNA339620	SRR4045837	Apis mellifera	single end	2017	NA	2015	USA	North America	22628346	3017265	13.33	131	0
PRJNA339620	SRR4045838	Apis mellifera	single end	2017	NA	2015	USA	North America	26406366	5723405	21.67	481	0
PRJNA339620	SRR4045839	Apis mellifera	single end	2017	NA	2015	USA	North America	30036919	7303205	24.31	733	0
PRJNA339620	SRR4045840	Apis mellifera	single end	2017	NA	2015	USA	North America	29912453	65	0	0	0
PRJNA339620	SRR4045841	Apis mellifera	single end	2017	NA	2015	USA	North America	33452787	5	0	0	0
PRJNA339620	SRR4045842	Apis mellifera	single end	2017	NA	2015	USA	North America	16463300	757	0	1	0
PRJNA339620	SRR4045843	Apis mellifera	single end	2017	NA	2015	USA	North America	30189296	3194	0.01	2	0
PRJNA339620	SRR4045844	Apis mellifera	single end	2017	NA	2015	USA	North America	26216431	7477	0.03	5	0
PRJNA339620	SRR4045845	Apis mellifera	single end	2017	NA	2015	USA	North America	29282020	4118	0.01	2	0
PRJNA339620	SRR4045846	Apis mellifera	single end	2017	NA	2015	USA	North America	28228172	896	0	0	0
PRJNA339620	SRR4045847	Apis mellifera	single end	2017	NA	2015	USA	North America	26988204	131	0	0	0
PRJNA339620	SRR4045848	Apis mellifera	single end	2017	NA	2015	USA	North America	29062242	25	0	0	0
PRJNA339620	SRR4045849	Apis mellifera	single end	2017	NA	2015	USA	North America	30614879	1003	0	0	0
PRJNA339620	SRR4045850	Apis mellifera	single end	2017	NA	2015	USA	North America	25320385	2034778	8.04	164	0
PRJNA339620	SRR4045851	Apis mellifera	single end	2017	NA	2015	USA	North America	21930832	509	0	0	0
PRJNA339620	SRR4045852	Apis mellifera	single end	2017	NA	2015	USA	North America	23805938	39	0	0	0
PRJNA339620	SRR4045853	Apis mellifera	single end	2017	NA	2015	USA	North America	21984880	12	0	0	0
PRJNA339620	SRR4045854	Apis mellifera	single end	2017	NA	2015	USA	North America	24910807	1092	0	2	0
PRJNA339620	SRR4045855	Apis mellifera	single end	2017	NA	2015	USA	North America	23081864	4728158	20.48	338	0
PRJNA339620	SRR4045856	Apis mellifera	single end	2017	NA	2015	USA	North America	26249109	362	0	0	0
PRJNA339620	SRR4045857	Apis mellifera	single end	2017	NA	2015	USA	North America	24024322	95	0	0	0
PRJNA339620	SRR4045858	Apis mellifera	single end	2017	NA	2015	USA	North America	25678839	331	0	0	0
PRJNA339620	SRR4045859	Apis mellifera	single end	2017	NA	2015	USA	North America	23881467	4453648	18.65	91	0
PRJNA343198	SRR4250143	Apis mellifera	single end	2017	NA	2015	USA	North America	30571929	78	0	2246398	7.35
PRJNA343198	SRR4250144	Apis mellifera	single end	2017	NA	2015	USA	North America	34124835	19	0	9026	0.03
PRJNA343198	SRR4250145	Apis mellifera	single end	2017	NA	2015	USA	North America	34257986	58	0	3642730	10.63
PRJNA343198	SRR4250146	Apis mellifera	single end	2017	NA	2015	USA	North America	33010279	1876	0.01	812	0
PRJNA343198	SRR4250147	Apis mellifera	single end	2017	NA	2015	USA	North America	34018660	1337	0	427	0
PRJNA343198	SRR4250148	Apis mellifera	single end	2017	NA	2015	USA	North America	32658223	166	0	611	0
PRJNA343198	SRR4250149	Apis mellifera	single end	2017	NA	2015	USA	North America	33765225	968	0	442	0
PRJNA343198	SRR4250150	Apis mellifera	single end	2017	NA	2015	USA	North America	31241970	25	0	328	0
PRJNA343198	SRR4250151	Apis mellifera	single end	2017	NA	2015	USA	North America	35247249	8	0	1291	0
PRJNA343198	SRR4250152	Apis mellifera	single end	2017	NA	2015	USA	North America	49255298	26	0	1720663	3.49
PRJNA343198	SRR4250153	Apis mellifera	single end	2017	NA	2015	USA	North America	43815220	28	0	314	0
PRJNA343198	SRR4250154	Apis mellifera	single end	2017	NA	2015	USA	North America	40088056	19	0	732	0
PRJNA343198	SRR4250155	Apis mellifera	single end	2017	NA	2015	USA	North America	33989685	1	0	596	0
PRJNA343198	SRR4250156	Apis mellifera	single end	2017	NA	2015	USA	North America	32299680	98	0	490	0
PRJNA343198	SRR4250157	Apis mellifera	single end	2017	NA	2015	USA	North America	33513685	14	0	668	0
PRJNA343198	SRR4250158	Apis mellifera	single end	2017	NA	2015	USA	North America	31337198	12	0	1178604	3.76
PRJNA343198	SRR4250159	Apis mellifera	single end	2017	NA	2015	USA	North America	35441644	0	0	858	0
PRJNA343198	SRR4250160	Apis mellifera	single end	2017	NA	2015	USA	North America	34320201	0	0	1019	0
PRJNA343198	SRR4250161	Apis mellifera	single end	2017	NA	2015	USA	North America	33093668	23	0	1025484	3.1
PRJNA343198	SRR4250162	Apis mellifera	single end	2017	NA	2015	USA	North America	32343273	1	0	710	0
PRJNA343198	SRR4250163	Apis mellifera	single end	2017	NA	2015	USA	North America	28489152	1	0	2132	0.01
PRJNA343198	SRR4250164	Apis mellifera	single end	2017	NA	2015	USA	North America	33286424	20	0	1567996	4.71
PRJNA343198	SRR4250165	Apis mellifera	single end	2017	NA	2015	USA	North America	32155302	0	0	1494	0
PRJNA343198	SRR4250166	Apis mellifera	single end	2017	NA	2015	USA	North America	31472915	22	0	2173636	6.91
PRJNA343198	SRR4250167	Apis mellifera	single end	2017	NA	2015	USA	North America	32519337	8	0	2476	0.01
PRJNA343198	SRR4250168	Apis mellifera	single end	2017	NA	2015	USA	North America	39067226	0	0	3364	0.01
PRJNA343198	SRR4250169	Apis mellifera	single end	2017	NA	2015	USA	North America	30507741	11	0	1261995	4.14
PRJNA343198	SRR4250170	Apis mellifera	single end	2017	NA	2015	USA	North America	29900129	16	0	1247276	4.17
PRJNA343198	SRR4250171	Apis mellifera	single end	2017	NA	2015	USA	North America	34522324	29	0	2119422	6.14
PRJNA343198	SRR4250172	Apis mellifera	single end	2017	NA	2015	USA	North America	32247367	37	0	2363288	7.33
PRJNA343198	SRR4250173	Apis mellifera	single end	2017	NA	2015	USA	North America	35707938	14	0	1508940	4.23
PRJNA343198	SRR4250174	Apis mellifera	single end	2017	NA	2015	USA	North America	35650162	2	0	1414	0
PRJNA343198	SRR4250175	Apis mellifera	single end	2017	NA	2015	USA	North America	34883913	24	0	2153185	6.17
PRJNA343198	SRR4250176	Apis mellifera	single end	2017	NA	2015	USA	North America	35055048	28	0	1607252	4.58
PRJNA343198	SRR4250177	Apis mellifera	single end	2017	NA	2015	USA	North America	31768288	70	0	3908011	12.3
PRJNA343198	SRR4250178	Apis mellifera	single end	2017	NA	2015	USA	North America	34468529	80	0	4466699	12.96

PRJNA343067	SRR4292727	Apis mellifera	single end	2017	2014	2014	Switzerland	Europe	31089426	0	0	493	0
PRJNA343067	SRR4292728	Apis mellifera	single end	2017	2014	2014	Switzerland	Europe	36702743	695	0	8848913	24.11
PRJNA343067	SRR4292729	Apis mellifera	single end	2017	2014	2014	Switzerland	Europe	31634609	4	0	812	0
PRJNA343067	SRR4292730	Apis mellifera	single end	2017	2014	2014	Switzerland	Europe	31436644	3	0	1323	0
PRJNA343067	SRR4292731	Apis mellifera	single end	2017	2014	2014	Switzerland	Europe	33943085	1	0	549	0
PRJNA343067	SRR4292732	Apis mellifera	single end	2017	2014	2014	Switzerland	Europe	38764197	47	0	168734	0.44
PRJNA343067	SRR4292733	Apis mellifera	single end	2017	2014	2014	Switzerland	Europe	33504650	4	0	1690	0.01
PRJNA343067	SRR4292734	Apis mellifera	single end	2017	2014	2014	Switzerland	Europe	38163413	1	0	934	0
PRJNA343067	SRR4292735	Apis mellifera	single end	2017	2014	2014	Switzerland	Europe	31107356	1	0	309	0
PRJNA343067	SRR4292736	Apis mellifera	single end	2017	2014	2014	Switzerland	Europe	34795526	1	0	444	0
PRJNA343067	SRR4292737	Apis mellifera	single end	2017	2014	2014	Switzerland	Europe	36067838	1	0	395	0
PRJNA343067	SRR4292738	Apis mellifera	single end	2017	2014	2014	Switzerland	Europe	35894741	3	0	942	0
PRJNA343067	SRR4292739	Apis mellifera	single end	2017	2014	2014	Switzerland	Europe	37887662	2	0	562	0
PRJNA343067	SRR4292740	Apis mellifera	single end	2017	2014	2014	Switzerland	Europe	39703796	1	0	842	0
PRJNA343067	SRR4292741	Apis mellifera	single end	2017	2014	2014	Switzerland	Europe	38352716	1	0	440	0
PRJNA345404	SRR4343845	Apis mellifera	single end	2017	NA	2015	NA	NA	8432479	1	0	392	0
PRJNA345404	SRR4343846	Apis mellifera	single end	2017	NA	2015	NA	NA	7690777	0	0	111	0
PRJNA352995	SRR5008279	Apis mellifera	single end	2016	2015	2015	Czech	Europe	74933	0	0	0	0
PRJNA352995	SRR5008280	Apis mellifera	single end	2016	2015	2015	Czech	Europe	84691	0	0	0	0
PRJNA352995	SRR5008281	Apis mellifera	single end	2016	2015	2015	Czech	Europe	74589	0	0	0	0
PRJNA352995	SRR5008282	Apis mellifera	single end	2016	2015	2015	Czech	Europe	80279	0	0	0	0
PRJNA352995	SRR5008283	Apis mellifera	single end	2016	2015	2015	Czech	Europe	74280	0	0	0	0
PRJNA352995	SRR5008284	Apis mellifera	single end	2016	2015	2015	Czech	Europe	75094	0	0	0	0
PRJNA352995	SRR5008285	Apis mellifera	single end	2016	2015	2015	Czech	Europe	71685	0	0	0	0
PRJNA352995	SRR5008286	Apis mellifera	single end	2016	2015	2015	Czech	Europe	79445	0	0	0	0
PRJNA352995	SRR5008287	Apis mellifera	single end	2016	2015	2015	Czech	Europe	43402	0	0	0	0
PRJNA352995	SRR5008288	Apis mellifera	single end	2016	2015	2015	Czech	Europe	50198	0	0	0	0
PRJNA352995	SRR5008289	Apis mellifera	single end	2016	2015	2015	Czech	Europe	50119	0	0	0	0
PRJNA352995	SRR5008290	Apis mellifera	single end	2016	2015	2015	Czech	Europe	84012	0	0	0	0
PRJNA352995	SRR5008291	Apis mellifera	single end	2016	2015	2015	Czech	Europe	79607	0	0	0	0
PRJNA352995	SRR5008292	Apis mellifera	single end	2016	2015	2015	Czech	Europe	99630	0	0	0	0
PRJNA352995	SRR5008293	Apis mellifera	single end	2016	2015	2015	Czech	Europe	76032	0	0	0	0
PRJNA352995	SRR5008294	Apis mellifera	single end	2016	2015	2015	Czech	Europe	66403	0	0	0	0
PRJNA352995	SRR5008295	Apis mellifera	single end	2016	2015	2015	Czech	Europe	66525	0	0	0	0
PRJNA352995	SRR5008296	Apis mellifera	single end	2016	2014	2014	Czech	Europe	70984	0	0	0	0
PRJNA352995	SRR5008297	Apis mellifera	single end	2016	2015	2015	Czech	Europe	82509	0	0	0	0
PRJNA352995	SRR5008298	Apis mellifera	single end	2016	2015	2015	Czech	Europe	68516	0	0	0	0
PRJNA352995	SRR5008299	Apis mellifera	single end	2016	2015	2015	Czech	Europe	78363	0	0	0	0
PRJNA352995	SRR5008300	Apis mellifera	single end	2016	2015	2015	Czech	Europe	83722	0	0	0	0
PRJNA352995	SRR5008301	Apis mellifera	single end	2016	2015	2015	Czech	Europe	80391	0	0	0	0
PRJNA352995	SRR5008302	Apis mellifera	single end	2016	2015	2015	Czech	Europe	81750	0	0	0	0
PRJNA352995	SRR5008303	Apis mellifera	single end	2016	2015	2015	Czech	Europe	67562	0	0	0	0
PRJNA352995	SRR5008304	Apis mellifera	single end	2016	2015	2015	Czech	Europe	78084	0	0	0	0
PRJNA352995	SRR5008305	Apis mellifera	single end	2016	2015	2015	Czech	Europe	71795	0	0	0	0
PRJNA352995	SRR5008306	Apis mellifera	single end	2016	2015	2015	Czech	Europe	14509	0	0	0	0
PRJNA352995	SRR5008307	Apis mellifera	single end	2016	2015	2015	Czech	Europe	79226	0	0	0	0
PRJNA352995	SRR5008308	Apis mellifera	single end	2016	2015	2015	Czech	Europe	89096	0	0	0	0
PRJNA352995	SRR5008309	Apis mellifera	single end	2016	2015	2015	Czech	Europe	62163	0	0	0	0
PRJNA352995	SRR5008310	Apis mellifera	single end	2016	2015	2015	Czech	Europe	75522	0	0	0	0
PRJNA352995	SRR5008311	Apis mellifera	single end	2016	2015	2015	Czech	Europe	75643	0	0	0	0
PRJNA352995	SRR5008312	Apis mellifera	single end	2016	2015	2015	Czech	Europe	63058	0	0	0	0
PRJNA352995	SRR5008313	Apis mellifera	single end	2016	2015	2015	Czech	Europe	56124	0	0	0	0
PRJNA352995	SRR5008314	Apis mellifera	single end	2016	2015	2015	Czech	Europe	57145	0	0	0	0
PRJNA352995	SRR5008315	Apis mellifera	single end	2016	2015	2015	Czech	Europe	90371	0	0	0	0
PRJNA352995	SRR5008316	Apis mellifera	single end	2016	2014	2014	Czech	Europe	79720	0	0	0	0
PRJNA352995	SRR5008317	Apis mellifera	single end	2016	2015	2015	Czech	Europe	80566	0	0	0	0
PRJNA353243	SRR5012743	Apis mellifera	single end	2016	2014	2014	Czech	Europe	12173	0	0	0	0
PRJNA353243	SRR5012744	Apis mellifera	single end	2016	2014	2014	Czech	Europe	42287	0	0	0	0
PRJNA353243	SRR5012745	Apis mellifera	single end	2016	2014	2014	Czech	Europe	50244	0	0	0	0
PRJNA353243	SRR5012746	Apis mellifera	single end	2016	2014	2014	Czech	Europe	74423	0	0	0	0
PRJNA353243	SRR5012747	Apis mellifera	single end	2016	2014	2014	Czech	Europe	70984	0	0	0	0
PRJNA353243	SRR5012748	Apis mellifera	single end	2016	2014	2014	Czech	Europe	26067	0	0	0	0
PRJNA353243	SRR5012749	Apis mellifera	single end	2016	2014	2014	Czech	Europe	47993	0	0	0	0
PRJNA353243	SRR5012750	Apis mellifera	single end	2016	2014	2014	Czech	Europe	30220	0	0	0	0
PRJNA353243	SRR5012752	Apis mellifera	single end	2016	2014	2014	Czech	Europe	40498	0	0	0	0
PRJNA353243	SRR5012753	Apis mellifera	single end	2016	2014	2014	Czech	Europe	46638	0	0	0	0
PRJNA353243	SRR5012754	Apis mellifera	single end	2016	2014	2014	Czech	Europe	74325	0	0	0	0
PRJNA353243	SRR5012755	Apis mellifera	single end	2016	2014	2014	Czech	Europe	41251	0	0	0	0
PRJNA353243	SRR5012756	Apis mellifera	single end	2016	2014	2014	Czech	Europe	79720	0	0	0	0
PRJNA353243	SRR5012757	Apis mellifera	single end	2016	2014	2014	Czech	Europe	72063	0	0	0	0
PRJNA353243	SRR5012758	Apis mellifera	single end	2016	2014	2014	Czech	Europe	56267	0	0	0	0
PRJNA353243	SRR5012759	Apis mellifera	single end	2016	2014	2014	Czech	Europe	43784	0	0	0	0
PRJNA353243	SRR5012760	Apis mellifera	single end	2016	2014	2014	Czech	Europe	82909	0	0	0	0
PRJNA353243	SRR5012761	Apis mellifera	single end	2016	2014	2014	Czech	Europe	20931	0	0	0	0
PRJNA353243	SRR5012762	Apis mellifera	single end	2016	2014	2014	Czech	Europe	69890	0	0	0	0
PRJNA353243	SRR5012763	Apis mellifera	single end	2016	2014	2014	Czech	Europe	49920	0	0	0	0
PRJNA353243	SRR5012764	Apis mellifera	single end	2016	2014	2014	Czech	Europe	51390	0	0	0	0
PRJNA353243	SRR5012765	Apis mellifera	single end	2016	2014	2014	Czech	Europe	46926	0	0	0	0
PRJNA353243	SRR5012766	Apis mellifera	single end	2016	2014	2014	Czech	Europe	50153	0	0	0	0
PRJNA353243	SRR5012767	Apis mellifera	single end	2016	2014	2014	Czech	Europe	14389	0	0	0	0
PRJNA374654	SRR5252792	Apis mellifera	single end	2017	NA	2015	USA	North America	27338758	22954928	83.96	71396	0.26
PRJNA374654	SRR5252793	Apis mellifera	single end	2017	NA	2015	USA	North America	18225222	137999	0.76	418	0
PRJNA374654	SRR5252794	Apis mellifera	single end	2017	NA	2015	USA	North America	24683141	90953	0.37	316	0
PRJNA374654	SRR5252795	Apis mellifera	single end	2017	NA	2015	USA	North America	19927120	64181	0.32	211	0
PRJNA374654	SRR5252796	Apis mellifera	single end	2017	NA	2015	USA	North America	16706666	73885	0.44	207	0
PRJNA374654	SRR5252797	Apis mellifera	single end	2017	NA	2015	USA	North America	26007148	16497773	63.44	99275	0.38
PRJNA374654	SRR5252798	Apis mellifera	single end	2017	NA	2015	USA	North America	27712817	19917453	71.87	100605	0.36
PRJNA374654	SRR5252799	Apis mellifera	single end	2017	NA	2015	USA	North America	10804739	305630	2.83	1964	0.02
PRJNA374654	SRR5252800	Apis mellifera	single end	2017	NA	2015	USA	North America	15344334	9899892	64.52	56666	0.37
PRJNA374654	SRR5252801	Apis mellifera	single end	2017	NA	2015	USA	North America	27732490	87694	0.32	516	0
PRJNA374654	SRR5252802	Apis mellifera	single end	2017	NA	2015	USA	North America	21260599	17849070	83.95	177928	0.84
PRJNA374654	SRR5252803	Apis mellifera	single end	2017	NA	2015	USA	North America	33012513	2935006	8.89	28222	0.09
PRJNA374654	SRR5252804	Apis mellifera	single end	2017	NA	2015	USA	North America	30313176	25874081	85.36	192104	0.63
PRJNA374654	SRR5252805	Apis mellifera	single end	2017	NA	2015	USA	North America	25472838	200384	0.79	1514	0.01
PRJNA374654	SRR5252806	Apis mellifera	single end	2017	NA	2015	USA	North America	24363190	18361721	75.37	124289	0.51
PRJNA172020	SRR547522	Apis mellifera	single end	2015	NA	2013	NA	NA	21057716	361824	1.72	176	0
PRJNA172020	SRR547523	Apis mellifera	single end	2015	NA	2013	NA	NA	30295220	237198	0.78	174	0
PRJNA175822	SRR575799	Apis mellifera	single end	2013	NA	2011	NA	NA	1762567	0	0	8	0
PRJNA175822	SRR575809	Apis mellifera	single end	2013	NA	2011	NA	NA	4000000	1	0	13	0
PRJNA172020	SRR634130	Apis mellifera	single end	2015	NA	2013	NA	NA	29999783	524026	1.75	693	0
PRJNA450078	SRR7000577	Apis mellifera	single end	2018	NA	2016	NA	NA	20544780	0	0	0	0
PRJNA450078	SRR7000578	Apis mellifera	single end	2018	NA	2016	NA	NA	21119844	0	0	0	0
PRJNA450078	SRR7000579	Apis mellifera	single end	2018	NA	2016	NA	NA	17619211	0	0	0	0
PRJNA450078	SRR7000580	Apis mellifera	single end	2018	NA	2016	NA	NA	17667933	0	0	0	0
PRJNA450078	SRR7000581	Apis mellifera	single end	2018	NA	2016	NA	NA	22374675	0	0	0	0
PRJNA450078	SRR7000582	Apis mellifera	single end	2018	NA	2016	NA	NA	20367923	1	0	0	0

PRJNA450078	SRR7000583	Apis mellifera	single end	2018	NA	2016	NA	NA	23883172	1	0	0	0
PRJNA450078	SRR7000584	Apis mellifera	single end	2018	NA	2016	NA	NA	20097670	0	0	0	0
PRJNA450078	SRR7000585	Apis mellifera	single end	2018	NA	2016	NA	NA	22815088	3	0	0	0
PRJNA450078	SRR7000586	Apis mellifera	single end	2018	NA	2016	NA	NA	20070166	0	0	0	0
PRJNA450078	SRR7000587	Apis mellifera	single end	2018	NA	2016	NA	NA	19282246	1	0	0	0
PRJNA450078	SRR7000588	Apis mellifera	single end	2018	NA	2016	NA	NA	15099563	2	0	52	0
PRJNA450078	SRR7000589	Apis mellifera	single end	2018	NA	2016	NA	NA	26668101	0	0	4	0
PRJNA450078	SRR7000590	Apis mellifera	single end	2018	NA	2016	NA	NA	26890398	0	0	0	0
PRJNA450078	SRR7000591	Apis mellifera	single end	2018	NA	2016	NA	NA	21858195	0	0	0	0
PRJNA450078	SRR7000592	Apis mellifera	single end	2018	NA	2016	NA	NA	27376151	0	0	0	0
PRJNA450078	SRR7000593	Apis mellifera	single end	2018	NA	2016	NA	NA	23495833	0	0	2	0
PRJNA450078	SRR7000594	Apis mellifera	single end	2018	NA	2016	NA	NA	19259492	0	0	0	0
PRJNA450078	SRR7000595	Apis mellifera	single end	2018	NA	2016	NA	NA	14052100	0	0	0	0
PRJNA450078	SRR7000596	Apis mellifera	single end	2018	NA	2016	NA	NA	24524926	0	0	0	0
PRJNA450078	SRR7000597	Apis mellifera	single end	2018	NA	2016	NA	NA	24143760	1	0	0	0
PRJNA450078	SRR7000598	Apis mellifera	single end	2018	NA	2016	NA	NA	25138170	0	0	0	0
PRJNA450078	SRR7000599	Apis mellifera	single end	2018	NA	2016	NA	NA	25047818	0	0	0	0
PRJNA450078	SRR7000600	Apis mellifera	single end	2018	NA	2016	NA	NA	21930459	0	0	0	0
PRJNA450078	SRR7000601	Apis mellifera	single end	2018	NA	2016	NA	NA	31440262	0	0	0	0
PRJNA450078	SRR7000602	Apis mellifera	single end	2018	NA	2016	NA	NA	28266862	0	0	0	0
PRJNA450078	SRR7000603	Apis mellifera	single end	2018	NA	2016	NA	NA	27817153	13	0	0	0
PRJNA450078	SRR7000604	Apis mellifera	single end	2018	NA	2016	NA	NA	25585652	0	0	0	0
PRJNA450078	SRR7000605	Apis mellifera	single end	2018	NA	2016	NA	NA	26926315	0	0	0	0
PRJNA450078	SRR7000606	Apis mellifera	single end	2018	NA	2016	NA	NA	25771585	32	0	0	0
PRJNA450078	SRR7000607	Apis mellifera	single end	2018	NA	2016	NA	NA	19322791	0	0	0	0
PRJNA450078	SRR7000608	Apis mellifera	single end	2018	NA	2016	NA	NA	20124412	0	0	0	0
PRJNA450078	SRR7000609	Apis mellifera	single end	2018	NA	2016	NA	NA	28607081	0	0	2	0
PRJNA450078	SRR7000610	Apis mellifera	single end	2018	NA	2016	NA	NA	23768862	0	0	0	0
PRJNA450078	SRR7000611	Apis mellifera	single end	2018	NA	2016	NA	NA	20568587	0	0	0	0
PRJNA450078	SRR7000612	Apis mellifera	single end	2018	NA	2016	NA	NA	19669264	0	0	0	0
PRJNA450078	SRR7000613	Apis mellifera	single end	2018	NA	2016	NA	NA	21229540	0	0	0	0
PRJNA450078	SRR7000614	Apis mellifera	single end	2018	NA	2016	NA	NA	18133293	0	0	0	0
PRJNA450078	SRR7000615	Apis mellifera	single end	2018	NA	2016	NA	NA	19509267	0	0	0	0
PRJNA450078	SRR7000616	Apis mellifera	single end	2018	NA	2016	NA	NA	20162485	0	0	3	0
PRJNA450078	SRR7000617	Apis mellifera	single end	2018	NA	2016	NA	NA	12438055	0	0	0	0
PRJNA450078	SRR7000618	Apis mellifera	single end	2018	NA	2016	NA	NA	19359056	0	0	0	0
PRJNA450078	SRR7000619	Apis mellifera	single end	2018	NA	2016	NA	NA	22230494	0	0	0	0
PRJNA450078	SRR7000620	Apis mellifera	single end	2018	NA	2016	NA	NA	18860083	0	0	0	0
PRJNA450078	SRR7000621	Apis mellifera	single end	2018	NA	2016	NA	NA	17437800	0	0	0	0
PRJNA450078	SRR7000622	Apis mellifera	single end	2018	NA	2016	NA	NA	21193633	0	0	0	0
PRJNA450078	SRR7000623	Apis mellifera	single end	2018	NA	2016	NA	NA	19582225	0	0	0	0
PRJNA450078	SRR7000624	Apis mellifera	single end	2018	NA	2016	NA	NA	26285762	0	0	0	0
PRJNA450078	SRR7000625	Apis mellifera	single end	2018	NA	2016	NA	NA	18286584	0	0	0	0
PRJNA450078	SRR7000626	Apis mellifera	single end	2018	NA	2016	NA	NA	29846684	0	0	0	0
PRJNA450078	SRR7000627	Apis mellifera	single end	2018	NA	2016	NA	NA	33332142	0	0	0	0
PRJNA450078	SRR7000628	Apis mellifera	single end	2018	NA	2016	NA	NA	28145006	0	0	0	0
PRJNA450078	SRR7000629	Apis mellifera	single end	2018	NA	2016	NA	NA	23717827	2	0	0	0
PRJNA450078	SRR7000630	Apis mellifera	single end	2018	NA	2016	NA	NA	27591605	0	0	0	0
PRJNA450078	SRR7000631	Apis mellifera	single end	2018	NA	2016	NA	NA	24643340	0	0	0	0
PRJNA450078	SRR7000632	Apis mellifera	single end	2018	NA	2016	NA	NA	15630500	0	0	0	0
PRJNA450078	SRR7000633	Apis mellifera	single end	2018	NA	2016	NA	NA	28618429	0	0	2	0
PRJNA450078	SRR7000634	Apis mellifera	single end	2018	NA	2016	NA	NA	23575837	0	0	0	0
PRJNA450078	SRR7000635	Apis mellifera	single end	2018	NA	2016	NA	NA	9196122	0	0	0	0
PRJNA450078	SRR7000636	Apis mellifera	single end	2018	NA	2016	NA	NA	20708963	0	0	0	0
PRJNA450078	SRR7000637	Apis mellifera	single end	2018	NA	2016	NA	NA	20541709	1	0	0	0
PRJNA450078	SRR7000638	Apis mellifera	single end	2018	NA	2016	NA	NA	20424476	0	0	0	0
PRJNA450078	SRR7000639	Apis mellifera	single end	2018	NA	2016	NA	NA	20749444	0	0	0	0
PRJNA450078	SRR7000640	Apis mellifera	single end	2018	NA	2016	NA	NA	26160797	0	0	0	0
PRJNA450078	SRR7000641	Apis mellifera	single end	2018	NA	2016	NA	NA	20825010	0	0	0	0
PRJNA450078	SRR7000642	Apis mellifera	single end	2018	NA	2016	NA	NA	21444317	0	0	0	0
PRJNA450078	SRR7000643	Apis mellifera	single end	2018	NA	2016	NA	NA	20317942	0	0	0	0
PRJNA450078	SRR7000644	Apis mellifera	single end	2018	NA	2016	NA	NA	19097797	0	0	0	0
PRJNA450078	SRR7000645	Apis mellifera	single end	2018	NA	2016	NA	NA	21546842	0	0	0	0
PRJNA450078	SRR7000646	Apis mellifera	single end	2018	NA	2016	NA	NA	33791285	0	0	0	0
PRJNA450078	SRR7000647	Apis mellifera	single end	2018	NA	2016	NA	NA	23021761	0	0	0	0
PRJNA450078	SRR7000648	Apis mellifera	single end	2018	NA	2016	NA	NA	15549269	0	0	0	0
PRJNA450078	SRR7000649	Apis mellifera	single end	2018	NA	2016	NA	NA	13951667	0	0	0	0
PRJNA450078	SRR7000650	Apis mellifera	single end	2018	NA	2016	NA	NA	15405711	0	0	0	0
PRJNA450078	SRR7000651	Apis mellifera	single end	2018	NA	2016	NA	NA	15453811	0	0	0	0
PRJNA450078	SRR7000652	Apis mellifera	single end	2018	NA	2016	NA	NA	20781463	0	0	0	0
PRJNA450078	SRR7000653	Apis mellifera	single end	2018	NA	2016	NA	NA	26191108	0	0	0	0
PRJNA450078	SRR7000654	Apis mellifera	single end	2018	NA	2016	NA	NA	18973789	0	0	0	0
PRJNA450078	SRR7000655	Apis mellifera	single end	2018	NA	2016	NA	NA	21278540	0	0	0	0
PRJNA450078	SRR7000656	Apis mellifera	single end	2018	NA	2016	NA	NA	23971299	0	0	0	0
PRJNA450078	SRR7000657	Apis mellifera	single end	2018	NA	2016	NA	NA	22647649	4	0	0	0
PRJNA450078	SRR7000658	Apis mellifera	single end	2018	NA	2016	NA	NA	22867081	0	0	0	0
PRJNA450078	SRR7000659	Apis mellifera	single end	2018	NA	2016	NA	NA	18580835	0	0	0	0
PRJNA450078	SRR7000660	Apis mellifera	single end	2018	NA	2016	NA	NA	22510392	0	0	0	0
PRJNA450078	SRR7000661	Apis mellifera	single end	2018	NA	2016	NA	NA	20596663	2	0	3	0
PRJNA450078	SRR7000662	Apis mellifera	single end	2018	NA	2016	NA	NA	25934016	3	0	0	0
PRJNA450078	SRR7000663	Apis mellifera	single end	2018	NA	2016	NA	NA	25242880	1	0	0	0
PRJNA450078	SRR7000664	Apis mellifera	single end	2018	NA	2016	NA	NA	26395181	0	0	0	0
PRJNA450078	SRR7000665	Apis mellifera	single end	2018	NA	2016	NA	NA	23753753	0	0	0	0
PRJNA450078	SRR7000666	Apis mellifera	single end	2018	NA	2016	NA	NA	21695130	0	0	0	0
PRJNA450078	SRR7000667	Apis mellifera	single end	2018	NA	2016	NA	NA	28301778	0	0	0	0
PRJNA450078	SRR7000668	Apis mellifera	single end	2018	NA	2016	NA	NA	22461849	0	0	0	0
PRJNA450078	SRR7000669	Apis mellifera	single end	2018	NA	2016	NA	NA	25501510	1	0	0	0
PRJNA450078	SRR7000670	Apis mellifera	single end	2018	NA	2016	NA	NA	30579905	0	0	0	0
PRJNA450078	SRR7000671	Apis mellifera	single end	2018	NA	2016	NA	NA	24746119	0	0	0	0
PRJNA450078	SRR7000672	Apis mellifera	single end	2018	NA	2016	NA	NA	18268264	0	0	0	0
PRJNA450078	SRR7000673	Apis mellifera	single end	2018	NA	2016	NA	NA	21547732	22	0	0	0
PRJNA450078	SRR7000674	Apis mellifera	single end	2018	NA	2016	NA	NA	20799739	0	0	0	0
PRJNA450078	SRR7000675	Apis mellifera	single end	2018	NA	2016	NA	NA	17779186	1	0	0	0
PRJNA450078	SRR7000676	Apis mellifera	single end	2018	NA	2016	NA	NA	21730152	0	0	0	0
PRJNA450078	SRR7000677	Apis mellifera	single end	2018	NA	2016	NA	NA	19136440	0	0	1	0
PRJNA450078	SRR7000678	Apis mellifera	single end	2018	NA	2016	NA	NA	20844007	0	0	0	0
PRJNA450078	SRR7000679	Apis mellifera	single end	2018	NA	2016	NA	NA	17661465	0	0	0	0
PRJNA450078	SRR7000680	Apis mellifera	single end	2018	NA	2016	NA	NA	20773711	0	0	0	0
PRJNA450078	SRR7000681	Apis mellifera	single end	2018	NA	2016	NA	NA	19174662	0	0	1	0
PRJNA450078	SRR7000682	Apis mellifera	single end	2018	NA	2016	NA	NA	21539659	0	0	0	0
PRJNA450078	SRR7000683	Apis mellifera	single end	2018	NA	2016	NA	NA	17641847	0	0	0	0
PRJNA450078	SRR7000684	Apis mellifera	single end	2018	NA	2016	NA	NA	22224371	0	0	0	0
PRJNA450078	SRR7000685	Apis mellifera	single end	2018	NA	2016	NA	NA	22929755	0	0	0	0
PRJNA450078	SRR7000686	Apis mellifera	single end	2018	NA	2016	NA	NA	22073135	0	0	0	0
PRJNA450078	SRR7000687	Apis mellifera	single end	2018	NA	2016	NA	NA	22226899	0	0	0	0
PRJNA450078	SRR7000688	Apis mellifera	single end	2018	NA	2016	NA	NA	22614210	0	0	0	0

PRJNA450078	SRR7000689	Apis mellifera	single end	2018	NA	2016	NA	NA	24362794	0	0	0	0
PRJNA450078	SRR7000690	Apis mellifera	single end	2018	NA	2016	NA	NA	26314007	0	0	0	0
PRJNA450078	SRR7000691	Apis mellifera	single end	2018	NA	2016	NA	NA	26156859	0	0	0	0
PRJNA450078	SRR7000692	Apis mellifera	single end	2018	NA	2016	NA	NA	23432487	0	0	0	0
PRJNA450078	SRR7000693	Apis mellifera	single end	2018	NA	2016	NA	NA	18977617	0	0	0	0
PRJNA450078	SRR7000694	Apis mellifera	single end	2018	NA	2016	NA	NA	24902412	3	0	0	0
PRJNA450078	SRR7000695	Apis mellifera	single end	2018	NA	2016	NA	NA	19975403	0	0	0	0
PRJNA450078	SRR7000696	Apis mellifera	single end	2018	NA	2016	NA	NA	21473451	2	0	0	0
PRJNA450078	SRR7000697	Apis mellifera	single end	2018	NA	2016	NA	NA	25272808	3	0	0	0
PRJNA450078	SRR7000698	Apis mellifera	single end	2018	NA	2016	NA	NA	24393584	3	0	0	0
PRJNA450078	SRR7000699	Apis mellifera	single end	2018	NA	2016	NA	NA	23973763	1	0	0	0
PRJNA450078	SRR7000700	Apis mellifera	single end	2018	NA	2016	NA	NA	24876095	0	0	0	0
PRJNA450078	SRR7000701	Apis mellifera	single end	2018	NA	2016	NA	NA	27831847	0	0	0	0
PRJNA450078	SRR7000702	Apis mellifera	single end	2018	NA	2016	NA	NA	15251429	0	0	0	0
PRJNA450078	SRR7000703	Apis mellifera	single end	2018	NA	2016	NA	NA	34409718	0	0	0	0
PRJNA450078	SRR7000704	Apis mellifera	single end	2018	NA	2016	NA	NA	23651806	0	0	0	0
PRJNA450078	SRR7000705	Apis mellifera	single end	2018	NA	2016	NA	NA	21156240	0	0	0	0
PRJNA450078	SRR7000706	Apis mellifera	single end	2018	NA	2016	NA	NA	25148343	0	0	0	0
PRJNA450078	SRR7000707	Apis mellifera	single end	2018	NA	2016	NA	NA	27433441	0	0	0	0
PRJNA450078	SRR7000708	Apis mellifera	single end	2018	NA	2016	NA	NA	25360702	0	0	0	0
PRJNA450078	SRR7000709	Apis mellifera	single end	2018	NA	2016	NA	NA	29137702	0	0	0	0
PRJNA450078	SRR7000710	Apis mellifera	single end	2018	NA	2016	NA	NA	26078253	1	0	0	0
PRJNA450078	SRR7000711	Apis mellifera	single end	2018	NA	2016	NA	NA	29053011	0	0	0	0
PRJNA450078	SRR7000712	Apis mellifera	single end	2018	NA	2016	NA	NA	30053315	0	0	0	0
PRJNA450078	SRR7000713	Apis mellifera	single end	2018	NA	2016	NA	NA	30012605	0	0	0	0
PRJNA450078	SRR7000714	Apis mellifera	single end	2018	NA	2016	NA	NA	26497917	0	0	0	0
PRJNA450078	SRR7000715	Apis mellifera	single end	2018	NA	2016	NA	NA	26218534	0	0	1	0
PRJNA450078	SRR7000716	Apis mellifera	single end	2018	NA	2016	NA	NA	27151687	0	0	0	0
PRJNA450078	SRR7000717	Apis mellifera	single end	2018	NA	2016	NA	NA	22475117	0	0	0	0
PRJNA450078	SRR7000718	Apis mellifera	single end	2018	NA	2016	NA	NA	27731133	0	0	0	0
PRJNA450078	SRR7000719	Apis mellifera	single end	2018	NA	2016	NA	NA	27940760	0	0	0	0
PRJNA450078	SRR7000720	Apis mellifera	single end	2018	NA	2016	NA	NA	27646430	0	0	0	0
PRJNA450078	SRR7000721	Apis mellifera	single end	2018	NA	2016	NA	NA	23914459	0	0	0	0
PRJNA450078	SRR7000722	Apis mellifera	single end	2018	NA	2016	NA	NA	24511679	0	0	0	0
PRJNA450078	SRR7000723	Apis mellifera	single end	2018	NA	2016	NA	NA	26515086	0	0	0	0
PRJNA450078	SRR7000724	Apis mellifera	single end	2018	NA	2016	NA	NA	27172631	0	0	0	0
PRJNA450078	SRR7000725	Apis mellifera	single end	2018	NA	2016	NA	NA	16998535	0	0	0	0
PRJNA450078	SRR7000726	Apis mellifera	single end	2018	NA	2016	NA	NA	30440885	0	0	0	0
PRJNA450078	SRR7000727	Apis mellifera	single end	2018	NA	2016	NA	NA	35680172	23	0	0	0
PRJNA450078	SRR7000728	Apis mellifera	single end	2018	NA	2016	NA	NA	20962393	3	0	0	0
PRJNA450078	SRR7000729	Apis mellifera	single end	2018	NA	2016	NA	NA	19724061	0	0	0	0
PRJNA450078	SRR7000730	Apis mellifera	single end	2018	NA	2016	NA	NA	18540883	0	0	0	0
PRJNA450078	SRR7000731	Apis mellifera	single end	2018	NA	2016	NA	NA	16970852	0	0	0	0
PRJNA450078	SRR7000732	Apis mellifera	single end	2018	NA	2016	NA	NA	17858860	0	0	0	0
PRJNA450078	SRR7000733	Apis mellifera	single end	2018	NA	2016	NA	NA	20336419	0	0	0	0
PRJNA450078	SRR7000734	Apis mellifera	single end	2018	NA	2016	NA	NA	30756406	0	0	0	0
PRJNA450078	SRR7000735	Apis mellifera	single end	2018	NA	2016	NA	NA	30462174	0	0	0	0
PRJNA450078	SRR7000736	Apis mellifera	single end	2018	NA	2016	NA	NA	14333112	0	0	0	0
PRJNA450078	SRR7000737	Apis mellifera	single end	2018	NA	2016	NA	NA	26818350	0	0	0	0
PRJNA450078	SRR7000738	Apis mellifera	single end	2018	NA	2016	NA	NA	15614549	0	0	0	0
PRJNA450078	SRR7000739	Apis mellifera	single end	2018	NA	2016	NA	NA	17894601	0	0	0	0
PRJNA450078	SRR7000740	Apis mellifera	single end	2018	NA	2016	NA	NA	23439930	0	0	0	0
PRJNA450078	SRR7000741	Apis mellifera	single end	2018	NA	2016	NA	NA	21952876	0	0	0	0
PRJNA450078	SRR7000742	Apis mellifera	single end	2018	NA	2016	NA	NA	25553227	0	0	0	0
PRJNA450078	SRR7000743	Apis mellifera	single end	2018	NA	2016	NA	NA	19512202	0	0	0	0
PRJNA450078	SRR7000744	Apis mellifera	single end	2018	NA	2016	NA	NA	24150561	0	0	0	0
PRJNA450078	SRR7000745	Apis mellifera	single end	2018	NA	2016	NA	NA	24912783	0	0	0	0
PRJNA450078	SRR7000747	Apis mellifera	single end	2018	NA	2016	NA	NA	24015150	0	0	0	0
PRJNA450078	SRR7000748	Apis mellifera	single end	2018	NA	2016	NA	NA	23676841	0	0	0	0
PRJNA450078	SRR7000749	Apis mellifera	single end	2018	NA	2016	NA	NA	24601596	0	0	0	0
PRJNA450078	SRR7000750	Apis mellifera	single end	2018	NA	2016	NA	NA	25052952	0	0	0	0
PRJNA450078	SRR7000751	Apis mellifera	single end	2018	NA	2016	NA	NA	24934015	0	0	0	0
PRJNA450078	SRR7000752	Apis mellifera	single end	2018	NA	2016	NA	NA	25489470	0	0	0	0
PRJNA450296	SRR7006968	Apis mellifera	single end	2019	NA	2017	NA	NA	39390754	0	0	2	0
PRJNA450296	SRR7006969	Apis mellifera	single end	2019	NA	2017	NA	NA	37034849	0	0	1	0
PRJNA450296	SRR7006970	Apis mellifera	single end	2019	NA	2017	NA	NA	37836619	0	0	1	0
PRJNA450296	SRR7006971	Apis mellifera	single end	2019	NA	2017	NA	NA	36679811	0	0	0	0
PRJNA450296	SRR7006972	Apis mellifera	single end	2019	NA	2017	NA	NA	38235449	0	0	2	0
PRJNA450296	SRR7006973	Apis mellifera	single end	2019	NA	2017	NA	NA	39541196	0	0	5	0
PRJNA450296	SRR7006974	Apis mellifera	single end	2019	NA	2017	NA	NA	43092778	0	0	1	0
PRJNA450296	SRR7006975	Apis mellifera	single end	2019	NA	2017	NA	NA	46858241	0	0	0	0
PRJNA450296	SRR7006976	Apis mellifera	single end	2019	NA	2017	NA	NA	40093974	0	0	0	0
PRJNA450296	SRR7006977	Apis mellifera	single end	2019	NA	2017	NA	NA	53086126	0	0	0	0
PRJNA450296	SRR7006978	Apis mellifera	single end	2019	NA	2017	NA	NA	26515570	0	0	0	0
PRJNA450296	SRR7006979	Apis mellifera	single end	2019	NA	2017	NA	NA	31583861	0	0	0	0
PRJNA450296	SRR7006980	Apis mellifera	single end	2019	NA	2017	NA	NA	34670974	0	0	0	0
PRJNA450296	SRR7006981	Apis mellifera	single end	2019	NA	2017	NA	NA	35204521	0	0	0	0
PRJNA450296	SRR7006982	Apis mellifera	single end	2019	NA	2017	NA	NA	42582787	0	0	0	0
PRJNA450296	SRR7006983	Apis mellifera	single end	2019	NA	2017	NA	NA	40181697	0	0	0	0
PRJNA450296	SRR7006984	Apis mellifera	single end	2019	NA	2017	NA	NA	30098947	0	0	0	0
PRJNA450296	SRR7006985	Apis mellifera	single end	2019	NA	2017	NA	NA	34498988	0	0	0	0
PRJNA450296	SRR7006986	Apis mellifera	single end	2019	NA	2017	NA	NA	36511805	0	0	0	0
PRJNA450296	SRR7006987	Apis mellifera	single end	2019	NA	2017	NA	NA	33943617	0	0	10	0
PRJNA450296	SRR7006988	Apis mellifera	single end	2019	NA	2017	NA	NA	38509096	0	0	0	0
PRJNA450296	SRR7006989	Apis mellifera	single end	2019	NA	2017	NA	NA	39337662	0	0	0	0
PRJNA450296	SRR7006990	Apis mellifera	single end	2019	NA	2017	NA	NA	714525	0	0	0	0
PRJNA450296	SRR7006991	Apis mellifera	single end	2019	NA	2017	NA	NA	49432273	0	0	0	0
PRJNA450296	SRR7006992	Apis mellifera	single end	2019	NA	2017	NA	NA	35956486	0	0	0	0
PRJNA450296	SRR7006993	Apis mellifera	single end	2019	NA	2017	NA	NA	42958298	0	0	0	0
PRJNA450296	SRR7006994	Apis mellifera	single end	2019	NA	2017	NA	NA	31612771	0	0	0	0
PRJNA450296	SRR7006995	Apis mellifera	single end	2019	NA	2017	NA	NA	31085450	0	0	0	0
PRJNA450296	SRR7006996	Apis mellifera	single end	2019	NA	2017	NA	NA	33861248	0	0	3	0
PRJNA450296	SRR7006997	Apis mellifera	single end	2019	NA	2017	NA	NA	40291727	0	0	0	0
PRJNA450296	SRR7006998	Apis mellifera	single end	2019	NA	2017	NA	NA	33348437	0	0	0	0
PRJNA450296	SRR7006999	Apis mellifera	single end	2019	NA	2017	NA	NA	47012416	0	0	2	0
PRJNA450296	SRR7007000	Apis mellifera	single end	2019	NA	2017	NA	NA	53358245	0	0	0	0
PRJNA450296	SRR7007001	Apis mellifera	single end	2019	NA	2017	NA	NA	31311001	0	0	0	0
PRJNA450296	SRR7007002	Apis mellifera	single end	2019	NA	2017	NA	NA	26361335	0	0	0	0
PRJNA450296	SRR7007003	Apis mellifera	single end	2019	NA	2017	NA	NA	40105055	0	0	0	0
PRJNA450296	SRR7007004	Apis mellifera	single end	2019	NA	2017	NA	NA	35777718	0	0	0	0
PRJNA450296	SRR7007005	Apis mellifera	single end	2019	NA	2017	NA	NA	37363020	0	0	0	0
PRJNA450296	SRR7007006	Apis mellifera	single end	2019	NA	2017	NA	NA	46344747	0	0	0	0
PRJNA450296	SRR7007007	Apis mellifera	single end	2019	NA	2017	NA	NA	31726667	0	0	0	0
PRJNA470779	SRR7143860	Apis mellifera	single end	2018	NA	2016	NA	NA	5307193	0	0	0	0
PRJNA470779	SRR7143861	Apis mellifera	single end	2018	NA	2016	NA	NA	4153679	0	0	0	0
PRJNA470779	SRR7143862	Apis mellifera	single end	2018	NA	2016	NA	NA	2770714	0	0	0	0

PRJNA470779	SRR7143863	Apis mellifera	single end	2018	NA	2016	NA	NA	3912692	0	0	0	0
PRJNA470779	SRR7143864	Apis mellifera	single end	2018	NA	2016	NA	NA	2710367	0	0	0	0
PRJNA470779	SRR7143865	Apis mellifera	single end	2018	NA	2016	NA	NA	2971306	0	0	0	0
PRJNA470779	SRR7143866	Apis mellifera	single end	2018	NA	2016	NA	NA	4407222	0	0	0	0
PRJNA470779	SRR7143867	Apis mellifera	single end	2018	NA	2016	NA	NA	8365812	0	0	0	0
PRJNA470779	SRR7143868	Apis mellifera	single end	2018	NA	2016	NA	NA	1699340	0	0	0	0
PRJNA477521	SRR7412452	Apis mellifera	single end	2018	2015	2015	Czech	Europe	65781	0	0	0	0
PRJNA477521	SRR7412453	Apis mellifera	single end	2018	2015	2015	Czech	Europe	96482	0	0	0	0
PRJNA477521	SRR7412454	Apis mellifera	single end	2018	2015	2015	Czech	Europe	83075	0	0	0	0
PRJNA477521	SRR7412455	Apis mellifera	single end	2018	2015	2015	Czech	Europe	80566	0	0	0	0
PRJNA477521	SRR7412458	Apis mellifera	single end	2018	2017	2017	Czech	Europe	106457	0	0	0	0
PRJNA477521	SRR7412459	Apis mellifera	single end	2018	2017	2017	Czech	Europe	116482	0	0	0	0
PRJNA477521	SRR7412460	Apis mellifera	single end	2018	2017	2017	Czech	Europe	109209	0	0	0	0
PRJNA477521	SRR7412461	Apis mellifera	single end	2018	2017	2017	Czech	Europe	110363	0	0	0	0
PRJNA477521	SRR7412462	Apis mellifera	single end	2018	2017	2017	Czech	Europe	97147	0	0	0	0
PRJNA477521	SRR7412463	Apis mellifera	single end	2018	2017	2017	Czech	Europe	106912	0	0	0	0
PRJNA477521	SRR7412464	Apis mellifera	single end	2018	2017	2017	Czech	Europe	108758	0	0	0	0
PRJNA477521	SRR7412465	Apis mellifera	single end	2018	2017	2017	Czech	Europe	98520	0	0	0	0
PRJNA477521	SRR7412466	Apis mellifera	single end	2018	2017	2017	Czech	Europe	125272	0	0	0	0
PRJNA477521	SRR7412467	Apis mellifera	single end	2018	2017	2017	Czech	Europe	104169	0	0	0	0
PRJNA477521	SRR7412468	Apis mellifera	single end	2018	2015	2015	Czech	Europe	75590	0	0	0	0
PRJNA477521	SRR7412469	Apis mellifera	single end	2018	2015	2015	Czech	Europe	49219	0	0	0	0
PRJNA477521	SRR7412470	Apis mellifera	single end	2018	2015	2015	Czech	Europe	62758	0	0	0	0
PRJNA477521	SRR7412471	Apis mellifera	single end	2018	2015	2015	Czech	Europe	69444	0	0	0	0
PRJNA477521	SRR7412472	Apis mellifera	single end	2018	2015	2015	Czech	Europe	85291	0	0	0	0
PRJNA477521	SRR7412473	Apis mellifera	single end	2018	2015	2015	Czech	Europe	60723	0	0	0	0
PRJNA477521	SRR7412474	Apis mellifera	single end	2018	2015	2015	Czech	Europe	70654	0	0	0	0
PRJNA477521	SRR7412475	Apis mellifera	single end	2018	2015	2015	Czech	Europe	91600	0	0	0	0
PRJNA477521	SRR7412476	Apis mellifera	single end	2018	2015	2015	Czech	Europe	57398	0	0	0	0
PRJNA477521	SRR7412477	Apis mellifera	single end	2018	2015	2015	Czech	Europe	70758	0	0	0	0
PRJNA477521	SRR7412478	Apis mellifera	single end	2018	2017	2017	Czech	Europe	123844	0	0	0	0
PRJNA477521	SRR7412479	Apis mellifera	single end	2018	2017	2017	Czech	Europe	118020	0	0	0	0
PRJNA477521	SRR7412480	Apis mellifera	single end	2018	2017	2017	Czech	Europe	105841	0	0	0	0
PRJNA477521	SRR7412481	Apis mellifera	single end	2018	2017	2017	Czech	Europe	116880	0	0	0	0
PRJNA477521	SRR7412482	Apis mellifera	single end	2018	2017	2017	Czech	Europe	112103	0	0	0	0
PRJNA477521	SRR7412483	Apis mellifera	single end	2018	2017	2017	Czech	Europe	113019	0	0	0	0
PRJNA477521	SRR7412484	Apis mellifera	single end	2018	2017	2017	Czech	Europe	104968	0	0	0	0
PRJNA477521	SRR7412485	Apis mellifera	single end	2018	2017	2017	Czech	Europe	97301	0	0	0	0
PRJNA477521	SRR7412486	Apis mellifera	single end	2018	2017	2017	Czech	Europe	119204	0	0	0	0
PRJNA477521	SRR7412487	Apis mellifera	single end	2018	2017	2017	Czech	Europe	94610	0	0	0	0
PRJNA477521	SRR7412488	Apis mellifera	single end	2018	2015	2015	Czech	Europe	73300	0	0	0	0
PRJNA477521	SRR7412489	Apis mellifera	single end	2018	2015	2015	Czech	Europe	77570	0	0	0	0
PRJNA477521	SRR7412490	Apis mellifera	single end	2018	2015	2015	Czech	Europe	65990	0	0	0	0
PRJNA477521	SRR7412491	Apis mellifera	single end	2018	2015	2015	Czech	Europe	73297	0	0	0	0
PRJNA477521	SRR7412492	Apis mellifera	single end	2018	2015	2015	Czech	Europe	82071	0	0	0	0
PRJNA477521	SRR7412493	Apis mellifera	single end	2018	2015	2015	Czech	Europe	80085	0	0	0	0
PRJNA477521	SRR7412494	Apis mellifera	single end	2018	2015	2015	Czech	Europe	82363	0	0	0	0
PRJNA477521	SRR7412495	Apis mellifera	single end	2018	2015	2015	Czech	Europe	74555	0	0	0	0
PRJNA477521	SRR7412496	Apis mellifera	single end	2018	2015	2015	Czech	Europe	69953	0	0	0	0
PRJNA477521	SRR7412497	Apis mellifera	single end	2018	2015	2015	Czech	Europe	83171	0	0	0	0
PRJNA477521	SRR7412498	Apis mellifera	single end	2018	2017	2017	Czech	Europe	106449	0	0	0	0
PRJNA477521	SRR7412499	Apis mellifera	single end	2018	2017	2017	Czech	Europe	116533	0	0	0	0
PRJNA477521	SRR7412500	Apis mellifera	single end	2018	2017	2017	Czech	Europe	82924	0	0	0	0
PRJNA477521	SRR7412501	Apis mellifera	single end	2018	2017	2017	Czech	Europe	99521	0	0	0	0
PRJNA477521	SRR7412502	Apis mellifera	single end	2018	2017	2017	Czech	Europe	110046	0	0	0	0
PRJNA477521	SRR7412503	Apis mellifera	single end	2018	2017	2017	Czech	Europe	92840	0	0	0	0
PRJNA477521	SRR7412504	Apis mellifera	single end	2018	2017	2017	Czech	Europe	60323	0	0	0	0
PRJNA477521	SRR7412505	Apis mellifera	single end	2018	2017	2017	Czech	Europe	90387	0	0	0	0
PRJNA477521	SRR7412506	Apis mellifera	single end	2018	2017	2017	Czech	Europe	84175	0	0	0	0
PRJNA477521	SRR7412507	Apis mellifera	single end	2018	2017	2017	Czech	Europe	82543	0	0	0	0
PRJNA477521	SRR7412508	Apis mellifera	single end	2018	2017	2017	Czech	Europe	94550	0	0	0	0
PRJNA477521	SRR7412509	Apis mellifera	single end	2018	2017	2017	Czech	Europe	98899	0	0	0	0
PRJNA477521	SRR7412510	Apis mellifera	single end	2018	2017	2017	Czech	Europe	26916	0	0	0	0
PRJNA477521	SRR7412511	Apis mellifera	single end	2018	2017	2017	Czech	Europe	105393	0	0	0	0
PRJNA477521	SRR7412512	Apis mellifera	single end	2018	2017	2017	Czech	Europe	51273	0	0	0	0
PRJNA477521	SRR7412513	Apis mellifera	single end	2018	2017	2017	Czech	Europe	73323	0	0	0	0
PRJNA479740	SRR7472351	Apis mellifera	single end	2019	NA	2017	Israel	Asia	91982369	1197	0	0	0
PRJNA479740	SRR7472352	Apis mellifera	single end	2019	NA	2017	Israel	Asia	85455513	67	0	0	0
PRJNA479740	SRR7472353	Apis mellifera	single end	2019	NA	2017	Israel	Asia	78911350	0	0	0	0
PRJNA479740	SRR7472354	Apis mellifera	single end	2019	NA	2017	Israel	Asia	68198141	4037358	5.92	1394	0
PRJNA479740	SRR7472355	Apis mellifera	single end	2019	NA	2017	Israel	Asia	71335775	73	0	0	0
PRJNA479740	SRR7472356	Apis mellifera	single end	2019	NA	2017	Israel	Asia	82270736	2189	0	1	0
PRJNA479740	SRR7472357	Apis mellifera	single end	2019	NA	2017	Israel	Asia	77033392	8978778	11.66	1061	0
PRJNA479740	SRR7472358	Apis mellifera	single end	2019	NA	2017	Israel	Asia	83946531	40	0	0	0
PRJNA479740	SRR7472359	Apis mellifera	single end	2019	NA	2017	Israel	Asia	90571141	5	0	1	0
PRJNA479740	SRR7472360	Apis mellifera	single end	2019	NA	2017	Israel	Asia	92133396	11	0	0	0
PRJNA479740	SRR7472361	Apis mellifera	single end	2019	NA	2017	Israel	Asia	53706119	53436	0.1	8	0
PRJNA479740	SRR7472362	Apis mellifera	single end	2019	NA	2017	Israel	Asia	131938627	61	0	0	0
PRJNA479740	SRR7472363	Apis mellifera	single end	2019	NA	2017	Israel	Asia	57406008	731	0	1	0
PRJNA172020	SRR765681	Apis mellifera	single end	2015	NA	2013	NA	NA	29462269	983595	3.34	259	0
PRJNA192195	SRR771439	Apis mellifera	single end	2014	NA	2012	NA	NA	6717618	425	0.01	13	0
PRJNA192195	SRR771440	Apis mellifera	single end	2014	NA	2012	NA	NA	7308203	1395	0.02	38	0
PRJNA192448	SRR771525	Apis mellifera	single end	2015	NA	2013	NA	NA	3649743	0	0	0	0
PRJNA192448	SRR771526	Apis mellifera	single end	2015	NA	2013	NA	NA	3727670	0	0	0	0
PRJNA488619	SRR7770431	Apis mellifera	single end	2019	NA	2017	China	Asia	13536823	28	0	1	0
PRJNA488619	SRR7770434	Apis mellifera	single end	2019	NA	2017	China	Asia	12986893	818	0.01	30	0
PRJNA488619	SRR7770437	Apis mellifera	single end	2019	NA	2017	China	Asia	13474091	3263	0.02	48	0
PRJNA193691	SRR789759	Apis mellifera	single end	2013	NA	2011	NA	NA	175796462	0	0	47	0
PRJNA193691	SRR789760	Apis mellifera	single end	2013	NA	2011	NA	NA	168267063	0	0	207	0
PRJNA193691	SRR789761	Apis mellifera	single end	2013	NA	2011	NA	NA	178934269	1	0	573	0
PRJNA193691	SRR789762	Apis mellifera	single end	2013	NA	2011	NA	NA	189487841	1	0	142	0
PRJNA193691	SRR789763	Apis mellifera	single end	2013	NA	2011	NA	NA	162754857	0	0	50	0
PRJNA193691	SRR789764	Apis mellifera	single end	2013	NA	2011	NA	NA	153692102	0	0	1352	0
PRJNA493573	SRR7908186	Apis mellifera	single end	2019	NA	2017	NA	NA	7160479	8	0	0	0
PRJNA493573	SRR7908187	Apis mellifera	single end	2019	NA	2017	NA	NA	6964277	2	0	0	0
PRJNA493573	SRR7908188	Apis mellifera	single end	2019	NA	2017	NA	NA	7503621	0	0	0	0
PRJNA493573	SRR7908189	Apis mellifera	single end	2019	NA	2017	NA	NA	7008828	0	0	0	0
PRJNA493573	SRR7908190	Apis mellifera	single end	2019	NA	2017	NA	NA	7192461	267	0	0	0
PRJNA493573	SRR7908191	Apis mellifera	single end	2019	NA	2017	NA	NA	7196797	270	0	0	0
PRJNA498667	SRR8113905	Apis mellifera	single end	2018	2012	2012	China	Asia	6048878	177712	2.94	3698	0.06
PRJNA498919	SRR8121077	Apis mellifera	single end	2018	NA	2016	NA	NA	7829091	1	0	1	0
PRJNA498919	SRR8121078	Apis mellifera	single end	2018	NA	2016	NA	NA	7985849	3	0	0	0
PRJNA498919	SRR8121079	Apis mellifera	single end	2018	NA	2016	NA	NA	6999266	3	0	0	0
PRJNA498919	SRR8121080	Apis mellifera	single end	2018	NA	2016	NA	NA	7122846	5	0	0	0
PRJNA498919	SRR8121081	Apis mellifera	single end	2018	NA	2016	NA	NA	7381546	3893	0.05	1	0
PRJNA498919	SRR8121082	Apis mellifera	single end	2018	NA	2016	NA	NA	7518704	3981	0.05	1	0

PRJNA498919	SRR8121083	Apis mellifera	single end	2018	NA	2016	NA	NA	6623278	4	0	1	0
PRJNA498919	SRR8121084	Apis mellifera	single end	2018	NA	2016	NA	NA	6757394	2	0	1	0
PRJNA498919	SRR8121085	Apis mellifera	single end	2018	NA	2016	NA	NA	5713379	0	0	0	0
PRJNA498919	SRR8121086	Apis mellifera	single end	2018	NA	2016	NA	NA	5701450	1	0	0	0
PRJNA498919	SRR8121087	Apis mellifera	single end	2018	NA	2016	NA	NA	5076375	0	0	1	0
PRJNA498919	SRR8121088	Apis mellifera	single end	2018	NA	2016	NA	NA	5079272	2	0	0	0
PRJNA498919	SRR8121089	Apis mellifera	single end	2018	NA	2016	NA	NA	6681281	8	0	3	0
PRJNA498919	SRR8121090	Apis mellifera	single end	2018	NA	2016	NA	NA	6806552	7	0	2	0
PRJNA498919	SRR8121091	Apis mellifera	single end	2018	NA	2016	NA	NA	5392764	6	0	1	0
PRJNA498919	SRR8121092	Apis mellifera	single end	2018	NA	2016	NA	NA	5513219	2	0	0	0
PRJNA498919	SRR8121093	Apis mellifera	single end	2018	NA	2016	NA	NA	6536387	6	0	4	0
PRJNA498919	SRR8121094	Apis mellifera	single end	2018	NA	2016	NA	NA	6519216	5	0	2	0
PRJNA498919	SRR8121095	Apis mellifera	single end	2018	NA	2016	NA	NA	6571468	1383	0.02	17	0
PRJNA498919	SRR8121096	Apis mellifera	single end	2018	NA	2016	NA	NA	6549426	1342	0.02	15	0
PRJNA498919	SRR8121097	Apis mellifera	single end	2018	NA	2016	NA	NA	7858990	3	0	1	0
PRJNA498919	SRR8121098	Apis mellifera	single end	2018	NA	2016	NA	NA	7845242	2	0	2	0
PRJNA498919	SRR8121099	Apis mellifera	single end	2018	NA	2016	NA	NA	7033175	2	0	2	0
PRJNA498919	SRR8121100	Apis mellifera	single end	2018	NA	2016	NA	NA	7019872	3	0	1	0
PRJNA498919	SRR8121101	Apis mellifera	single end	2018	NA	2016	NA	NA	4941484	1	0	0	0
PRJNA498919	SRR8121102	Apis mellifera	single end	2018	NA	2016	NA	NA	5049220	3	0	0	0
PRJNA498919	SRR8121103	Apis mellifera	single end	2018	NA	2016	NA	NA	7000364	1	0	0	0
PRJNA498919	SRR8121104	Apis mellifera	single end	2018	NA	2016	NA	NA	7141426	1	0	0	0
PRJNA498919	SRR8121105	Apis mellifera	single end	2018	NA	2016	NA	NA	7351445	5	0	0	0
PRJNA498919	SRR8121106	Apis mellifera	single end	2018	NA	2016	NA	NA	7489702	9	0	0	0
PRJNA498919	SRR8121107	Apis mellifera	single end	2018	NA	2016	NA	NA	8824889	2	0	1	0
PRJNA498919	SRR8121108	Apis mellifera	single end	2018	NA	2016	NA	NA	8998819	2	0	0	0
PRJNA498919	SRR8121109	Apis mellifera	single end	2018	NA	2016	NA	NA	8209434	209	0	0	0
PRJNA498919	SRR8121110	Apis mellifera	single end	2018	NA	2016	NA	NA	8214288	210	0	2	0
PRJNA498919	SRR8121111	Apis mellifera	single end	2018	NA	2016	NA	NA	7035205	7	0	0	0
PRJNA498919	SRR8121112	Apis mellifera	single end	2018	NA	2016	NA	NA	7011817	9	0	0	0
PRJNA498919	SRR8121113	Apis mellifera	single end	2018	NA	2016	NA	NA	7145807	3	0	3	0
PRJNA498919	SRR8121114	Apis mellifera	single end	2018	NA	2016	NA	NA	7291094	8	0	5	0
PRJNA498919	SRR8121115	Apis mellifera	single end	2018	NA	2016	NA	NA	6617980	8	0	1	0
PRJNA498919	SRR8121116	Apis mellifera	single end	2018	NA	2016	NA	NA	6746529	6	0	3	0
PRJNA498919	SRR8121117	Apis mellifera	single end	2018	NA	2016	NA	NA	6572678	2	0	2	0
PRJNA498919	SRR8121118	Apis mellifera	single end	2018	NA	2016	NA	NA	6564364	3	0	1	0
PRJNA498919	SRR8121119	Apis mellifera	single end	2018	NA	2016	NA	NA	6599951	1	0	3	0
PRJNA498919	SRR8121120	Apis mellifera	single end	2018	NA	2016	NA	NA	6599505	5	0	1	0
PRJNA498919	SRR8121121	Apis mellifera	single end	2018	NA	2016	NA	NA	7396232	3	0	21	0
PRJNA498919	SRR8121122	Apis mellifera	single end	2018	NA	2016	NA	NA	7404053	4	0	23	0
PRJNA498919	SRR8121123	Apis mellifera	single end	2018	NA	2016	NA	NA	5832551	1	0	0	0
PRJNA498919	SRR8121124	Apis mellifera	single end	2018	NA	2016	NA	NA	5815553	2	0	0	0
PRJNA509310	SRR8297880	Apis mellifera	single end	2019	NA	2017	NA	NA	25151700	0	0	0	0
PRJNA509310	SRR8297881	Apis mellifera	single end	2019	NA	2017	NA	NA	26931529	0	0	0	0
PRJNA509310	SRR8297882	Apis mellifera	single end	2019	NA	2017	NA	NA	31153280	0	0	0	0
PRJNA509310	SRR8297883	Apis mellifera	single end	2019	NA	2017	NA	NA	11946112	0	0	0	0
PRJNA509310	SRR8297884	Apis mellifera	single end	2019	NA	2017	NA	NA	29411726	0	0	0	0
PRJNA509310	SRR8297885	Apis mellifera	single end	2019	NA	2017	NA	NA	29534124	0	0	0	0
PRJNA509310	SRR8297886	Apis mellifera	single end	2019	NA	2017	NA	NA	24099805	0	0	0	0
PRJNA509310	SRR8297887	Apis mellifera	single end	2019	NA	2017	NA	NA	61142238	0	0	0	0
PRJNA509310	SRR8297888	Apis mellifera	single end	2019	NA	2017	NA	NA	30770220	0	0	0	0
PRJNA509310	SRR8297889	Apis mellifera	single end	2019	NA	2017	NA	NA	21854918	0	0	0	0
PRJNA509310	SRR8297890	Apis mellifera	single end	2019	NA	2017	NA	NA	23006419	0	0	0	0
PRJNA509310	SRR8297891	Apis mellifera	single end	2019	NA	2017	NA	NA	22773174	0	0	0	0
PRJNA200755	SRR838836	Apis mellifera	single end	2015	NA	2013	China	Asia	5968823	0	0	0	0
PRJNA200755	SRR838837	Apis mellifera	single end	2015	NA	2013	China	Asia	6235889	0	0	0	0
PRJNA200755	SRR838838	Apis mellifera	single end	2015	NA	2013	China	Asia	6044451	0	0	0	0
PRJNA201010	SRR839279	Apis mellifera	single end	2015	2011	2011	China	Asia	5812242	0	0	0	0
PRJNA201010	SRR839350	Apis mellifera	single end	2015	2011	2011	China	Asia	5769851	0	0	0	0
PRJNA201386	SRR850492	Apis mellifera	single end	2015	2011	2011	China	Asia	5814195	0	0	0	0
PRJNA201386	SRR850493	Apis mellifera	single end	2015	2011	2011	China	Asia	6071332	0	0	0	0
PRJNA524311	SRR8632761	Apis mellifera	single end	2019	NA	2017	NA	NA	44727939	0	0	0	0
PRJNA524311	SRR8632762	Apis mellifera	single end	2019	NA	2017	NA	NA	38806853	0	0	0	0
PRJNA524311	SRR8632763	Apis mellifera	single end	2019	NA	2017	NA	NA	51076634	0	0	0	0
PRJNA524311	SRR8632764	Apis mellifera	single end	2019	NA	2017	NA	NA	74375492	0	0	0	0
PRJNA524311	SRR8632765	Apis mellifera	single end	2019	NA	2017	NA	NA	68303763	0	0	0	0
PRJNA524311	SRR8632766	Apis mellifera	single end	2019	NA	2017	NA	NA	56388221	0	0	0	0
PRJNA524311	SRR8632767	Apis mellifera	single end	2019	NA	2017	NA	NA	59082926	0	0	0	0
PRJNA524311	SRR8632768	Apis mellifera	single end	2019	NA	2017	NA	NA	50519949	0	0	0	0
PRJNA524311	SRR8632769	Apis mellifera	single end	2019	NA	2017	NA	NA	55420982	0	0	0	0
PRJNA524311	SRR8632770	Apis mellifera	single end	2019	NA	2017	NA	NA	45973938	0	0	0	0
PRJNA524311	SRR8632771	Apis mellifera	single end	2019	NA	2017	NA	NA	57558336	0	0	0	0
PRJNA524311	SRR8632772	Apis mellifera	single end	2019	NA	2017	NA	NA	49949665	0	0	0	0
PRJNA524311	SRR8632773	Apis mellifera	single end	2019	NA	2017	NA	NA	47790096	0	0	0	0
PRJNA524311	SRR8632774	Apis mellifera	single end	2019	NA	2017	NA	NA	53142699	1	0	0	0
PRJNA524311	SRR8632775	Apis mellifera	single end	2019	NA	2017	NA	NA	62186933	0	0	0	0
PRJNA524311	SRR8632776	Apis mellifera	single end	2019	NA	2017	NA	NA	49571778	0	0	0	0
PRJNA524311	SRR8632777	Apis mellifera	single end	2019	NA	2017	NA	NA	53911242	0	0	0	0
PRJNA524311	SRR8632778	Apis mellifera	single end	2019	NA	2017	NA	NA	63554891	0	0	0	0
PRJNA203871	SRR863701	Apis mellifera	single end	2015	NA	2013	NA	NA	3573941	0	0	0	0
PRJNA203871	SRR863702	Apis mellifera	single end	2015	NA	2013	NA	NA	3562730	0	0	0	0
PRJNA203871	SRR863703	Apis mellifera	single end	2015	NA	2013	NA	NA	3551541	0	0	0	0
PRJNA203871	SRR863704	Apis mellifera	single end	2015	NA	2013	NA	NA	3524453	0	0	0	0
PRJNA203871	SRR863705	Apis mellifera	single end	2015	NA	2013	NA	NA	3615558	0	0	0	0
PRJNA204963	SRR866904	Apis mellifera	single end	2015	NA	2013	China	Asia	6077700	0	0	0	0
PRJNA204963	SRR866905	Apis mellifera	single end	2015	NA	2013	China	Asia	5968823	0	0	0	0
PRJNA205104	SRR867198	Apis mellifera	single end	2015	2012	2012	China	Asia	12000000	0	0	0	0
PRJNA205104	SRR867202	Apis mellifera	single end	2015	2012	2012	China	Asia	12000000	0	0	0	0
PRJNA205329	SRR868713	Apis mellifera	single end	2015	NA	2013	NA	NA	5999932	0	0	0	0
PRJNA205329	SRR868714	Apis mellifera	single end	2015	NA	2013	NA	NA	6090470	0	0	0	0
PRJNA205329	SRR868715	Apis mellifera	single end	2015	NA	2013	NA	NA	6090470	0	0	0	0
PRJNA528102	SRR8754003	Apis mellifera	single end	2019	2015	2015	USA	North America	72815162	3593	0	28	0
PRJNA528102	SRR8754004	Apis mellifera	single end	2019	2015	2015	USA	North America	63979027	88577	0.14	336	0
PRJNA528102	SRR8754005	Apis mellifera	single end	2019	2015	2015	USA	North America	71942514	3169	0	15	0
PRJNA528102	SRR8754006	Apis mellifera	single end	2019	2015	2015	USA	North America	53645693	3244	0.01	16	0
PRJNA528102	SRR8754007	Apis mellifera	single end	2019	2015	2015	USA	North America	56405749	3801	0.01	18	0
PRJNA528102	SRR8754008	Apis mellifera	single end	2019	2015	2015	USA	North America	77241988	10458	0.01	40	0
PRJNA528102	SRR8754009	Apis mellifera	single end	2019	2015	2015	USA	North America	94642154	3832	0	15	0
PRJNA528102	SRR8754010	Apis mellifera	single end	2019	2015	2015	USA	North America	48938453	2689	0.01	7	0
PRJNA528102	SRR8754011	Apis mellifera	single end	2019	2015	2015	USA	North America	41309867	15595	0.04	50	0
PRJNA528102	SRR8754012	Apis mellifera	single end	2019	2015	2015	USA	North America	69898738	3626391	5.19	14438	0.02
PRJNA528102	SRR8754013	Apis mellifera	single end	2019	2015	2015	USA	North America	59092018	3221	0.01	4	0
PRJNA528102	SRR8754014	Apis mellifera	single end	2019	2015	2015	USA	North America	67128679	4666	0.01	23	0
PRJNA528102	SRR8754015	Apis mellifera	single end	2019	2015	2015	USA	North America	65478974	3834	0.01	21	0
PRJNA528102	SRR8754016	Apis mellifera	single end	2019	2015	2015	USA	North America	64490416	2844	0	18	0
PRJNA528102	SRR8754017	Apis mellifera	single end	2019	2015	2015	USA	North America	74797382	4966	0.01	16	0

PRJNA528102	SRR8754018	Apis mellifera	single end	2019	2015	2015	USA	North America	53047267	2890	0.01	10	0
PRJNA528102	SRR8754019	Apis mellifera	single end	2019	2015	2015	USA	North America	90816956	23581978	25.97	93646	0.1
PRJNA528102	SRR8754020	Apis mellifera	single end	2019	2015	2015	USA	North America	58415925	2951	0.01	11	0
PRJNA533756	SRR9705226	Apis mellifera	single end	2019	2016	2016	Japan	Asia	15486411	2673	0.02	204	0
PRJNA533756	SRR9705227	Apis mellifera	single end	2019	2016	2016	Japan	Asia	19061256	596	0	53	0
PRJNA533756	SRR9705228	Apis mellifera	single end	2019	2016	2016	Japan	Asia	24164968	1432	0.01	116	0
PRJNA533756	SRR9705229	Apis mellifera	single end	2019	2016	2016	Japan	Asia	18570697	452	0	49	0
PRJNA533756	SRR9705230	Apis mellifera	single end	2019	2016	2016	Japan	Asia	10416804	1344	0.01	232	0
PRJNA533756	SRR9705231	Apis mellifera	single end	2019	2016	2016	Japan	Asia	11365034	999	0.01	129	0
PRJNA533756	SRR9705232	Apis mellifera	single end	2019	2016	2016	Japan	Asia	9333900	1271	0.01	257	0
PRJNA533756	SRR9705233	Apis mellifera	single end	2019	2016	2016	Japan	Asia	17293918	1787	0.01	328	0
PRJNA533756	SRR9705234	Apis mellifera	single end	2019	2016	2016	Japan	Asia	19729923	193	0	25	0
PRJNA533756	SRR9705235	Apis mellifera	single end	2019	2016	2016	Japan	Asia	16406968	942	0.01	149	0
PRJNA533756	SRR9705236	Apis mellifera	single end	2019	2016	2016	Japan	Asia	14039971	2135	0.02	365	0
PRJNA533756	SRR9705237	Apis mellifera	single end	2019	2016	2016	Japan	Asia	15783175	1712	0.01	309	0
PRJNA533756	SRR9705238	Apis mellifera	single end	2019	2016	2016	Japan	Asia	17808966	3490	0.02	311	0
PRJNA533756	SRR9705239	Apis mellifera	single end	2019	2016	2016	Japan	Asia	14285040	3466	0.02	438	0
PRJNA533756	SRR9705240	Apis mellifera	single end	2019	2016	2016	Japan	Asia	9064418	1813	0.02	325	0
PRJNA533756	SRR9705241	Apis mellifera	single end	2019	2016	2016	Japan	Asia	10936575	1586	0.01	237	0
PRJNA533756	SRR9705242	Apis mellifera	single end	2019	2016	2016	Japan	Asia	17124324	280	0	17	0
PRJNA533756	SRR9705243	Apis mellifera	single end	2019	2016	2016	Japan	Asia	16010731	789	0	88	0
PRJNA533756	SRR9705244	Apis mellifera	single end	2019	2016	2016	Japan	Asia	14292033	1595	0.01	184	0
PRJNA533756	SRR9705245	Apis mellifera	single end	2019	2016	2016	Japan	Asia	18148190	867	0	119	0
PRJNA533756	SRR9705246	Apis mellifera	single end	2019	2016	2016	Japan	Asia	9661733	2196	0.02	347	0
PRJNA533756	SRR9705247	Apis mellifera	single end	2019	2016	2016	Japan	Asia	14314346	3421	0.02	469	0
PRJNA533756	SRR9705248	Apis mellifera	single end	2019	2016	2016	Japan	Asia	16036487	197	0	31	0
PRJNA533756	SRR9705249	Apis mellifera	single end	2019	2016	2016	Japan	Asia	14647016	6906	0.05	687	0
PRJNA533756	SRR9705250	Apis mellifera	single end	2019	2016	2016	Japan	Asia	27808197	159	0	29	0
PRJNA533756	SRR9705251	Apis mellifera	single end	2019	2016	2016	Japan	Asia	21800855	901	0	84	0
PRJNA533756	SRR9705252	Apis mellifera	single end	2019	2016	2016	Japan	Asia	10890306	823	0.01	120	0
PRJNA533756	SRR9705253	Apis mellifera	single end	2019	2016	2016	Japan	Asia	9167335	7	0	2	0
PRJNA533756	SRR9705254	Apis mellifera	single end	2019	2016	2016	Japan	Asia	20643996	4170	0.02	317	0
PRJNA533756	SRR9705255	Apis mellifera	single end	2019	2016	2016	Japan	Asia	14029384	74	0	10	0
PRJNA533756	SRR9705256	Apis mellifera	single end	2019	2016	2016	Japan	Asia	13007755	1603	0.01	263	0
PRJNA533756	SRR9705257	Apis mellifera	single end	2019	2016	2016	Japan	Asia	19661861	5690	0.03	644	0
PRJNA533756	SRR9705258	Apis mellifera	single end	2019	2016	2016	Japan	Asia	15544593	1264	0.01	208	0
PRJNA533756	SRR9705259	Apis mellifera	single end	2019	2016	2016	Japan	Asia	17570463	6098	0.03	636	0
PRJNA533756	SRR9705260	Apis mellifera	single end	2019	2016	2016	Japan	Asia	19589050	819	0	85	0
PRJNA533756	SRR9705261	Apis mellifera	single end	2019	2016	2016	Japan	Asia	17704182	171	0	32	0
PRJNA533756	SRR9705262	Apis mellifera	single end	2019	2016	2016	Japan	Asia	14740052	6097	0.04	641	0
PRJNA533756	SRR9705263	Apis mellifera	single end	2019	2016	2016	Japan	Asia	18298556	7608	0.04	794	0
PRJNA533756	SRR9705264	Apis mellifera	single end	2019	2016	2016	Japan	Asia	18787150	5771	0.03	587	0
PRJNA533756	SRR9705265	Apis mellifera	single end	2019	2016	2016	Japan	Asia	12991441	2549	0.02	192	0
PRJNA533756	SRR9705266	Apis mellifera	single end	2019	2016	2016	Japan	Asia	25765138	2748	0.01	209	0
PRJNA533756	SRR9705267	Apis mellifera	single end	2019	2016	2016	Japan	Asia	16185159	2914788	18.01	483309	2.99
PRJNA533756	SRR9705268	Apis mellifera	single end	2019	2016	2016	Japan	Asia	16654120	3112017	18.69	588743	3.54
PRJNA533756	SRR9705269	Apis mellifera	single end	2019	2016	2016	Japan	Asia	15268086	3743058	24.52	713167	4.67
PRJNA533756	SRR9705270	Apis mellifera	single end	2019	2016	2016	Japan	Asia	16545887	3802769	22.98	704270	4.26
PRJNA533756	SRR9705271	Apis mellifera	single end	2019	2016	2016	Japan	Asia	13146986	3509636	26.7	468846	3.57
PRJNA533756	SRR9705272	Apis mellifera	single end	2019	2016	2016	Japan	Asia	15255349	519383	3.4	95135	0.62
PRJNA533756	SRR9705273	Apis mellifera	single end	2019	2016	2016	Japan	Asia	15241937	638310	4.19	101015	0.66
PRJNA533756	SRR9705274	Apis mellifera	single end	2019	2016	2016	Japan	Asia	18507565	8824795	47.68	634361	3.43
PRJNA533756	SRR9705275	Apis mellifera	single end	2019	2016	2016	Japan	Asia	18081128	544815	3.01	122450	0.68
PRJNA533756	SRR9705276	Apis mellifera	single end	2019	2016	2016	Japan	Asia	9524757	3034640	31.86	290782	3.05
PRJNA533756	SRR9705277	Apis mellifera	single end	2019	2016	2016	Japan	Asia	12949070	8364	0.06	751	0.01
PRJNA533756	SRR9705278	Apis mellifera	single end	2019	2016	2016	Japan	Asia	14797182	175613	1.19	25068	0.17
PRJNA533756	SRR9705279	Apis mellifera	single end	2019	2016	2016	Japan	Asia	16299022	2229210	13.68	282308	1.73
PRJNA533756	SRR9705280	Apis mellifera	single end	2019	2016	2016	Japan	Asia	13468117	522	0	65	0
PRJNA533756	SRR9705281	Apis mellifera	single end	2019	2016	2016	Japan	Asia	17006643	97516	0.57	16713	0.1
PRJNA533756	SRR9705282	Apis mellifera	single end	2019	2016	2016	Japan	Asia	13217665	1285755	9.73	155826	1.18
PRJNA533756	SRR9705283	Apis mellifera	single end	2019	2016	2016	Japan	Asia	13644886	5991	0.04	689	0.01
PRJNA533756	SRR9705284	Apis mellifera	single end	2019	2016	2016	Japan	Asia	19846028	1285	0.01	246	0
PRJNA533756	SRR9705285	Apis mellifera	single end	2019	2016	2016	Japan	Asia	18126737	1862	0.01	299	0
PRJNA533756	SRR9705286	Apis mellifera	single end	2019	2016	2016	Japan	Asia	9833589	1929	0.02	204	0
PRJNA533756	SRR9705287	Apis mellifera	single end	2019	2016	2016	Japan	Asia	20463031	2576	0.01	439	0
PRJNA533756	SRR9705288	Apis mellifera	single end	2019	2016	2016	Japan	Asia	19867944	1979	0.01	265	0
PRJNA533756	SRR9705289	Apis mellifera	single end	2019	2016	2016	Japan	Asia	21583506	773	0	81	0
PRJNA533756	SRR9705290	Apis mellifera	single end	2019	2016	2016	Japan	Asia	18186520	1965154	10.81	153299	0.84
PRJNA533756	SRR9705291	Apis mellifera	single end	2019	2016	2016	Japan	Asia	19105467	692	0	85	0
PRJNA533756	SRR9705292	Apis mellifera	single end	2019	2016	2016	Japan	Asia	22567200	81	0	9	0
PRJNA533756	SRR9705293	Apis mellifera	single end	2019	2016	2016	Japan	Asia	16611456	195	0	31	0
PRJNA533756	SRR9705294	Apis mellifera	single end	2019	2016	2016	Japan	Asia	14602711	979	0.01	164	0
PRJNA533756	SRR9705295	Apis mellifera	single end	2019	2016	2016	Japan	Asia	20450423	215	0	42	0
PRJNA533756	SRR9705296	Apis mellifera	single end	2019	2016	2016	Japan	Asia	15847281	820	0.01	79	0
PRJNA533756	SRR9705297	Apis mellifera	single end	2019	2016	2016	Japan	Asia	19970892	78	0	13	0
PRJNA533756	SRR9705298	Apis mellifera	single end	2019	2016	2016	Japan	Asia	20470983	744	0	104	0
PRJNA533756	SRR9705299	Apis mellifera	single end	2019	2016	2016	Japan	Asia	19971631	1329	0.01	274	0
PRJNA533756	SRR9705300	Apis mellifera	single end	2019	2016	2016	Japan	Asia	17354004	1027	0.01	136	0
PRJNA533756	SRR9705301	Apis mellifera	single end	2019	2016	2016	Japan	Asia	21827705	719	0	70	0
PRJNA533756	SRR9705302	Apis mellifera	single end	2019	2016	2016	Japan	Asia	15331276	216	0	18	0
PRJNA533756	SRR9705303	Apis mellifera	single end	2019	2016	2016	Japan	Asia	15989008	1113	0.01	166	0
PRJNA533756	SRR9705304	Apis mellifera	single end	2019	2016	2016	Japan	Asia	10118280	1484	0.01	265	0
PRJNA533756	SRR9705305	Apis mellifera	single end	2019	2016	2016	Japan	Asia	16880583	3	0	1	0
PRJNA533756	SRR9705306	Apis mellifera	single end	2019	2016	2016	Japan	Asia	18639647	1076	0.01	146	0
PRJNA533756	SRR9705307	Apis mellifera	single end	2019	2016	2016	Japan	Asia	20556795	738877	3.59	61773	0.3
PRJNA533756	SRR9705308	Apis mellifera	single end	2019	2016	2016	Japan	Asia	16754022	380	0	48	0
PRJNA533756	SRR9705309	Apis mellifera	single end	2019	2016	2016	Japan	Asia	17077488	1328234	7.78	140867	0.82
PRJNA533756	SRR9705310	Apis mellifera	single end	2019	2016	2016	Japan	Asia	17246845	1306	0.01	240	0
PRJNA533756	SRR9705311	Apis mellifera	single end	2019	2016	2016	Japan	Asia	19929764	1022	0.01	167	0
PRJNA533756	SRR9705312	Apis mellifera	single end	2019	2016	2016	Japan	Asia	16974357	39	0	9	0
PRJNA219484	SRR989674	Apis mellifera	single end	2015	NA	2013	NA	NA	5386166	1992542	36.99	593	0.01
PRJNA219484	SRR989675	Apis mellifera	single end	2015	NA	2013	NA	NA	3444202	3	0	0	0
PRJNA219484	SRR989676	Apis mellifera	single end	2015	NA	2013	NA	NA	5810514	2595130	44.66	266	0
PRJNA219484	SRR989677	Apis mellifera	single end	2015	NA	2013	NA	NA	11120630	3455	0.03	0	0
PRJDB6538	DRR152975	Apis mellifera	paired end	2018	NA	2016	NA	NA	27602518	0	0	2	0
PRJDB6538	DRR152976	Apis mellifera	paired end	2018	NA	2016	NA	NA	28308036	0	0	4	0
PRJDB6538	DRR152977	Apis mellifera	paired end	2018	NA	2016	NA	NA	31416414	0	0	0	0
PRJDB6538	DRR152978	Apis mellifera	paired end	2018	NA	2016	NA	NA	26907050	2	0	0	0
PRJDB6538	DRR152979	Apis mellifera	paired end	2018	NA	2016	NA	NA	27929356	0	0	3	0
PRJDB6538	DRR152980	Apis mellifera	paired end	2018	NA	2016	NA	NA	32120980	0	0	0	0
PRJEB26475	ERR2540051	Apis mellifera	paired end	2018	NA	2016	NA	NA	61933608	89600	0.14	7979466	12.88
PRJEB26475	ERR2540052	Apis mellifera	paired end	2018	NA	2016	NA	NA	63280202	423434	0.67	7748300	12.24
PRJEB26475	ERR2540053	Apis mellifera	paired end	2018	NA	2016	NA	NA	70681312	359212	0.51	3336460	4.72
PRJEB35177	ERR3632874	Apis mellifera	paired end	2020	NA	2018	NA	NA	19287544	0	0	0	0
PRJEB35177	ERR3632875	Apis mellifera	paired end	2020	NA	2018	NA	NA	14808496	0	0	0	0
PRJEB35177	ERR3632876	Apis mellifera	paired end	2020	NA	2018	NA	NA	21762378	18	0	0	0

PRJEB35177	ERR3632877	Apis mellifera	paired end	2020	NA	2018	NA	NA	18647232	0	0	0	0
PRJEB35177	ERR3632878	Apis mellifera	paired end	2020	NA	2018	NA	NA	14258288	0	0	1056	0.01
PRJEB35177	ERR3632879	Apis mellifera	paired end	2020	NA	2018	NA	NA	17980672	48	0	4	0
PRJEB35177	ERR3632880	Apis mellifera	paired end	2020	NA	2018	NA	NA	19762434	0	0	0	0
PRJEB35177	ERR3632881	Apis mellifera	paired end	2020	NA	2018	NA	NA	16326394	0	0	0	0
PRJEB35177	ERR3632882	Apis mellifera	paired end	2020	NA	2018	NA	NA	16516580	0	0	0	0
PRJEB35177	ERR3632883	Apis mellifera	paired end	2020	NA	2018	NA	NA	23348800	0	0	0	0
PRJEB35177	ERR3632884	Apis mellifera	paired end	2020	NA	2018	NA	NA	21523968	0	0	0	0
PRJEB35177	ERR3632885	Apis mellifera	paired end	2020	NA	2018	NA	NA	18276218	0	0	0	0
PRJEB35177	ERR3632886	Apis mellifera	paired end	2020	NA	2018	NA	NA	16118430	0	0	0	0
PRJEB35177	ERR3632887	Apis mellifera	paired end	2020	NA	2018	NA	NA	15211364	0	0	0	0
PRJNA564247	SRR10078309	Apis mellifera	paired end	2020	NA	2018	NA	NA	64846592	0	0	0	0
PRJNA564247	SRR10078310	Apis mellifera	paired end	2020	NA	2018	NA	NA	56571686	0	0	0	0
PRJNA564247	SRR10078311	Apis mellifera	paired end	2020	NA	2018	NA	NA	80281760	0	0	0	0
PRJNA564247	SRR10078312	Apis mellifera	paired end	2020	NA	2018	NA	NA	56556090	0	0	0	0
PRJNA564247	SRR10078313	Apis mellifera	paired end	2020	NA	2018	NA	NA	64968842	0	0	0	0
PRJNA564247	SRR10078314	Apis mellifera	paired end	2020	NA	2018	NA	NA	82956694	0	0	0	0
PRJNA564247	SRR10078315	Apis mellifera	paired end	2020	NA	2018	NA	NA	82016568	0	0	0	0
PRJNA564247	SRR10078316	Apis mellifera	paired end	2020	NA	2018	NA	NA	76021434	0	0	0	0
PRJNA564247	SRR10078317	Apis mellifera	paired end	2020	NA	2018	NA	NA	72417278	2	0	0	0
PRJNA564247	SRR10078318	Apis mellifera	paired end	2020	NA	2018	NA	NA	65545512	0	0	0	0
PRJNA575879	SRR10231776	Apis mellifera	paired end	2019	NA	2017	USA	North America	16694920	3829760	22.94	1563	0.01
PRJNA575879	SRR10231787	Apis mellifera	paired end	2019	NA	2017	USA	North America	17586768	4033714	22.94	1537	0.01
PRJNA575879	SRR10231798	Apis mellifera	paired end	2019	NA	2017	USA	North America	15448916	48	0	648	0
PRJNA575879	SRR10231809	Apis mellifera	paired end	2019	NA	2017	USA	North America	16064920	29	0	334	0
PRJNA575879	SRR10231820	Apis mellifera	paired end	2019	NA	2017	USA	North America	15061152	69	0	855	0.01
PRJNA575879	SRR10231823	Apis mellifera	paired end	2019	NA	2017	USA	North America	18381088	1543	0.01	4186	0.02
PRJNA575879	SRR10231824	Apis mellifera	paired end	2019	NA	2017	USA	North America	18952582	1122	0.01	3608	0.02
PRJNA575879	SRR10231825	Apis mellifera	paired end	2019	NA	2017	USA	North America	17978628	2176	0.01	6141	0.03
PRJNA575879	SRR10231826	Apis mellifera	paired end	2019	NA	2017	USA	North America	18746926	1433	0.01	4214	0.02
PRJNA575879	SRR10231827	Apis mellifera	paired end	2019	NA	2017	USA	North America	18817368	6601	0.04	1637	0.01
PRJNA575879	SRR10231828	Apis mellifera	paired end	2019	NA	2017	USA	North America	19455452	6654	0.03	915	0
PRJNA575879	SRR10231829	Apis mellifera	paired end	2019	NA	2017	USA	North America	18402286	6740	0.04	2235	0.01
PRJNA575879	SRR10231830	Apis mellifera	paired end	2019	NA	2017	USA	North America	19240830	6490	0.03	1168	0.01
PRJNA575879	SRR10231831	Apis mellifera	paired end	2019	NA	2017	USA	North America	15844380	44	0	507	0
PRJNA575879	SRR10231832	Apis mellifera	paired end	2019	NA	2017	USA	North America	16025180	566	0	2192	0.01
PRJNA575879	SRR10231833	Apis mellifera	paired end	2019	NA	2017	USA	North America	16537662	411	0	1685	0.01
PRJNA575879	SRR10231834	Apis mellifera	paired end	2019	NA	2017	USA	North America	15685398	831	0.01	2790	0.02
PRJNA575879	SRR10231835	Apis mellifera	paired end	2019	NA	2017	USA	North America	16340458	595	0	2084	0.01
PRJNA575879	SRR10231836	Apis mellifera	paired end	2019	NA	2017	USA	North America	20783994	2903	0.01	5289	0.03
PRJNA575879	SRR10231837	Apis mellifera	paired end	2019	NA	2017	USA	North America	21599012	2599	0.01	5350	0.02
PRJNA575879	SRR10231838	Apis mellifera	paired end	2019	NA	2017	USA	North America	20281062	3742	0.02	6037	0.03
PRJNA575879	SRR10231839	Apis mellifera	paired end	2019	NA	2017	USA	North America	21345106	2570	0.01	5459	0.03
PRJNA575879	SRR10231840	Apis mellifera	paired end	2019	NA	2017	USA	North America	18128986	2094	0.01	31292	0.17
PRJNA575879	SRR10231841	Apis mellifera	paired end	2019	NA	2017	USA	North America	18951098	2409	0.01	36475	0.19
PRJNA575879	SRR10231842	Apis mellifera	paired end	2019	NA	2017	USA	North America	15522864	16096	0.1	1884466	12.14
PRJNA575879	SRR10231843	Apis mellifera	paired end	2019	NA	2017	USA	North America	17652400	2078	0.01	31026	0.18
PRJNA575879	SRR10231844	Apis mellifera	paired end	2019	NA	2017	USA	North America	18680394	2114	0.01	33863	0.18
PRJNA575879	SRR10231845	Apis mellifera	paired end	2019	NA	2017	USA	North America	16748274	104	0	2317	0.01
PRJNA575879	SRR10231846	Apis mellifera	paired end	2019	NA	2017	USA	North America	17404484	94	0	1581	0.01
PRJNA575879	SRR10231847	Apis mellifera	paired end	2019	NA	2017	USA	North America	16325936	114	0	3369	0.02
PRJNA575879	SRR10231848	Apis mellifera	paired end	2019	NA	2017	USA	North America	17180312	122	0	1927	0.01
PRJNA575879	SRR10231849	Apis mellifera	paired end	2019	NA	2017	USA	North America	13300192	3876254	29.14	7370940	55.42
PRJNA575879	SRR10231850	Apis mellifera	paired end	2019	NA	2017	USA	North America	13641982	3976360	29.15	7570660	55.5
PRJNA575879	SRR10231851	Apis mellifera	paired end	2019	NA	2017	USA	North America	13002764	3787080	29.13	7199220	55.37
PRJNA575879	SRR10231852	Apis mellifera	paired end	2019	NA	2017	USA	North America	13484998	3924860	29.11	7478980	55.46
PRJNA575879	SRR10231853	Apis mellifera	paired end	2019	NA	2017	USA	North America	16177306	15989	0.1	1968892	12.17
PRJNA575879	SRR10231854	Apis mellifera	paired end	2019	NA	2017	USA	North America	17204424	8646	0.05	4252040	24.71
PRJNA575879	SRR10231855	Apis mellifera	paired end	2019	NA	2017	USA	North America	17764458	8645	0.05	4391320	24.72
PRJNA575879	SRR10231856	Apis mellifera	paired end	2019	NA	2017	USA	North America	16787430	8238	0.05	4147660	24.71
PRJNA575879	SRR10231857	Apis mellifera	paired end	2019	NA	2017	USA	North America	17559002	8595	0.05	4333696	24.68
PRJNA575879	SRR10231858	Apis mellifera	paired end	2019	NA	2017	USA	North America	14210014	22620	0.16	8872980	62.44
PRJNA575879	SRR10231859	Apis mellifera	paired end	2019	NA	2017	USA	North America	14639796	26385	0.18	9136640	62.41
PRJNA575879	SRR10231860	Apis mellifera	paired end	2019	NA	2017	USA	North America	13884640	23754	0.17	8644578	62.26
PRJNA575879	SRR10231861	Apis mellifera	paired end	2019	NA	2017	USA	North America	14480222	25284	0.17	9031140	62.37
PRJNA575879	SRR10231862	Apis mellifera	paired end	2019	NA	2017	USA	North America	17136568	3939960	22.99	1478	0.01
PRJNA575879	SRR10231863	Apis mellifera	paired end	2019	NA	2017	USA	North America	17801904	4090900	22.98	1555	0.01
PRJNA575879	SRR10231864	Apis mellifera	paired end	2019	NA	2017	USA	North America	15102388	15820	0.1	1836206	12.16
PRJNA575879	SRR10231865	Apis mellifera	paired end	2019	NA	2017	USA	North America	15973522	15948	0.1	1941292	12.15
PRJNA587756	SRR10397460	Apis mellifera	paired end	2020	2018	2018	Argentina	South America	43968980	0	0	0	0
PRJNA587756	SRR10397461	Apis mellifera	paired end	2020	2018	2018	Argentina	South America	44497750	0	0	0	0
PRJNA587756	SRR10397462	Apis mellifera	paired end	2020	2018	2018	Argentina	South America	48663970	0	0	0	0
PRJNA587756	SRR10397463	Apis mellifera	paired end	2020	2018	2018	Argentina	South America	46433414	0	0	0	0
PRJNA587756	SRR10397464	Apis mellifera	paired end	2020	2018	2018	Argentina	South America	44856508	0	0	0	0
PRJNA587756	SRR10397465	Apis mellifera	paired end	2020	2018	2018	Argentina	South America	52816588	0	0	0	0
PRJNA601517	SRR10901285	Apis mellifera	paired end	2021	2013	2013	USA	North America	129474798	71	0	4	0
PRJNA601517	SRR10901286	Apis mellifera	paired end	2021	2013	2013	USA	North America	28205978	18	0	2	0
PRJNA601517	SRR10901287	Apis mellifera	paired end	2021	2013	2013	USA	North America	9381356	0	0	0	0
PRJNA601517	SRR10901288	Apis mellifera	paired end	2021	2013	2013	USA	North America	18124744	4	0	0	0
PRJNA601517	SRR10901289	Apis mellifera	paired end	2021	2013	2013	USA	North America	25663268	0	0	0	0
PRJNA601517	SRR10901290	Apis mellifera	paired end	2021	2013	2013	USA	North America	25453714	30	0	0	0
PRJNA601517	SRR10901291	Apis mellifera	paired end	2021	2013	2013	USA	North America	26050876	0	0	0	0
PRJNA601517	SRR10901292	Apis mellifera	paired end	2021	2013	2013	USA	North America	27291634	2	0	0	0
PRJNA601517	SRR10901293	Apis mellifera	paired end	2021	2013	2013	USA	North America	37320976	16	0	8	0
PRJNA601517	SRR10901294	Apis mellifera	paired end	2021	2013	2013	USA	North America	28481710	0	0	0	0
PRJNA601517	SRR10901295	Apis mellifera	paired end	2021	2013	2013	USA	North America	26106168	0	0	0	0
PRJNA601517	SRR10901296	Apis mellifera	paired end	2021	2013	2013	USA	North America	20240570	12	0	0	0
PRJNA601517	SRR10901297	Apis mellifera	paired end	2021	2013	2013	USA	North America	21654688	0	0	0	0
PRJNA601517	SRR10901298	Apis mellifera	paired end	2021	2013	2013	USA	North America	29363428	0	0	0	0
PRJNA601517	SRR10901299	Apis mellifera	paired end	2021	2013	2013	USA	North America	23795162	0	0	0	0
PRJNA601517	SRR10901300	Apis mellifera	paired end	2021	2013	2013	USA	North America	25814386	0	0	0	0
PRJNA601517	SRR10901301	Apis mellifera	paired end	2021	2013	2013	USA	North America	27015926	4	0	0	0
PRJNA601517	SRR10901302	Apis mellifera	paired end	2021	2013	2013	USA	North America	24371408	18	0	2	0
PRJNA601517	SRR10901303	Apis mellifera	paired end	2021	2013	2013	USA	North America	130931962	10	0	2	0
PRJNA601517	SRR10901304	Apis mellifera	paired end	2021	2013	2013	USA	North America	22798190	2	0	0	0
PRJNA601517	SRR10901305	Apis mellifera	paired end	2021	2013	2013	USA	North America	25161370	8	0	0	0
PRJNA601517	SRR10901306	Apis mellifera	paired end	2021	2013	2013	USA	North America	32795470	2	0	0	0
PRJNA601517	SRR10901307	Apis mellifera	paired end	2021	2013	2013	USA	North America	23653120	0	0	0	0
PRJNA601517	SRR10901308	Apis mellifera	paired end	2021	2013	2013	USA	North America	19306370	0	0	0	0
PRJNA602047	SRR10912937	Apis mellifera	paired end	2020	NA	2018	NA	NA	57727436	2	0	0	0
PRJNA602047	SRR10912938	Apis mellifera	paired end	2020	NA	2018	NA	NA	50897546	0	0	0	0
PRJNA602047	SRR10912939	Apis mellifera	paired end	2020	NA	2018	NA	NA	53963400	0	0	0	0
PRJNA602047	SRR10912940	Apis mellifera	paired end	2020	NA	2018	NA	NA	47351558	0	0	0	0
PRJNA602047	SRR10912941	Apis mellifera	paired end	2020	NA	2018	NA	NA	45510252	0	0	0	0
PRJNA602047	SRR10912942	Apis mellifera	paired end	2020	NA	2018	NA	NA	45070094	0	0	0	0
PRJNA602047	SRR10912943	Apis mellifera	paired end	2020	NA	2018	NA	NA	57354362	21	0	0	0

PRJNA602047	SRR10912944	Apis mellifera	paired end	2020	NA	2018	NA	NA	54047242	134	0	0	0
PRJNA602047	SRR10912945	Apis mellifera	paired end	2020	NA	2018	NA	NA	58876422	0	0	0	0
PRJNA613391	SRR11349374	Apis mellifera	paired end	2020	2017	2017	Australia	Oceania	71907920	0	0	0	0
PRJNA628981	SRR11634539	Apis mellifera	paired end	2021	2018	2018	UK	Europe	61343190	5044	0.01	91304	0.15
PRJNA628981	SRR11634540	Apis mellifera	paired end	2021	2018	2018	UK	Europe	52203232	1793	0	2821	0.01
PRJNA628981	SRR11634541	Apis mellifera	paired end	2021	2018	2018	UK	Europe	63255904	1155	0	29858	0.05
PRJNA628981	SRR11634542	Apis mellifera	paired end	2021	2018	2018	UK	Europe	69974472	237	0	549	0
PRJNA628981	SRR11634543	Apis mellifera	paired end	2021	2018	2018	UK	Europe	53742100	514	0	2479	0
PRJNA628981	SRR11634544	Apis mellifera	paired end	2021	2018	2018	UK	Europe	54341492	3550	0.01	2223	0
PRJNA628981	SRR11634545	Apis mellifera	paired end	2021	2018	2018	UK	Europe	65505056	6105	0.01	1922	0
PRJNA628981	SRR11634546	Apis mellifera	paired end	2021	2018	2018	UK	Europe	56016440	620	0	932	0
PRJNA628981	SRR11634547	Apis mellifera	paired end	2021	2018	2018	UK	Europe	66744350	7274	0.01	10833	0.02
PRJNA630027	SRR11671127	Apis mellifera	paired end	2020	NA	2018	NA	NA	82767632	150	0	8	0
PRJNA630027	SRR11671128	Apis mellifera	paired end	2020	NA	2018	NA	NA	79728078	786	0	511	0
PRJNA630027	SRR11671129	Apis mellifera	paired end	2020	NA	2018	NA	NA	98928126	37290	0.04	10	0
PRJNA630027	SRR11671130	Apis mellifera	paired end	2020	NA	2018	NA	NA	88630708	68	0	12	0
PRJNA630027	SRR11671131	Apis mellifera	paired end	2020	NA	2018	NA	NA	74555736	14107	0.02	16	0
PRJNA630027	SRR11671132	Apis mellifera	paired end	2020	NA	2018	NA	NA	85472672	178	0	38	0
PRJNA630027	SRR11671133	Apis mellifera	paired end	2020	NA	2018	NA	NA	71933302	4457	0.01	803	0
PRJNA630027	SRR11671134	Apis mellifera	paired end	2020	NA	2018	NA	NA	94430180	10273	0.01	224	0
PRJNA238833	SRR1182310	Apis mellifera	paired end	2014	NA	2012	NA	NA	51534456	4096060	7.95	50924	0.1
PRJNA238833	SRR1182312	Apis mellifera	paired end	2014	NA	2012	NA	NA	43564950	3894360	8.94	7109	0.02
PRJNA238833	SRR1182313	Apis mellifera	paired end	2014	NA	2012	NA	NA	75458732	10835	0.01	879	0
PRJNA240064	SRR1182528	Apis mellifera	paired end	2014	2011	2011	USA	North America	17205992	3562456	20.7	10089	0.06
PRJNA240064	SRR1182529	Apis mellifera	paired end	2014	2011	2011	USA	North America	21147498	2880876	13.62	9169	0.04
PRJNA240064	SRR1182578	Apis mellifera	paired end	2014	2011	2011	USA	North America	30871554	3024034	9.8	9008	0.03
PRJNA240064	SRR1182579	Apis mellifera	paired end	2014	2011	2011	USA	North America	21556600	4670396	21.67	12111	0.06
PRJNA240064	SRR1182580	Apis mellifera	paired end	2014	2011	2011	USA	North America	32012050	5587920	17.46	17042	0.05
PRJNA240064	SRR1182581	Apis mellifera	paired end	2014	2011	2011	USA	North America	16131564	2452148	15.2	6439	0.04
PRJNA240064	SRR1182637	Apis mellifera	paired end	2014	2011	2011	USA	North America	51410174	3283728	6.39	7725	0.02
PRJNA240064	SRR1182638	Apis mellifera	paired end	2014	2011	2011	USA	North America	19401608	3510290	18.09	8401	0.04
PRJNA240064	SRR1182639	Apis mellifera	paired end	2014	2011	2011	USA	North America	17642008	2911920	16.51	8315	0.05
PRJNA240064	SRR1182640	Apis mellifera	paired end	2014	2011	2011	USA	North America	22318092	1528260	6.85	3377	0.02
PRJNA240064	SRR1182641	Apis mellifera	paired end	2014	2011	2011	USA	North America	24240944	4591680	18.94	10799	0.04
PRJNA240064	SRR1182642	Apis mellifera	paired end	2014	2011	2011	USA	North America	16972664	4137820	24.38	10955	0.06
PRJNA635960	SRR11879879	Apis mellifera	paired end	2020	2014	2014	Solomon_Islands	Oceania	122615250	414	0	139	0
PRJNA635960	SRR11879880	Apis mellifera	paired end	2020	2014	2014	Papua_New_Guinea	Oceania	104622470	0	0	14	0
PRJNA636804	SRR11940309	Apis mellifera	paired end	2020	2018	2018	Germany	Europe	206457564	0	0	0	0
PRJNA636804	SRR11940310	Apis mellifera	paired end	2020	2018	2018	Germany	Europe	214681890	0	0	0	0
PRJNA636804	SRR11940311	Apis mellifera	paired end	2020	2018	2018	Germany	Europe	218349306	0	0	0	0
PRJNA639933	SRR12031953	Apis mellifera	paired end	2020	2019	2019	China	Asia	43346592	0	0	0	0
PRJNA639933	SRR12031954	Apis mellifera	paired end	2020	2019	2019	China	Asia	41601872	0	0	0	0
PRJNA639933	SRR12031955	Apis mellifera	paired end	2020	2019	2019	China	Asia	44479454	0	0	0	0
PRJNA639933	SRR12031956	Apis mellifera	paired end	2020	2019	2019	China	Asia	74266086	0	0	0	0
PRJNA639933	SRR12031957	Apis mellifera	paired end	2020	2019	2019	China	Asia	63055150	0	0	0	0
PRJNA639933	SRR12031958	Apis mellifera	paired end	2020	2019	2019	China	Asia	79322368	0	0	0	0
PRJNA639933	SRR12031959	Apis mellifera	paired end	2020	2019	2019	China	Asia	73968648	0	0	0	0
PRJNA639933	SRR12031960	Apis mellifera	paired end	2020	2019	2019	China	Asia	69434358	0	0	0	0
PRJNA639933	SRR12031961	Apis mellifera	paired end	2020	2019	2019	China	Asia	44663500	0	0	0	0
PRJNA639933	SRR12031962	Apis mellifera	paired end	2020	2019	2019	China	Asia	50303700	0	0	0	0
PRJNA639933	SRR12031963	Apis mellifera	paired end	2020	2019	2019	China	Asia	42710574	0	0	0	0
PRJNA639933	SRR12031964	Apis mellifera	paired end	2020	2019	2019	China	Asia	49704788	0	0	0	0
PRJNA639933	SRR12031965	Apis mellifera	paired end	2020	2019	2019	China	Asia	59051322	0	0	0	0
PRJNA639933	SRR12031966	Apis mellifera	paired end	2020	2019	2019	China	Asia	51542312	0	0	0	0
PRJNA639933	SRR12031967	Apis mellifera	paired end	2020	2019	2019	China	Asia	41238792	0	0	0	0
PRJNA639933	SRR12031968	Apis mellifera	paired end	2020	2019	2019	China	Asia	45676858	0	0	0	0
PRJNA639933	SRR12031969	Apis mellifera	paired end	2020	2019	2019	China	Asia	97593694	0	0	0	0
PRJNA639933	SRR12031970	Apis mellifera	paired end	2020	2019	2019	China	Asia	58203126	0	0	0	0
PRJNA243651	SRR1239302	Apis mellifera	paired end	2014	NA	2012	USA	North America	64110330	27823164	43.4	2185880	3.41
PRJNA243651	SRR1239303	Apis mellifera	paired end	2014	NA	2012	USA	North America	73361990	852720	1.16	1685	0
PRJNA243651	SRR1239304	Apis mellifera	paired end	2014	NA	2012	USA	North America	68187072	1606720	2.36	2751	0
PRJNA243651	SRR1239305	Apis mellifera	paired end	2014	NA	2012	USA	North America	68058182	1109286	1.63	2148	0
PRJNA243651	SRR1239306	Apis mellifera	paired end	2014	NA	2012	USA	North America	67841628	1551236	2.29	2399	0
PRJNA243651	SRR1239307	Apis mellifera	paired end	2014	NA	2012	USA	North America	78770592	22073000	28.02	19045	0.02
PRJNA243651	SRR1239308	Apis mellifera	paired end	2014	NA	2012	USA	North America	42519996	8189	0.02	774	0
PRJNA243651	SRR1239309	Apis mellifera	paired end	2014	NA	2012	USA	North America	45329928	13898	0.03	509	0
PRJNA243651	SRR1239310	Apis mellifera	paired end	2014	NA	2012	USA	North America	54545576	7454	0.01	525	0
PRJNA243651	SRR1239311	Apis mellifera	paired end	2014	NA	2012	USA	North America	51534456	4096060	7.95	50924	0.1
PRJNA243651	SRR1239312	Apis mellifera	paired end	2014	NA	2012	USA	North America	43564950	3894360	8.94	7109	0.02
PRJNA243651	SRR1239313	Apis mellifera	paired end	2014	NA	2012	USA	North America	75458732	10835	0.01	879	0
PRJNA656275	SRR12424195	Apis mellifera	paired end	2021	NA	2019	USA	North America	26824402	2160	0.01	44	0
PRJNA656275	SRR12424196	Apis mellifera	paired end	2021	NA	2019	USA	North America	27295078	1957	0.01	56	0
PRJNA656275	SRR12424197	Apis mellifera	paired end	2021	NA	2019	USA	North America	31239038	4013	0.01	118	0
PRJNA656275	SRR12424198	Apis mellifera	paired end	2021	NA	2019	USA	North America	31927636	4199	0.01	93	0
PRJNA656275	SRR12424199	Apis mellifera	paired end	2021	NA	2019	USA	North America	25491530	25421	0.1	801	0
PRJNA656275	SRR12424200	Apis mellifera	paired end	2021	NA	2019	USA	North America	25990288	25709	0.1	832	0
PRJNA656275	SRR12424201	Apis mellifera	paired end	2021	NA	2019	USA	North America	31674260	1419	0	38	0
PRJNA656275	SRR12424202	Apis mellifera	paired end	2021	NA	2019	USA	North America	32186808	1371	0	36	0
PRJNA656275	SRR12424203	Apis mellifera	paired end	2021	NA	2019	USA	North America	28592338	1024	0	14	0
PRJNA656275	SRR12424204	Apis mellifera	paired end	2021	NA	2019	USA	North America	29158804	1021	0	29	0
PRJNA656275	SRR12424205	Apis mellifera	paired end	2021	NA	2019	USA	North America	26534786	1611	0.01	29	0
PRJNA656275	SRR12424206	Apis mellifera	paired end	2021	NA	2019	USA	North America	27108340	1577	0.01	28	0
PRJNA656275	SRR12424207	Apis mellifera	paired end	2021	NA	2019	USA	North America	30517992	1401	0	25	0
PRJNA656275	SRR12424208	Apis mellifera	paired end	2021	NA	2019	USA	North America	31328464	1457	0	45	0
PRJNA656275	SRR12424209	Apis mellifera	paired end	2021	NA	2019	USA	North America	28068980	1787	0.01	58	0
PRJNA656275	SRR12424210	Apis mellifera	paired end	2021	NA	2019	USA	North America	28491660	1734	0.01	44	0
PRJNA656275	SRR12424211	Apis mellifera	paired end	2021	NA	2019	USA	North America	27878866	1733	0.01	59	0
PRJNA656275	SRR12424212	Apis mellifera	paired end	2021	NA	2019	USA	North America	28307228	1644	0.01	46	0
PRJNA656275	SRR12424213	Apis mellifera	paired end	2021	NA	2019	USA	North America	28472220	2639	0.01	75	0
PRJNA656275	SRR12424214	Apis mellifera	paired end	2021	NA	2019	USA	North America	28999614	2537	0.01	66	0
PRJNA656275	SRR12424215	Apis mellifera	paired end	2021	NA	2019	USA	North America	28932050	1306	0	33	0
PRJNA656275	SRR12424216	Apis mellifera	paired end	2021	NA	2019	USA	North America	29401788	1341	0	26	0
PRJNA656275	SRR12424217	Apis mellifera	paired end	2021	NA	2019	USA	North America	25868560	1885	0.01	50	0
PRJNA656275	SRR12424218	Apis mellifera	paired end	2021	NA	2019	USA	North America	26440660	1894	0.01	26	0
PRJNA656275	SRR12424219	Apis mellifera	paired end	2021	NA	2019	USA	North America	30653230	12968974	42.31	425588	1.39
PRJNA656275	SRR12424220	Apis mellifera	paired end	2021	NA	2019	USA	North America	31168352	13177240	42.28	430684	1.38
PRJNA656275	SRR12424221	Apis mellifera	paired end	2021	NA	2019	USA	North America	29936408	1489	0	24	0
PRJNA656275	SRR12424222	Apis mellifera	paired end	2021	NA	2019	USA	North America	30480284	1376	0	38	0
PRJNA656275	SRR12424223	Apis mellifera	paired end	2021	NA	2019	USA	North America	26143668	1455	0.01	28	0
PRJNA656275	SRR12424224	Apis mellifera	paired end	2021	NA	2019	USA	North America	26666462	1266	0	23	0
PRJNA656275	SRR12424225	Apis mellifera	paired end	2021	NA	2019	USA	North America	30853210	1309	0	24	0
PRJNA656275	SRR12424226	Apis mellifera	paired end	2021	NA	2019	USA	North America	31254522	1193	0	28	0
PRJNA656275	SRR12424227	Apis mellifera	paired end	2021	NA	2019	USA	North America	31675792	2695	0.01	63	0
PRJNA656275	SRR12424228	Apis mellifera	paired end	2021	NA	2019	USA	North America	32446246	2482	0.01	61	0
PRJNA656275	SRR12424229	Apis mellifera	paired end	2021	NA	2019	USA	North America	27963174	1629	0.01	27	0
PRJNA656275	SRR12424230	Apis mellifera	paired end	2021	NA	2019	USA	North America	28899292	1557	0.01	30	0

PRJNA656275	SRR12424231	Apis mellifera	paired end	2021	NA	2019	USA	North America	38524308	1885	0	32	0
PRJNA656275	SRR12424232	Apis mellifera	paired end	2021	NA	2019	USA	North America	39554956	1604	0	38	0
PRJNA656275	SRR12424233	Apis mellifera	paired end	2021	NA	2019	USA	North America	27844884	987	0	20	0
PRJNA656275	SRR12424234	Apis mellifera	paired end	2021	NA	2019	USA	North America	28631068	937	0	20	0
PRJNA656275	SRR12424235	Apis mellifera	paired end	2021	NA	2019	USA	North America	30513864	1560	0.01	24	0
PRJNA656275	SRR12424236	Apis mellifera	paired end	2021	NA	2019	USA	North America	31346646	1413	0	35	0
PRJNA656275	SRR12424237	Apis mellifera	paired end	2021	NA	2019	USA	North America	31528374	2350	0.01	41	0
PRJNA656275	SRR12424238	Apis mellifera	paired end	2021	NA	2019	USA	North America	32334468	2354	0.01	44	0
PRJNA656275	SRR12424239	Apis mellifera	paired end	2021	NA	2019	USA	North America	35191578	23482	0.07	685	0
PRJNA656275	SRR12424240	Apis mellifera	paired end	2021	NA	2019	USA	North America	35853754	24009	0.07	776	0
PRJNA656275	SRR12424241	Apis mellifera	paired end	2021	NA	2019	USA	North America	30981112	1077	0	20	0
PRJNA656275	SRR12424242	Apis mellifera	paired end	2021	NA	2019	USA	North America	31792990	1108	0	33	0
PRJNA243651	SRR1254946	Apis mellifera	paired end	2014	NA	2012	USA	North America	84696498	3534	0	56	0
PRJNA243651	SRR1254947	Apis mellifera	paired end	2014	NA	2012	USA	North America	71604084	303116	0.42	770	0
PRJNA243651	SRR1254948	Apis mellifera	paired end	2014	NA	2012	USA	North America	100771288	5817	0.01	94	0
PRJNA243651	SRR1254950	Apis mellifera	paired end	2014	NA	2012	USA	North America	68152256	7646840	11.22	829784	1.22
PRJNA243651	SRR1254951	Apis mellifera	paired end	2014	NA	2012	USA	North America	99894982	8788948	8.8	25126	0.03
PRJNA243651	SRR1254952	Apis mellifera	paired end	2014	NA	2012	USA	North America	87014454	3774940	4.34	10125	0.01
PRJNA243651	SRR1254954	Apis mellifera	paired end	2014	NA	2012	USA	North America	102186182	7752	0.01	90	0
PRJNA243651	SRR1254956	Apis mellifera	paired end	2014	NA	2012	USA	North America	95800088	87758	0.09	199	0
PRJNA243651	SRR1254957	Apis mellifera	paired end	2014	NA	2012	USA	North America	78254338	1075500	1.37	2244	0
PRJNA243651	SRR1254958	Apis mellifera	paired end	2014	NA	2012	USA	North America	100449442	10808280	10.76	32560	0.03
PRJNA243651	SRR1254959	Apis mellifera	paired end	2014	NA	2012	USA	North America	88821448	1154870	1.3	7233	0.01
PRJNA243651	SRR1254960	Apis mellifera	paired end	2014	NA	2012	USA	North America	85473762	9869460	11.55	20850	0.02
PRJNA243651	SRR1255009	Apis mellifera	paired end	2014	NA	2012	USA	North America	56342908	795114	1.41	37029	0.07
PRJNA243651	SRR1255010	Apis mellifera	paired end	2014	NA	2012	USA	North America	80527564	19474	0.02	440	0
PRJNA243651	SRR1255011	Apis mellifera	paired end	2014	NA	2012	USA	North America	113374830	5201300	4.59	7600	0.01
PRJNA243651	SRR1255012	Apis mellifera	paired end	2014	NA	2012	USA	North America	59049388	7979264	13.51	484424	0.82
PRJNA243651	SRR1255013	Apis mellifera	paired end	2014	NA	2012	USA	North America	63975208	3380922	5.28	7098	0.01
PRJNA243651	SRR1255014	Apis mellifera	paired end	2014	NA	2012	USA	North America	116391070	21193842	18.21	25770	0.02
PRJNA243651	SRR1255064	Apis mellifera	paired end	2014	NA	2012	USA	North America	104850402	6346506	6.05	9435	0.01
PRJNA243651	SRR1255065	Apis mellifera	paired end	2014	NA	2012	USA	North America	68997582	15535	0.02	314	0
PRJNA243651	SRR1255066	Apis mellifera	paired end	2014	NA	2012	USA	North America	60957858	26367	0.04	382	0
PRJNA243651	SRR1255068	Apis mellifera	paired end	2014	NA	2012	USA	North America	72782026	21679800	29.79	1577332	2.17
PRJNA243651	SRR1255149	Apis mellifera	paired end	2014	NA	2012	USA	North America	68118350	3485340	5.12	4063	0.01
PRJNA243651	SRR1255150	Apis mellifera	paired end	2014	NA	2012	USA	North America	58141740	17579700	30.24	16476	0.03
PRJNA243651	SRR1255151	Apis mellifera	paired end	2014	NA	2012	USA	North America	59131754	5422	0.01	219	0
PRJNA243651	SRR1255152	Apis mellifera	paired end	2014	NA	2012	USA	North America	68500084	1728416	2.52	2192	0
PRJNA243651	SRR1255153	Apis mellifera	paired end	2014	NA	2012	USA	North America	71890206	3656020	5.09	6414	0.01
PRJNA243651	SRR1255154	Apis mellifera	paired end	2014	NA	2012	USA	North America	58745720	19965820	33.99	1779912	3.03
PRJNA243651	SRR1255260	Apis mellifera	paired end	2014	NA	2012	USA	North America	86045054	4637180	5.39	5140	0.01
PRJNA243651	SRR1255326	Apis mellifera	paired end	2014	NA	2012	USA	North America	70998226	13539896	19.07	18682	0.03
PRJNA243651	SRR1255456	Apis mellifera	paired end	2014	NA	2012	USA	North America	53098508	25777550	48.55	2684676	5.06
PRJNA243651	SRR1255541	Apis mellifera	paired end	2014	NA	2012	USA	North America	68678890	61437	0.09	287508	0.42
PRJNA243651	SRR1255542	Apis mellifera	paired end	2014	NA	2012	USA	North America	73889574	1545	0	103	0
PRJNA243651	SRR1255543	Apis mellifera	paired end	2014	NA	2012	USA	North America	69568686	1251	0	77	0
PRJNA243651	SRR1255544	Apis mellifera	paired end	2014	NA	2012	USA	North America	65110644	622	0	51	0
PRJNA243651	SRR1255545	Apis mellifera	paired end	2014	NA	2012	USA	North America	74756862	1266	0	96	0
PRJNA243651	SRR1255546	Apis mellifera	paired end	2014	NA	2012	USA	North America	63355332	4601934	7.26	12599	0.02
PRJNA663557	SRR12659846	Apis mellifera	paired end	2020	2020	2020	USA	North America	132101622	2	0	0	0
PRJNA663557	SRR12659847	Apis mellifera	paired end	2020	2020	2020	USA	North America	148296726	4	0	0	0
PRJNA663557	SRR12659848	Apis mellifera	paired end	2020	2020	2020	USA	North America	101339070	38	0	16	0
PRJNA663557	SRR12659849	Apis mellifera	paired end	2020	2020	2020	USA	North America	141699340	36	0	12	0
PRJNA663557	SRR12659850	Apis mellifera	paired end	2020	2020	2020	USA	North America	143203894	892	0	33	0
PRJNA663557	SRR12659851	Apis mellifera	paired end	2020	2020	2020	USA	North America	129497282	2	0	0	0
PRJNA663557	SRR12659852	Apis mellifera	paired end	2020	2020	2020	USA	North America	123861260	6	0	0	0
PRJNA663557	SRR12659853	Apis mellifera	paired end	2020	2020	2020	USA	North America	161299456	16	0	0	0
PRJNA663557	SRR12659854	Apis mellifera	paired end	2020	2020	2020	USA	North America	137371570	10	0	2	0
PRJNA663557	SRR12659855	Apis mellifera	paired end	2020	2020	2020	USA	North America	143686348	40	0	26	0
PRJNA663557	SRR12659856	Apis mellifera	paired end	2020	2020	2020	USA	North America	145397858	1489	0	265	0
PRJNA663557	SRR12659857	Apis mellifera	paired end	2020	2020	2020	USA	North America	132669328	10	0	0	0
PRJNA663557	SRR12659858	Apis mellifera	paired end	2020	2020	2020	USA	North America	143789630	6	0	0	0
PRJNA663557	SRR12659859	Apis mellifera	paired end	2020	2020	2020	USA	North America	140946686	94	0	0	0
PRJNA243651	SRR1269199	Apis mellifera	paired end	2014	NA	2012	USA	North America	47314632	7465	0.02	924	0
PRJNA668910	SRR12822680	Apis mellifera	paired end	2020	NA	2018	NA	NA	32428634	0	0	0	0
PRJNA668910	SRR12822681	Apis mellifera	paired end	2020	NA	2018	NA	NA	47694494	8	0	0	0
PRJNA668910	SRR12822682	Apis mellifera	paired end	2020	NA	2018	NA	NA	36755418	42	0	0	0
PRJNA668910	SRR12822683	Apis mellifera	paired end	2020	NA	2018	NA	NA	43692420	0	0	0	0
PRJNA668910	SRR12822684	Apis mellifera	paired end	2020	NA	2018	NA	NA	40124630	0	0	0	0
PRJNA668910	SRR12822685	Apis mellifera	paired end	2020	NA	2018	NA	NA	38680758	0	0	0	0
PRJNA783076	SRR12918249	Apis mellifera	paired end	2020	NA	2018	NA	NA	47455326	0	0	0	0
PRJNA670620	SRR12918250	Apis mellifera	paired end	2020	NA	2018	NA	NA	49460832	0	0	0	0
PRJNA670620	SRR12918251	Apis mellifera	paired end	2020	NA	2018	NA	NA	42909530	0	0	0	0
PRJNA670620	SRR12918252	Apis mellifera	paired end	2020	NA	2018	NA	NA	37537460	0	0	0	0
PRJNA783076	SRR12918253	Apis mellifera	paired end	2020	NA	2018	NA	NA	28388802	0	0	0	0
PRJNA783076	SRR12918254	Apis mellifera	paired end	2020	NA	2018	NA	NA	27165236	0	0	0	0
PRJNA783076	SRR12918255	Apis mellifera	paired end	2020	NA	2018	NA	NA	32740430	0	0	0	0
PRJNA765472	SRR12918256	Apis mellifera	paired end	2020	NA	2018	NA	NA	38193764	48	0	0	0
PRJNA765472	SRR12918257	Apis mellifera	paired end	2020	NA	2018	NA	NA	32317364	0	0	0	0
PRJNA765472	SRR12918258	Apis mellifera	paired end	2020	NA	2018	NA	NA	40987628	6	0	0	0
PRJNA765472	SRR12918259	Apis mellifera	paired end	2020	NA	2018	NA	NA	33751558	2	0	0	0
PRJNA765472	SRR12918261	Apis mellifera	paired end	2020	NA	2018	NA	NA	45223350	120	0	2	0
PRJNA765472	SRR12918262	Apis mellifera	paired end	2020	NA	2018	NA	NA	39645952	4	0	0	0
PRJNA670620	SRR12918263	Apis mellifera	paired end	2020	NA	2018	NA	NA	37123970	0	0	0	0
PRJNA670620	SRR12918264	Apis mellifera	paired end	2020	NA	2018	NA	NA	47573294	0	0	0	0
PRJNA670620	SRR12918265	Apis mellifera	paired end	2020	NA	2018	NA	NA	47223204	0	0	0	0
PRJNA783076	SRR12918266	Apis mellifera	paired end	2020	NA	2018	NA	NA	42780882	4	0	0	0
PRJNA783076	SRR12918267	Apis mellifera	paired end	2020	NA	2018	NA	NA	32825650	0	0	0	0
PRJNA783076	SRR12918268	Apis mellifera	paired end	2020	NA	2018	NA	NA	44504424	4	0	0	0
PRJNA783076	SRR12918269	Apis mellifera	paired end	2020	NA	2018	NA	NA	46869270	0	0	0	0
PRJNA783076	SRR12918270	Apis mellifera	paired end	2020	NA	2018	NA	NA	48940684	0	0	0	0
PRJNA681941	SRR13189057	Apis mellifera	paired end	2020	NA	2018	NA	NA	32850332	0	0	0	0
PRJNA681941	SRR13189058	Apis mellifera	paired end	2020	NA	2018	NA	NA	37358100	0	0	0	0
PRJNA681941	SRR13189059	Apis mellifera	paired end	2020	NA	2018	NA	NA	35753438	2	0	0	0
PRJNA681941	SRR13189060	Apis mellifera	paired end	2020	NA	2018	NA	NA	37257226	14	0	0	0
PRJNA681941	SRR13189061	Apis mellifera	paired end	2020	NA	2018	NA	NA	37731756	4	0	0	0
PRJNA681941	SRR13189063	Apis mellifera	paired end	2020	NA	2018	NA	NA	36666998	2557	0.01	4	0
PRJNA681941	SRR13189064	Apis mellifera	paired end	2020	NA	2018	NA	NA	15280886	4192324	27.44	12651	0.08
PRJNA681941	SRR13189065	Apis mellifera	paired end	2020	NA	2018	NA	NA	37901762	590	0	0	0
PRJNA681941	SRR13189066	Apis mellifera	paired end	2020	NA	2018	NA	NA	37086520	8704920	23.47	20025	0.05
PRJNA681941	SRR13189067	Apis mellifera	paired end	2020	NA	2018	NA	NA	37577972	1495	0	2	0
PRJNA683098	SRR13213116	Apis mellifera	paired end	2020	2019	2019	Korea	Asia	40053170	151	0	0	0
PRJNA683673	SRR13221833	Apis mellifera	paired end	2020	2019	2019	Korea	Asia	38651510	11399720	29.49	190391	0.49
PRJNA683681	SRR13221838	Apis mellifera	paired end	2020	2019	2019	Korea	Asia	37621550	747442	1.99	19096	0.05
PRJNA691031	SRR13397502	Apis mellifera	paired end	2021	2015	2015	USA	North America	19568314	2180	0.01	3	0
PRJNA691031	SRR13397505	Apis mellifera	paired end	2021	2015	2015	USA	North America	18468162	3337	0.02	7	0

PRJNA687318	SRR13404629	Apis mellifera	paired end	2021	2018	2018	Israel	Asia	24333066	629082	2.59	698886	2.87
PRJNA687318	SRR13404632	Apis mellifera	paired end	2021	2018	2018	Israel	Asia	111277030	26	0	43	0
PRJNA687318	SRR13404634	Apis mellifera	paired end	2021	2018	2018	Israel	Asia	91329024	68	0	63	0
PRJNA687318	SRR13404636	Apis mellifera	paired end	2021	2018	2018	Israel	Asia	83834060	147	0	149	0
PRJNA687318	SRR13404638	Apis mellifera	paired end	2021	2018	2018	Israel	Asia	8265130	1837186	22.23	1749056	21.16
PRJNA687318	SRR13404641	Apis mellifera	paired end	2021	2018	2018	Israel	Asia	81608252	2207214	2.7	2384876	2.92
PRJNA691740	SRR13425963	Apis mellifera	paired end	2021	2017	2017	China	Asia	56195216	2523740	4.49	4825	0.01
PRJNA691740	SRR13425964	Apis mellifera	paired end	2021	2017	2017	China	Asia	64229850	384408	0.6	815	0
PRJNA691740	SRR13425965	Apis mellifera	paired end	2021	2017	2017	China	Asia	52867010	2005574	3.79	3884	0.01
PRJNA691740	SRR13425966	Apis mellifera	paired end	2021	2017	2017	China	Asia	55450444	3941840	7.11	7639	0.01
PRJNA691740	SRR13425967	Apis mellifera	paired end	2021	2017	2017	China	Asia	48407264	1072426	2.22	1342	0
PRJNA691740	SRR13425968	Apis mellifera	paired end	2021	2017	2017	China	Asia	63378254	3114688	4.91	4953	0.01
PRJNA694695	SRR13523810	Apis mellifera	paired end	2021	2014	2014	USA	North America	25535446	20050400	78.52	23139	0.09
PRJNA694695	SRR13523811	Apis mellifera	paired end	2021	2014	2014	USA	North America	27144956	14721160	54.23	35162	0.13
PRJNA694695	SRR13523812	Apis mellifera	paired end	2021	2014	2014	USA	North America	33834082	23631400	69.84	20609	0.06
PRJNA694695	SRR13523813	Apis mellifera	paired end	2021	2014	2014	USA	North America	29821808	26221600	87.93	31879	0.11
PRJNA694695	SRR13523814	Apis mellifera	paired end	2021	2014	2014	USA	North America	26315780	8572590	32.58	7813	0.03
PRJNA694695	SRR13523815	Apis mellifera	paired end	2021	2014	2014	USA	North America	29568938	19321406	65.34	24531	0.08
PRJNA235974	SRR1386316	Apis mellifera	paired end	2014	2013	2013	Korea	Asia	27286036	911012	3.34	663	0
PRJNA707149	SRR13889652	Apis mellifera	paired end	2021	2016	2016	Germany	Europe	40215294	0	0	0	0
PRJNA707149	SRR13889687	Apis mellifera	paired end	2021	2016	2016	Germany	Europe	41690046	0	0	0	0
PRJNA707149	SRR13889692	Apis mellifera	paired end	2021	2016	2016	Germany	Europe	40743708	0	0	0	0
PRJNA707149	SRR13889693	Apis mellifera	paired end	2021	2016	2016	Germany	Europe	41757890	0	0	0	0
PRJNA707149	SRR13889694	Apis mellifera	paired end	2021	2016	2016	Germany	Europe	41725864	0	0	0	0
PRJNA707149	SRR13889695	Apis mellifera	paired end	2021	2016	2016	Germany	Europe	41680744	0	0	0	0
PRJNA707149	SRR13889696	Apis mellifera	paired end	2021	2016	2016	Germany	Europe	40531432	0	0	10	0
PRJNA707149	SRR13889697	Apis mellifera	paired end	2021	2016	2016	Germany	Europe	40891038	0	0	0	0
PRJNA707149	SRR13889698	Apis mellifera	paired end	2021	2016	2016	Germany	Europe	41171436	0	0	0	0
PRJNA707149	SRR13889699	Apis mellifera	paired end	2021	2016	2016	Germany	Europe	41897754	0	0	12	0
PRJNA707149	SRR13889701	Apis mellifera	paired end	2021	2016	2016	Germany	Europe	41266480	0	0	0	0
PRJNA707149	SRR13889702	Apis mellifera	paired end	2021	2016	2016	Germany	Europe	41197250	0	0	0	0
PRJNA707149	SRR13889703	Apis mellifera	paired end	2021	2016	2016	Germany	Europe	41997632	0	0	0	0
PRJNA707149	SRR13889704	Apis mellifera	paired end	2021	2016	2016	Germany	Europe	41546830	0	0	0	0
PRJNA707149	SRR13889705	Apis mellifera	paired end	2021	2016	2016	Germany	Europe	41838158	0	0	0	0
PRJNA707149	SRR13889706	Apis mellifera	paired end	2021	2016	2016	Germany	Europe	40491840	0	0	0	0
PRJNA707149	SRR13889707	Apis mellifera	paired end	2021	2016	2016	Germany	Europe	40809324	0	0	0	0
PRJNA707149	SRR13889708	Apis mellifera	paired end	2021	2016	2016	Germany	Europe	41922854	0	0	72	0
PRJNA707149	SRR13889709	Apis mellifera	paired end	2021	2016	2016	Germany	Europe	40648112	0	0	0	0
PRJNA707149	SRR13889710	Apis mellifera	paired end	2021	2016	2016	Germany	Europe	41570838	0	0	0	0
PRJNA707149	SRR13889712	Apis mellifera	paired end	2021	2016	2016	Germany	Europe	40703208	0	0	34	0
PRJNA707149	SRR13889713	Apis mellifera	paired end	2021	2016	2016	Germany	Europe	40766764	0	0	0	0
PRJNA707149	SRR13889714	Apis mellifera	paired end	2021	2016	2016	Germany	Europe	40701824	0	0	0	0
PRJNA707149	SRR13889715	Apis mellifera	paired end	2021	2016	2016	Germany	Europe	41150762	0	0	162	0
PRJNA707149	SRR13889716	Apis mellifera	paired end	2021	2016	2016	Germany	Europe	40330752	0	0	0	0
PRJNA707149	SRR13889717	Apis mellifera	paired end	2021	2016	2016	Germany	Europe	41278742	0	0	0	0
PRJNA707149	SRR13889718	Apis mellifera	paired end	2021	2016	2016	Germany	Europe	40690824	0	0	0	0
PRJNA707149	SRR13889719	Apis mellifera	paired end	2021	2016	2016	Germany	Europe	41238640	0	0	0	0
PRJNA235974	SRR1407793	Apis mellifera	paired end	2014	2013	2013	Korea	Asia	27922064	400997	1.44	5	0
PRJNA235974	SRR1408090	Apis mellifera	paired end	2014	2013	2013	Korea	Asia	25981706	5244076	20.18	76	0
PRJNA260604	SRR1571716	Apis mellifera	paired end	2015	NA	2013	NA	NA	24706974	0	0	0	0
PRJNA260604	SRR1571717	Apis mellifera	paired end	2015	NA	2013	NA	NA	15594902	0	0	0	0
PRJNA260604	SRR1571719	Apis mellifera	paired end	2015	NA	2013	NA	NA	14881274	0	0	0	0
PRJNA260604	SRR1571720	Apis mellifera	paired end	2015	NA	2013	NA	NA	60163402	0	0	0	0
PRJNA260604	SRR1571721	Apis mellifera	paired end	2015	NA	2013	NA	NA	29314642	0	0	0	0
PRJNA260604	SRR1571722	Apis mellifera	paired end	2015	NA	2013	NA	NA	20329860	0	0	0	0
PRJNA260604	SRR1571723	Apis mellifera	paired end	2015	NA	2013	NA	NA	9024410	0	0	0	0
PRJNA260604	SRR1571724	Apis mellifera	paired end	2015	NA	2013	NA	NA	11233148	0	0	0	0
PRJNA260604	SRR1571725	Apis mellifera	paired end	2015	NA	2013	NA	NA	12917528	0	0	0	0
PRJNA260604	SRR1571726	Apis mellifera	paired end	2015	NA	2013	NA	NA	12370526	0	0	0	0
PRJNA260604	SRR1571727	Apis mellifera	paired end	2015	NA	2013	NA	NA	10541742	0	0	0	0
PRJNA260604	SRR1571728	Apis mellifera	paired end	2015	NA	2013	NA	NA	13588366	0	0	0	0
PRJNA260604	SRR1571729	Apis mellifera	paired end	2015	NA	2013	NA	NA	18439692	0	0	0	0
PRJNA260604	SRR1571730	Apis mellifera	paired end	2015	NA	2013	NA	NA	14297628	0	0	0	0
PRJNA260604	SRR1571731	Apis mellifera	paired end	2015	NA	2013	NA	NA	18391094	0	0	0	0
PRJNA274674	SRR1790682	Apis mellifera	paired end	2015	NA	2013	NA	NA	52410858	8	0	0	0
PRJNA274674	SRR1790683	Apis mellifera	paired end	2015	NA	2013	NA	NA	52253560	0	0	0	0
PRJNA274674	SRR1790684	Apis mellifera	paired end	2015	NA	2013	NA	NA	53182352	6	0	0	0
PRJNA274674	SRR1790685	Apis mellifera	paired end	2015	NA	2013	NA	NA	53051222	8	0	0	0
PRJNA274674	SRR1790686	Apis mellifera	paired end	2015	NA	2013	NA	NA	51231170	23123	0.05	0	0
PRJNA274674	SRR1790687	Apis mellifera	paired end	2015	NA	2013	NA	NA	53823222	6	0	0	0
PRJNA277772	SRR2032812	Apis mellifera	paired end	2015	2008	2008	NA	NA	43050520	6	0	0	0
PRJNA277772	SRR2032813	Apis mellifera	paired end	2015	2008	2008	NA	NA	39364294	2	0	0	0
PRJNA277772	SRR2032814	Apis mellifera	paired end	2015	2008	2008	NA	NA	36395536	28	0	0	0
PRJNA277772	SRR2032816	Apis mellifera	paired end	2015	2008	2008	NA	NA	43767714	3	0	0	0
PRJNA277772	SRR2032817	Apis mellifera	paired end	2015	2008	2008	NA	NA	14887322	4	0	0	0
PRJNA277772	SRR2032827	Apis mellifera	paired end	2015	2008	2008	NA	NA	12690520	2	0	0	0
PRJNA277772	SRR2032844	Apis mellifera	paired end	2015	2008	2008	NA	NA	44576936	2	0	0	0
PRJNA277772	SRR2032857	Apis mellifera	paired end	2015	2008	2008	NA	NA	60196844	4	0	0	0
PRJNA277772	SRR2032888	Apis mellifera	paired end	2015	2008	2008	NA	NA	33495904	5	0	0	0
PRJNA277772	SRR2032907	Apis mellifera	paired end	2015	2008	2008	NA	NA	71717328	22	0	0	0
PRJNA277772	SRR2032921	Apis mellifera	paired end	2015	2008	2008	NA	NA	44884920	9	0	0	0
PRJNA277772	SRR2032930	Apis mellifera	paired end	2015	2008	2008	NA	NA	89512416	4	0	0	0
PRJNA277772	SRR2033014	Apis mellifera	paired end	2015	2008	2008	NA	NA	19122346	0	0	0	0
PRJNA277772	SRR2033015	Apis mellifera	paired end	2015	2008	2008	NA	NA	88655922	1364	0	0	0
PRJNA284414	SRR2033979	Apis mellifera	paired end	2016	2013	2013	USA	North America	97152478	0	0	0	0
PRJNA284414	SRR2034245	Apis mellifera	paired end	2016	2013	2013	USA	North America	104232110	0	0	0	0
PRJNA284414	SRR2034249	Apis mellifera	paired end	2016	2013	2013	USA	North America	100647170	56	0	0	0
PRJNA284414	SRR2034253	Apis mellifera	paired end	2016	2013	2013	USA	North America	99690100	2	0	0	0
PRJNA284414	SRR2034262	Apis mellifera	paired end	2016	2013	2013	USA	North America	104330086	60475	0.06	0	0
PRJNA284414	SRR2034266	Apis mellifera	paired end	2016	2013	2013	USA	North America	100236838	16	0	0	0
PRJNA284414	SRR2034269	Apis mellifera	paired end	2016	2013	2013	USA	North America	88941804	7841	0.01	0	0
PRJNA284414	SRR2034271	Apis mellifera	paired end	2016	2013	2013	USA	North America	90724100	1403	0	8	0
PRJNA292006	SRR2146246	Apis mellifera	paired end	2015	NA	2013	China	Asia	33840916	4248083	12.55	247	0
PRJNA292006	SRR2146247	Apis mellifera	paired end	2015	NA	2013	China	Asia	34718208	5920445	17.05	1373	0
PRJNA292006	SRR2146248	Apis mellifera	paired end	2015	NA	2013	China	Asia	36562750	5978326	16.35	2309	0.01
PRJNA292006	SRR2146249	Apis mellifera	paired end	2015	NA	2013	China	Asia	35947082	8391767	23.34	2865	0.01
PRJNA292006	SRR2146250	Apis mellifera	paired end	2015	NA	2013	China	Asia	38509164	104220	0.27	42	0
PRJNA292006	SRR2146251	Apis mellifera	paired end	2015	NA	2013	China	Asia	38579116	511605	1.33	87	0
PRJNA292367	SRR2153227	Apis mellifera	paired end	2015	NA	2013	NA	NA	35856924	0	0	0	0
PRJNA292367	SRR2153228	Apis mellifera	paired end	2015	NA	2013	NA	NA	32760100	0	0	0	0
PRJNA292367	SRR2153229	Apis mellifera	paired end	2015	NA	2013	NA	NA	39689474	0	0	0	0
PRJNA292367	SRR2153257	Apis mellifera	paired end	2015	NA	2013	NA	NA	32244884	0	0	0	0
PRJNA292367	SRR2153259	Apis mellifera	paired end	2015	NA	2013	NA	NA	32970958	0	0	0	0
PRJNA292367	SRR2153260	Apis mellifera	paired end	2015	NA	2013	NA	NA	36707338	0	0	0	0
PRJNA292367	SRR2153280	Apis mellifera	paired end	2015	NA	2013	NA	NA	33883960	0	0	0	0
PRJNA292367	SRR2153281	Apis mellifera	paired end	2015	NA	2013	NA	NA	34022922	0	0	0	0

PRJNA292367	SRR2153282	Apis mellifera	paired end	2015	NA	2013	NA	NA	32595952	0	0	0	0
PRJNA292367	SRR2153283	Apis mellifera	paired end	2015	NA	2013	NA	NA	33682510	0	0	0	0
PRJNA292367	SRR2153286	Apis mellifera	paired end	2015	NA	2013	NA	NA	32331020	0	0	0	0
PRJNA292367	SRR2153287	Apis mellifera	paired end	2015	NA	2013	NA	NA	31253780	0	0	0	0
PRJNA306498	SRR3033249	Apis mellifera	paired end	2015	NA	2013	USA	North America	34893952	882	0	0	0
PRJNA306498	SRR3033250	Apis mellifera	paired end	2015	NA	2013	USA	North America	29357976	1002	0	0	0
PRJNA306498	SRR3033251	Apis mellifera	paired end	2015	NA	2013	USA	North America	43506120	2187	0.01	0	0
PRJNA306498	SRR3033252	Apis mellifera	paired end	2015	NA	2013	USA	North America	36786804	1477	0	0	0
PRJNA306498	SRR3033253	Apis mellifera	paired end	2015	NA	2013	USA	North America	44528964	1171	0	0	0
PRJNA306498	SRR3033254	Apis mellifera	paired end	2015	NA	2013	USA	North America	39600028	12133684	30.64	4	0
PRJNA306498	SRR3033255	Apis mellifera	paired end	2015	NA	2013	USA	North America	34761798	3106	0.01	0	0
PRJNA306498	SRR3033256	Apis mellifera	paired end	2015	NA	2013	USA	North America	20513676	660	0	0	0
PRJNA306498	SRR3033257	Apis mellifera	paired end	2015	NA	2013	USA	North America	40135492	1398	0	0	0
PRJNA306498	SRR3033258	Apis mellifera	paired end	2015	NA	2013	USA	North America	36052958	789	0	0	0
PRJNA306498	SRR3033259	Apis mellifera	paired end	2015	NA	2013	USA	North America	44365574	6412	0.01	0	0
PRJNA306498	SRR3033260	Apis mellifera	paired end	2015	NA	2013	USA	North America	43936368	1215	0	0	0
PRJNA306498	SRR3033261	Apis mellifera	paired end	2015	NA	2013	USA	North America	28026396	720	0	0	0
PRJNA306498	SRR3033262	Apis mellifera	paired end	2015	NA	2013	USA	North America	47304832	9172	0.02	0	0
PRJNA306498	SRR3033263	Apis mellifera	paired end	2015	NA	2013	USA	North America	44920678	3518	0.01	0	0
PRJNA306498	SRR3033264	Apis mellifera	paired end	2015	NA	2013	USA	North America	35799452	1381	0	0	0
PRJNA306498	SRR3033265	Apis mellifera	paired end	2015	NA	2013	USA	North America	32720998	12380056	37.84	371	0
PRJNA306498	SRR3033266	Apis mellifera	paired end	2015	NA	2013	USA	North America	36144262	21269792	58.85	14	0
PRJNA306498	SRR3033267	Apis mellifera	paired end	2015	NA	2013	USA	North America	39348262	17742	0.05	2	0
PRJNA306498	SRR3033268	Apis mellifera	paired end	2015	NA	2013	USA	North America	44621244	1800	0	0	0
PRJNA306498	SRR3033269	Apis mellifera	paired end	2015	NA	2013	USA	North America	43300692	2269	0.01	0	0
PRJNA306498	SRR3033270	Apis mellifera	paired end	2015	NA	2013	USA	North America	33221370	2502	0.01	0	0
PRJNA306498	SRR3033271	Apis mellifera	paired end	2015	NA	2013	USA	North America	41063770	1205	0	0	0
PRJNA306498	SRR3033272	Apis mellifera	paired end	2015	NA	2013	USA	North America	37019256	2145	0.01	0	0
PRJNA306498	SRR3033273	Apis mellifera	paired end	2015	NA	2013	USA	North America	42191066	1630	0	0	0
PRJNA308280	SRR3095836	Apis mellifera	paired end	2016	NA	2014	China	Asia	61252688	0	0	0	0
PRJNA308280	SRR3095837	Apis mellifera	paired end	2016	NA	2014	China	Asia	56542232	0	0	0	0
PRJNA308280	SRR3095838	Apis mellifera	paired end	2016	NA	2014	China	Asia	71928078	0	0	0	0
PRJNA308280	SRR3095839	Apis mellifera	paired end	2016	NA	2014	China	Asia	37936248	2	0	0	0
PRJNA308280	SRR3095842	Apis mellifera	paired end	2016	NA	2014	China	Asia	38630224	2	0	0	0
PRJNA308280	SRR3095843	Apis mellifera	paired end	2016	NA	2014	China	Asia	38746076	2	0	0	0
PRJNA308280	SRR3095845	Apis mellifera	paired end	2016	NA	2014	China	Asia	57529296	0	0	0	0
PRJNA308280	SRR3095846	Apis mellifera	paired end	2016	NA	2014	China	Asia	64377590	0	0	0	0
PRJNA308280	SRR3095847	Apis mellifera	paired end	2016	NA	2014	China	Asia	66253180	0	0	0	0
PRJNA308280	SRR3095848	Apis mellifera	paired end	2016	NA	2014	China	Asia	40573280	5	0	0	0
PRJNA308280	SRR3095849	Apis mellifera	paired end	2016	NA	2014	China	Asia	39374288	23	0	0	0
PRJNA308280	SRR3095850	Apis mellifera	paired end	2016	NA	2014	China	Asia	40207406	4	0	0	0
PRJNA308280	SRR3095863	Apis mellifera	paired end	2016	NA	2014	China	Asia	58623948	0	0	0	0
PRJNA308280	SRR3095864	Apis mellifera	paired end	2016	NA	2014	China	Asia	57986420	0	0	0	0
PRJNA308280	SRR3095865	Apis mellifera	paired end	2016	NA	2014	China	Asia	62037130	0	0	0	0
PRJNA308280	SRR3095866	Apis mellifera	paired end	2016	NA	2014	China	Asia	40890226	17	0	0	0
PRJNA308280	SRR3095867	Apis mellifera	paired end	2016	NA	2014	China	Asia	43069980	2	0	0	0
PRJNA308280	SRR3095868	Apis mellifera	paired end	2016	NA	2014	China	Asia	42971330	1	0	0	0
PRJNA308280	SRR3095870	Apis mellifera	paired end	2016	NA	2014	China	Asia	63437492	0	0	0	0
PRJNA308280	SRR3095871	Apis mellifera	paired end	2016	NA	2014	China	Asia	64041608	0	0	0	0
PRJNA308280	SRR3095872	Apis mellifera	paired end	2016	NA	2014	China	Asia	69113124	0	0	0	0
PRJNA308280	SRR3095873	Apis mellifera	paired end	2016	NA	2014	China	Asia	40205518	0	0	0	0
PRJNA308280	SRR3095874	Apis mellifera	paired end	2016	NA	2014	China	Asia	40658716	0	0	0	0
PRJNA308280	SRR3095876	Apis mellifera	paired end	2016	NA	2014	China	Asia	40320978	0	0	0	0
PRJNA308790	SRR3102934	Apis mellifera	paired end	2016	NA	2014	China	Asia	33883960	0	0	0	0
PRJNA308790	SRR3123272	Apis mellifera	paired end	2016	NA	2014	China	Asia	34022922	0	0	0	0
PRJNA308790	SRR3123273	Apis mellifera	paired end	2016	NA	2014	China	Asia	32595952	0	0	0	0
PRJNA308790	SRR3123275	Apis mellifera	paired end	2016	NA	2014	China	Asia	33682510	0	0	0	0
PRJNA308790	SRR3123276	Apis mellifera	paired end	2016	NA	2014	China	Asia	32331020	0	0	0	0
PRJNA308790	SRR3123277	Apis mellifera	paired end	2016	NA	2014	China	Asia	31253780	0	0	0	0
PRJNA308790	SRR3123279	Apis mellifera	paired end	2016	NA	2014	China	Asia	35856924	0	0	0	0
PRJNA308790	SRR3123281	Apis mellifera	paired end	2016	NA	2014	China	Asia	32760100	0	0	0	0
PRJNA308790	SRR3123337	Apis mellifera	paired end	2016	NA	2014	China	Asia	39689474	0	0	0	0
PRJNA308790	SRR3123340	Apis mellifera	paired end	2016	NA	2014	China	Asia	32244884	0	0	0	0
PRJNA308790	SRR3123341	Apis mellifera	paired end	2016	NA	2014	China	Asia	32970958	0	0	0	0
PRJNA308790	SRR3123342	Apis mellifera	paired end	2016	NA	2014	China	Asia	36707338	0	0	0	0
PRJNA308790	SRR3123355	Apis mellifera	paired end	2016	NA	2014	China	Asia	33873020	0	0	0	0
PRJNA308790	SRR3123357	Apis mellifera	paired end	2016	NA	2014	China	Asia	32369610	0	0	0	0
PRJNA308790	SRR3123359	Apis mellifera	paired end	2016	NA	2014	China	Asia	27768686	0	0	0	0
PRJNA308790	SRR3123361	Apis mellifera	paired end	2016	NA	2014	China	Asia	33399192	0	0	0	0
PRJNA308790	SRR3123362	Apis mellifera	paired end	2016	NA	2014	China	Asia	33433554	0	0	0	0
PRJNA308790	SRR3123364	Apis mellifera	paired end	2016	NA	2014	China	Asia	36454980	1	0	0	0
PRJNA308790	SRR3123372	Apis mellifera	paired end	2016	NA	2014	China	Asia	33426734	4	0	0	0
PRJNA308790	SRR3123380	Apis mellifera	paired end	2016	NA	2014	China	Asia	40275754	0	0	0	0
PRJNA308790	SRR3123385	Apis mellifera	paired end	2016	NA	2014	China	Asia	32183792	0	0	0	0
PRJNA308790	SRR3123388	Apis mellifera	paired end	2016	NA	2014	China	Asia	32317174	0	0	0	0
PRJNA308790	SRR3123389	Apis mellifera	paired end	2016	NA	2014	China	Asia	32989456	0	0	0	0
PRJNA308790	SRR3123390	Apis mellifera	paired end	2016	NA	2014	China	Asia	35212984	0	0	0	0
PRJNA308790	SRR3123400	Apis mellifera	paired end	2016	NA	2014	China	Asia	32833290	0	0	0	0
PRJNA308790	SRR3123402	Apis mellifera	paired end	2016	NA	2014	China	Asia	36296438	1	0	0	0
PRJNA308790	SRR3123404	Apis mellifera	paired end	2016	NA	2014	China	Asia	34241936	0	0	0	0
PRJNA308790	SRR3123406	Apis mellifera	paired end	2016	NA	2014	China	Asia	37974664	0	0	0	0
PRJNA308790	SRR3123407	Apis mellifera	paired end	2016	NA	2014	China	Asia	40124606	0	0	0	0
PRJNA308790	SRR3123408	Apis mellifera	paired end	2016	NA	2014	China	Asia	33364166	0	0	0	0
PRJNA308790	SRR3123443	Apis mellifera	paired end	2016	NA	2014	China	Asia	35289980	0	0	0	0
PRJNA308790	SRR3123445	Apis mellifera	paired end	2016	NA	2014	China	Asia	32344952	0	0	0	0
PRJNA308790	SRR3123446	Apis mellifera	paired end	2016	NA	2014	China	Asia	27296956	0	0	0	0
PRJNA308790	SRR3123448	Apis mellifera	paired end	2016	NA	2014	China	Asia	33222808	0	0	0	0
PRJNA308790	SRR3123449	Apis mellifera	paired end	2016	NA	2014	China	Asia	34466998	0	0	0	0
PRJNA308790	SRR3123451	Apis mellifera	paired end	2016	NA	2014	China	Asia	36370760	0	0	0	0
PRJNA310321	SRR3144549	Apis mellifera	paired end	2017	NA	2015	China	Asia	41010136	0	0	0	0
PRJNA310321	SRR3144556	Apis mellifera	paired end	2017	NA	2015	China	Asia	36899854	0	0	0	0
PRJNA310321	SRR3144557	Apis mellifera	paired end	2017	NA	2015	China	Asia	30375792	0	0	0	0
PRJNA310321	SRR3144559	Apis mellifera	paired end	2017	NA	2015	China	Asia	30871944	0	0	0	0
PRJNA310321	SRR3144561	Apis mellifera	paired end	2017	NA	2015	China	Asia	35434822	0	0	0	0
PRJNA310321	SRR3144562	Apis mellifera	paired end	2017	NA	2015	China	Asia	34188640	0	0	0	0
PRJNA310321	SRR3144563	Apis mellifera	paired end	2017	NA	2015	China	Asia	30267660	0	0	0	0
PRJNA310321	SRR3144564	Apis mellifera	paired end	2017	NA	2015	China	Asia	41010136	0	0	0	0
PRJNA310321	SRR3144566	Apis mellifera	paired end	2017	NA	2015	China	Asia	33403486	0	0	0	0
PRJNA310321	SRR3144567	Apis mellifera	paired end	2017	NA	2015	China	Asia	26314554	0	0	0	0
PRJNA322424	SRR3569809	Apis mellifera	paired end	2017	NA	2015	NA	NA	53709244	19	0	0	0
PRJNA322424	SRR3569811	Apis mellifera	paired end	2017	NA	2015	NA	NA	76738426	0	0	0	0
PRJNA322424	SRR3569812	Apis mellifera	paired end	2017	NA	2015	NA	NA	75631112	3706	0	0	0
PRJNA322424	SRR3569813	Apis mellifera	paired end	2017	NA	2015	NA	NA	65738632	6671	0.01	1	0
PRJNA327226	SRR3730220	Apis mellifera	paired end	2016	2014	2014	Czech	Europe	74460	0	0	0	0
PRJNA327226	SRR3730221	Apis mellifera	paired end	2016	2014	2014	Czech	Europe	71214	0	0	0	0
PRJNA327226	SRR3730222	Apis mellifera	paired end	2016	2014	2014	Czech	Europe	59872	0	0	0	0

PRJNA327226	SRR3730223	Apis mellifera	paired end	2016	2014	2014	Czech	Europe	61260	0	0	0	0
PRJNA327226	SRR3730224	Apis mellifera	paired end	2016	2014	2014	Czech	Europe	59444	0	0	0	0
PRJNA327226	SRR3730225	Apis mellifera	paired end	2016	2014	2014	Czech	Europe	51014	0	0	0	0
PRJNA327226	SRR3730226	Apis mellifera	paired end	2016	2014	2014	Czech	Europe	92908	0	0	0	0
PRJNA327226	SRR3730227	Apis mellifera	paired end	2016	2014	2014	Czech	Europe	104802	0	0	0	0
PRJNA327226	SRR3730228	Apis mellifera	paired end	2016	2014	2014	Czech	Europe	123252	0	0	0	0
PRJNA327226	SRR3730229	Apis mellifera	paired end	2016	2014	2014	Czech	Europe	61768	0	0	0	0
PRJNA327226	SRR3730231	Apis mellifera	paired end	2016	2014	2014	Czech	Europe	26792	0	0	0	0
PRJNA327226	SRR3730232	Apis mellifera	paired end	2016	2014	2014	Czech	Europe	107480	0	0	0	0
PRJNA327226	SRR3730233	Apis mellifera	paired end	2016	2014	2014	Czech	Europe	25876	0	0	0	0
PRJNA327226	SRR3730234	Apis mellifera	paired end	2016	2014	2014	Czech	Europe	104230	0	0	0	0
PRJNA327226	SRR3730235	Apis mellifera	paired end	2016	2014	2014	Czech	Europe	59334	0	0	0	0
PRJNA327226	SRR3730236	Apis mellifera	paired end	2016	2014	2014	Czech	Europe	57366	0	0	0	0
PRJNA327226	SRR3730237	Apis mellifera	paired end	2016	2014	2014	Czech	Europe	83906	0	0	0	0
PRJNA327226	SRR3730238	Apis mellifera	paired end	2016	2014	2014	Czech	Europe	43332	0	0	0	0
PRJNA327226	SRR3730239	Apis mellifera	paired end	2016	2014	2014	Czech	Europe	38596	0	0	0	0
PRJNA327226	SRR3730240	Apis mellifera	paired end	2016	2014	2014	Czech	Europe	70082	0	0	0	0
PRJNA327226	SRR3730241	Apis mellifera	paired end	2016	2014	2014	Czech	Europe	76556	0	0	0	0
PRJNA327226	SRR3730242	Apis mellifera	paired end	2016	2014	2014	Czech	Europe	84114	0	0	0	0
PRJNA329428	SRR3927497	Apis mellifera	paired end	2016	2014	2014	Israel	Asia	62442992	4754573	7.61	12266902	19.64
PRJNA329428	SRR3927501	Apis mellifera	paired end	2016	2014	2014	Israel	Asia	142947386	707138	0.49	2038118	1.43
PRJNA336281	SRR4002869	Apis mellifera	paired end	2016	NA	2014	Belgium	Europe	15976786	14989	0.09	10932322	68.43
PRJNA336281	SRR4002870	Apis mellifera	paired end	2016	NA	2014	Belgium	Europe	20364816	7095	0.03	12431382	61.04
PRJNA336281	SRR4002871	Apis mellifera	paired end	2016	NA	2014	Belgium	Europe	19894694	11886	0.06	11923090	59.93
PRJNA336281	SRR4002872	Apis mellifera	paired end	2016	NA	2014	Belgium	Europe	18386468	5640	0.03	10402447	56.58
PRJNA336281	SRR4002873	Apis mellifera	paired end	2016	NA	2014	Belgium	Europe	16070270	7138	0.04	7559409	47.04
PRJNA336281	SRR4002874	Apis mellifera	paired end	2016	NA	2014	Belgium	Europe	15662748	6913	0.04	6351604	40.55
PRJNA336281	SRR4002875	Apis mellifera	paired end	2016	NA	2014	Belgium	Europe	17414086	8837	0.05	12545002	72.04
PRJNA336281	SRR4002876	Apis mellifera	paired end	2016	NA	2014	Belgium	Europe	16863568	8881	0.05	9404899	55.77
PRJNA336281	SRR4002877	Apis mellifera	paired end	2016	NA	2014	Belgium	Europe	19222374	20373	0.11	14237820	74.07
PRJNA336281	SRR4002878	Apis mellifera	paired end	2016	NA	2014	Belgium	Europe	18016372	2865	0.02	10196717	56.6
PRJNA336281	SRR4002880	Apis mellifera	paired end	2016	NA	2014	Belgium	Europe	19493048	0	0	4239	0.02
PRJNA336281	SRR4002881	Apis mellifera	paired end	2016	NA	2014	Belgium	Europe	14443630	9	0	4660	0.03
PRJNA336281	SRR4002882	Apis mellifera	paired end	2016	NA	2014	Belgium	Europe	17475374	0	0	3744	0.02
PRJNA336281	SRR4002883	Apis mellifera	paired end	2016	NA	2014	Belgium	Europe	15897470	6	0	11900	0.07
PRJNA336281	SRR4002884	Apis mellifera	paired end	2016	NA	2014	Belgium	Europe	18509746	3	0	5243	0.03
PRJNA336281	SRR4002885	Apis mellifera	paired end	2016	NA	2014	Belgium	Europe	16543900	7	0	3387	0.02
PRJNA336281	SRR4002886	Apis mellifera	paired end	2016	NA	2014	Belgium	Europe	15963186	13	0	12940	0.08
PRJNA336281	SRR4002888	Apis mellifera	paired end	2016	NA	2014	Belgium	Europe	19114308	4	0	3686	0.02
PRJNA336281	SRR4002889	Apis mellifera	paired end	2016	NA	2014	Belgium	Europe	17909002	4	0	3338	0.02
PRJNA336281	SRR4002890	Apis mellifera	paired end	2016	NA	2014	Belgium	Europe	24121738	12041	0.05	14613880	60.58
PRJNA336281	SRR4002891	Apis mellifera	paired end	2016	NA	2014	Belgium	Europe	20293638	481	0	7344221	36.19
PRJNA336281	SRR4002892	Apis mellifera	paired end	2016	NA	2014	Belgium	Europe	24827514	3492	0.01	13062544	52.61
PRJNA336281	SRR4002893	Apis mellifera	paired end	2016	NA	2014	Belgium	Europe	18220696	1012	0.01	6179510	33.91
PRJNA336281	SRR4002894	Apis mellifera	paired end	2016	NA	2014	Belgium	Europe	17798332	16305	0.09	12203111	68.56
PRJNA336281	SRR4002895	Apis mellifera	paired end	2016	NA	2014	Belgium	Europe	21076724	6377	0.03	10120263	48.02
PRJNA336281	SRR4002896	Apis mellifera	paired end	2016	NA	2014	Belgium	Europe	23226848	8175	0.04	15245191	65.64
PRJNA336281	SRR4002897	Apis mellifera	paired end	2016	NA	2014	Belgium	Europe	17463768	3787	0.02	6425807	36.8
PRJNA336281	SRR4002898	Apis mellifera	paired end	2016	NA	2014	Belgium	Europe	13734454	4641	0.03	9300770	67.72
PRJNA336281	SRR4002899	Apis mellifera	paired end	2016	NA	2014	Belgium	Europe	16917144	8806	0.05	9501469	56.16
PRJNA338112	SRR4013500	Apis mellifera	paired end	2017	NA	2015	NA	NA	28511468	71922	0.25	0	0
PRJNA338112	SRR4013514	Apis mellifera	paired end	2017	NA	2015	NA	NA	25747960	2	0	0	0
PRJNA338112	SRR4013515	Apis mellifera	paired end	2017	NA	2015	NA	NA	24205380	0	0	0	0
PRJNA338112	SRR4013516	Apis mellifera	paired end	2017	NA	2015	NA	NA	26043632	239461	0.92	0	0
PRJNA338112	SRR4013517	Apis mellifera	paired end	2017	NA	2015	NA	NA	31893992	11	0	0	0
PRJNA338112	SRR4013518	Apis mellifera	paired end	2017	NA	2015	NA	NA	30790968	0	0	0	0
PRJNA338112	SRR4013519	Apis mellifera	paired end	2017	NA	2015	NA	NA	24758732	207	0	0	0
PRJNA338112	SRR4013520	Apis mellifera	paired end	2017	NA	2015	NA	NA	29008662	2	0	0	0
PRJNA338112	SRR4013521	Apis mellifera	paired end	2017	NA	2015	NA	NA	34519110	0	0	0	0
PRJNA338450	SRR4017751	Apis mellifera	paired end	2016	NA	2014	USA	North America	51181416	18507651	36.16	67	0
PRJNA338450	SRR4017752	Apis mellifera	paired end	2016	NA	2014	USA	North America	49408622	18274	0.04	0	0
PRJNA338450	SRR4017753	Apis mellifera	paired end	2016	NA	2014	USA	North America	42437846	3052706	7.19	8	0
PRJNA338450	SRR4017754	Apis mellifera	paired end	2016	NA	2014	USA	North America	51259100	19971471	38.96	21	0
PRJNA338450	SRR4017755	Apis mellifera	paired end	2016	NA	2014	USA	North America	45879604	12103491	26.38	73	0
PRJNA338450	SRR4017756	Apis mellifera	paired end	2016	NA	2014	USA	North America	65560506	8383395	12.79	45	0
PRJNA338450	SRR4017757	Apis mellifera	paired end	2016	NA	2014	USA	North America	63082340	13771839	21.83	8628	0.01
PRJNA338450	SRR4017758	Apis mellifera	paired end	2016	NA	2014	USA	North America	57642432	9898585	17.17	54	0
PRJNA338450	SRR4017759	Apis mellifera	paired end	2016	NA	2014	USA	North America	50782012	10435922	20.55	1906	0
PRJNA338450	SRR4017760	Apis mellifera	paired end	2016	NA	2014	USA	North America	53442138	5776107	10.81	6	0
PRJNA338450	SRR4017761	Apis mellifera	paired end	2016	NA	2014	USA	North America	61025042	10152706	16.64	13	0
PRJNA338450	SRR4017762	Apis mellifera	paired end	2016	NA	2014	USA	North America	50485938	13313330	26.37	85	0
PRJNA338450	SRR4017763	Apis mellifera	paired end	2016	NA	2014	USA	North America	56988970	16297017	28.6	49	0
PRJNA338450	SRR4017764	Apis mellifera	paired end	2016	NA	2014	USA	North America	60904226	23685307	38.89	100	0
PRJNA338450	SRR4017765	Apis mellifera	paired end	2016	NA	2014	USA	North America	56708662	18468322	32.57	34	0
PRJNA338450	SRR4017766	Apis mellifera	paired end	2016	NA	2014	USA	North America	48755352	9957133	20.42	39	0
PRJNA338450	SRR4017767	Apis mellifera	paired end	2016	NA	2014	USA	North America	39293720	14550369	37.03	79	0
PRJNA338450	SRR4017768	Apis mellifera	paired end	2016	NA	2014	USA	North America	53299160	12294223	23.07	4	0
PRJNA338450	SRR4017769	Apis mellifera	paired end	2016	NA	2014	USA	North America	102196820	28760879	28.14	17883	0.02
PRJNA338450	SRR4017770	Apis mellifera	paired end	2016	NA	2014	USA	North America	64178156	19785203	30.83	119	0
PRJNA338450	SRR4017771	Apis mellifera	paired end	2016	NA	2014	USA	North America	72556254	9495695	13.09	15	0
PRJNA338450	SRR4017772	Apis mellifera	paired end	2016	NA	2014	USA	North America	64819558	10248725	15.81	37	0
PRJNA338450	SRR4017773	Apis mellifera	paired end	2016	NA	2014	USA	North America	53843376	12714344	23.61	83	0
PRJNA338450	SRR4017774	Apis mellifera	paired end	2016	NA	2014	USA	North America	85459052	15438921	18.07	14	0
PRJNA338450	SRR4017775	Apis mellifera	paired end	2016	NA	2014	USA	North America	63433196	33728	0.05	0	0
PRJNA338450	SRR4017776	Apis mellifera	paired end	2016	NA	2014	USA	North America	48010558	19889	0.04	0	0
PRJNA338450	SRR4017777	Apis mellifera	paired end	2016	NA	2014	USA	North America	40696180	8383	0.02	1	0
PRJNA338450	SRR4017778	Apis mellifera	paired end	2016	NA	2014	USA	North America	38067200	611	0	0	0
PRJNA338450	SRR4017779	Apis mellifera	paired end	2016	NA	2014	USA	North America	80053346	41430	0.05	5	0
PRJNA338450	SRR4017780	Apis mellifera	paired end	2016	NA	2014	USA	North America	71320450	33755	0.05	0	0
PRJNA338450	SRR4017781	Apis mellifera	paired end	2016	NA	2014	USA	North America	83495362	36851	0.04	1	0
PRJNA338450	SRR4017782	Apis mellifera	paired end	2016	NA	2014	USA	North America	49109706	11913	0.02	0	0
PRJNA338450	SRR4017783	Apis mellifera	paired end	2016	NA	2014	USA	North America	30620130	38238	0.12	0	0
PRJNA338450	SRR4017784	Apis mellifera	paired end	2016	NA	2014	USA	North America	79211868	19045	0.02	2	0
PRJNA338450	SRR4017785	Apis mellifera	paired end	2016	NA	2014	USA	North America	55394036	1844190	3.33	6	0
PRJNA338450	SRR4017786	Apis mellifera	paired end	2016	NA	2014	USA	North America	50717458	597549	1.18	411	0
PRJNA338450	SRR4017787	Apis mellifera	paired end	2016	NA	2014	USA	North America	44121178	890	0	0	0
PRJNA338450	SRR4017788	Apis mellifera	paired end	2016	NA	2014	USA	North America	58576326	43434	0.07	0	0
PRJNA338450	SRR4017792	Apis mellifera	paired end	2016	NA	2014	USA	North America	80346480	53256	0.07	2	0
PRJNA338450	SRR4017793	Apis mellifera	paired end	2016	NA	2014	USA	North America	44215084	13877	0.03	0	0
PRJNA338450	SRR4017794	Apis mellifera	paired end	2016	NA	2014	USA	North America	37483302	17521	0.05	0	0
PRJNA338450	SRR4017795	Apis mellifera	paired end	2016	NA	2014	USA	North America	24546714	33547	0.14	0	0
PRJNA338450	SRR4017796	Apis mellifera	paired end	2016	NA	2014	USA	North America	42247298	17534	0.04	1	0
PRJNA338450	SRR4017797	Apis mellifera	paired end	2016	NA	2014	USA	North America	58886050	400817	0.68	1	0
PRJNA338450	SRR4017798	Apis mellifera	paired end	2016	NA	2014	USA	North America	55910000	11440	0.02	0	0
PRJNA339509	SRR4051855	Apis mellifera	paired end	2016	NA	2014	NA	NA	562002374	17	0	0	0
PRJNA310321	SRR4063653	Apis mellifera	paired end	2016	NA	2014	China	Asia	331173934	0	0	0	0

PRJNA339906	SRR4084091	Apis mellifera	paired end	2017	2015	2015	China	Asia	34762568	13	0	0	0
PRJNA339906	SRR4084092	Apis mellifera	paired end	2017	2015	2015	China	Asia	32497468	4	0	0	0
PRJNA339906	SRR4084093	Apis mellifera	paired end	2017	2015	2015	China	Asia	29534074	8	0	0	0
PRJNA339906	SRR4084094	Apis mellifera	paired end	2017	2015	2015	China	Asia	30629132	28	0	0	0
PRJNA339906	SRR4084095	Apis mellifera	paired end	2017	2015	2015	China	Asia	30237796	12	0	0	0
PRJNA339906	SRR4084096	Apis mellifera	paired end	2017	2015	2015	China	Asia	28774864	35	0	0	0
PRJNA339906	SRR4084097	Apis mellifera	paired end	2017	2015	2015	China	Asia	29564924	23	0	0	0
PRJNA339906	SRR4084098	Apis mellifera	paired end	2017	2015	2015	China	Asia	30217832	25	0	0	0
PRJNA339906	SRR4084099	Apis mellifera	paired end	2017	2015	2015	China	Asia	47235172	16	0	0	0
PRJNA339906	SRR4084100	Apis mellifera	paired end	2017	2015	2015	China	Asia	26715638	4	0	0	0
PRJNA339906	SRR4084101	Apis mellifera	paired end	2017	2015	2015	China	Asia	28544942	3	0	0	0
PRJNA339906	SRR4084102	Apis mellifera	paired end	2017	2015	2015	China	Asia	32768988	12	0	0	0
PRJNA339509	SRR4096528	Apis mellifera	paired end	2016	NA	2014	NA	NA	590018372	0	0	0	0
PRJNA345404	SRR4343847	Apis mellifera	paired end	2017	NA	2015	NA	NA	11687658	0	0	2	0
PRJNA345404	SRR4343848	Apis mellifera	paired end	2017	NA	2015	NA	NA	11862194	0	0	7325	0.06
PRJNA104931	SRR445999	Apis mellifera	paired end	2012	NA	2010	USA	North America	427668328	124806752	29.18	1375	0
PRJNA104931	SRR446000	Apis mellifera	paired end	2012	NA	2010	USA	North America	590830686	79083063	13.39	1727	0
PRJNA104931	SRR446001	Apis mellifera	paired end	2012	NA	2010	USA	North America	303929248	73490388	24.18	547	0
PRJNA104931	SRR446002	Apis mellifera	paired end	2012	NA	2010	USA	North America	308963852	82055487	26.56	740	0
PRJNA104931	SRR446003	Apis mellifera	paired end	2012	NA	2010	USA	North America	277277252	43762427	15.78	348	0
PRJNA104931	SRR446004	Apis mellifera	paired end	2012	NA	2010	USA	North America	451987942	95519037	21.13	622	0
PRJNA104931	SRR446005	Apis mellifera	paired end	2012	NA	2010	USA	North America	357892462	104112427	29.09	1320	0
PRJNA104931	SRR446006	Apis mellifera	paired end	2012	NA	2010	USA	North America	386101840	58774161	15.22	786	0
PRJNA104931	SRR446007	Apis mellifera	paired end	2012	NA	2010	USA	North America	242309834	56448901	23.3	539	0
PRJNA104931	SRR446008	Apis mellifera	paired end	2012	NA	2010	USA	North America	329168752	86073701	26.15	809	0
PRJNA104931	SRR446009	Apis mellifera	paired end	2012	NA	2010	USA	North America	359328208	78031806	21.72	456	0
PRJNA104931	SRR446010	Apis mellifera	paired end	2012	NA	2010	USA	North America	278812238	73890307	26.5	566	0
PRJNA478161	SRR498622	Apis mellifera	paired end	2012	NA	2010	USA	North America	38230006	31798	0.08	1	0
PRJNA478161	SRR499808	Apis mellifera	paired end	2012	NA	2010	USA	North America	40959240	107390	0.26	5	0
PRJNA478161	SRR499882	Apis mellifera	paired end	2012	NA	2010	USA	North America	32336648	17936	0.06	1	0
PRJNA478161	SRR499883	Apis mellifera	paired end	2012	NA	2010	USA	North America	37025856	131895	0.36	3	0
PRJNA478161	SRR499919	Apis mellifera	paired end	2012	NA	2010	USA	North America	36316808	44064	0.12	3	0
PRJNA478161	SRR499920	Apis mellifera	paired end	2012	NA	2010	USA	North America	37847774	279155	0.74	2	0
PRJNA478161	SRR499992	Apis mellifera	paired end	2012	NA	2010	USA	North America	38449538	64077	0.17	0	0
PRJNA478161	SRR499993	Apis mellifera	paired end	2012	NA	2010	USA	North America	36704784	46502	0.13	0	0
PRJNA478161	SRR499994	Apis mellifera	paired end	2012	NA	2010	USA	North America	47488940	31340277	65.99	106	0
PRJNA478161	SRR499995	Apis mellifera	paired end	2012	NA	2010	USA	North America	34686976	28845	0.08	2	0
PRJNA357165	SRR5109820	Apis mellifera	paired end	2017	2013	2013	South_Africa	Africa	40214438	212	0	1095	0
PRJNA357165	SRR5109821	Apis mellifera	paired end	2017	2015	2015	Tonga	Oceania	42618838	3507	0.01	17593	0.04
PRJNA357165	SRR5109822	Apis mellifera	paired end	2017	2015	2015	Tonga	Oceania	37316706	1663396	4.46	8069220	21.62
PRJNA357165	SRR5109823	Apis mellifera	paired end	2017	2014	2014	Netherlands	Europe	86837530	1828689	2.11	61646744	70.99
PRJNA357165	SRR5109826	Apis mellifera	paired end	2017	2013	2013	South_Africa	Africa	84044582	4447	0.01	6683	0.01
PRJNA357165	SRR5109828	Apis mellifera	paired end	2017	2015	2015	Tonga	Oceania	42283492	491	0	2328	0.01
PRJNA357165	SRR5109829	Apis mellifera	paired end	2017	2014	2014	Netherlands	Europe	90787598	211133	0.23	43761301	48.2
PRJNA357165	SRR5109831	Apis mellifera	paired end	2017	2013	2013	South_Africa	Africa	37640156	1423	0	24	0
PRJNA357165	SRR5109832	Apis mellifera	paired end	2017	2013	2013	South_Africa	Africa	89462246	6885	0.01	8344	0.01
PRJNA357165	SRR5109833	Apis mellifera	paired end	2017	2013	2013	South_Africa	Africa	41030460	335	0	1744	0
PRJNA357165	SRR5109834	Apis mellifera	paired end	2017	2015	2015	Tonga	Oceania	38406846	327	0	1636	0
PRJNA357705	SRR5116303	Apis mellifera	paired end	2016	2012	2012	USA	North America	27672054	10134104	36.62	89	0
PRJNA357705	SRR5116304	Apis mellifera	paired end	2016	2012	2012	USA	North America	23939232	12896284	53.87	18152	0.08
PRJNA357705	SRR5116305	Apis mellifera	paired end	2016	2012	2012	USA	North America	49788778	1772431	3.56	36812169	73.94
PRJNA357705	SRR5116306	Apis mellifera	paired end	2016	2012	2012	USA	North America	49265160	36601550	74.29	669	0
PRJNA357705	SRR5116307	Apis mellifera	paired end	2016	2012	2012	USA	North America	28415146	23658438	83.26	14821	0.05
PRJNA357705	SRR5116308	Apis mellifera	paired end	2016	2012	2012	USA	North America	47278224	29764101	62.96	38030	0.08
PRJNA357523	SRR5117442	Apis mellifera	paired end	2017	2014	2014	Australia	Oceania	61193250	0	0	0	0
PRJNA357523	SRR5117443	Apis mellifera	paired end	2017	2014	2014	Australia	Oceania	94212890	0	0	0	0
PRJNA357523	SRR5117444	Apis mellifera	paired end	2017	2013	2013	Australia	Oceania	118366806	0	0	0	0
PRJNA357523	SRR5117445	Apis mellifera	paired end	2017	2013	2013	Australia	Oceania	114948672	0	0	0	0
PRJNA357523	SRR5117446	Apis mellifera	paired end	2017	2014	2014	Australia	Oceania	60012246	0	0	0	0
PRJNA357523	SRR5117447	Apis mellifera	paired end	2017	2014	2014	Australia	Oceania	105713768	0	0	0	0
PRJNA357523	SRR5117448	Apis mellifera	paired end	2017	2014	2014	Australia	Oceania	78728734	0	0	0	0
PRJNA357523	SRR5117449	Apis mellifera	paired end	2017	2014	2014	Australia	Oceania	96652584	0	0	0	0
PRJNA357523	SRR5117450	Apis mellifera	paired end	2017	2014	2014	Australia	Oceania	74494242	0	0	0	0
PRJNA359486	SRR5136448	Apis mellifera	paired end	2017	NA	2015	China	Asia	99634250	498	0	0	0
PRJNA359486	SRR5136449	Apis mellifera	paired end	2017	NA	2015	China	Asia	101848792	636	0	0	0
PRJNA359486	SRR5136450	Apis mellifera	paired end	2017	NA	2015	China	Asia	104160918	501	0	0	0
PRJNA359486	SRR5136451	Apis mellifera	paired end	2017	NA	2015	China	Asia	98132370	860950	0.88	0	0
PRJNA359486	SRR5136452	Apis mellifera	paired end	2017	NA	2015	China	Asia	128177728	2045	0	0	0
PRJNA359486	SRR5136453	Apis mellifera	paired end	2017	NA	2015	China	Asia	100160218	1045873	1.04	7	0
PRJNA359486	SRR5136454	Apis mellifera	paired end	2017	NA	2015	China	Asia	95494324	936192	0.98	0	0
PRJNA359486	SRR5136455	Apis mellifera	paired end	2017	NA	2015	China	Asia	83810010	19908	0.02	0	0
PRJNA359486	SRR5136456	Apis mellifera	paired end	2017	NA	2015	China	Asia	90240988	58	0	0	0
PRJNA359486	SRR5136457	Apis mellifera	paired end	2017	NA	2015	China	Asia	105607542	4832344	4.58	0	0
PRJNA359486	SRR5136458	Apis mellifera	paired end	2017	NA	2015	China	Asia	87929650	39243	0.04	0	0
PRJNA359486	SRR5136459	Apis mellifera	paired end	2017	NA	2015	China	Asia	148447384	32808630	22.1	82	0
PRJNA369540	SRR5223579	Apis mellifera	paired end	2019	NA	2017	NA	NA	167821922	14982803	8.93	1377	0
PRJNA369540	SRR5223580	Apis mellifera	paired end	2019	NA	2017	NA	NA	158879070	45155	0.03	99	0
PRJNA369540	SRR5223581	Apis mellifera	paired end	2019	NA	2017	NA	NA	159186198	14629606	9.19	291	0
PRJNA369540	SRR5223582	Apis mellifera	paired end	2019	NA	2017	NA	NA	168392988	8643	0.01	6	0
PRJNA380305	SRR5383925	Apis mellifera	paired end	2018	NA	2016	NA	NA	24077114	3911756	16.25	80	0
PRJNA380316	SRR5383927	Apis mellifera	paired end	2017	NA	2015	China	Asia	34732180	6157107	17.73	66	0
PRJNA380316	SRR5383937	Apis mellifera	paired end	2017	NA	2015	China	Asia	32930712	4733122	14.37	7	0
PRJNA380316	SRR5383939	Apis mellifera	paired end	2017	NA	2015	China	Asia	27041110	2081072	7.7	38	0
PRJNA380305	SRR5383942	Apis mellifera	paired end	2018	NA	2016	NA	NA	34732180	6157104	17.73	66	0
PRJNA380316	SRR5387735	Apis mellifera	paired end	2017	NA	2015	China	Asia	31507082	3551044	11.27	10	0
PRJNA380316	SRR5387737	Apis mellifera	paired end	2017	NA	2015	China	Asia	24077114	3911753	16.25	80	0
PRJNA380316	SRR5387738	Apis mellifera	paired end	2017	NA	2015	China	Asia	26538502	5533193	20.85	105	0
PRJNA383947	SRR5478349	Apis mellifera	paired end	2017	NA	2015	NA	NA	31589514	50	0	0	0
PRJNA383947	SRR5478350	Apis mellifera	paired end	2017	NA	2015	NA	NA	27106792	13	0	0	0
PRJNA386067	SRR5534311	Apis mellifera	paired end	2017	2014	2014	Korea	Asia	66531568	0	0	0	0
PRJNA386067	SRR5534312	Apis mellifera	paired end	2017	2014	2014	Korea	Asia	78712308	0	0	0	0
PRJNA386067	SRR5534313	Apis mellifera	paired end	2017	2014	2014	Korea	Asia	69209606	0	0	0	0
PRJNA386067	SRR5534314	Apis mellifera	paired end	2017	2014	2014	Korea	Asia	84122858	0	0	0	0
PRJNA386859	SRR5615334	Apis mellifera	paired end	2017	2016	2016	USA	North America	85983194	1365	0	3	0
PRJNA386859	SRR5615335	Apis mellifera	paired end	2017	2016	2016	USA	North America	75144328	1973	0	0	0
PRJNA386859	SRR5615342	Apis mellifera	paired end	2017	2016	2016	USA	North America	82332006	56187649	68.25	3581	0
PRJNA386859	SRR5615343	Apis mellifera	paired end	2017	2016	2016	USA	North America	40006034	25598677	63.99	2232	0.01
NA	SRR566980	Apis mellifera	paired end	2012	NA	2010	NA	NA	12976874	11	0	0	0
NA	SRR567163	Apis mellifera	paired end	2012	NA	2010	NA	NA	12976874	9	0	0	0
NA	SRR567629	Apis mellifera	paired end	2012	NA	2010	NA	NA	12976874	13	0	0	0
NA	SRR567630	Apis mellifera	paired end	2012	NA	2010	NA	NA	12976874	7	0	0	0
NA	SRR567631	Apis mellifera	paired end	2012	NA	2010	NA	NA	12976874	9	0	0	0
NA	SRR567646	Apis mellifera	paired end	2012	NA	2010	NA	NA	12976874	2	0	0	0
NA	SRR567647	Apis mellifera	paired end	2012	NA	2010	NA	NA	12976874	10	0	0	0
NA	SRR567648	Apis mellifera	paired end	2012	NA	2010	NA	NA	12976874	12	0	0	0
NA	SRR567649	Apis mellifera	paired end	2012	NA	2010	NA	NA	12976874	7	0	0	0

PRJNA175445	SRR584147	Apis mellifera	paired end	2013	2011	2011	USA	North America	32012050	5336050	16.67	8742	0.03
PRJNA175445	SRR584156	Apis mellifera	paired end	2013	2011	2011	USA	North America	16131564	2278967	14.13	3235	0.02
PRJNA175445	SRR584160	Apis mellifera	paired end	2013	2011	2011	USA	North America	16972664	3858278	22.73	7407	0.04
PRJNA175445	SRR584163	Apis mellifera	paired end	2013	2011	2011	USA	North America	24240944	4363381	18	6987	0.03
PRJNA175445	SRR584169	Apis mellifera	paired end	2013	2011	2011	USA	North America	22318092	1447659	6.49	2785	0.01
PRJNA175445	SRR584172	Apis mellifera	paired end	2013	2011	2011	USA	North America	21556600	4442527	20.61	14330	0.07
PRJNA175445	SRR584177	Apis mellifera	paired end	2013	2011	2011	USA	North America	19401608	3303238	17.03	4725	0.02
PRJNA175445	SRR584184	Apis mellifera	paired end	2013	2011	2011	USA	North America	51410174	3150595	6.13	7542	0.01
PRJNA175445	SRR584187	Apis mellifera	paired end	2013	2011	2011	USA	North America	30871554	2877227	9.32	6252	0.02
PRJNA175445	SRR584189	Apis mellifera	paired end	2013	2011	2011	USA	North America	21147498	2729020	12.9	7519	0.04
PRJNA175445	SRR584192	Apis mellifera	paired end	2013	2011	2011	USA	North America	17205992	3368992	19.58	8250	0.05
PRJNA175445	SRR585833	Apis mellifera	paired end	2013	2011	2011	USA	North America	17642008	2778380	15.75	7088	0.04
PRJNA406998	SRR6031640	Apis mellifera	paired end	2018	2017	2017	China	Asia	208589832	131	0	0	0
PRJNA406998	SRR6031641	Apis mellifera	paired end	2018	2017	2017	China	Asia	173097006	191	0	0	0
PRJNA406998	SRR6031642	Apis mellifera	paired end	2018	2017	2017	China	Asia	160844082	170	0	0	0
PRJNA406998	SRR6031643	Apis mellifera	paired end	2018	2017	2017	China	Asia	129878194	16	0	0	0
PRJNA406998	SRR6031644	Apis mellifera	paired end	2018	2017	2017	China	Asia	113683898	2	0	0	0
PRJNA406998	SRR6031645	Apis mellifera	paired end	2018	2017	2017	China	Asia	160537248	1	0	0	0
PRJNA406998	SRR6031646	Apis mellifera	paired end	2018	2017	2017	China	Asia	149230808	9	0	0	0
PRJNA406998	SRR6031647	Apis mellifera	paired end	2018	2017	2017	China	Asia	131386354	12	0	0	0
PRJNA406998	SRR6031648	Apis mellifera	paired end	2018	2017	2017	China	Asia	152323278	1322	0	0	0
PRJNA406998	SRR6031649	Apis mellifera	paired end	2018	2017	2017	China	Asia	200417896	1177	0	0	0
PRJNA406998	SRR6031650	Apis mellifera	paired end	2018	2017	2017	China	Asia	126667596	943	0	0	0
PRJNA406998	SRR6031651	Apis mellifera	paired end	2018	2017	2017	China	Asia	249473666	208	0	0	0
PRJNA407316	SRR6047306	Apis mellifera	paired end	2018	NA	2016	China	Asia	56359504	72	0	0	0
PRJNA407316	SRR6047307	Apis mellifera	paired end	2018	NA	2016	China	Asia	79136518	39	0	0	0
PRJNA407316	SRR6047308	Apis mellifera	paired end	2018	NA	2016	China	Asia	55776806	54	0	0	0
PRJNA407316	SRR6047309	Apis mellifera	paired end	2018	NA	2016	China	Asia	60368568	155478	0.26	0	0
PRJNA407316	SRR6047310	Apis mellifera	paired end	2018	NA	2016	China	Asia	75071086	247	0	0	0
PRJNA407316	SRR6047311	Apis mellifera	paired end	2018	NA	2016	China	Asia	58556824	246397	0.42	1	0
PRJNA407316	SRR6047312	Apis mellifera	paired end	2018	NA	2016	China	Asia	59872588	103800	0.17	0	0
PRJNA407316	SRR6047313	Apis mellifera	paired end	2018	NA	2016	China	Asia	62554784	47	0	0	0
PRJNA407316	SRR6047314	Apis mellifera	paired end	2018	NA	2016	China	Asia	56668426	29	0	0	0
PRJNA407316	SRR6047315	Apis mellifera	paired end	2018	NA	2016	China	Asia	59045000	916953	1.55	0	0
PRJNA407316	SRR6047316	Apis mellifera	paired end	2018	NA	2016	China	Asia	60314752	1277	0	0	0
PRJNA407316	SRR6047317	Apis mellifera	paired end	2018	NA	2016	China	Asia	59057944	3696913	6.26	16	0
PRJNA418780	SRR6326775	Apis mellifera	paired end	2018	2015	2015	USA	North America	26930730	101	0	0	0
PRJNA418780	SRR6326776	Apis mellifera	paired end	2018	2015	2015	USA	North America	24962760	103034	0.41	1	0
PRJNA418780	SRR6326777	Apis mellifera	paired end	2018	2015	2015	USA	North America	26033666	98	0	0	0
PRJNA418780	SRR6326778	Apis mellifera	paired end	2018	2015	2015	USA	North America	26502548	322122	1.22	1	0
PRJNA418780	SRR6326779	Apis mellifera	paired end	2018	2015	2015	USA	North America	23805036	75	0	0	0
PRJNA418780	SRR6326780	Apis mellifera	paired end	2018	2015	2015	USA	North America	27730112	81	0	0	0
PRJNA429508	SRR6489887	Apis mellifera	paired end	2019	2015	2015	USA	North America	43163710	20764290	48.11	17	0
PRJNA429508	SRR6489888	Apis mellifera	paired end	2019	2015	2015	USA	North America	58285804	5774	0.01	0	0
PRJNA429508	SRR6489890	Apis mellifera	paired end	2019	2015	2015	USA	North America	44723652	28064782	62.75	10	0
PRJNA429508	SRR6489891	Apis mellifera	paired end	2019	2015	2015	USA	North America	40996312	30484	0.07	0	0
PRJNA429508	SRR6489892	Apis mellifera	paired end	2019	2015	2015	USA	North America	39278566	28327860	72.12	28	0
PRJNA429508	SRR6489893	Apis mellifera	paired end	2019	2015	2015	USA	North America	43233678	22747	0.05	0	0
PRJNA429508	SRR6489894	Apis mellifera	paired end	2019	2015	2015	USA	North America	50299286	4932	0.01	0	0
PRJNA429508	SRR6489895	Apis mellifera	paired end	2019	2015	2015	USA	North America	44472368	1255	0	0	0
PRJNA429508	SRR6489896	Apis mellifera	paired end	2019	2015	2015	USA	North America	40160214	16423764	40.9	3	0
PRJNA429508	SRR6489897	Apis mellifera	paired end	2019	2015	2015	USA	North America	43965288	269301	0.61	3	0
PRJNA429508	SRR6489915	Apis mellifera	paired end	2019	2015	2015	USA	North America	42313086	26828405	63.4	16	0
PRJNA429508	SRR6489916	Apis mellifera	paired end	2019	2015	2015	USA	North America	40298130	2755	0.01	0	0
PRJNA429508	SRR6489917	Apis mellifera	paired end	2019	2015	2015	USA	North America	45326130	956	0	0	0
PRJNA429508	SRR6489918	Apis mellifera	paired end	2019	2015	2015	USA	North America	42325700	1135	0	0	0
PRJNA429508	SRR6489919	Apis mellifera	paired end	2019	2015	2015	USA	North America	42521258	3318	0.01	0	0
PRJNA429508	SRR6489920	Apis mellifera	paired end	2019	2015	2015	USA	North America	42938196	7111	0.02	0	0
PRJNA429508	SRR6489921	Apis mellifera	paired end	2019	2015	2015	USA	North America	38790148	17503	0.05	0	0
PRJNA429508	SRR6489922	Apis mellifera	paired end	2019	2015	2015	USA	North America	36641658	1020	0	0	0
PRJNA434146	SRR6727829	Apis mellifera	paired end	2018	NA	2016	NA	NA	14345520	0	0	0	0
PRJNA434146	SRR6727830	Apis mellifera	paired end	2018	NA	2016	NA	NA	14110580	0	0	0	0
PRJNA434146	SRR6727831	Apis mellifera	paired end	2018	NA	2016	NA	NA	14500834	0	0	0	0
PRJNA434146	SRR6727832	Apis mellifera	paired end	2018	NA	2016	NA	NA	14297964	0	0	0	0
PRJNA434146	SRR6727833	Apis mellifera	paired end	2018	NA	2016	NA	NA	14494274	0	0	0	0
PRJNA434146	SRR6727834	Apis mellifera	paired end	2018	NA	2016	NA	NA	14344702	0	0	0	0
PRJNA434146	SRR6727835	Apis mellifera	paired end	2018	NA	2016	NA	NA	14634746	0	0	0	0
PRJNA434146	SRR6727836	Apis mellifera	paired end	2018	NA	2016	NA	NA	14501614	0	0	0	0
PRJNA434146	SRR6727837	Apis mellifera	paired end	2018	NA	2016	NA	NA	13451964	0	0	0	0
PRJNA434146	SRR6727838	Apis mellifera	paired end	2018	NA	2016	NA	NA	13268808	0	0	0	0
PRJNA434146	SRR6727839	Apis mellifera	paired end	2018	NA	2016	NA	NA	13605474	0	0	0	0
PRJNA434146	SRR6727840	Apis mellifera	paired end	2018	NA	2016	NA	NA	13418334	0	0	0	0
PRJNA434146	SRR6727841	Apis mellifera	paired end	2018	NA	2016	NA	NA	14056768	0	0	0	0
PRJNA434146	SRR6727842	Apis mellifera	paired end	2018	NA	2016	NA	NA	13865456	0	0	0	0
PRJNA434146	SRR6727843	Apis mellifera	paired end	2018	NA	2016	NA	NA	14224416	0	0	0	0
PRJNA434146	SRR6727844	Apis mellifera	paired end	2018	NA	2016	NA	NA	14043804	0	0	0	0
PRJNA434146	SRR6727845	Apis mellifera	paired end	2018	NA	2016	NA	NA	12212406	0	0	0	0
PRJNA434146	SRR6727846	Apis mellifera	paired end	2018	NA	2016	NA	NA	12059184	0	0	0	0
PRJNA434146	SRR6727847	Apis mellifera	paired end	2018	NA	2016	NA	NA	12333314	0	0	0	0
PRJNA434146	SRR6727848	Apis mellifera	paired end	2018	NA	2016	NA	NA	12195416	0	0	0	0
PRJNA434146	SRR6727849	Apis mellifera	paired end	2018	NA	2016	NA	NA	12952440	0	0	0	0
PRJNA434146	SRR6727850	Apis mellifera	paired end	2018	NA	2016	NA	NA	12715912	0	0	0	0
PRJNA434146	SRR6727851	Apis mellifera	paired end	2018	NA	2016	NA	NA	12999334	0	0	0	0
PRJNA434146	SRR6727852	Apis mellifera	paired end	2018	NA	2016	NA	NA	12849116	0	0	0	0
PRJNA434146	SRR6727853	Apis mellifera	paired end	2018	NA	2016	NA	NA	14062306	0	0	0	0
PRJNA434146	SRR6727854	Apis mellifera	paired end	2018	NA	2016	NA	NA	13828908	0	0	0	0
PRJNA434146	SRR6727855	Apis mellifera	paired end	2018	NA	2016	NA	NA	14210140	0	0	0	0
PRJNA434146	SRR6727856	Apis mellifera	paired end	2018	NA	2016	NA	NA	13999284	0	0	0	0
PRJNA434146	SRR6727857	Apis mellifera	paired end	2018	NA	2016	NA	NA	13677078	0	0	0	0
PRJNA434146	SRR6727858	Apis mellifera	paired end	2018	NA	2016	NA	NA	13447088	0	0	0	0
PRJNA641576	SRR12080862	Apis mellifera	paired end	2020	2019	2019	China	Asia	42404432	26	0	0	0
PRJNA641576	SRR12080863	Apis mellifera	paired end	2020	2019	2019	China	Asia	41368044	37770	0.09	0	0
PRJNA641576	SRR12080864	Apis mellifera	paired end	2020	2019	2019	China	Asia	44996028	976	0	0	0
PRJNA641576	SRR12080865	Apis mellifera	paired end	2020	2019	2019	China	Asia	47061750	3	0	0	0
PRJNA641576	SRR12080866	Apis mellifera	paired end	2020	2019	2019	China	Asia	46558518	0	0	0	0
PRJNA641576	SRR12080867	Apis mellifera	paired end	2020	2019	2019	China	Asia	41695556	6	0	0	0
PRJNA641576	SRR12080868	Apis mellifera	paired end	2020	2019	2019	China	Asia	43297720	15	0	0	0
PRJNA641576	SRR12080869	Apis mellifera	paired end	2020	2019	2019	China	Asia	46501782	28	0	0	0
PRJNA641576	SRR12080870	Apis mellifera	paired end	2020	2019	2019	China	Asia	45976724	52	0	0	0
PRJNA434146	SRR6727859	Apis mellifera	paired end	2018	NA	2016	NA	NA	13823678	0	0	0	0
PRJNA434146	SRR6727860	Apis mellifera	paired end	2018	NA	2016	NA	NA	13614450	0	0	0	0
PRJNA436408	SRR6793737	Apis mellifera	paired end	2020	NA	2018	China	Asia	56873770	7644468	13.44	2	0
PRJNA436408	SRR6793738	Apis mellifera	paired end	2020	NA	2018	China	Asia	45353108	486868	1.07	0	0
PRJNA436408	SRR6793739	Apis mellifera	paired end	2020	NA	2018	China	Asia	56547932	2279936	4.03	1	0
PRJNA436408	SRR6793740	Apis mellifera	paired end	2020	NA	2018	China	Asia	52685948	4290930	8.14	0	0
PRJNA436408	SRR6793741	Apis mellifera	paired end	2020	NA	2018	China	Asia	53674326	6473418	12.06	6	0

PRJNA436408	SRR6793742	Apis mellifera	paired end	2020	NA	2018	China	Asia	58869218	3955790	6.72	1	0
PRJNA437728	SRR6823683	Apis mellifera	paired end	2018	2004	2004	Syria/Jordan	Asia	69703502	24096054	34.57	7429642	10.66
PRJNA437730	SRR6823685	Apis mellifera	paired end	2018	2014	2014	Algeria	Africa	44877638	13126495	29.25	23941	0.05
PRJNA438231	SRR6833955	Apis mellifera	paired end	2018	2015	2015	NA	NA	58364376	153980	0.26	4458380	7.64
PRJNA438231	SRR6833956	Apis mellifera	paired end	2018	2015	2015	South_Africa	Africa	67105306	168	0	25624	0.04
PRJNA438231	SRR6833957	Apis mellifera	paired end	2018	2015	2015	South_Africa	Africa	55592538	1025	0	111724	0.2
PRJNA438231	SRR6833958	Apis mellifera	paired end	2018	2015	2015	South_Africa	Africa	65872128	262	0	7372	0.01
PRJNA438231	SRR6833959	Apis mellifera	paired end	2018	2015	2015	South_Africa	Africa	54684282	680	0	51823	0.09
PRJNA438231	SRR6833960	Apis mellifera	paired end	2018	2015	2015	South_Africa	Africa	67004760	592090	0.88	15998936	23.88
PRJNA438231	SRR6833961	Apis mellifera	paired end	2018	2015	2015	South_Africa	Africa	65956034	925	0	7925744	12.02
PRJNA438231	SRR6833962	Apis mellifera	paired end	2018	2015	2015	South_Africa	Africa	73950658	373892	0.51	10997739	14.87
PRJNA438231	SRR6833963	Apis mellifera	paired end	2018	2015	2015	South_Africa	Africa	60409760	612	0	63047	0.1
PRJNA438231	SRR6833964	Apis mellifera	paired end	2018	2015	2015	South_Africa	Africa	76350296	178	0	4741	0.01
PRJNA438231	SRR6833965	Apis mellifera	paired end	2018	2015	2015	South_Africa	Africa	70905126	134	0	3799	0.01
PRJNA438231	SRR6833966	Apis mellifera	paired end	2018	2015	2015	South_Africa	Africa	64880868	562	0	55251	0.09
PRJNA442330	SRR6880250	Apis mellifera	paired end	2019	2015	2015	USA	North America	20268910	13853881	68.35	3402	0.02
PRJNA442330	SRR6880251	Apis mellifera	paired end	2019	2015	2015	USA	North America	23520970	17388748	73.93	2181665	9.28
PRJNA442330	SRR6880252	Apis mellifera	paired end	2019	2015	2015	USA	North America	29621190	58224	0.2	594	0
PRJNA442330	SRR6880253	Apis mellifera	paired end	2019	2015	2015	USA	North America	19788698	14014104	70.82	2933	0.01
PRJNA442330	SRR6880254	Apis mellifera	paired end	2019	2015	2015	USA	North America	24220668	16794176	69.34	4540	0.02
PRJNA442330	SRR6880255	Apis mellifera	paired end	2019	2015	2015	USA	North America	22098768	15889963	71.9	4095	0.02
PRJNA442330	SRR6880256	Apis mellifera	paired end	2019	2015	2015	USA	North America	27954156	20633718	73.81	8335	0.03
PRJNA442330	SRR6880257	Apis mellifera	paired end	2019	2015	2015	USA	North America	16817192	8385374	49.86	1605	0.01
PRJNA442330	SRR6880258	Apis mellifera	paired end	2019	2015	2015	USA	North America	33340186	29020311	87.04	22069	0.07
PRJNA442330	SRR6880259	Apis mellifera	paired end	2019	2015	2015	USA	North America	28515310	24762776	86.84	13666	0.05
PRJNA442330	SRR6880260	Apis mellifera	paired end	2019	2015	2015	USA	North America	31132666	18653295	59.92	6541042	21.01
PRJNA442330	SRR6880261	Apis mellifera	paired end	2019	2015	2015	USA	North America	29510186	12520078	42.43	4545	0.02
PRJNA442330	SRR6880262	Apis mellifera	paired end	2019	2015	2015	USA	North America	3917742	66918	1.71	1289	0.03
PRJNA442330	SRR6880263	Apis mellifera	paired end	2019	2015	2015	USA	North America	21678462	16020004	73.9	3649	0.02
PRJNA442330	SRR6880264	Apis mellifera	paired end	2019	2015	2015	USA	North America	19538436	15847798	81.11	3781	0.02
PRJNA442330	SRR6880265	Apis mellifera	paired end	2019	2015	2015	USA	North America	25895368	18897515	72.98	3809	0.01
PRJNA442330	SRR6880266	Apis mellifera	paired end	2019	2015	2015	USA	North America	25419724	17699500	69.63	7322	0.03
PRJNA442330	SRR6880267	Apis mellifera	paired end	2019	2015	2015	USA	North America	25090682	19271451	76.81	22079	0.09
PRJNA442330	SRR6880268	Apis mellifera	paired end	2019	2015	2015	USA	North America	21582152	15692490	72.71	4107	0.02
PRJNA442330	SRR6880269	Apis mellifera	paired end	2019	2015	2015	USA	North America	27536872	9990313	36.28	2407	0.01
PRJNA442330	SRR6880270	Apis mellifera	paired end	2019	2015	2015	USA	North America	23441522	15394427	65.67	3405426	14.53
PRJNA442330	SRR6880271	Apis mellifera	paired end	2019	2015	2015	USA	North America	24613614	18331118	74.48	1294868	5.26
PRJNA442330	SRR6880272	Apis mellifera	paired end	2019	2015	2015	USA	North America	25752396	15878519	61.66	4008	0.02
PRJNA442330	SRR6880273	Apis mellifera	paired end	2019	2015	2015	USA	North America	26685764	19558876	73.29	1012	0
PRJNA442330	SRR6880274	Apis mellifera	paired end	2019	2015	2015	USA	North America	25508960	21864221	85.71	10584	0.04
PRJNA442330	SRR6880275	Apis mellifera	paired end	2019	2015	2015	USA	North America	18177778	15336585	84.37	11024	0.06
PRJNA442330	SRR6880276	Apis mellifera	paired end	2019	2015	2015	USA	North America	22824984	13677844	59.92	3856	0.02
PRJNA442330	SRR6880277	Apis mellifera	paired end	2019	2015	2015	USA	North America	23104294	4475999	19.37	1200	0.01
PRJNA442330	SRR6880278	Apis mellifera	paired end	2019	2015	2015	USA	North America	27661150	85815	0.31	105	0
PRJNA442330	SRR6880279	Apis mellifera	paired end	2019	2015	2015	USA	North America	24972816	301235	1.21	376	0
PRJNA442330	SRR6880280	Apis mellifera	paired end	2019	2015	2015	USA	North America	19000250	12290184	64.68	5492	0.03
PRJNA442330	SRR6880281	Apis mellifera	paired end	2019	2015	2015	USA	North America	19178428	6363479	33.18	2196	0.01
PRJNA442330	SRR6880282	Apis mellifera	paired end	2019	2015	2015	USA	North America	18404646	8442088	45.87	21370	0.12
PRJNA442330	SRR6880283	Apis mellifera	paired end	2019	2015	2015	USA	North America	19602438	12776097	65.18	14892	0.08
PRJNA442330	SRR6880284	Apis mellifera	paired end	2019	2015	2015	USA	North America	22396172	17265128	77.09	5961	0.03
PRJNA442330	SRR6880285	Apis mellifera	paired end	2019	2015	2015	USA	North America	21717778	15535581	71.53	762324	3.51
PRJNA442330	SRR6880286	Apis mellifera	paired end	2019	2015	2015	USA	North America	23308460	8486	0.04	27	0
PRJNA442330	SRR6880287	Apis mellifera	paired end	2019	2015	2015	USA	North America	20273876	10800279	53.27	13961	0.07
PRJNA442330	SRR6880288	Apis mellifera	paired end	2019	2015	2015	USA	North America	26010980	67852	0.26	145	0
PRJNA442330	SRR6880289	Apis mellifera	paired end	2019	2015	2015	USA	North America	23184972	20152	0.09	53	0
PRJNA442330	SRR6880290	Apis mellifera	paired end	2019	2015	2015	USA	North America	26993328	11486803	42.55	15613	0.06
PRJNA442330	SRR6880291	Apis mellifera	paired end	2019	2015	2015	USA	North America	25506054	14430	0.06	39	0
PRJNA442330	SRR6880292	Apis mellifera	paired end	2019	2015	2015	USA	North America	19176090	15767854	82.23	8459	0.04
PRJNA442330	SRR6880293	Apis mellifera	paired end	2019	2015	2015	USA	North America	23697780	19819420	83.63	10714	0.05
PRJNA442330	SRR6880294	Apis mellifera	paired end	2019	2015	2015	USA	North America	27025460	18392587	68.06	14932	0.06
PRJNA442330	SRR6880295	Apis mellifera	paired end	2019	2015	2015	USA	North America	26070984	9975535	38.26	25910	0.1
PRJNA442330	SRR6880296	Apis mellifera	paired end	2019	2015	2015	USA	North America	20604302	16264508	78.94	10924	0.05
PRJNA442330	SRR6880297	Apis mellifera	paired end	2019	2015	2015	USA	North America	26682450	18717230	70.15	4139	0.02
PRJNA478908	SRR7460780	Apis mellifera	paired end	2019	NA	2017	NA	NA	60532264	0	0	0	0
PRJNA478908	SRR7460781	Apis mellifera	paired end	2019	NA	2017	NA	NA	56132332	2	0	0	0
PRJNA478908	SRR7460782	Apis mellifera	paired end	2019	NA	2017	NA	NA	53748498	0	0	0	0
PRJNA478908	SRR7460783	Apis mellifera	paired end	2019	NA	2017	NA	NA	57487168	1	0	0	0
PRJNA478908	SRR7460784	Apis mellifera	paired end	2019	NA	2017	NA	NA	54216268	0	0	0	0
PRJNA478908	SRR7460785	Apis mellifera	paired end	2019	NA	2017	NA	NA	54001658	0	0	0	0
PRJNA478908	SRR7460786	Apis mellifera	paired end	2019	NA	2017	NA	NA	54615604	1	0	0	0
PRJNA478908	SRR7460787	Apis mellifera	paired end	2019	NA	2017	NA	NA	47131026	0	0	0	0
PRJNA478908	SRR7460788	Apis mellifera	paired end	2019	NA	2017	NA	NA	56494396	0	0	0	0
PRJNA478908	SRR7460789	Apis mellifera	paired end	2019	NA	2017	NA	NA	49210692	0	0	0	0
PRJNA478908	SRR7460790	Apis mellifera	paired end	2019	NA	2017	NA	NA	55782734	0	0	0	0
PRJNA478908	SRR7460791	Apis mellifera	paired end	2019	NA	2017	NA	NA	56071232	0	0	0	0
PRJNA478908	SRR7460792	Apis mellifera	paired end	2019	NA	2017	NA	NA	56564188	0	0	0	0
PRJNA478908	SRR7460793	Apis mellifera	paired end	2019	NA	2017	NA	NA	52276120	0	0	0	0
PRJNA478908	SRR7460794	Apis mellifera	paired end	2019	NA	2017	NA	NA	48975296	0	0	0	0
PRJNA478908	SRR7460795	Apis mellifera	paired end	2019	NA	2017	NA	NA	52667940	1	0	0	0
PRJNA478908	SRR7460796	Apis mellifera	paired end	2019	NA	2017	NA	NA	54600532	0	0	0	0
PRJNA478908	SRR7460797	Apis mellifera	paired end	2019	NA	2017	NA	NA	52751508	5	0	0	0
PRJNA478908	SRR7460798	Apis mellifera	paired end	2019	NA	2017	NA	NA	47431192	0	0	0	0
PRJNA478908	SRR7460799	Apis mellifera	paired end	2019	NA	2017	NA	NA	56993022	4	0	0	0
PRJNA478908	SRR7460800	Apis mellifera	paired end	2019	NA	2017	NA	NA	67819870	0	0	0	0
PRJNA478908	SRR7460801	Apis mellifera	paired end	2019	NA	2017	NA	NA	56873292	0	0	0	0
PRJNA478908	SRR7460802	Apis mellifera	paired end	2019	NA	2017	NA	NA	57667608	0	0	0	0
PRJNA478908	SRR7460803	Apis mellifera	paired end	2019	NA	2017	NA	NA	57099948	0	0	0	0
PRJNA480381	SRR7513056	Apis mellifera	paired end	2019	NA	2017	China	Asia	100829996	24802863	24.6	51	0
PRJNA480381	SRR7513057	Apis mellifera	paired end	2019	NA	2017	China	Asia	59983080	1481152	2.47	1	0
PRJNA480381	SRR7513058	Apis mellifera	paired end	2019	NA	2017	China	Asia	50196234	998392	1.99	2	0
PRJNA480381	SRR7513059	Apis mellifera	paired end	2019	NA	2017	China	Asia	51967326	620	0	0	0
PRJNA480381	SRR7513060	Apis mellifera	paired end	2019	NA	2017	China	Asia	102482840	16581208	16.18	28	0
PRJNA480381	SRR7513061	Apis mellifera	paired end	2019	NA	2017	China	Asia	60403556	2587167	4.28	5	0
PRJNA486418	SRR7716628	Apis mellifera	paired end	2019	NA	2017	NA	NA	118429032	23	0	0	0
PRJNA486418	SRR7716629	Apis mellifera	paired end	2019	NA	2017	NA	NA	114555190	4	0	0	0
PRJNA486418	SRR7716630	Apis mellifera	paired end	2019	NA	2017	NA	NA	113592540	36	0	0	0
PRJNA486418	SRR7716631	Apis mellifera	paired end	2019	NA	2017	NA	NA	108517062	2	0	0	0
PRJNA486418	SRR7716632	Apis mellifera	paired end	2019	NA	2017	NA	NA	108687050	8	0	0	0
PRJNA486418	SRR7716633	Apis mellifera	paired end	2019	NA	2017	NA	NA	122967148	6	0	0	0
PRJNA486418	SRR7716634	Apis mellifera	paired end	2019	NA	2017	NA	NA	143095384	0	0	0	0
PRJNA486418	SRR7716635	Apis mellifera	paired end	2019	NA	2017	NA	NA	117742600	8	0	0	0
PRJNA486418	SRR7716636	Apis mellifera	paired end	2019	NA	2017	NA	NA	123126070	2	0	0	0
PRJNA488619	SRR7770429	Apis mellifera	paired end	2019	NA	2017	China	Asia	112738988	3991	0	0	0
PRJNA488619	SRR7770430	Apis mellifera	paired end	2019	NA	2017	China	Asia	98301624	2970	0	0	0
PRJNA488619	SRR7770432	Apis mellifera	paired end	2019	NA	2017	China	Asia	110986460	6626404	5.97	2337	0
PRJNA488619	SRR7770433	Apis mellifera	paired end	2019	NA	2017	China	Asia	91011854	3199522	3.52	856	0

PRJNA488619	SRR7770435	Apis mellifera	paired end	2019	NA	2017	China	Asia	112861484	3132032	2.78	113	0
PRJNA488619	SRR7770436	Apis mellifera	paired end	2019	NA	2017	China	Asia	100326436	147913	0.15	0	0
PRJNA490324	SRR7829127	Apis mellifera	paired end	2019	2014	2014	Brazil	South America	69766364	0	0	0	0
PRJNA490324	SRR7829129	Apis mellifera	paired end	2019	2014	2014	Brazil	South America	77068018	0	0	0	0
PRJNA490324	SRR7829130	Apis mellifera	paired end	2019	2014	2014	Brazil	South America	97479876	0	0	0	0
PRJNA490324	SRR7829131	Apis mellifera	paired end	2019	2014	2014	Brazil	South America	100562906	0	0	0	0
PRJNA490324	SRR7829132	Apis mellifera	paired end	2019	2014	2014	Brazil	South America	58393140	0	0	0	0
PRJNA490324	SRR7829133	Apis mellifera	paired end	2019	2014	2014	Brazil	South America	78422602	0	0	0	0
PRJNA490324	SRR7829134	Apis mellifera	paired end	2019	2014	2014	Brazil	South America	82227144	0	0	0	0
PRJNA490324	SRR7829135	Apis mellifera	paired end	2019	2014	2014	Brazil	South America	48047516	0	0	0	0
PRJNA490324	SRR7829136	Apis mellifera	paired end	2019	2014	2014	Brazil	South America	80922592	0	0	0	0
PRJNA194157	SRR801788	Apis mellifera	paired end	2013	NA	2011	NA	NA	28646970	1903	0.01	111	0
PRJNA194157	SRR801844	Apis mellifera	paired end	2013	NA	2011	NA	NA	27984962	62	0	18	0
PRJNA194157	SRR802386	Apis mellifera	paired end	2013	NA	2011	NA	NA	33738484	182	0	59	0
PRJNA194157	SRR802538	Apis mellifera	paired end	2013	NA	2011	NA	NA	36193280	191	0	0	0
PRJNA194157	SRR802556	Apis mellifera	paired end	2013	NA	2011	NA	NA	35517036	1	0	0	0
PRJNA194157	SRR802899	Apis mellifera	paired end	2013	NA	2011	NA	NA	24636090	76	0	20	0
PRJNA194157	SRR806508	Apis mellifera	paired end	2013	NA	2011	NA	NA	92004718	279	0	72	0
PRJNA194157	SRR806550	Apis mellifera	paired end	2013	NA	2011	NA	NA	124193172	148	0	28	0
PRJNA194157	SRR806693	Apis mellifera	paired end	2013	NA	2011	NA	NA	85795228	396	0	2	0
PRJNA194157	SRR806701	Apis mellifera	paired end	2013	NA	2011	NA	NA	64517386	528	0	51	0
PRJNA194157	SRR806709	Apis mellifera	paired end	2013	NA	2011	NA	NA	90233990	19	0	2	0
PRJNA194157	SRR806711	Apis mellifera	paired end	2013	NA	2011	NA	NA	90233990	19	0	2	0
PRJNA510543	SRR8335255	Apis mellifera	paired end	2018	2017	2017	China	Asia	57832244	225	0	0	0
PRJNA510543	SRR8335256	Apis mellifera	paired end	2018	2017	2017	China	Asia	55803140	1951244	3.5	0	0
PRJNA510543	SRR8335257	Apis mellifera	paired end	2018	2017	2017	China	Asia	54772400	3794	0.01	0	0
PRJNA510543	SRR8335258	Apis mellifera	paired end	2018	2017	2017	China	Asia	61526972	55	0	0	0
PRJNA510495	SRR8335305	Apis mellifera	paired end	2018	2017	2017	China	Asia	28076708	9481	0.03	0	0
PRJNA510495	SRR8335306	Apis mellifera	paired end	2018	2017	2017	China	Asia	27293038	3704	0.01	0	0
PRJNA510495	SRR8335307	Apis mellifera	paired end	2018	2017	2017	China	Asia	44005106	2973518	6.76	1479	0
PRJNA510495	SRR8335308	Apis mellifera	paired end	2018	2017	2017	China	Asia	43702512	4130377	9.45	1997	0
PRJNA510495	SRR8335309	Apis mellifera	paired end	2018	2017	2017	China	Asia	27873590	963614	3.46	13	0
PRJNA510495	SRR8335310	Apis mellifera	paired end	2018	2017	2017	China	Asia	27735218	838	0	0	0
PRJNA510495	SRR8335311	Apis mellifera	paired end	2018	2017	2017	China	Asia	27866854	1733	0.01	0	0
PRJNA510495	SRR8335312	Apis mellifera	paired end	2018	2017	2017	China	Asia	27316582	94412	0.35	0	0
PRJNA201395	SRR849282	Apis mellifera	paired end	2013	2011	2011	China	Asia	48971186	0	0	0	0
PRJNA201395	SRR849283	Apis mellifera	paired end	2013	2011	2011	China	Asia	49358642	0	0	0	0
PRJNA521949	SRR8567892	Apis mellifera	paired end	2019	2018	2018	Taiwan	Asia	10634944	624	0.01	0	0
PRJNA521949	SRR8567893	Apis mellifera	paired end	2019	2018	2018	Taiwan	Asia	12245720	832	0.01	0	0
PRJNA521949	SRR8567894	Apis mellifera	paired end	2019	2018	2018	Taiwan	Asia	14652912	684	0	0	0
PRJNA521949	SRR8567895	Apis mellifera	paired end	2019	2018	2018	Taiwan	Asia	11071136	825	0.01	0	0
PRJNA521949	SRR8567896	Apis mellifera	paired end	2019	2018	2018	Taiwan	Asia	10844048	497	0	0	0
PRJNA521949	SRR8567897	Apis mellifera	paired end	2019	2018	2018	Taiwan	Asia	23922560	749	0	0	0
PRJNA521949	SRR8567898	Apis mellifera	paired end	2019	2018	2018	Taiwan	Asia	12730504	512	0	0	0
PRJNA521949	SRR8567899	Apis mellifera	paired end	2019	2018	2018	Taiwan	Asia	19464522	834	0	0	0
PRJNA521949	SRR8567900	Apis mellifera	paired end	2019	2018	2018	Taiwan	Asia	22308188	3708	0.02	0	0
PRJNA521949	SRR8567901	Apis mellifera	paired end	2019	2018	2018	Taiwan	Asia	44538322	87610	0.2	0	0
PRJNA521949	SRR8567902	Apis mellifera	paired end	2019	2018	2018	Taiwan	Asia	18831416	1025	0.01	0	0
PRJNA521949	SRR8567903	Apis mellifera	paired end	2019	2018	2018	Taiwan	Asia	20523770	896	0	0	0
PRJNA521949	SRR8567904	Apis mellifera	paired end	2019	2018	2018	Taiwan	Asia	7326144	82	0	0	0
PRJNA521949	SRR8567905	Apis mellifera	paired end	2019	2018	2018	Taiwan	Asia	36517912	444	0	0	0
PRJNA521949	SRR8567906	Apis mellifera	paired end	2019	2018	2018	Taiwan	Asia	88924928	3334	0	0	0
PRJNA521949	SRR8567907	Apis mellifera	paired end	2019	2018	2018	Taiwan	Asia	86740316	14282575	16.47	0	0
PRJNA521949	SRR8567908	Apis mellifera	paired end	2019	2018	2018	Taiwan	Asia	35792442	248	0	0	0
PRJNA521949	SRR8567909	Apis mellifera	paired end	2019	2018	2018	Taiwan	Asia	12287256	93	0	0	0
PRJNA521949	SRR8567910	Apis mellifera	paired end	2019	2018	2018	Taiwan	Asia	38990652	272	0	0	0
PRJNA521949	SRR8567911	Apis mellifera	paired end	2019	2018	2018	Taiwan	Asia	31308148	274	0	0	0
PRJNA521949	SRR8567912	Apis mellifera	paired end	2019	2018	2018	Taiwan	Asia	33471874	300	0	0	0
PRJNA521949	SRR8567913	Apis mellifera	paired end	2019	2018	2018	Taiwan	Asia	19560138	3355	0.02	0	0
PRJNA521949	SRR8567914	Apis mellifera	paired end	2019	2018	2018	Taiwan	Asia	31208684	248	0	0	0
PRJNA521949	SRR8567915	Apis mellifera	paired end	2019	2018	2018	Taiwan	Asia	60377388	7784	0.01	0	0
PRJNA521949	SRR8567916	Apis mellifera	paired end	2019	2018	2018	Taiwan	Asia	20907978	4639	0.02	0	0
PRJNA521949	SRR8567917	Apis mellifera	paired end	2019	2018	2018	Taiwan	Asia	38006542	276	0	0	0
PRJNA521949	SRR8567918	Apis mellifera	paired end	2019	2018	2018	Taiwan	Asia	16589418	909	0.01	0	0
PRJNA521949	SRR8567919	Apis mellifera	paired end	2019	2018	2018	Taiwan	Asia	16801578	500	0	0	0
PRJNA521949	SRR8567920	Apis mellifera	paired end	2019	2018	2018	Taiwan	Asia	12461456	239609	1.92	0	0
PRJNA521949	SRR8567921	Apis mellifera	paired end	2019	2018	2018	Taiwan	Asia	13626226	430	0	0	0
PRJNA521949	SRR8567922	Apis mellifera	paired end	2019	2018	2018	Taiwan	Asia	113568310	7991	0.01	0	0
PRJNA521949	SRR8567923	Apis mellifera	paired end	2019	2018	2018	Taiwan	Asia	17550116	563	0	0	0
PRJNA521949	SRR8567924	Apis mellifera	paired end	2019	2018	2018	Taiwan	Asia	14844286	507	0	0	0
PRJNA521949	SRR8567925	Apis mellifera	paired end	2019	2018	2018	Taiwan	Asia	16421956	525	0	0	0
PRJNA521949	SRR8567926	Apis mellifera	paired end	2019	2018	2018	Taiwan	Asia	37752276	269	0	0	0
PRJNA521949	SRR8567927	Apis mellifera	paired end	2019	2018	2018	Taiwan	Asia	15017552	950	0.01	0	0
PRJNA521949	SRR8567928	Apis mellifera	paired end	2019	2018	2018	Taiwan	Asia	17513782	650	0	0	0
PRJNA521949	SRR8567929	Apis mellifera	paired end	2019	2018	2018	Taiwan	Asia	77277366	5392	0.01	0	0
PRJNA521949	SRR8567930	Apis mellifera	paired end	2019	2018	2018	Taiwan	Asia	19180708	862	0	0	0
PRJNA521949	SRR8567931	Apis mellifera	paired end	2019	2018	2018	Taiwan	Asia	19910742	868	0	0	0
PRJNA521949	SRR8567932	Apis mellifera	paired end	2019	2018	2018	Taiwan	Asia	34973778	427	0	0	0
PRJNA521949	SRR8567933	Apis mellifera	paired end	2019	2018	2018	Taiwan	Asia	123983530	5628	0	0	0
PRJNA521949	SRR8567934	Apis mellifera	paired end	2019	2018	2018	Taiwan	Asia	79269970	4182	0.01	0	0
PRJNA521949	SRR8567935	Apis mellifera	paired end	2019	2018	2018	Taiwan	Asia	19921192	35390	0.18	0	0
PRJNA521949	SRR8567936	Apis mellifera	paired end	2019	2018	2018	Taiwan	Asia	24937580	32542	0.13	0	0
PRJNA521949	SRR8567937	Apis mellifera	paired end	2019	2018	2018	Taiwan	Asia	20628728	2274	0.01	0	0
PRJNA521949	SRR8567938	Apis mellifera	paired end	2019	2018	2018	Taiwan	Asia	18675190	913	0	0	0
PRJNA521949	SRR8567939	Apis mellifera	paired end	2019	2018	2018	Taiwan	Asia	16818730	632	0	0	0
PRJNA521949	SRR8567940	Apis mellifera	paired end	2019	2018	2018	Taiwan	Asia	24549408	931	0	0	0
PRJNA521949	SRR8567941	Apis mellifera	paired end	2019	2018	2018	Taiwan	Asia	14856954	450	0	0	0
PRJNA521949	SRR8567942	Apis mellifera	paired end	2019	2018	2018	Taiwan	Asia	23484842	1542	0.01	0	0
PRJNA521949	SRR8567943	Apis mellifera	paired end	2019	2018	2018	Taiwan	Asia	35053370	274	0	0	0
PRJNA521949	SRR8567944	Apis mellifera	paired end	2019	2018	2018	Taiwan	Asia	43168074	7516	0.02	0	0
PRJNA521949	SRR8567945	Apis mellifera	paired end	2019	2018	2018	Taiwan	Asia	19768946	139	0	0	0
PRJNA521949	SRR8567946	Apis mellifera	paired end	2019	2018	2018	Taiwan	Asia	35949398	533	0	0	0
PRJNA521949	SRR8567947	Apis mellifera	paired end	2019	2018	2018	Taiwan	Asia	73755318	1635535	2.22	0	0
PRJNA521949	SRR8567948	Apis mellifera	paired end	2019	2018	2018	Taiwan	Asia	84164988	5751	0.01	0	0
PRJNA521949	SRR8567949	Apis mellifera	paired end	2019	2018	2018	Taiwan	Asia	12182660	1098	0.01	0	0
PRJNA521949	SRR8567950	Apis mellifera	paired end	2019	2018	2018	Taiwan	Asia	34350328	345	0	0	0
PRJNA521949	SRR8567951	Apis mellifera	paired end	2019	2018	2018	Taiwan	Asia	87897880	12080498	13.74	0	0
PRJNA527270	SRR8734356	Apis mellifera	paired end	2019	NA	2017	NA	NA	83830428	55	0	0	0
PRJNA527270	SRR8734357	Apis mellifera	paired end	2019	NA	2017	NA	NA	74849692	95	0	0	0
PRJNA527270	SRR8734358	Apis mellifera	paired end	2019	NA	2017	NA	NA	93250658	43	0	0	0
PRJNA527270	SRR8734359	Apis mellifera	paired end	2019	NA	2017	NA	NA	76210920	99907	0.13	0	0
PRJNA527270	SRR8734360	Apis mellifera	paired end	2019	NA	2017	NA	NA	107865312	545212	0.51	0	0
PRJNA527270	SRR8734361	Apis mellifera	paired end	2019	NA	2017	NA	NA	75040262	298	0	0	0
PRJNA495845	SRR8754026	Apis mellifera	paired end	2019	2012	2012	USA	North America	29053970	484	0	0	0
PRJNA495845	SRR8754027	Apis mellifera	paired end	2019	2012	2012	USA	North America	34951060	32238	0.09	0	0
PRJNA495845	SRR8754028	Apis mellifera	paired end	2019	2012	2012	USA	North America	29318906	1987	0.01	0	0

PRJNA495845	SRR8754029	Apis mellifera	paired end	2019	2012	2012	USA	North America	29212226	512990	1.76	0	0
PRJNA495845	SRR8754030	Apis mellifera	paired end	2019	2012	2012	USA	North America	32634896	3371617	10.33	1	0
PRJNA495845	SRR8754031	Apis mellifera	paired end	2019	2012	2012	USA	North America	36899356	10916	0.03	0	0
PRJNA495845	SRR8754032	Apis mellifera	paired end	2019	2012	2012	USA	North America	32962124	1218	0	0	0
PRJNA495845	SRR8754033	Apis mellifera	paired end	2019	2012	2012	USA	North America	35569972	1168	0	0	0
PRJNA495845	SRR8754034	Apis mellifera	paired end	2019	2012	2012	USA	North America	31979064	581	0	0	0
PRJNA495845	SRR8754035	Apis mellifera	paired end	2019	2012	2012	USA	North America	37409362	599	0	0	0
PRJNA495845	SRR8754036	Apis mellifera	paired end	2019	2012	2012	USA	North America	33156916	70959	0.21	4	0
PRJNA495845	SRR8754037	Apis mellifera	paired end	2019	2012	2012	USA	North America	30570846	451	0	2	0
PRJNA495845	SRR8754038	Apis mellifera	paired end	2019	2012	2012	USA	North America	34058376	78248	0.23	1	0
PRJNA495845	SRR8754039	Apis mellifera	paired end	2019	2012	2012	USA	North America	29089854	11874687	40.82	840	0
PRJNA495845	SRR8754040	Apis mellifera	paired end	2019	2012	2012	USA	North America	24981824	83745	0.34	0	0
PRJNA495845	SRR8754041	Apis mellifera	paired end	2019	2012	2012	USA	North America	21312870	749	0	0	0
PRJNA495845	SRR8754042	Apis mellifera	paired end	2019	2012	2012	USA	North America	42523320	694	0	0	0
PRJNA495845	SRR8754043	Apis mellifera	paired end	2019	2012	2012	USA	North America	26427434	3698	0.01	0	0
PRJNA495845	SRR8754044	Apis mellifera	paired end	2019	2012	2012	USA	North America	20601326	7119	0.03	0	0
PRJNA495845	SRR8754045	Apis mellifera	paired end	2019	2012	2012	USA	North America	29901958	1705	0.01	0	0
PRJNA495845	SRR8754046	Apis mellifera	paired end	2019	2012	2012	USA	North America	23700278	270	0	0	0
PRJNA495845	SRR8754047	Apis mellifera	paired end	2019	2012	2012	USA	North America	20982360	315	0	0	0
PRJNA495845	SRR8754048	Apis mellifera	paired end	2019	2012	2012	USA	North America	31842020	577	0	0	0
PRJNA495845	SRR8754049	Apis mellifera	paired end	2019	2012	2012	USA	North America	29237770	461	0	0	0
PRJNA495845	SRR8754050	Apis mellifera	paired end	2019	2012	2012	USA	North America	29362664	2699	0.01	0	0
PRJNA495845	SRR8754051	Apis mellifera	paired end	2019	2012	2012	USA	North America	28541336	909	0	0	0
PRJNA495845	SRR8754052	Apis mellifera	paired end	2019	2012	2012	USA	North America	27522294	678	0	0	0
PRJNA521949	SRR8769531	Apis mellifera	paired end	2019	2018	2018	Taiwan	Asia	189701076	1865209	0.98	0	0
PRJNA521949	SRR8769532	Apis mellifera	paired end	2019	2018	2018	Taiwan	Asia	166597826	6004	0	0	0
PRJNA521949	SRR8769533	Apis mellifera	paired end	2019	2018	2018	Taiwan	Asia	142158270	10755168	7.57	0	0
PRJNA521949	SRR8769534	Apis mellifera	paired end	2019	2018	2018	Taiwan	Asia	122640330	555548	0.45	0	0
PRJNA521949	SRR8769535	Apis mellifera	paired end	2019	2018	2018	Taiwan	Asia	182654420	152428	0.08	0	0
PRJNA521949	SRR8769536	Apis mellifera	paired end	2019	2018	2018	Taiwan	Asia	96874536	9097373	9.39	0	0
PRJNA521949	SRR8769537	Apis mellifera	paired end	2019	2018	2018	Taiwan	Asia	137253504	15627	0.01	0	0
PRJNA521949	SRR8769538	Apis mellifera	paired end	2019	2018	2018	Taiwan	Asia	137987144	17836	0.01	0	0
PRJNA521949	SRR8769539	Apis mellifera	paired end	2019	2018	2018	Taiwan	Asia	136378444	11532	0.01	0	0
PRJNA521949	SRR8769540	Apis mellifera	paired end	2019	2018	2018	Taiwan	Asia	150885934	219356	0.15	0	0
PRJNA521949	SRR8769541	Apis mellifera	paired end	2019	2018	2018	Taiwan	Asia	136816074	5084	0	0	0
PRJNA521949	SRR8769542	Apis mellifera	paired end	2019	2018	2018	Taiwan	Asia	186146500	8677	0	0	0
PRJNA521949	SRR8769543	Apis mellifera	paired end	2019	2018	2018	Taiwan	Asia	113920108	3259	0	0	0
PRJNA521949	SRR8769544	Apis mellifera	paired end	2019	2018	2018	Taiwan	Asia	127599446	4166	0	0	0
PRJNA521949	SRR8769545	Apis mellifera	paired end	2019	2018	2018	Taiwan	Asia	101274712	3545	0	0	0
PRJNA530116	SRR8823603	Apis mellifera	paired end	2019	2018	2018	NA	NA	22701638	0	0	0	0
PRJNA530116	SRR8823604	Apis mellifera	paired end	2019	2018	2018	NA	NA	18644624	0	0	0	0
PRJNA530116	SRR8823605	Apis mellifera	paired end	2019	2018	2018	NA	NA	20446520	0	0	0	0
PRJNA530116	SRR8823606	Apis mellifera	paired end	2019	2018	2018	NA	NA	21512882	4	0	0	0
PRJNA530116	SRR8823607	Apis mellifera	paired end	2019	2018	2018	NA	NA	19777484	0	0	0	0
PRJNA530116	SRR8823608	Apis mellifera	paired end	2019	2018	2018	NA	NA	22786038	0	0	0	0
PRJNA531527	SRR8867392	Apis mellifera	paired end	2019	2012	2012	USA	North America	27672054	10135743	36.63	90	0
PRJNA531527	SRR8867393	Apis mellifera	paired end	2019	2012	2012	USA	North America	35562276	7753299	21.8	21717	0.06
PRJNA531527	SRR8867394	Apis mellifera	paired end	2019	2012	2012	USA	North America	47278224	29768635	62.96	38154	0.08
PRJNA531527	SRR8867395	Apis mellifera	paired end	2019	2012	2012	USA	North America	24509166	36513	0.15	33238	0.14
PRJNA207482	SRR893021	Apis mellifera	paired end	2014	NA	2012	NA	NA	31171884	5940	0.02	0	0
PRJNA207482	SRR893022	Apis mellifera	paired end	2014	NA	2012	NA	NA	30206240	5759	0.02	0	0
PRJNA207482	SRR893023	Apis mellifera	paired end	2014	2011	2011	USA	North America	28501132	1147	0	0	0
PRJNA207482	SRR893024	Apis mellifera	paired end	2014	2011	2011	USA	North America	27684600	1089	0	0	0
PRJNA207482	SRR893025	Apis mellifera	paired end	2014	2011	2011	USA	North America	28126698	3072	0.01	0	0
PRJNA207482	SRR893026	Apis mellifera	paired end	2014	2011	2011	USA	North America	27576452	2915	0.01	0	0
PRJNA207482	SRR893027	Apis mellifera	paired end	2014	2011	2011	USA	North America	35688708	722	0	0	0
PRJNA207482	SRR893028	Apis mellifera	paired end	2014	2011	2011	USA	North America	34684012	679	0	0	0
PRJNA207482	SRR893029	Apis mellifera	paired end	2014	2011	2011	USA	North America	29592314	280	0	0	0
PRJNA207482	SRR893030	Apis mellifera	paired end	2014	2011	2011	USA	North America	28699416	358	0	0	0
PRJNA207482	SRR893031	Apis mellifera	paired end	2014	2011	2011	USA	North America	29026682	277	0	0	0
PRJNA207482	SRR893032	Apis mellifera	paired end	2014	2011	2011	USA	North America	28217372	256	0	0	0
PRJNA207482	SRR893033	Apis mellifera	paired end	2014	2011	2011	USA	North America	22991690	4003	0.02	0	0
PRJNA207482	SRR893034	Apis mellifera	paired end	2014	2011	2011	USA	North America	22326184	3784	0.02	0	0
PRJNA207482	SRR893035	Apis mellifera	paired end	2014	2011	2011	USA	North America	37104640	10387	0.03	0	0
PRJNA207482	SRR893036	Apis mellifera	paired end	2014	2011	2011	USA	North America	36086676	9792	0.03	0	0
PRJNA207482	SRR893037	Apis mellifera	paired end	2014	2011	2011	USA	North America	29784172	9517	0.03	0	0
PRJNA207482	SRR893038	Apis mellifera	paired end	2014	2011	2011	USA	North America	28785676	8956	0.03	0	0
PRJNA207482	SRR893039	Apis mellifera	paired end	2014	2011	2011	USA	North America	33704050	2311	0.01	0	0
PRJNA207482	SRR893040	Apis mellifera	paired end	2014	2011	2011	USA	North America	32712474	2255	0.01	0	0
PRJNA207482	SRR893041	Apis mellifera	paired end	2014	2011	2011	USA	North America	32029802	8801	0.03	0	0
PRJNA207482	SRR893042	Apis mellifera	paired end	2014	2011	2011	USA	North America	30946726	8159	0.03	0	0
PRJNA207482	SRR893043	Apis mellifera	paired end	2014	2011	2011	USA	North America	35414296	6125	0.02	0	0
PRJNA207482	SRR893044	Apis mellifera	paired end	2014	2011	2011	USA	North America	34352316	5545	0.02	0	0
PRJNA542364	SRR9041546	Apis mellifera	paired end	2020	2017	2017	USA	North America	109245238	23506	0.02	17366	0.02
PRJNA542364	SRR9041547	Apis mellifera	paired end	2020	2017	2017	USA	North America	97702914	689434	0.71	20988415	21.48
PRJNA542364	SRR9041548	Apis mellifera	paired end	2020	2017	2017	USA	North America	97240676	126282	0.13	2966203	3.05
PRJNA542364	SRR9041549	Apis mellifera	paired end	2020	2017	2017	USA	North America	87784410	205551	0.23	5394685	6.15
PRJNA542364	SRR9041550	Apis mellifera	paired end	2020	2017	2017	USA	North America	89019172	7	0	38	0
PRJNA542364	SRR9041551	Apis mellifera	paired end	2020	2017	2017	USA	North America	96046446	41	0	606	0
PRJNA542364	SRR9041552	Apis mellifera	paired end	2020	2017	2017	USA	North America	92924064	39	0	801	0
PRJNA542364	SRR9041553	Apis mellifera	paired end	2020	2017	2017	USA	North America	91687404	139	0	125	0
PRJNA542364	SRR9041554	Apis mellifera	paired end	2020	2017	2017	USA	North America	87030102	4208	0	442	0
PRJNA209400	SRR922438	Apis mellifera	paired end	2014	NA	2012	NA	NA	66868224	0	0	0	0
PRJNA209400	SRR922440	Apis mellifera	paired end	2014	NA	2012	NA	NA	98761682	0	0	0	0
PRJNA549281	SRR9312721	Apis mellifera	paired end	2021	NA	2019	NA	NA	56019142	0	0	0	0
PRJNA549281	SRR9312722	Apis mellifera	paired end	2021	NA	2019	NA	NA	75471780	0	0	0	0
PRJNA549281	SRR9312723	Apis mellifera	paired end	2021	NA	2019	NA	NA	82149974	0	0	0	0
PRJNA549281	SRR9312724	Apis mellifera	paired end	2021	NA	2019	NA	NA	68629974	0	0	0	0
PRJNA549281	SRR9312725	Apis mellifera	paired end	2021	NA	2019	NA	NA	64583486	0	0	0	0
PRJNA549281	SRR9312726	Apis mellifera	paired end	2021	NA	2019	NA	NA	72964220	0	0	0	0
PRJNA549281	SRR9312727	Apis mellifera	paired end	2021	NA	2019	NA	NA	77747452	0	0	0	0
PRJNA549281	SRR9312728	Apis mellifera	paired end	2021	NA	2019	NA	NA	65422338	0	0	0	0
PRJNA549281	SRR9312729	Apis mellifera	paired end	2021	NA	2019	NA	NA	71781546	0	0	0	0
PRJNA211831	SRR935172	Apis mellifera	paired end	2014	NA	2012	NA	NA	66042848	0	0	0	0
PRJNA211831	SRR935173	Apis mellifera	paired end	2014	NA	2012	NA	NA	61323428	0	0	0	0
PRJNA211831	SRR935174	Apis mellifera	paired end	2014	NA	2012	NA	NA	53731756	2	0	0	0
PRJNA211831	SRR935175	Apis mellifera	paired end	2014	NA	2012	NA	NA	71175712	0	0	0	0
PRJNA211831	SRR935176	Apis mellifera	paired end	2014	NA	2012	NA	NA	64037696	0	0	0	0
PRJNA211831	SRR935177	Apis mellifera	paired end	2014	NA	2012	NA	NA	58590986	0	0	0	0
PRJNA211831	SRR935178	Apis mellifera	paired end	2014	NA	2012	NA	NA	82638202	0	0	0	0
PRJNA211831	SRR935179	Apis mellifera	paired end	2014	NA	2012	NA	NA	74502098	0	0	0	0
PRJNA211831	SRR935180	Apis mellifera	paired end	2014	NA	2012	NA	NA	59593708	0	0	0	0
PRJEB9366	ERR883767	Bombus terrestris	single end	2015	NA	2013	NA	NA	19228973	0	0	0	0
PRJEB9366	ERR883768	Bombus terrestris	single end	2015	NA	2013	NA	NA	17832666	0	0	0	0
PRJEB9366	ERR883769	Bombus terrestris	single end	2015	NA	2013	NA	NA	20337107	0	0	0	0
PRJEB9366	ERR883770	Bombus terrestris	single end	2015	NA	2013	NA	NA	18829975	0	0	0	0

PRJEB9366	ERR883771	Bombus terrestris	single end	2015	NA	2013	NA	NA	14589067	0	0	0	0
PRJEB9366	ERR883772	Bombus terrestris	single end	2015	NA	2013	NA	NA	14720280	0	0	0	0
PRJEB9366	ERR883773	Bombus terrestris	single end	2015	NA	2013	NA	NA	16266154	0	0	0	0
PRJEB9366	ERR883774	Bombus terrestris	single end	2015	NA	2013	NA	NA	14603360	0	0	0	0
PRJEB9366	ERR883775	Bombus terrestris	single end	2015	NA	2013	NA	NA	16310880	0	0	0	0
PRJEB9366	ERR883776	Bombus terrestris	single end	2015	NA	2013	NA	NA	19235640	0	0	0	0
PRJEB9366	ERR883777	Bombus terrestris	single end	2015	NA	2013	NA	NA	16800890	0	0	0	0
PRJEB9366	ERR883778	Bombus terrestris	single end	2015	NA	2013	NA	NA	15488784	0	0	0	0
PRJEB9366	ERR883779	Bombus terrestris	single end	2015	NA	2013	NA	NA	14338587	0	0	0	0
PRJEB9366	ERR883780	Bombus terrestris	single end	2015	NA	2013	NA	NA	17780019	0	0	0	0
PRJEB9366	ERR883781	Bombus terrestris	single end	2015	NA	2013	NA	NA	18791069	0	0	0	0
PRJEB9366	ERR883782	Bombus terrestris	single end	2015	NA	2013	NA	NA	13855780	0	0	0	0
PRJEB9366	ERR883783	Bombus terrestris	single end	2015	NA	2013	NA	NA	20696845	0	0	0	0
PRJEB9366	ERR883784	Bombus terrestris	single end	2015	NA	2013	NA	NA	17339549	0	0	0	0
PRJEB9366	ERR883785	Bombus terrestris	single end	2015	NA	2013	NA	NA	16057607	0	0	0	0
PRJEB9366	ERR883786	Bombus terrestris	single end	2015	NA	2013	NA	NA	17407931	0	0	0	0
PRJEB9366	ERR883787	Bombus terrestris	single end	2015	NA	2013	NA	NA	17962612	0	0	0	0
PRJEB9366	ERR883788	Bombus terrestris	single end	2015	NA	2013	NA	NA	16388426	0	0	0	0
PRJEB9366	ERR883789	Bombus terrestris	single end	2015	NA	2013	NA	NA	17050136	0	0	0	0
PRJEB9366	ERR883790	Bombus terrestris	single end	2015	NA	2013	NA	NA	17154058	0	0	0	0
PRJEB9366	ERR883791	Bombus terrestris	single end	2015	NA	2013	NA	NA	16956446	0	0	0	0
PRJEB9366	ERR883792	Bombus terrestris	single end	2015	NA	2013	NA	NA	23722321	0	0	0	0
PRJEB9366	ERR883793	Bombus terrestris	single end	2015	NA	2013	NA	NA	19589338	0	0	0	0
PRJEB9937	ERR968678	Bombus terrestris	single end	2015	NA	2013	NA	NA	16499033	0	0	0	0
PRJEB9937	ERR968679	Bombus terrestris	single end	2015	NA	2013	NA	NA	39907164	0	0	0	0
PRJNA753273	SRR15402281	Bombus terrestris	single end	2021	2019	2019	Turkey	Asia	50021104	2	0	0	0
PRJNA753273	SRR15402282	Bombus terrestris	single end	2021	2019	2019	Turkey	Asia	49731094	0	0	0	0
PRJNA753273	SRR15402283	Bombus terrestris	single end	2021	2019	2019	Turkey	Asia	49823898	0	0	0	0
PRJNA753273	SRR15402284	Bombus terrestris	single end	2021	2019	2019	Turkey	Asia	49369054	0	0	1	0
PRJNA753273	SRR15402285	Bombus terrestris	single end	2021	2019	2019	Turkey	Asia	49157674	67	0	499	0
PRJNA753273	SRR15402286	Bombus terrestris	single end	2021	2019	2019	Turkey	Asia	57250656	117	0	300	0
PRJNA753273	SRR15402287	Bombus terrestris	single end	2021	2019	2019	Turkey	Asia	48803200	515	0	579	0
PRJNA753273	SRR15402288	Bombus terrestris	single end	2021	2019	2019	Turkey	Asia	41726990	2	0	2	0
PRJNA753273	SRR15402289	Bombus terrestris	single end	2021	2019	2019	Turkey	Asia	49544528	0	0	0	0
PRJNA753273	SRR15402290	Bombus terrestris	single end	2021	2019	2019	Turkey	Asia	48577950	0	0	0	0
PRJNA753273	SRR15402291	Bombus terrestris	single end	2021	2019	2019	Turkey	Asia	48968070	1	0	0	0
PRJNA753273	SRR15402292	Bombus terrestris	single end	2021	2019	2019	Turkey	Asia	48317646	0	0	3	0
PRJNA271157	SRR1734818	Bombus terrestris	single end	2015	NA	2013	NA	NA	5669480	0	0	1	0
PRJNA271157	SRR1734819	Bombus terrestris	single end	2015	NA	2013	NA	NA	7029923	0	0	1	0
PRJNA271157	SRR1734820	Bombus terrestris	single end	2015	NA	2013	NA	NA	2996910	0	0	0	0
PRJNA271157	SRR1734821	Bombus terrestris	single end	2015	NA	2013	NA	NA	3950811	0	0	0	0
PRJNA271157	SRR1734822	Bombus terrestris	single end	2015	NA	2013	NA	NA	4101572	0	0	0	0
PRJNA271157	SRR1734823	Bombus terrestris	single end	2015	NA	2013	NA	NA	3934876	0	0	0	0
PRJNA271157	SRR1734824	Bombus terrestris	single end	2015	NA	2013	NA	NA	4659419	0	0	0	0
PRJNA271157	SRR1734825	Bombus terrestris	single end	2015	NA	2013	NA	NA	5506360	0	0	0	0
PRJNA295976	SRR2396633	Bombus terrestris	single end	2015	NA	2013	NA	NA	6325143	14	0	0	0
PRJNA295976	SRR2396634	Bombus terrestris	single end	2015	NA	2013	NA	NA	7638890	3	0	0	0
PRJNA295976	SRR2396635	Bombus terrestris	single end	2015	NA	2013	NA	NA	7854028	1	0	0	0
PRJNA295976	SRR2396636	Bombus terrestris	single end	2015	NA	2013	NA	NA	6855909	0	0	0	0
PRJNA295976	SRR2396637	Bombus terrestris	single end	2015	NA	2013	NA	NA	8969750	0	0	0	0
PRJNA295976	SRR2396638	Bombus terrestris	single end	2015	NA	2013	NA	NA	9081069	1	0	0	0
PRJNA295976	SRR2396639	Bombus terrestris	single end	2015	NA	2013	NA	NA	9116604	0	0	0	0
PRJNA295976	SRR2396640	Bombus terrestris	single end	2015	NA	2013	NA	NA	8378724	1	0	0	0
PRJNA295976	SRR2396641	Bombus terrestris	single end	2015	NA	2013	NA	NA	7204794	0	0	0	0
PRJNA295976	SRR2396642	Bombus terrestris	single end	2015	NA	2013	NA	NA	6492516	4	0	0	0
PRJNA295976	SRR2396643	Bombus terrestris	single end	2015	NA	2013	NA	NA	7611257	0	0	0	0
PRJNA295976	SRR2396644	Bombus terrestris	single end	2015	NA	2013	NA	NA	8445768	3	0	0	0
PRJNA295976	SRR2396645	Bombus terrestris	single end	2015	NA	2013	NA	NA	8545171	3	0	0	0
PRJNA295976	SRR2396646	Bombus terrestris	single end	2015	NA	2013	NA	NA	9036778	2	0	0	0
PRJNA295976	SRR2396647	Bombus terrestris	single end	2015	NA	2013	NA	NA	7146573	5	0	0	0
PRJNA295976	SRR2396648	Bombus terrestris	single end	2015	NA	2013	NA	NA	8081346	0	0	0	0
PRJNA295976	SRR2396649	Bombus terrestris	single end	2015	NA	2013	NA	NA	7957466	2	0	0	0
PRJNA295976	SRR2396650	Bombus terrestris	single end	2015	NA	2013	NA	NA	6644153	1	0	0	0
PRJNA295976	SRR2396651	Bombus terrestris	single end	2015	NA	2013	NA	NA	6821075	2	0	0	0
PRJNA295976	SRR2396652	Bombus terrestris	single end	2015	NA	2013	NA	NA	8054617	0	0	0	0
PRJNA295976	SRR2396653	Bombus terrestris	single end	2015	NA	2013	NA	NA	7541784	0	0	0	0
PRJNA295976	SRR2396654	Bombus terrestris	single end	2015	NA	2013	NA	NA	7222207	0	0	0	0
PRJNA295976	SRR2396655	Bombus terrestris	single end	2015	NA	2013	NA	NA	9257337	1	0	0	0
PRJNA295976	SRR2396656	Bombus terrestris	single end	2015	NA	2013	NA	NA	8366303	8	0	0	0
PRJNA295976	SRR2396657	Bombus terrestris	single end	2015	NA	2013	NA	NA	7621052	0	0	0	0
PRJNA318490	SRR3383868	Bombus terrestris	single end	2016	2015	2015	Belgium	Europe	1717200	578	0.03	577	0.03
PRJNA355856	SRR5068586	Bombus terrestris	single end	2020	NA	2018	NA	NA	53726533	0	0	0	0
PRJNA355856	SRR5068587	Bombus terrestris	single end	2020	NA	2018	NA	NA	44622577	0	0	0	0
PRJNA355856	SRR5068588	Bombus terrestris	single end	2020	NA	2018	NA	NA	35535745	0	0	0	0
PRJNA355856	SRR5068589	Bombus terrestris	single end	2020	NA	2018	NA	NA	39752246	0	0	0	0
PRJNA355856	SRR5068590	Bombus terrestris	single end	2020	NA	2018	NA	NA	54677881	0	0	0	0
PRJNA355856	SRR5068591	Bombus terrestris	single end	2020	NA	2018	NA	NA	43740987	0	0	0	0
PRJNA355856	SRR5068592	Bombus terrestris	single end	2020	NA	2018	NA	NA	54674616	0	0	0	0
PRJNA355856	SRR5068593	Bombus terrestris	single end	2020	NA	2018	NA	NA	43077403	0	0	0	0
PRJNA355856	SRR5068594	Bombus terrestris	single end	2020	NA	2018	NA	NA	22098444	0	0	0	0
PRJNA355856	SRR5068595	Bombus terrestris	single end	2020	NA	2018	NA	NA	67547913	0	0	0	0
PRJNA355856	SRR5068596	Bombus terrestris	single end	2020	NA	2018	NA	NA	47648503	0	0	0	0
PRJNA355856	SRR5068597	Bombus terrestris	single end	2020	NA	2018	NA	NA	47917967	0	0	0	0
PRJNA355856	SRR5068598	Bombus terrestris	single end	2020	NA	2018	NA	NA	47127609	0	0	0	0
PRJNA355856	SRR5068599	Bombus terrestris	single end	2020	NA	2018	NA	NA	48433636	0	0	0	0
PRJNA355856	SRR5068600	Bombus terrestris	single end	2020	NA	2018	NA	NA	42868233	0	0	0	0
PRJNA355856	SRR5068601	Bombus terrestris	single end	2020	NA	2018	NA	NA	27111079	1	0	0	0
PRJNA355856	SRR5068602	Bombus terrestris	single end	2020	NA	2018	NA	NA	46612893	0	0	0	0
PRJNA355856	SRR5068603	Bombus terrestris	single end	2020	NA	2018	NA	NA	37547227	0	0	0	0
PRJNA355856	SRR5068604	Bombus terrestris	single end	2020	NA	2018	NA	NA	53461561	0	0	0	0
PRJNA355856	SRR5068605	Bombus terrestris	single end	2020	NA	2018	NA	NA	35120784	0	0	0	0
PRJNA508397	SRR8288022	Bombus terrestris	single end	2019	2016	2016	NA	NA	5637792	0	0	0	0
PRJNA508397	SRR8288023	Bombus terrestris	single end	2019	2016	2016	NA	NA	11338978	0	0	0	0
PRJNA508397	SRR8288201	Bombus terrestris	single end	2019	2016	2016	NA	NA	2453469	0	0	0	0
PRJNA508397	SRR8288202	Bombus terrestris	single end	2019	2016	2016	NA	NA	15985465	0	0	0	0
PRJNA508397	SRR8288214	Bombus terrestris	single end	2019	2016	2016	NA	NA	19279113	0	0	0	0
PRJNA508397	SRR8288215	Bombus terrestris	single end	2019	2016	2016	NA	NA	2906939	0	0	0	0
PRJNA508397	SRR8288242	Bombus terrestris	single end	2019	2016	2016	NA	NA	12282977	0	0	0	0
PRJNA508397	SRR8288243	Bombus terrestris	single end	2019	2016	2016	NA	NA	6958906	0	0	0	0
PRJNA508397	SRR8288337	Bombus terrestris	single end	2019	2016	2016	NA	NA	16645209	0	0	0	0
PRJNA508397	SRR8288338	Bombus terrestris	single end	2019	2016	2016	NA	NA	3942153	0	0	0	0
PRJNA508397	SRR8288339	Bombus terrestris	single end	2019	2016	2016	NA	NA	4000635	0	0	0	0
PRJNA508397	SRR8288340	Bombus terrestris	single end	2019	2016	2016	NA	NA	13975211	0	0	0	0
PRJNA508397	SRR8289668	Bombus terrestris	single end	2019	2016	2016	NA	NA	21481204	0	0	0	0
PRJNA508397	SRR8289669	Bombus terrestris	single end	2019	2016	2016	NA	NA	2404969	0	0	0	0
PRJNA508397	SRR8289689	Bombus terrestris	single end	2019	2016	2016	NA	NA	8706081	0	0	0	0

PRJNA508397	SRR8289690	Bombus terrestris	single end	2019	2016	2016	NA	NA	11718157	0	0	0	0
PRJNA508397	SRR8289691	Bombus terrestris	single end	2019	2016	2016	NA	NA	16709755	0	0	0	0
PRJNA508397	SRR8289692	Bombus terrestris	single end	2019	2016	2016	NA	NA	5926844	0	0	0	0
PRJNA508397	SRR8289714	Bombus terrestris	single end	2019	2016	2016	NA	NA	17784270	0	0	0	0
PRJNA508397	SRR8289715	Bombus terrestris	single end	2019	2016	2016	NA	NA	1715559	0	0	0	0
PRJNA508397	SRR8290146	Bombus terrestris	single end	2019	2016	2016	NA	NA	10023294	0	0	0	0
PRJNA508397	SRR8290147	Bombus terrestris	single end	2019	2016	2016	NA	NA	11616219	0	0	0	0
PRJNA508397	SRR8290169	Bombus terrestris	single end	2019	2016	2016	NA	NA	16457257	0	0	0	0
PRJNA508397	SRR8290170	Bombus terrestris	single end	2019	2016	2016	NA	NA	3928753	0	0	0	0
PRJNA508397	SRR8290191	Bombus terrestris	single end	2019	2016	2016	NA	NA	6897349	0	0	0	0
PRJNA508397	SRR8290192	Bombus terrestris	single end	2019	2016	2016	NA	NA	10385894	0	0	0	0
PRJNA508397	SRR8291682	Bombus terrestris	single end	2019	2016	2016	NA	NA	9000633	0	0	0	0
PRJNA508397	SRR8291683	Bombus terrestris	single end	2019	2016	2016	NA	NA	7172826	0	0	0	0
PRJNA508397	SRR8291751	Bombus terrestris	single end	2019	2016	2016	NA	NA	10661096	0	0	0	0
PRJNA508397	SRR8291752	Bombus terrestris	single end	2019	2016	2016	NA	NA	5878640	0	0	0	0
PRJNA508397	SRR8291869	Bombus terrestris	single end	2019	2016	2016	NA	NA	6224881	0	0	0	0
PRJNA508397	SRR8291870	Bombus terrestris	single end	2019	2016	2016	NA	NA	14370377	0	0	0	0
PRJNA508397	SRR8291892	Bombus terrestris	single end	2019	2016	2016	NA	NA	10494196	0	0	0	0
PRJNA508397	SRR8291893	Bombus terrestris	single end	2019	2016	2016	NA	NA	6791730	0	0	0	0
PRJNA508397	SRR8292121	Bombus terrestris	single end	2019	2016	2016	NA	NA	1941692	0	0	0	0
PRJNA508397	SRR8292122	Bombus terrestris	single end	2019	2016	2016	NA	NA	16364665	0	0	0	0
PRJNA508397	SRR8292125	Bombus terrestris	single end	2019	2016	2016	NA	NA	8391529	0	0	0	0
PRJNA508397	SRR8292126	Bombus terrestris	single end	2019	2016	2016	NA	NA	1456693	0	0	0	0
PRJNA508397	SRR8292128	Bombus terrestris	single end	2019	2016	2016	NA	NA	7206200	0	0	0	0
PRJNA508397	SRR8292129	Bombus terrestris	single end	2019	2016	2016	NA	NA	12183898	0	0	0	0
PRJNA508397	SRR8292135	Bombus terrestris	single end	2019	2016	2016	NA	NA	4448714	0	0	0	0
PRJNA508397	SRR8292136	Bombus terrestris	single end	2019	2016	2016	NA	NA	13659362	0	0	0	0
PRJNA508397	SRR8292162	Bombus terrestris	single end	2019	2016	2016	NA	NA	3422837	0	0	0	0
PRJNA508397	SRR8292163	Bombus terrestris	single end	2019	2016	2016	NA	NA	12367542	0	0	0	0
PRJNA508397	SRR8292230	Bombus terrestris	single end	2019	2016	2016	NA	NA	7088102	0	0	0	0
PRJNA508397	SRR8292231	Bombus terrestris	single end	2019	2016	2016	NA	NA	9414732	0	0	0	0
PRJNA508397	SRR8292237	Bombus terrestris	single end	2019	2016	2016	NA	NA	4253744	0	0	0	0
PRJNA508397	SRR8292238	Bombus terrestris	single end	2019	2016	2016	NA	NA	10371671	0	0	0	0
PRJNA533306	SRR8929852	Bombus terrestris	single end	2019	2016	2016	Belgium	Europe	2699327	0	0	0	0
PRJDB6538	DRR152981	Bombus terrestris	paired end	2018	NA	2016	NA	NA	22982038	0	0	0	0
PRJDB6538	DRR152982	Bombus terrestris	paired end	2018	NA	2016	NA	NA	14913126	0	0	0	0
PRJDB6538	DRR152983	Bombus terrestris	paired end	2018	NA	2016	NA	NA	13682372	0	0	0	0
PRJDB6538	DRR152984	Bombus terrestris	paired end	2018	NA	2016	NA	NA	34202834	0	0	0	0
PRJDB6538	DRR152985	Bombus terrestris	paired end	2018	NA	2016	NA	NA	32248374	0	0	0	0
PRJDB6538	DRR152986	Bombus terrestris	paired end	2018	NA	2016	NA	NA	30226176	0	0	0	0
PRJDB6538	DRR152987	Bombus terrestris	paired end	2018	NA	2016	NA	NA	22630006	0	0	0	0
PRJDB6538	DRR152988	Bombus terrestris	paired end	2018	NA	2016	NA	NA	22341088	0	0	0	0
PRJDB6538	DRR152989	Bombus terrestris	paired end	2018	NA	2016	NA	NA	21438236	0	0	0	0
PRJDB6538	DRR152990	Bombus terrestris	paired end	2018	NA	2016	NA	NA	38534344	0	0	0	0
PRJEB45168	ERR6363272	Bombus terrestris	paired end	2021	2020	2020	United Kingdom	Europe	50715972	0	0	0	0
PRJNA615177	SRR11445089	Bombus terrestris	paired end	2021	2004	2004	United Kingdom	Europe	112347920	0	0	0	0
PRJNA615177	SRR11445090	Bombus terrestris	paired end	2021	2004	2004	United Kingdom	Europe	100783008	0	0	0	0
PRJNA615177	SRR11445091	Bombus terrestris	paired end	2021	2004	2004	United Kingdom	Europe	99546426	0	0	0	0
PRJNA615177	SRR11448239	Bombus terrestris	paired end	2021	2004	2004	United Kingdom	Europe	103211992	0	0	0	0
PRJNA615177	SRR11448240	Bombus terrestris	paired end	2021	2004	2004	United Kingdom	Europe	97723116	0	0	0	0
PRJNA615177	SRR11448241	Bombus terrestris	paired end	2021	2004	2004	United Kingdom	Europe	97207226	0	0	0	0
PRJNA238307	SRR1169734	Bombus terrestris	paired end	2015	NA	2013	NA	NA	12312556	0	0	0	0
PRJNA238307	SRR1169735	Bombus terrestris	paired end	2015	NA	2013	NA	NA	13107886	0	0	0	0
PRJNA238307	SRR1169736	Bombus terrestris	paired end	2015	NA	2013	NA	NA	12632346	0	0	0	0
PRJNA238307	SRR1169737	Bombus terrestris	paired end	2015	NA	2013	NA	NA	12610988	0	0	0	0
PRJNA238307	SRR1169738	Bombus terrestris	paired end	2015	NA	2013	NA	NA	13277232	0	0	0	0
PRJNA238307	SRR1169739	Bombus terrestris	paired end	2015	NA	2013	NA	NA	12685250	0	0	0	0
PRJNA238307	SRR1169740	Bombus terrestris	paired end	2015	NA	2013	NA	NA	12642718	0	0	0	0
PRJNA238307	SRR1169741	Bombus terrestris	paired end	2015	NA	2013	NA	NA	13153178	0	0	0	0
PRJNA238307	SRR1169742	Bombus terrestris	paired end	2015	NA	2013	NA	NA	13229900	0	0	0	0
PRJNA238307	SRR1169743	Bombus terrestris	paired end	2015	NA	2013	NA	NA	12370652	0	0	0	0
PRJNA238307	SRR1169744	Bombus terrestris	paired end	2015	NA	2013	NA	NA	12839842	0	0	0	0
PRJNA238307	SRR1169745	Bombus terrestris	paired end	2015	NA	2013	NA	NA	12663482	0	0	0	0
PRJNA238307	SRR1169746	Bombus terrestris	paired end	2015	NA	2013	NA	NA	12990764	0	0	0	0
PRJNA238307	SRR1169747	Bombus terrestris	paired end	2015	NA	2013	NA	NA	12603724	0	0	0	0
PRJNA238307	SRR1169748	Bombus terrestris	paired end	2015	NA	2013	NA	NA	12750240	0	0	0	0
PRJNA238307	SRR1169749	Bombus terrestris	paired end	2015	NA	2013	NA	NA	12411698	0	0	0	0
PRJNA636805	SRR11943140	Bombus terrestris	paired end	2020	2018	2018	Germany	Europe	184151966	0	0	12	0
PRJNA637188	SRR12587876	Bombus terrestris	paired end	2021	NA	2019	NA	NA	117397290	0	0	0	0
PRJNA637188	SRR12587877	Bombus terrestris	paired end	2021	NA	2019	NA	NA	64538324	0	0	0	0
PRJNA704259	SRR13769018	Bombus terrestris	paired end	2021	2009	2009	United Kingdom	Europe	74846232	0	0	0	0
PRJNA704259	SRR13769019	Bombus terrestris	paired end	2021	2009	2009	United Kingdom	Europe	92831608	0	0	0	0
PRJNA779160	SRR16931588	Bombus terrestris	paired end	2021	2020	2020	China	Asia	46928336	36	0	0	0
PRJNA779160	SRR16931589	Bombus terrestris	paired end	2021	2020	2020	China	Asia	46400154	79	0	0	0
PRJNA779160	SRR16931590	Bombus terrestris	paired end	2021	2020	2020	China	Asia	50930234	80	0	0	0
PRJNA779160	SRR16931591	Bombus terrestris	paired end	2021	2020	2020	China	Asia	50112682	152	0	0	0
PRJNA779160	SRR16931592	Bombus terrestris	paired end	2021	2020	2020	China	Asia	55362860	638	0	0	0
PRJNA779160	SRR16931593	Bombus terrestris	paired end	2021	2020	2020	China	Asia	62248100	1386	0	0	0
PRJNA328517	SRR3879945	Bombus terrestris	paired end	2016	2012	2012	Switzerland	Europe	13523412	0	0	0	0
PRJNA328517	SRR3879947	Bombus terrestris	paired end	2016	2012	2012	Switzerland	Europe	13860004	0	0	0	0
PRJNA328517	SRR3879950	Bombus terrestris	paired end	2016	2012	2012	Switzerland	Europe	12829240	0	0	0	0
PRJNA328517	SRR3879952	Bombus terrestris	paired end	2016	2012	2012	Switzerland	Europe	13567618	0	0	0	0
PRJNA328517	SRR3879954	Bombus terrestris	paired end	2016	2012	2012	Switzerland	Europe	13536596	0	0	0	0
PRJNA328517	SRR3879956	Bombus terrestris	paired end	2016	2012	2012	Switzerland	Europe	13542410	0	0	0	0
PRJNA328517	SRR3879958	Bombus terrestris	paired end	2016	2012	2012	Switzerland	Europe	13256560	0	0	0	0
PRJNA328517	SRR3879960	Bombus terrestris	paired end	2016	2012	2012	Switzerland	Europe	13520526	0	0	0	0
PRJNA328517	SRR3879962	Bombus terrestris	paired end	2016	2012	2012	Switzerland	Europe	12941528	0	0	0	0
PRJNA358479	SRR5125102	Bombus terrestris	paired end	2017	NA	2015	NA	NA	47019680	0	0	0	0
PRJNA358479	SRR5125103	Bombus terrestris	paired end	2017	NA	2015	NA	NA	49501766	0	0	0	0
PRJNA358479	SRR5125104	Bombus terrestris	paired end	2017	NA	2015	NA	NA	54975072	0	0	0	0
PRJNA358479	SRR5125105	Bombus terrestris	paired end	2017	NA	2015	NA	NA	50117162	0	0	0	0
PRJNA358479	SRR5125106	Bombus terrestris	paired end	2017	NA	2015	NA	NA	53179288	0	0	0	0
PRJNA358479	SRR5125107	Bombus terrestris	paired end	2017	NA	2015	NA	NA	48482052	0	0	0	0
PRJNA358479	SRR5125108	Bombus terrestris	paired end	2017	NA	2015	NA	NA	56013116	0	0	0	0
PRJNA358479	SRR5125109	Bombus terrestris	paired end	2017	NA	2015	NA	NA	65410272	0	0	0	0
PRJNA358479	SRR5125110	Bombus terrestris	paired end	2017	NA	2015	NA	NA	61104222	0	0	0	0
PRJNA358479	SRR5125111	Bombus terrestris	paired end	2017	NA	2015	NA	NA	55263298	0	0	0	0
PRJNA358479	SRR5125112	Bombus terrestris	paired end	2017	NA	2015	NA	NA	59601460	0	0	0	0
PRJNA358479	SRR5125113	Bombus terrestris	paired end	2017	NA	2015	NA	NA	45703860	0	0	0	0
PRJNA358479	SRR5125114	Bombus terrestris	paired end	2017	NA	2015	NA	NA	48200466	0	0	0	0
PRJNA358479	SRR5125115	Bombus terrestris	paired end	2017	NA	2015	NA	NA	51781024	0	0	0	0
PRJNA358479	SRR5125116	Bombus terrestris	paired end	2017	NA	2015	NA	NA	49408288	0	0	0	0
PRJNA358479	SRR5125117	Bombus terrestris	paired end	2017	NA	2015	NA	NA	50832100	0	0	0	0
PRJNA358479	SRR5125118	Bombus terrestris	paired end	2017	NA	2015	NA	NA	50514114	0	0	0	0
PRJNA358479	SRR5125119	Bombus terrestris	paired end	2017	NA	2015	NA	NA	52102490	0	0	0	0
PRJNA358479	SRR5125120	Bombus terrestris	paired end	2017	NA	2015	NA	NA	51323512	0	0	0	0

PRJNA358479	SRR5125121	Bombus terrestris	paired end	2017	NA	2015	NA	NA	53081034	0	0	0	0
PRJNA358479	SRR5125122	Bombus terrestris	paired end	2017	NA	2015	NA	NA	46953490	0	0	0	0
PRJNA358479	SRR5125123	Bombus terrestris	paired end	2017	NA	2015	NA	NA	50370060	0	0	0	0
PRJNA358479	SRR5125124	Bombus terrestris	paired end	2017	NA	2015	NA	NA	59129662	0	0	0	0
PRJNA358479	SRR5125125	Bombus terrestris	paired end	2017	NA	2015	NA	NA	55749468	0	0	0	0
PRJNA358479	SRR5125126	Bombus terrestris	paired end	2017	NA	2015	NA	NA	57860990	0	0	0	0
PRJNA358479	SRR5125127	Bombus terrestris	paired end	2017	NA	2015	NA	NA	53607412	0	0	0	0
PRJNA358479	SRR5125128	Bombus terrestris	paired end	2017	NA	2015	NA	NA	49923908	0	0	0	0
PRJNA358479	SRR5125129	Bombus terrestris	paired end	2017	NA	2015	NA	NA	53394062	0	0	0	0
PRJNA358479	SRR5125130	Bombus terrestris	paired end	2017	NA	2015	NA	NA	57439134	0	0	0	0
PRJNA358479	SRR5125131	Bombus terrestris	paired end	2017	NA	2015	NA	NA	60548752	0	0	0	0
PRJNA358479	SRR5125132	Bombus terrestris	paired end	2017	NA	2015	NA	NA	51650938	0	0	0	0
PRJNA358479	SRR5125133	Bombus terrestris	paired end	2017	NA	2015	NA	NA	52118076	0	0	0	0
PRJNA358479	SRR5125134	Bombus terrestris	paired end	2017	NA	2015	NA	NA	47491950	0	0	0	0
PRJNA386859	SRR5614374	Bombus terrestris	paired end	2017	2016	2016	United Kingdom	Europe	21701860	0	0	0	0
PRJNA386859	SRR5614375	Bombus terrestris	paired end	2017	2016	2016	United Kingdom	Europe	17464500	0	0	0	0
PRJNA386859	SRR5614825	Bombus terrestris	paired end	2017	2016	2016	United Kingdom	Europe	39639922	0	0	0	0
PRJNA386859	SRR5614826	Bombus terrestris	paired end	2017	2016	2016	United Kingdom	Europe	66335852	0	0	0	0
PRJNA386859	SRR5614828	Bombus terrestris	paired end	2017	2016	2016	United Kingdom	Europe	57089018	0	0	0	0
PRJNA391408	SRR5739351	Bombus terrestris	paired end	2017	2012	2012	NA	NA	86381322	0	0	0	0
PRJNA411946	SRR6148368	Bombus terrestris	paired end	2018	2015	2015	Belgium	Europe	30991226	2	0	2	0
PRJNA411946	SRR6148370	Bombus terrestris	paired end	2018	2015	2015	Belgium	Europe	30343908	0	0	50	0
PRJNA411946	SRR6148374	Bombus terrestris	paired end	2018	2015	2015	Belgium	Europe	31094888	0	0	0	0
PRJNA497863	SRR8085426	Bombus terrestris	paired end	2019	2012	2012	Israel	Asia	69708528	630	0	108	0
PRJNA497863	SRR8085427	Bombus terrestris	paired end	2019	2012	2012	Israel	Asia	62695770	249	0	33	0
PRJNA497863	SRR8085428	Bombus terrestris	paired end	2019	2012	2012	Israel	Asia	64750316	152	0	28	0
PRJNA497863	SRR8085429	Bombus terrestris	paired end	2019	2012	2012	Israel	Asia	88891280	258	0	34	0
PRJNA497863	SRR8085430	Bombus terrestris	paired end	2019	2012	2012	Israel	Asia	71710530	104	0	15	0
PRJNA497863	SRR8085431	Bombus terrestris	paired end	2019	2012	2012	Israel	Asia	56814368	0	0	1	0
PRJNA497863	SRR8085432	Bombus terrestris	paired end	2019	2012	2012	Israel	Asia	68615816	4	0	4	0
PRJNA497863	SRR8085433	Bombus terrestris	paired end	2019	2012	2012	Israel	Asia	72861098	154	0	12	0
PRJNA497863	SRR8085434	Bombus terrestris	paired end	2019	2012	2012	Israel	Asia	64089978	8	0	2	0
PRJNA497863	SRR8085435	Bombus terrestris	paired end	2019	2012	2012	Israel	Asia	65988758	140	0	24	0
PRJNA497863	SRR8085436	Bombus terrestris	paired end	2019	2012	2012	Israel	Asia	69733244	40	0	9	0
PRJNA497863	SRR8085437	Bombus terrestris	paired end	2019	2012	2012	Israel	Asia	66538798	10	0	0	0
PRJNA497863	SRR8085438	Bombus terrestris	paired end	2019	2012	2012	Israel	Asia	69394740	94	0	13	0
PRJNA497863	SRR8085439	Bombus terrestris	paired end	2019	2012	2012	Israel	Asia	70771096	144	0	38	0
PRJNA497863	SRR8085440	Bombus terrestris	paired end	2019	2012	2012	Israel	Asia	76684616	1333	0	229	0
PRJNA497863	SRR8085441	Bombus terrestris	paired end	2019	2012	2012	Israel	Asia	64789228	206	0	57	0
PRJNA497863	SRR8085442	Bombus terrestris	paired end	2019	2012	2012	Israel	Asia	68917264	213	0	26	0
PRJNA497863	SRR8085443	Bombus terrestris	paired end	2019	2012	2012	Israel	Asia	82007530	420	0	54	0
PRJNA497863	SRR8085444	Bombus terrestris	paired end	2019	2012	2012	Israel	Asia	65172014	3	0	0	0
PRJNA497863	SRR8085445	Bombus terrestris	paired end	2019	2012	2012	Israel	Asia	70713174	44	0	9	0
PRJNA497863	SRR8085446	Bombus terrestris	paired end	2019	2012	2012	Israel	Asia	70305610	52	0	10	0
PRJNA497863	SRR8085447	Bombus terrestris	paired end	2019	2012	2012	Israel	Asia	62854238	821	0	97	0
PRJNA497863	SRR8085448	Bombus terrestris	paired end	2019	2012	2012	Israel	Asia	74894848	665	0	110	0
PRJNA497863	SRR8085449	Bombus terrestris	paired end	2019	2012	2012	Israel	Asia	75672738	103	0	14	0
PRJNA497863	SRR8085450	Bombus terrestris	paired end	2019	2012	2012	Israel	Asia	70843306	231	0	48	0
PRJNA497863	SRR8085451	Bombus terrestris	paired end	2019	2012	2012	Israel	Asia	67983610	428	0	71	0
PRJNA497863	SRR8085452	Bombus terrestris	paired end	2019	2012	2012	Israel	Asia	72462156	80	0	31	0
PRJNA497863	SRR8085453	Bombus terrestris	paired end	2019	2012	2012	Israel	Asia	68567196	9407	0.01	2127	0
PRJNA497863	SRR8085454	Bombus terrestris	paired end	2019	2012	2012	Israel	Asia	65870484	1493	0	352	0
PRJNA497863	SRR8085455	Bombus terrestris	paired end	2019	2012	2012	Israel	Asia	62564618	4352	0.01	891	0
PRJNA497863	SRR8085459	Bombus terrestris	paired end	2019	2012	2012	Israel	Asia	69164392	225	0	28	0
PRJNA497863	SRR8085460	Bombus terrestris	paired end	2019	2012	2012	Israel	Asia	69181190	1057	0	234	0
PRJNA497863	SRR8085461	Bombus terrestris	paired end	2019	2012	2012	Israel	Asia	60032064	466	0	89	0
PRJNA497863	SRR8085462	Bombus terrestris	paired end	2019	2012	2012	Israel	Asia	58444844	332	0	51	0
PRJNA497863	SRR8085463	Bombus terrestris	paired end	2019	2012	2012	Israel	Asia	63689854	324	0	56	0
PRJNA497863	SRR8085464	Bombus terrestris	paired end	2019	2012	2012	Israel	Asia	62224930	896	0	169	0
PRJNA497863	SRR8085465	Bombus terrestris	paired end	2019	2012	2012	Israel	Asia	63516008	474	0	123	0
PRJNA497863	SRR8085466	Bombus terrestris	paired end	2019	2012	2012	Israel	Asia	71413910	1317	0	202	0
PRJNA497863	SRR8085467	Bombus terrestris	paired end	2019	2012	2012	Israel	Asia	71435684	982	0	148	0
PRJNA497863	SRR8085468	Bombus terrestris	paired end	2019	2012	2012	Israel	Asia	85975636	2	0	0	0
PRJNA497863	SRR8085469	Bombus terrestris	paired end	2019	2012	2012	Israel	Asia	65536234	1637	0	286	0
PRJNA497863	SRR8085470	Bombus terrestris	paired end	2019	2012	2012	Israel	Asia	70589158	364	0	59	0
PRJNA497863	SRR8085471	Bombus terrestris	paired end	2019	2012	2012	Israel	Asia	61697640	1474	0	312	0
PRJNA497863	SRR8085473	Bombus terrestris	paired end	2019	2012	2012	Israel	Asia	49158730	1949	0	318	0
PRJNA497863	SRR8085474	Bombus terrestris	paired end	2019	2012	2012	Israel	Asia	67227952	2423	0	403	0
PRJNA497863	SRR8085475	Bombus terrestris	paired end	2019	2012	2012	Israel	Asia	66110664	1711	0	400	0
PRJNA497863	SRR8085476	Bombus terrestris	paired end	2019	2012	2012	Israel	Asia	71173444	2145	0	469	0
PRJNA497863	SRR8085477	Bombus terrestris	paired end	2019	2012	2012	Israel	Asia	62938736	275	0	45	0
PRJNA497863	SRR8085478	Bombus terrestris	paired end	2019	2012	2012	Israel	Asia	69597230	390	0	61	0
PRJNA497863	SRR8085479	Bombus terrestris	paired end	2019	2012	2012	Israel	Asia	70654764	1064	0	167	0
PRJNA497863	SRR8085480	Bombus terrestris	paired end	2019	2012	2012	Israel	Asia	66894892	536	0	55	0
PRJNA497863	SRR8085481	Bombus terrestris	paired end	2019	2012	2012	Israel	Asia	64673342	236	0	47	0
PRJNA497863	SRR8085482	Bombus terrestris	paired end	2019	2012	2012	Israel	Asia	68801416	276	0	47	0
PRJNA497863	SRR8085483	Bombus terrestris	paired end	2019	2012	2012	Israel	Asia	62078710	4098	0.01	943	0
PRJNA497863	SRR8085484	Bombus terrestris	paired end	2019	2012	2012	Israel	Asia	69737588	1436	0	237	0
PRJNA497863	SRR8085485	Bombus terrestris	paired end	2019	2012	2012	Israel	Asia	67190268	226	0	51	0
PRJNA497863	SRR8085486	Bombus terrestris	paired end	2019	2012	2012	Israel	Asia	70683146	219	0	34	0
PRJNA497863	SRR8330353	Bombus terrestris	paired end	2019	2012	2012	Israel	Asia	62855654	102	0	22	0
PRJNA533306	SRR8929838	Bombus terrestris	paired end	2019	2016	2016	Belgium	Europe	25702148	0	0	2	0
PRJNA533306	SRR8929839	Bombus terrestris	paired end	2019	2016	2016	Belgium	Europe	25681830	0	0	0	0
PRJNA533306	SRR8929840	Bombus terrestris	paired end	2019	2016	2016	Belgium	Europe	25642320	0	0	0	0
PRJNA533306	SRR8929841	Bombus terrestris	paired end	2019	2016	2016	Belgium	Europe	21529528	0	0	0	0
PRJNA533306	SRR8929842	Bombus terrestris	paired end	2019	2016	2016	Belgium	Europe	22985932	0	0	0	0
PRJNA533306	SRR8929843	Bombus terrestris	paired end	2019	2016	2016	Belgium	Europe	25626120	0	0	0	0
PRJNA533306	SRR8929848	Bombus terrestris	paired end	2019	2016	2016	Belgium	Europe	25163446	0	0	0	0
PRJNA533306	SRR8929849	Bombus terrestris	paired end	2019	2016	2016	Belgium	Europe	21613036	0	0	0	0
PRJNA533306	SRR8929852	Bombus terrestris	paired end	2019	2016	2016	Belgium	Europe	20257476	0	0	0	0
PRJNA533306	SRR8929853	Bombus terrestris	paired end	2019	2016	2016	Belgium	Europe	23669718	0	0	0	0
PRJNA533306	SRR8929854	Bombus terrestris	paired end	2019	2016	2016	Belgium	Europe	23591134	0	0	0	0
PRJNA533306	SRR8929855	Bombus terrestris	paired end	2019	2016	2016	Belgium	Europe	21342128	0	0	0	0
PRJNA533306	SRR8929856	Bombus terrestris	paired end	2019	2016	2016	Belgium	Europe	21633444	0	0	0	0
PRJNA533306	SRR8929857	Bombus terrestris	paired end	2019	2016	2016	Belgium	Europe	23595028	0	0	0	0
PRJNA533306	SRR8929858	Bombus terrestris	paired end	2019	2016	2016	Belgium	Europe	21539078	0	0	0	0
PRJNA533306	SRR8929859	Bombus terrestris	paired end	2019	2016	2016	Belgium	Europe	21500360	0	0	0	0
PRJNA533306	SRR8929860	Bombus terrestris	paired end	2019	2016	2016	Belgium	Europe	25674368	0	0	0	0
PRJNA533306	SRR8929861	Bombus terrestris	paired end	2019	2016	2016	Belgium	Europe	23571936	0	0	0	0
PRJNA533942	SRR8935708	Bombus terrestris	paired end	2020	2011	2011	NA	NA	10526704	0	0	0	0
PRJNA533942	SRR8935709	Bombus terrestris	paired end	2020	2011	2011	NA	NA	12930962	0	0	0	0
PRJNA533942	SRR8935710	Bombus terrestris	paired end	2020	2011	2011	NA	NA	12785708	0	0	0	0
PRJNA533942	SRR8935719	Bombus terrestris	paired end	2020	2011	2011	NA	NA	11140584	0	0	0	0
PRJNA533942	SRR8935729	Bombus terrestris	paired end	2020	2011	2011	NA	NA	12807972	0	0	0	0
PRJNA533942	SRR8935730	Bombus terrestris	paired end	2020	2011	2011	NA	NA	10584632	0	0	0	0
PRJNA533942	SRR8935736	Bombus terrestris	paired end	2020	2012	2012	NA	NA	28644952	0	0	0	0

PRJNA533942	SRR8935776	Bombus terrestris	paired end	2020	2012	2012	NA	NA	27951178	0	0	0	0
PRJNA533942	SRR8935807	Bombus terrestris	paired end	2020	2012	2012	NA	NA	31174982	0	0	0	0
PRJNA533942	SRR8935808	Bombus terrestris	paired end	2020	2012	2012	NA	NA	34881406	0	0	0	0
PRJNA533942	SRR8935840	Bombus terrestris	paired end	2020	2012	2012	NA	NA	31826642	0	0	0	0
PRJNA533942	SRR8935842	Bombus terrestris	paired end	2020	2012	2012	NA	NA	23416436	0	0	0	0
PRJNA533942	SRR8935859	Bombus terrestris	paired end	2020	2012	2012	NA	NA	29622448	0	0	0	0
PRJNA533942	SRR8935860	Bombus terrestris	paired end	2020	2012	2012	NA	NA	22624544	0	0	0	0
PRJNA533942	SRR8936095	Bombus terrestris	paired end	2020	2012	2012	NA	NA	24036230	0	0	0	0
PRJNA533942	SRR8936109	Bombus terrestris	paired end	2020	2012	2012	NA	NA	21708414	0	0	0	0
PRJNA533942	SRR8936121	Bombus terrestris	paired end	2020	2012	2012	NA	NA	16071782	0	0	0	0
PRJNA533942	SRR8936123	Bombus terrestris	paired end	2020	2012	2012	NA	NA	26617866	0	0	0	0
PRJNA533942	SRR8936139	Bombus terrestris	paired end	2020	2012	2012	NA	NA	21274674	0	0	0	0
PRJNA533942	SRR8936181	Bombus terrestris	paired end	2020	2012	2012	NA	NA	32374524	0	0	0	0
PRJNA524132	SRR8946237	Bombus terrestris	paired end	2020	2016	2016	United Kingdom	Europe	21667094	0	0	0	0
PRJNA524132	SRR8946238	Bombus terrestris	paired end	2020	2016	2016	United Kingdom	Europe	21622814	0	0	0	0
PRJNA524132	SRR8946239	Bombus terrestris	paired end	2020	2016	2016	United Kingdom	Europe	21799674	0	0	0	0
PRJNA524132	SRR8946240	Bombus terrestris	paired end	2020	2016	2016	United Kingdom	Europe	21901988	0	0	0	0
PRJNA524132	SRR8946247	Bombus terrestris	paired end	2020	2016	2016	United Kingdom	Europe	21765318	0	0	0	0
PRJNA524132	SRR8946248	Bombus terrestris	paired end	2020	2016	2016	United Kingdom	Europe	21714428	0	0	0	0
PRJNA524132	SRR8946249	Bombus terrestris	paired end	2020	2016	2016	United Kingdom	Europe	21845528	0	0	0	0
PRJNA524132	SRR8946250	Bombus terrestris	paired end	2020	2016	2016	United Kingdom	Europe	21817204	0	0	0	0
PRJNA524132	SRR8946251	Bombus terrestris	paired end	2020	2016	2016	United Kingdom	Europe	22037050	0	0	0	0
PRJNA524132	SRR8946252	Bombus terrestris	paired end	2020	2016	2016	United Kingdom	Europe	21901898	0	0	0	0
PRJNA524132	SRR8946253	Bombus terrestris	paired end	2020	2016	2016	United Kingdom	Europe	21595178	0	0	0	0
PRJNA524132	SRR8946254	Bombus terrestris	paired end	2020	2016	2016	United Kingdom	Europe	22044110	0	0	0	0
PRJNA524132	SRR8946255	Bombus terrestris	paired end	2020	2016	2016	United Kingdom	Europe	21951458	0	0	0	0
PRJNA524132	SRR8946256	Bombus terrestris	paired end	2020	2016	2016	United Kingdom	Europe	22014462	0	0	0	0
PRJNA524132	SRR8946257	Bombus terrestris	paired end	2020	2016	2016	United Kingdom	Europe	21883800	0	0	0	0
PRJNA524132	SRR8946258	Bombus terrestris	paired end	2020	2016	2016	United Kingdom	Europe	21732358	0	0	0	0
PRJNA524132	SRR8946259	Bombus terrestris	paired end	2020	2016	2016	United Kingdom	Europe	21759216	0	0	0	0
PRJNA524132	SRR8946260	Bombus terrestris	paired end	2020	2016	2016	United Kingdom	Europe	21163154	0	0	0	0
PRJNA646192	SRR12215891	Varroa destructor	single end	2021	2018	2018	Germany	Europe	27866085	101034	0.36	24561096	88.14
PRJNA646192	SRR12215902	Varroa destructor	single end	2021	2018	2018	Germany	Europe	24770899	387556	1.56	19953203	80.55
PRJNA646192	SRR12215913	Varroa destructor	single end	2021	2018	2018	Germany	Europe	21918003	77487	0.35	15748802	71.85
PRJNA646192	SRR12215924	Varroa destructor	single end	2021	2018	2018	Germany	Europe	21899381	1114	0.01	17710084	80.87
PRJNA646192	SRR12215935	Varroa destructor	single end	2021	2018	2018	Germany	Europe	22260731	1556	0.01	270767	1.22
PRJNA646192	SRR12215936	Varroa destructor	single end	2021	2018	2018	Germany	Europe	27396424	32928	0.12	5081115	18.55
PRJNA357165	SRR5109825	Varroa destructor	single end	2017	2013	2013	South Africa	Africa	26376162	0	0	1	0
PRJNA357165	SRR5109827	Varroa destructor	single end	2017	2013	2013	South Africa	Africa	29102112	3	0	1	0
PRJNA172020	SRR533974	Varroa destructor	single end	2015	NA	2013	NA	NA	22920031	4123915	17.99	1489	0.01
PRJNA392105	SRR5760812	Varroa destructor	single end	2018	NA	2016	France	Europe	28709594	7925	0.03	12772419	44.49
PRJNA392105	SRR5760813	Varroa destructor	single end	2018	NA	2016	France	Europe	31329405	1816	0.01	890019	2.84
PRJNA392105	SRR5760814	Varroa destructor	single end	2018	NA	2016	France	Europe	26670037	1172445	4.4	3934703	14.75
PRJNA392105	SRR5760815	Varroa destructor	single end	2018	NA	2016	France	Europe	24130717	1024	0	3325120	13.78
PRJNA392105	SRR5760816	Varroa destructor	single end	2018	NA	2016	France	Europe	26531852	411	0	10654	0.04
PRJNA392105	SRR5760817	Varroa destructor	single end	2018	NA	2016	France	Europe	27083570	1416	0.01	24888	0.09
PRJNA392105	SRR5760818	Varroa destructor	single end	2018	NA	2016	France	Europe	27017330	1090	0	11625	0.04
PRJNA392105	SRR5760819	Varroa destructor	single end	2018	NA	2016	France	Europe	27902246	421	0	1327730	4.76
PRJNA392105	SRR5760820	Varroa destructor	single end	2018	NA	2016	France	Europe	25675991	4558	0.02	15961	0.06
PRJNA392105	SRR5760821	Varroa destructor	single end	2018	NA	2016	France	Europe	36551386	2572	0.01	15698	0.04
PRJNA392105	SRR5760822	Varroa destructor	single end	2018	NA	2016	France	Europe	41269677	181	0	3109	0.01
PRJNA392105	SRR5760823	Varroa destructor	single end	2018	NA	2016	France	Europe	38107409	6819	0.02	16235	0.04
PRJNA392105	SRR5760824	Varroa destructor	single end	2018	NA	2016	France	Europe	37024580	24	0	1748	0
PRJNA392105	SRR5760825	Varroa destructor	single end	2018	NA	2016	France	Europe	39456450	186	0	2201	0.01
PRJNA392105	SRR5760826	Varroa destructor	single end	2018	NA	2016	France	Europe	28754022	1587	0.01	3765	0.01
PRJNA392105	SRR5760827	Varroa destructor	single end	2018	NA	2016	France	Europe	33433690	34	0	1496	0
PRJNA392105	SRR5760828	Varroa destructor	single end	2018	NA	2016	France	Europe	41817870	128	0	2785	0.01
PRJNA392105	SRR5760829	Varroa destructor	single end	2018	NA	2016	France	Europe	17467280	96	0	1635	0.01
PRJNA392105	SRR5760830	Varroa destructor	single end	2018	NA	2016	France	Europe	22878039	530	0	3951289	17.27
PRJNA392105	SRR5760831	Varroa destructor	single end	2018	NA	2016	France	Europe	27023481	20	0	1286	0
PRJNA392105	SRR5760832	Varroa destructor	single end	2018	NA	2016	France	Europe	21327691	159	0	5782	0.03
PRJNA392105	SRR5760833	Varroa destructor	single end	2018	NA	2016	France	Europe	21528331	15	0	17160	0.08
PRJNA392105	SRR5760834	Varroa destructor	single end	2018	NA	2016	France	Europe	21519896	25124	0.12	54726	0.25
PRJNA392105	SRR5760835	Varroa destructor	single end	2018	NA	2016	France	Europe	21762618	1457	0.01	4080	0.02
PRJNA392105	SRR5760836	Varroa destructor	single end	2018	NA	2016	France	Europe	19848879	31	0	3818	0.02
PRJNA392105	SRR5760837	Varroa destructor	single end	2018	NA	2016	France	Europe	20814439	47	0	2773	0.01
PRJNA392105	SRR5760838	Varroa destructor	single end	2018	NA	2016	France	Europe	22233902	288	0	8717	0.04
PRJNA392105	SRR5760839	Varroa destructor	single end	2018	NA	2016	France	Europe	19908519	472	0	172642	0.87
PRJNA392105	SRR5760840	Varroa destructor	single end	2018	NA	2016	France	Europe	22131893	4467	0.02	212495	0.96
PRJNA392105	SRR5760841	Varroa destructor	single end	2018	NA	2016	France	Europe	37352785	6103	0.02	141836	0.38
PRJNA392105	SRR5760842	Varroa destructor	single end	2018	NA	2016	France	Europe	38386922	14155	0.04	19811530	51.61
PRJNA392105	SRR5760843	Varroa destructor	single end	2018	NA	2016	France	Europe	34156388	51891	0.15	9884607	28.94
PRJNA392105	SRR5760844	Varroa destructor	single end	2018	NA	2016	France	Europe	33659627	7491	0.02	10576076	31.42
PRJNA392105	SRR5760845	Varroa destructor	single end	2018	NA	2016	France	Europe	31188064	3846	0.01	10137599	32.5
PRJNA392105	SRR5760846	Varroa destructor	single end	2018	NA	2016	France	Europe	36849086	19795	0.05	14681437	39.84
PRJNA392105	SRR5760847	Varroa destructor	single end	2018	NA	2016	France	Europe	27248138	5892	0.02	11386068	41.79
PRJNA392105	SRR5760848	Varroa destructor	single end	2018	NA	2016	France	Europe	28625532	4830	0.02	7926929	27.69
PRJNA392105	SRR5760849	Varroa destructor	single end	2018	NA	2016	France	Europe	32563456	8639987	26.53	11238183	34.51
PRJNA392105	SRR5760850	Varroa destructor	single end	2018	NA	2016	France	Europe	29446920	5151	0.02	1596821	5.42
PRJNA392105	SRR5760851	Varroa destructor	single end	2018	NA	2016	France	Europe	25029153	2125	0.01	407680	1.63
PRJNA437729	SRR6824277	Varroa destructor	single end	2018	2014	2014	Syria	Asia	36692614	20164628	54.96	3069616	8.37
PRJNA628859	SRR11624168	Varroa destructor	paired end	2020	2019	2019	China	Asia	111930228	6402392	5.72	13082	0.01
PRJNA628859	SRR11624169	Varroa destructor	paired end	2020	2019	2019	China	Asia	84224988	1910192	2.27	1828	0
PRJNA628859	SRR11624170	Varroa destructor	paired end	2020	2019	2019	China	Asia	88932536	3606394	4.06	3717	0
PRJNA642791	SRR12108021	Varroa destructor	paired end	2021	NA	2019	Belgium	Europe	38869728	12928676	33.26	13532884	34.82
PRJNA642791	SRR12108022	Varroa destructor	paired end	2021	NA	2019	Belgium	Europe	37454684	3911	0.01	4607	0.01
PRJNA642791	SRR12108023	Varroa destructor	paired end	2021	NA	2019	Belgium	Europe	35294754	9300592	26.35	471544	1.34
PRJNA642791	SRR12108024	Varroa destructor	paired end	2021	NA	2019	Belgium	Europe	26886100	4649	0.02	4329	0.02
PRJNA642791	SRR12108025	Varroa destructor	paired end	2021	NA	2019	Belgium	Europe	40795332	1343760	3.29	4181200	10.25
PRJNA642791	SRR12108026	Varroa destructor	paired end	2021	NA	2019	Belgium	Europe	40206770	7798378	19.4	1383972	3.44
PRJNA642791	SRR12108027	Varroa destructor	paired end	2021	NA	2019	Belgium	Europe	34998098	107099	0.31	2367114	6.76
PRJNA642791	SRR12108028	Varroa destructor	paired end	2021	NA	2019	Belgium	Europe	36686522	33369	0.09	43717	0.12
PRJNA642791	SRR12108029	Varroa destructor	paired end	2021	NA	2019	Belgium	Europe	43452536	13844060	31.86	1731218	3.98
PRJNA642791	SRR12108030	Varroa destructor	paired end	2021	NA	2019	Belgium	Europe	33431462	1239082	3.71	7155720	21.4
PRJNA642791	SRR12108031	Varroa destructor	paired end	2021	NA	2019	Belgium	Europe	31382966	239098	0.76	80744	0.26
PRJNA642791	SRR12108032	Varroa destructor	paired end	2021	NA	2019	Belgium	Europe	33955598	7230840	21.29	9445880	27.82
PRJNA329427	SRR3927486	Varroa destructor	paired end	2016	2014	2014	Israel	Asia	61001078	27968824	45.85	27713000	45.43
PRJNA329427	SRR3927496	Varroa destructor	paired end	2016	2014	2014	Israel	Asia	160000000	42637184	26.65	71686200	44.8
PRJNA380433	SRR5377263	Varroa destructor	paired end	2017	2013	2013	USA	North America	75969228	49068800	64.59	29460	0.04
PRJNA380433	SRR5377264	Varroa destructor	paired end	2017	2013	2013	USA	North America	47905680	9156968	19.11	6909	0.01
PRJNA380433	SRR5377265	Varroa destructor	paired end	2017	2013	2013	USA	North America	91630440	20919134	22.83	13344	0.01
PRJNA380433	SRR5377266	Varroa destructor	paired end	2017	2013	2013	USA	North America	51110172	36141000	70.71	27665	0.05
PRJNA380433	SRR5377267	Varroa destructor	paired end	2017	2013	2013	USA	North America	59669402	41268588	69.16	33442	0.06
PRJNA380433	SRR5377268	Varroa destructor	paired end	2017	2013	2013	USA	North America	54361322	43289400	79.63	32596	0.06
PRJNA380433	SRR5377269	Varroa destructor	paired end	2017	2013	2013	USA	North America	100585714	84902148	84.41	54437	0.05
PRJNA380433	SRR5377270	Varroa destructor	paired end	2017	2013	2013	USA	North America	90907006	520656	0.57	368	0

PRJNA383492	SRR5457677	Varroa destructor	paired end	2018	2016	2016	Israel	Asia	145512510	64192930	44.12	51708130	35.54
PRJNA383492	SRR5457678	Varroa destructor	paired end	2018	2016	2016	Israel	Asia	131312486	55363600	42.16	45646800	34.76
PRJNA383492	SRR5457680	Varroa destructor	paired end	2018	2016	2016	Israel	Asia	147718332	80584000	54.55	45016158	30.47
PRJNA383492	SRR5457681	Varroa destructor	paired end	2018	2016	2016	Israel	Asia	134744390	61271600	45.47	31271600	23.21
PRJNA437729	SRR6823684	Varroa destructor	paired end	2018	2014	2014	Syria	Asia	73385228	40455294	55.13	5956320	8.12
PRJNA437731	SRR6823686	Varroa destructor	paired end	2018	2014	2014	Algeria	Africa	70972834	1491700	2.1	42441	0.06
PRJNA473410	SRR7252454	Varroa destructor	paired end	2018	2016	2016	Israel	Asia	39756204	9341734	23.5	7298152	18.36
PRJNA473410	SRR7252455	Varroa destructor	paired end	2018	2016	2016	Israel	Asia	36248728	9535444	26.31	9755266	26.91
PRJNA473410	SRR7252456	Varroa destructor	paired end	2018	2016	2016	Israel	Asia	41689426	13827532	33.17	12188626	29.24
PRJNA473410	SRR7252457	Varroa destructor	paired end	2018	2016	2016	Israel	Asia	42013158	19472500	46.35	11316460	26.94
PRJNA473410	SRR7252458	Varroa destructor	paired end	2018	2016	2016	Israel	Asia	38804090	13349320	34.4	7504114	19.34
PRJNA473410	SRR7252459	Varroa destructor	paired end	2018	2016	2016	Israel	Asia	40341546	9763260	24.2	10562400	26.18
PRJNA473410	SRR7252460	Varroa destructor	paired end	2018	2016	2016	Israel	Asia	42994404	15990860	37.19	7773900	18.08
PRJNA473410	SRR7252461	Varroa destructor	paired end	2018	2016	2016	Israel	Asia	42516272	17768780	41.79	10156098	23.89
PRJNA473410	SRR7252462	Varroa destructor	paired end	2018	2016	2016	Israel	Asia	46892994	23259776	49.6	12250656	26.12
PRJNA473410	SRR7252463	Varroa destructor	paired end	2018	2016	2016	Israel	Asia	43819920	20978800	47.88	11975640	27.33
PRJNA473410	SRR7252464	Varroa destructor	paired end	2018	2016	2016	Israel	Asia	43047370	10842980	25.19	5949568	13.82
PRJNA473410	SRR7252465	Varroa destructor	paired end	2018	2016	2016	Israel	Asia	38810416	8810196	22.7	7841536	20.2
PRJNA475855	SRR7339931	Varroa destructor	paired end	2018	2016	2016	Thailand	Asia	255750226	12951	0.01	4139	0
PRJNA486893	SRR8100122	Varroa destructor	paired end	2019	2017	2017	China	Asia	205516488	122068692	59.4	114731	0.06
PRJNA486893	SRR8100123	Varroa destructor	paired end	2019	2017	2017	China	Asia	51391404	35493640	69.07	46129	0.09
PRJNA486893	SRR8100124	Varroa destructor	paired end	2019	2017	2017	China	Asia	204318580	60770400	29.74	2306980	1.13
PRJNA531374	SRR8864012	Varroa destructor	paired end	2019	2014	2014	United Kingdom	Europe	321761388	282156	0.09	80278722	24.95
PRJNA531527	SRR8867385	Varroa destructor	paired end	2019	2012	2012	USA	North America	27157060	21083200	77.63	4240280	15.61

Table S2. First records of DWV-B from a country or region in the literature.

	DOI/www of reference	Detection	Sequencing	DWV-A prevalence	DWV-B prevalence	sample size/type
1	10.1099/vir.0.80470-0	PCR	Sanger of genome	n=1	n=1	pool of mites
2	10.1007/s00705-020-04863-5	qPCR	no	n=1	n=1	1 colony
3	10.3390/v9110314	qPCR	Sanger of PCR product	100%	57%	14 colonies
4	10.1016/j.virol.2011.05.009	PCR	Sanger of PCR product	100%	100%	3 colonies
5	10.1371/journal.pone.0016217	PCR	no	100%	67%	30 queens
6	10.1126/science.aac9976	PCR	Sanger of PCR product	67%	73%	15 bees
7	10.1126/science.aac9976	PCR	Sanger of PCR product	80%	40%	2 bees, 3 mites
8	10.1371/journal.pone.0206938	NGS	Ion Torrent	high	very few reads	7 NGS libraries
9	10.3389/fgene.2015.00100	NGS	Illumina	all 6 sites	all 6 sites	6 sites/NGS pools
10	10.1038/s41598-017-17802-3	qPCR	Illumina	100%	3%	75 colonies
11	http://www.bulletinofinsectology.org/pdfarticles/vol71-2018-273-282abdi.pdf	qPCR	Sanger of PCR product	27%	7%	56 colonies
12	10.1038/nature12977	PCR	Sanger of PCR product	3%	8%	490 workers
13	10.1515/jas-2015-0005	qPCR	no	90%	100%	20 colonies
14	10.1098/rspb.2016.2149	NGS	Illumina	n=0	n=1	n=1 colony
15	10.1038/ismej.2015.186	NGS	Illumina	all 4 libraries	all 4 libraries	4 NGS libraries
16	https://www.au-ibar.org/bulletin-of-animal-health-and-production-in-africa?showall=&start=1	NGS	454	45% (reads)	55% (reads)	16 NGS libraries
17	10.1016/j.jip.2017.04.002	MLPA	Sanger of PCR product	>29%	<29%	40 bees
18	10.1038/s41598-017-15358-w	qPCR	no	not screened	high	4 colonies (401 bee
19	10.1007/s13592-017-0530-6	PCR	no	38%	6%	16 sites
20	10.1038/srep10454	NGS	Illumina	present	present	8 NGS libraries
21	10.1016/j.jip.2020.107324	qPCR	no	100%	100%	2 colonies
22	10.1007/s13592-019-00660-5	qPCR	Sanger of genome	90%	90%	10 colonies
23	10.1099/jgv.0.001206	qPCR	Sanger of PCR product	100%	11%	27 colonies
24	10.1080/00218839.2019.1670993	qPCR	Sanger of PCR product	71%	3%	612 colonies
25	10.1038/s41598-019-48618-y	qPCR	Sanger of PCR product	46%	2%	117 colonies
26	10.1098/rsbl.2018.0001	qPCR	Sanger of PCR product	0%	5%	20 Eristalis arbusto
27	10.1080/00218839.2019.1670993	qPCR	Sanger of PCR product	91%	47%	45 apiaries
28	10.3390/v12111218	qPCR	Illumina	0%	100%	20 colonies
29	10.1016/j.mcp.2021.101731	PCR	Sanger of PCR product	all sites	all sites	14 sites
30	10.3390/insects12121051	qPCR	no	58%	26%	250 colonies
31	10.3390/app12042134	qPCR	no	51%	18%	171 colonies

NB Thaduri et al. (2018) and post 2010 records from Europe (highlighted in yellow) have not been added to Figure 2.

NNB First records in Bombus spp. and a hover fly sp. (highlighted in green) have not been added to Figure 2.

Host species	Year of collection	Region	Country	Publication	Journal	Source in original publication
Varroa destructor	2001	Europe	The Netherlands	Ongus et al., 2004	Journal of General Viology	text
Apis mellifera	2004	Africa	South Africa	de Souza et al., 2021	Archives of Virology	Table 1
Apis mellifera	2006	Europe	England	Kevill et al., 2017	Viruses	Fig. 6
Apis mellifera	2008	Asia	Israel	Zioni et al., 2011	Virology	text
Apis mellifera	2007-2009	Europe	France	Gauthier et al., 2011	PLoS ONE	Table 3
Apis mellifera	2009	Europe	Germany	Wilfert et al., 2016	Science	Table S1
Apis mellifera	2009	Europe	Romania	Wilfert et al., 2016	Science	Table S1
Apis mellifera	2009-2010	Europe	Sweden	Thaduri et al., 2018	PLoS ONE	text
Apis mellifera	2010	Asia	Turkey	Tozkar et al., 2015	Frontiers in Genetics	Table 4 (in Supplement)
Apis mellifera	2010	N. America	USA	Ryabov et al., 2017	Scientific Reports	Fig. 2
Apis mellifera	2011	Africa	Tunisia	Abdi et al., 2018	Bulletin of Insectology	Table S1
Bombus spp.	2011	Europe	UK	Furst et al., 2014	Nature	Inferred from Fig. 3, text and Fig. S1
Apis mellifera	2011-2012	Europe	Luxemburg	Clermont et al., 2015	Journal of Apicultural Sciience	based on 1st sample date
Apis mellifera	2012	Europe	Belgium	Benaets et al., 2017	Proceedings Royal Society London B	Suppl. Methods, text
Apis mellifera	2012-2013	Pacific	USA (Hawaii)	Mordecai et al., 2016	ISME Journal	Fig. 3, Table S1
Apis mellifera	2012-2013	Africa	Kenya	Onyango et al., 2016	Bulletin Animal Health Prodution Africa	Table 1
Apis mellifera	2014	Asia	Georgia	Radzevičiūtė et al., 2017	Journal of Invertebrate Pathology	Fig. 4, Appendix Table A2
Apis mellifera	2014	Europe	Austria	Tritschler et al., 2017	Scientific Reports	text, Fig. 3
Apis mellifera	2014 *	Asia	Yemen	Haddad et al., 2018	Apidologie	* date pers comm Horth
Apis mellifera	2014	Pacific	New Zealand	Mondet et al., 2015	Scientific Reports	text
Apis mellifera	2015 *	Asia	Philippines	de Guzman et al., 2020	Journal of Invertebrate Pathology	* date pers comm Simone-Finstrom
Apis mellifera	2015	Europe	Spain	Barroso-Arévalo et al., 2019	Apidologie	Suppl. Data 4 and 5
Apis mellifera	2015	S. America	Brazil	de Souza et al., 2019	Journal of General Virology	Fig. 3, text
Apis mellifera	2015	S. America	Chile	Riveros et al., 2020	Journal of Apicultural Research	text
Apis mellifera	2015	Asia	China	Diao et al., 2019	Scientific Reports	text
Eristalis arbustorum	2016	Europe	UK	Bailes et al., 2018	Biology Letters	Table 1, text
Apis mellifera	2016	S. America	Argentina	Brasesco et al., 2021	Bulletin of Entomnological Research	Table 1, text
Apis mellifera	2017	Africa	Ethiopia	Gebremedhn et al., 2020	Viruses	Fig. 3, Fig. 4
Apis mellifera	2017-2018	Africa	Egypt	Abd-El-Samie et al., 2021	Molecular and Cellular Probes	text
Apis mellifera	2019	Europe	Czech Republic	Mráz et al., 2021	Insects	estimate from Fig. 5
Apis mellifera	2020	Europe	Italy	Bordin et al., 2022	Applied Sciences	text

Table S3. DWV prevalence in honey bees at 7 sites in 2011 and 2017 on mainland UK

Site code	Site code in McMahon et al. 2016	N honey bees (2011)	N DWV-A(2011)	N DWV-B (2011)	N honey bees (2017)	N DWV-A (2017)	N DWV-B (2017)	pA(2011)	pA(2017)	pB(2011)	pB(2017)
AM	29	10	7	0	13	1	5	0.700	0.077	0.000	0.385
BM	4	10	0	0	22	2	15	0.000	0.091	0.000	0.682
CM	5	8	4	1	29	0	4	0.500	0.000	0.125	0.138
IM	11	2	0	0	8	1	3	0.000	0.125	0.000	0.375
NM (F)	16	8	1	0	7	1	0	0.125	0.143	0.000	0.000
O(M)	17	8	2	1	18	6	2	0.250	0.333	0.125	0.111
WM	25	9	3	9	9	0	2	0.333	0.000	1.000	0.222
Total		55	17	11	106	11	31	0.309	0.104	0.200	0.292

Table S4. Results of screening German colonies from 2013, 2019 and 2020, with primers used in RT-qPCR

DWV (1)	For	TTTGCAAGATGCTGTATGTGG	Gauthier et al., Apidologie 38 (2007) 426–435
	Rev	GTCGTGCAGCTCGATAGGAT	

DWV (2)	For	GGTCCGCGGCTAAGATTGTA	Blanchard et al., Journal of Virological Methods 141 (2007) 7–13
	Rev	CGGCTGTTTGATGGAAGAAGTT	

DWV (3)	For	CCTGCTAATCAACAAGGACCTGG	Genersch, The Veterinary Journal 169 (2005) 121-123
	Rev	CAGAACCAATGTCTAACGCTAACCC	

Evaluation:	++++	+++	++	+	(+)	-
Classic PCR (band on gel	very strong	strong	medium	weak	very weak	no

DWV-A	For	GCGGCTAAGATTGTAAATTG	Schurr et al., Journal of Virological Methods 270 (2019) 70–78
	Rev	GTGACTAGCATAACCATGATTA	
	Probe	CCTTGACCAGTAGACACAGCATC	
DVW-B	For	GGTCTGAAGCGAAAATAG	
	Rev	CTAGCATATCCATGATTATAAAC	
	Probe	CCTTGTCCAGTAGATACAGCATCACA	

Evaluation:	++++	+++	++	+	(+)	-
RT-qPCR	Cq < 20	20 ≤ Cq < 28	28 ≤ Cq < 32	32 ≤ Cq< 35	35 ≤ Cq < *	Cq ≥ *

(*DWV-A=36; *DWV-B=37)

ß-Actin	For	AGGAATGGAAGCTTGCGGTA	Chen et al., Applied and Environmental Microbiology 71(1) (2005) 436-441
	Rev	AATTTTCATGGTGGATGGTGC	
	Probe	ATGCCAACACTGTCCTTTCTGGAGGTA	

		Classic PCR	RT-qPCR analysed in 2021				
2013	federal state	DWV (1)	DWV-A	Cq	DWV-B	Cq	ß-Actin [Cq]
1	BW	++	(+)	35.99	++++	8.80	25.45
2	MV	+++	+++	21.58	++++	11.15	25.04
3	BY	+++	++++	17.54	++++	5.86	24.77
4	SN	++	+	34.00	++++	10.45	26.28
5	BY	+++	++++	18.17	++++	10.69	26.31
6	SN	++	++	30.17	++	30.34	26.19
7	SN	+++	++++	17.82	++++	15.28	26.31
8	BW	++	++	31.84	++++	11.77	26.50
9	BW	++	+	34.77	++++	12.33	25.99
10	NI	+++	++	29.32	++++	14.41	27.68
11	NI	+++	-	N/A	++++	12.26	26.40
12	HE	+++	+++	24.97	++++	14.44	26.64
13	NW	+++	+++	27.17	+++	27.81	24.81
14	BY	+++	++	30.33	++++	17.32	27.04
15	SH	+++	++	30.09	++++	19.51	28.27
16	SH	+++	++	28.83	+++	26.27	26.30
17	NI	-	-	N/A	++	29.18	24.76
18	NI	+	-	N/A	(+)	35.50	26.39
19	SA	+++	++++	15.43	++++	14.24	26.84
20	BW	++	++	29.51	+++	27.64	25.25
21	BW	++	++	30.53	++++	12.32	23.50
22	BY	++	++	30.11	++	29.47	23.65
23	NI	++	+	32.38	++	29.17	24.25
24	NI	++	+	32.06	+++	26.30	22.95
25	BW	++	+	34.53	+++	25.35	26.92
26	TH	+++	+	33.37	++++	16.99	24.58
27	ST	++	+	34.28	++	28.05	25.71
28	NI	-	(+)	35.32	++++	15.33	26.56
29	NI	++	-	N/A	++++	16.30	27.22
30	NI	++	-	N/A	++++	18.23	25.28
31	NI	++	+	33.30	++++	15.88	24.85
32	NI	+	-	N/A	+++	26.13	27.47
33	NI	+	(+)	35.66	++	30.10	25.58
34	BW	-	-	N/A	+++	26.84	28.13
35	BW	-	-	N/A	+++	25.22	26.26
36	BW	-	-	N/A	++++	18.55	26.15
37	SL	-	-	N/A	++	31.76	28.50

38	BW	-	-	N/A	+++	20.24	25.41
39	BW	-	-	N/A	+++	23.34	24.98
40	BW	+++	-	N/A	+++	26.24	24.76
41	BW	+	-	N/A	++	28.52	24.41
42	NI	-	-	N/A	+	32.23	26.39
43	SH	-	-	N/A	+++	26.20	25.35
44	BB	-	-	N/A	-	N/A	25.34
45	NW	++	-	N/A	+	34.95	22.98
46	HE	-	-	N/A	+	33.08	25.35
47	SH	++	-	N/A	++	30.03	25.80
48	BY	-	-	N/A	+	34.98	25.10
49	BY	+++	++++	19.57	(+)	36.64	23.07
50	BY	++	+	34.23	+	33.20	19.36
51	BY	++	+	32.42	+	32.17	21.56
52	NI	-	-	N/A	+++	26.50	25.28
53	RP	-	(+)	35.23	++	29.63	24.78
54	BB	-	-	N/A	(+)	35.90	22.38
55	BB	-	-	N/A	(+)	36.67	25.13
56	BW	-	-	N/A	++	31.20	25.17
57	SN	++	+	33.18	++	29.33	26.05
58	NW	+	-	N/A	+	33.57	27.22
59	BY	+	-	N/A	+	34.93	24.78
60	HE	+	-	N/A	+	33.84	25.71
61	SN	++	+	32.10	++	29.78	26.41
62	NW	++	-	N/A	+++	22.65	25.63
63	NI	+	-	N/A	++++	15.04	22.21
64	SN	+++	+++	23.11	++	28.98	23.27
65	SN	+++	+++	24.47	++	30.61	25.05
66	NI	++	-	N/A	++++	18.67	25.74
67	HE	+	++	31.59	++++	13.17	25.61
68	SN	++	++++	15.04	++++	13.17	25.87
69	SN	++	++++	11.57	++++	13.71	25.79
70	SN	++	++++	11.30	++++	12.03	22.32

		Classic PCR	RT-qPCR		Classic PCR	RT-qPCR		
**2019**	**federal state**	**DWV (2)**	**DWV-A**	**Cq**	**DWV (3)**	**DWV-B**	**Cq**	**ß-Actin [Cq]**
1	SN	-	-	N/A	++	++++	19.17	22.63
2	SN	-	-	N/A	+++	++++	9.47	23.11
3	ST	-	-	N/A	+++	++++	12.60	24.04
4	MV	++++	++++	18.70	+++	++++	11.40	24.01
5	BB	-	-	N/A	+++	++++	12.61	21.29
6	BY	++	+++	26.80	+++	++++	7.93	24.26
7	BY	-	-	N/A	+	+++	24.93	20.08
8	MV	-	-	N/A	(+)	+++	26.75	26.74
9	SH	-	-	N/A	+++	++++	12.76	26.76
10	SN	-	-	N/A	+++	++++	11.13	21.27
11	BY	-	-	N/A	+++	++++	7.26	21.04
12	MV	-	-	N/A	+++	++++	9.77	23.91
13	MV	-	-	N/A	+++	++++	10.20	23.76
14	MV	-	-	N/A	+++	++++	13.08	21.20
15	MV	-	-	N/A	+++	++++	9.40	23.32
16	NI	-	-	N/A	-	++	29.54	19.75
17	SH	-	-	N/A	+++	+++	24.47	23.97
18	BY	-	-	N/A	++++	++++	10.54	22.44
19	SN	-	-	N/A	++++	++++	12.63	23.43
20	SN	-	-	N/A	+++	++++	14.71	25.96
21	BY	-	-	N/A	++	++	31.44	26.07
22	HE	-	-	N/A	+++	+++	25.06	26.46
23	NW	-	-	N/A	+++	+++	27.31	25.24
24	SN	-	-	N/A	+++	+++	26.11	25.14
25	HH	-	-	N/A	(+)	++	31.40	23.00
26	BW	-	-	N/A	-	+	32.48	25.70
27	BY	-	-	N/A	++++	++++	19.31	24.63
28	NW	-	-	N/A	-	+	34.38	23.93
29	BW	-	-	N/A	-	++	29.96	25.57
30	BW	-	-	N/A	++++	++++	19.86	25.72
31	BY	-	-	N/A	(+)	++	31.33	22.18
32	BY	-	-	N/A	(+)	+++	26.65	23.93
33	TH	-	-	N/A	++++	++++	11.03	24.48
34	BB	-	-	N/A	++++	++++	16.27	24.50

35	HE	+	-	N/A	-	+	32.29	24.41
36	SN	-	-	N/A	(+)	++	31.33	24.97
37	BY	-	-	N/A	+++	++++	8.33	21.55
38	HE	-	-	N/A	(+)	+++	27.63	24.34
39	NW	-	-	N/A	+++	++	30.28	24.79
40	BW	-	-	N/A	-	++++	17.37	23.38
41	BW	-	-	N/A	-	(+)	35.41	25.42
42	BW	-	-	N/A	+++	++++	18.59	25.55
43	ST	-	-	N/A	++++	++++	13.52	23.74
44	BY	-	-	N/A	-	+	33.04	26.21
45	RP	-	-	N/A	(+)	++	28.19	22.72
46	BY	-	-	N/A	++++	+++	23.29	22.42
47	BY	-	-	N/A	+	+++	23.81	24.09
48	BW	-	-	N/A	+	+++	22.77	20.42
49	BY	-	-	N/A	+++	+++	23.45	20.54
50	MV	-	-	N/A	-	++	31.92	19.49
51	NW	-	-	N/A	++++	++++	6.97	20.18
52	BY	-	-	N/A	++	+++	24.21	22.08
53	BY	-	-	N/A	+++	+++	22.16	22.39
54	BW	-	-	N/A	+++	++++	19.30	21.21
55	BY	-	-	N/A	-	++	28.60	20.34
56	BY	-	-	N/A	++++	++++	11.11	21.21
57	TH	-	-	N/A	++++	++++	17.68	19.05
58	BY	-	-	N/A	++	+++	26.11	22.79
59	NW	-	-	N/A	+	++	28.36	22.78
60	BY	-	-	N/A	+	++	29.01	23.09
61	NI	-	-	N/A	-	(+)	35.57	23.85
62	BW	-	-	N/A	++++	++++	10.12	20.50
63	SN	-	-	N/A	++++	++++	10.80	25.82
64	SN	-	-	N/A	++++	++++	10.86	23.11
65	MV	-	-	N/A	++++	++++	10.89	24.23
66	BY	-	-	N/A	++++	++++	9.71	18.51
67	HE	-	-	N/A	+++	++++	18.23	19.00
68	SH	-	-	N/A	++	++++	16.18	21.80
69	BY	-	-	N/A	+++	++++	12.83	25.50
70	SN	-	-	N/A	++++	++++	13.89	21.77

		Classic PCR	RT-qPCR		Classic PCR	RT-qPCR		
**2020**	**federal state**	**DWV (2)**	**DWV-A**	**Cq**	**DWV (3)**	**DWV-B**	**Cq**	**ß-Actin [Cq]**
1	BY	-	-	N/A	++++	++++	10.30	24.14
2	NI	-	-	N/A	++++	++++	13.03	25.08
3	BY	-	-	N/A	++++	++++	11.35	23.77
4	NI	-	-	N/A	++	+++	20.94	21.24
5	BY	-	-	N/A	++++	++++	8.46	23.95
6	BY	++++	++++	16.14	+++	++++	11.23	19.53
7	NI	-	(+)	35.26	+++	++++	19.17	23.83
8	RP	NP	+	34.39	NP	++++	9.78	20.18
9	MV	NP	+	34.84	NP	+++	22.29	24.85
10	NI	NP	-	N/A	NP	++++	8.14	26.22
11	HE	NP	-	N/A	NP	++++	13.02	26.26
12	BY	NP	-	N/A	NP	++++	7.76	22.31
13	NW	NP	-	N/A	NP	++++	10.63	27.96
14	BW	NP	++	30.3	NP	++++	16.85	24.43
15	BW	NP	-	N/A	NP	++++	10.98	22.32
16	BB	NP	(+)	35.77	NP	+++	27.28	18.01
17	BW	NP	-	N/A	NP	+++	25.99	18.95
18	BW	NP	-	N/A	NP	++	30.89	20.09
19	BW	NP	+	32.63	NP	++++	13.80	22.44
20	SN	NP	-	N/A	NP	++	28.32	20.27
21	BY	NP	-	N/A	NP	+++	21.21	19.66
22	BW	NP	-	N/A	NP	++	31.83	19.26
23	NW	NP	-	N/A	NP	+++	24.60	20.30
24	BW	NP	-	N/A	NP	+	32.22	19.76
25	BY	NP	-	N/A	NP	++	31.28	19.36
26	BW	NP	-	N/A	NP	+++	20.26	21.35
27	BB	NP	-	N/A	NP	++	30.01	21.74
28	BY	NP	-	N/A	NP	++++	19.93	20.13
29	NW	NP	-	N/A	NP	+++	22.12	21.73
30	NW	NP	-	N/A	NP	++++	14.54	16.07
31	BY	NP	-	N/A	NP	++	29.18	22.19

32	BE	NP	-	N/A	NP	++	30.44	17.83
33	NI	NP	-	N/A	NP	+++	27.09	21.16
34	BW	NP	-	N/A	NP	+++	27.60	20.40
35	SN	NP	-	N/A	NP	++	30.78	21.31
36	TH	NP	-	N/A	NP	++	30.58	21.65
37	BY	NP	-	N/A	NP	(+)	35.24	19.83
38	BW	NP	-	N/A	NP	+	34.32	21.69
39	SN	NP	-	N/A	NP	++	31.80	21.40
40	NI	NP	-	N/A	NP	++	29.50	20.01
41	BY	NP	-	N/A	NP	+	32.50	19.40
42	NW	NP	-	N/A	NP	++	29.06	18.03
43	BW	NP	-	N/A	NP	++	28.15	23.56
44	BW	NP	-	N/A	NP	++	30.14	21.80
45	NI	NP	-	N/A	NP	++++	12.18	23.51
46	BW	NP	-	N/A	NP	++	28.77	22.57
47	RP	NP	-	N/A	NP	(+)	35.17	23.43
48	NW	NP	-	N/A	NP	++	28.01	17.35
49	BY	NP	-	N/A	NP	+++	23.29	21.34
50	NW	NP	-	N/A	NP	++++	13.58	21.54
51	MV	NP	-	N/A	NP	+++	23.69	25.05
52	MV	NP	-	N/A	NP	+++	26.87	25.08
53	NI	NP	-	N/A	NP	+++	27.07	19.67
54	MV	NP	-	N/A	NP	+++	25.78	24.88
55	SN	NP	-	N/A	NP	+++	26.02	18.53
56	NI	NP	-	N/A	NP	+	33.62	22.00
57	SL	NP	(+)	35.01	NP	++	31.48	21.42
58	BW	NP	-	N/A	NP	+++	27.68	22.41
59	HE	NP	-	N/A	NP	++	30.38	21.20
60	BY	NP	-	N/A	NP	+	33.23	21.13
61	HH	NP	-	N/A	NP	(+)	35.10	22.19
62	BW	NP	-	N/A	NP	+	34.85	21.10
63	HE	NP	-	N/A	NP	++	31.57	21.56
64	BY	NP	-	N/A	NP	+	32.94	24.84
65	BW	NP	-	N/A	NP	+++	23.46	21.56
66	BY	NP	-	N/A	NP	++	30.27	24.78
67	SN	NP	-	N/A	NP	+++	23.39	23.43
68	BY	NP	-	N/A	NP	+++	23.27	24.64
69	BY	NP	-	N/A	NP	++++	5.97	20.77
70	NI	NP	-	N/A	NP	(+)	36.07	*17.81*
71	NY	NP	-	N/A	NP	+++	27.74	23.70
72	BW	NP	-	N/A	NP	++++	7.83	*19.13*
73	BW	NP	-	N/A	NP	+++	22.27	23.37
74	NI	NP	-	N/A	NP	++++	11.74	23.96
75	NW	NP	-	N/A	NP	++++	13.36	25.68
76	BB	NP	-	N/A	NP	++++	13.68	23.97
77	NW	NP	-	N/A	NP	++++	13.25	23.68
78	BB	NP	-	N/A	NP	++++	11.12	23.11
79	ST	NP	++++	16.45	NP	++++	11.53	23.13
80	NW	NP	(+)	35.62	NP	++++	9.83	25.00
81	BY	NP	-	N/A	NP	++++	12.00	25.53
82	NI	NP	-	N/A	NP	++++	11.42	24.22

NP: not performed
N/A: no Cq value

	**federal state**
BE	Berlin
BY	Bavaria
BB	Brandenburg
BW	Baden-Württemberg
HE	Hesse
HH	Hamburg
MV	Mecklenburg-West Pomerania
NI	Lower Saxony
NW	North Rhine-Westphalia
RP	Rhineland-Palatinate
SN	Saxony
ST	Saxony-Anhalt
SH	Schleswig-Holstein
SL	Saarland

TH	Thuringia

Table S5. Results of screening Italian honey bees from 2009, 2011, 2013, 2018 and 2020 by NGS (transcriptome) analysis

	year	sample	total number of reads	reads mapping on DWV (DWV-C included)	reads mapping on DWV (DWV-C excluded)	reads mapping on DWV-A	reads mapping on DWV-B
**Dataset 1**	2009	uninfested pool 1	11536226	1110888	1110888	1110888	0
		uninfested pool 2	16201184	917173	917172	917172	0
		uninfested pool 3	15251861	2442	2442	2442	0
		uninfested pool 4	18497764	14309	14309	14309	0
		uninfested pool 5	14257889	2071	2071	2071	0
		uninfested pool 6	17501781	1146	1146	1146	0
		**total reads**	**93246705.00**	**2048029.00**	**2048028.00**	**2048028.00**	**0.00**
		**total DWV-C reads**		1			
		mite-infested pool 1	16538777	1245925	1245925	1245925	0
		mite-infested pool 2	16784485	2063448	2063448	2063448	0
		mite-infested pool 3	13663436	2139109	2139109	2139107	2
		mite-infested pool 4	15456204	3381768	3381768	3381763	5
		mite-infested pool 5	14470976	25387	25387	25387	0
		**total reads**	**76913878**	**8855637.00**	**8855637.00**	**8855630**	**7**
		**total DWV-C reads**		0			
**Dataset 2**		uninfested bee 1	27338758	24581894	24490083	24439478	50605
		uninfested bee 2	18225222	140409	139766	139604	162
		uninfested bee 3	24683141	91893	91508	91379	129
		uninfested bee 4	19927120	65067	64837	64737	100
		uninfested bee 5	16706666	74394	74116	73965	151
		**total reads**	**106880907**	**24953657.00**	**24860310.00**	**24809163**	**51147**
		**total DWV-C reads**		93347			
		mite-infested bee 1	26007148	15788464	15746550	15655241	91309
		mite-infested bee 2	27712817	19703485	19644010	19624466	19544
		mite-infested bee 3	10804739	315161	314459	312790	1669
		mite-infested bee 4	15344334	9362012	9345969	9261359	84610
		mite-infested bee 5	27732490	82576	82374	81986	388
		mite-infested bee 6	21260599	18295895	18252915	18060353	192562
		mite-infested bee 7	33012513	2836339	2829168	2801257	27911
		mite-infested bee 8	30313176	26418753	26374137	26175384	198753
		mite-infested bee 9	25472838	195539	195072	193629	1443
		mite-infested bee 10	24363190	17240125	17233102	17130081	103021
		**total reads**	**242023844**	**110238349**	**110017756**	**109296546**	**721210**
		**total DWV-C reads**		220593			
**Dataset 3**	2011	uninfested bee 1	16585099	2545148	2544935	2538049	6886
		uninfested bee 2	18661501	1142493	1142388	1139158	3230
		uninfested bee 3	13668515	6401096	6399878	6380508	19370
		uninfested bee 4	64987582	63092	63043	62739	304
		uninfested bee 5	20691901	1477744	1477603	1473447	4156
		uninfested bee 6	27549466	43609	43587	43421	166
		uninfested bee 7	40532610	9655456	9654551	9651044	3507
		uninfested bee 8	27133067	7088572	7088125	7068622	19503
		uninfested bee 9	27589678	8196912	8196299	8173465	22834
		uninfested bee 10	26444038	7024101	7023453	7002948	20505
		uninfested bee 11	35507263	2001104	2000906	1995092	5814
		uninfested bee 12	30643697	52795	52775	52558	217
		**total reads**	**349994417**	**45692122**	**45687543**	**45581051**	**106492**
		**total DWV-C reads**		4579			
**Dataset 4**		uninfested bee 1	71305572	72484	72385	71984	401
		uninfested bee 2	15419572	8033	8025	7988	37
		uninfested bee 3	24120778	8671	8659	8618	41
		uninfested bee 4	17990726	10419	10405	10355	50
		uninfested bee 5	8111239	4919	4905	4889	16
		uninfested bee 6	42176770	15141	15126	15039	87
		uninfested bee 7	64845317	75898	75823	75412	411
		uninfested bee 8	29190286	13111	13093	13038	55
		**total reads**	**273160260**	**208676**	**208421**	**207323**	**1098**
		**total DWV-C reads**		255			
		mite-infested bee 1	13786900	4467251	4466823	4450683	16140
		mite-infested bee 2	20815189	7311308	7310205	7302671	7534
		mite-infested bee 3	89603735	59103402	59095591	58873136	222455
		mite-infested bee 4	29549793	8184379	8183918	8178226	5692
		mite-infested bee 5	36029093	4041408	4041234	4034433	6801
		mite-infested bee 6	9595069	595227	595200	593419	1781
		mite-infested bee 7	74198847	19901331	19894818	19866740	28078
		mite-infested bee 8	29049478	6747669	6747241	6726767	20474
		**total reads**	**302628104**	**110351975**	**110335030**	**110026075**	**308955**
		**total DWV-C reads**		16945			
**Dataset 5**	2013	uninfested bee 1	13467853	0	0	0	0
		uninfested bee 2	14117562	0	0	0	0
		uninfested bee 3	12358540	1	1	1	0
		uninfested bee 4	13743339	2	2	2	0
		uninfested bee 5	11857628	1	1	1	0
		**total reads**	**65544922**	**4**	**4**	**4**	**0**
		**total DWV-C reads**		0			
		mite-infested bee 1	65519	50327	50327	50327	0
		mite-infested bee 2	181002	115151	115151	115151	0
		mite-infested bee 3	147800	116213	116213	116213	0
		mite-infested bee 4	147595	109693	109693	109693	0
		mite-infested bee 5	147078	122643	122643	122643	0
		mite-infested bee 6	204784	168354	168354	168354	0
		mite-infested bee 7	160839	123704	123704	123704	0
		mite-infested bee 8	101776	77837	77837	77837	0
		mite-infested bee 9	231039	177524	177524	177524	0
		**total reads**	**1387432**	**1061446**	**1061446**	**1061446**	**0**
		**total DWV-C reads**		0			
**Dataset 6**	2018	uninfested bee 1	19141031	4847	4847	93	4754
		uninfested bee 2	16524486	5251	5251	1	5250
		uninfested bee 3	20462249	19311	19311	5	19306
		uninfested bee 4	16133716	663	663	0	663
		uninfested bee 5	16230611	151	151	4	147
		uninfested bee 6	17500546	6797	6797	645	6152
		uninfested bee 7	16749836	11886	11886	432	11454
		**total reads**	**122742475**	**48906**	**48906**	**1180**	**47726**
		**total DWV-C reads**		0			
**Dataset 7**	2020	uninfested bee 1	14431503	6419731	6419731	128157	6291574
		uninfested bee 2	17069982	1090	1090	9	1081
		uninfested bee 3	16067757	309	309	1	308
		uninfested bee 4	15482104	69	69	0	69

uninfested bee 5	15883722	366	366	0	366
uninfested bee 6	17927750	47	47	0	47
uninfested bee 7	14414577	360	360	0	360
uninfested bee 8	19214407	146	146	0	146
uninfested bee 9	15741775	815	815	1	814
uninfested bee 10	14283263	50	50	0	50
uninfested bee 11	17668010	290204	290204	486	289718
uninfested bee 12	16393828	1321	1321	29	1292
uninfested bee 13	17074188	9188	9188	14	9174
uninfested bee 14	19399753	48	48	0	48
**total reads**	**231052619**	**6723744**	**6723744**	**128697**	**6595047**
**total DWV-C reads**		0			
mite-infested bee 1	16919989	11638998	11638998	4291	11634707
mite-infested bee 2	16930615	11255	11255	44	11211
mite-infested bee 3	20376974	15787640	15787640	505044	15282596
mite-infested bee 4	14767139	2851	2851	11	2840
mite-infested bee 5	19222572	8936775	8936775	4	8936771
mite-infested bee 6	26633315	12649166	12649166	6	12649160
mite-infested bee 7	14745920	2538	2538	4	2534
mite-infested bee 8	21243366	16232426	16232426	626545	15605881
mite-infested bee 9	19561829	14749896	14749896	514674	14235222
mite-infested bee 10	16203254	11928140	11928140	18161	11909979
mite-infested bee 11	17846502	63094	63094	5	63089
mite-infested bee 12	19194956	12641150	12641150	42	12641108
mite-infested bee 13	20896155	13392559	13392559	719	13391840
mite-infested bee 14	14674535	9461771	9461771	1391	9460380
**total reads**	**259217121**	**127498259**	**127498259**	**1670941**	**125827318**
**total DWV-C reads**		0			
2018-20 total reads	613012215				
2009-2013 total reads	1618661376.00				
